# Purine–Hydrazone Scaffolds as Potential Dual EGFR/HER2 Inhibitors

**DOI:** 10.3390/ph18071051

**Published:** 2025-07-17

**Authors:** Fatemah S. Albalawi, Mashooq A. Bhat, Ahmed H. Bakheit, A. F. M. Motiur Rahman, Nawaf A. Alsaif, Alan M. Jones, Isolda Romero-Canelon

**Affiliations:** 1 School of Pharmacy, University of Birmingham, Edgbaston, Birmingham B15 2TT, UK; i.romerocanelon@bham.ac.uk; 2 Department of Pharmaceutical Chemistry, College of Pharmacy, King Saud University, Riyadh 11451, Saudi Arabia; mabhat@ksu.edu.sa (M.A.B.); abakheit@ksu.edu.sa (A.H.B.); afmrahman@ksu.edu.sa (A.F.M.M.R.); nalsaif@ksu.edu.sa (N.A.A.)

**Keywords:** cancer, EGFR, HER2, EGFR- and HER2-driven cancers, purine/hydrazone-containing compounds, lapatinib, erlotinib

## Abstract

**Background/Objectives:** The dual targeting of epidermal growth factor receptor (EGFR) and human epidermal growth factor receptor 2 (HER2) represents an effective approach for cancer treatment. The current study involved the design, synthesis, and biological evaluation of a new series of purine-containing hydrazones, **6**–**24** (**a**,**b**), as anticancer agents targeting EGFR and HER2 kinases. **Methods:** The proposed compounds were initially screened in silico using molecular docking to investigate their binding affinity to the active sites of EGFR and HER2 kinase domains. Subsequently, the compounds were synthesized and evaluated in vitro for their antiproliferative activity, using the MTT assay, against the various cancer cell lines A549, SKOV-3, A2780, and SKBR-3, with lapatinib as the reference drug. The most active derivatives were then examined to determine their inhibitory activity against EGFR and HER2 kinases. **Results:** Among the assessed compounds, significant antiproliferative activity was demonstrated by **19a**, **16b**, and **22b**. **19a** exhibited substantial anticancer efficacy against A549 and SKBR-3, with IC_50_ values of 0.81 µM and 1.41 µM, respectively. This activity surpassed lapatinib, which has an IC_50_ of 11.57 µM on A549 and 8.54 µM on SKBR-3 cells. Furthermore, **19a**, **16b**, and **22b** exhibited superior EGFR inhibitory efficacy compared with lapatinib (IC_50_ = 0.13 µM), with IC_50_ values of 0.08, 0.06, and 0.07 µM, respectively. Regarding HER2, **22b** demonstrated the greatest potency with an IC_50_ of 0.03 µM, equipotent to lapatinib (IC_50_ = 0.03 µM). Flow cytometry analysis of A549 cells treated with **19a** and **22b** indicated their ability to arrest the cell cycle during the G1 phase and to trigger cellular apoptosis. **Conclusions:** Compounds **19a**, **16b**, and **22b** represent intriguing candidates for the development of an anticancer agent targeting EGFR and HER2 kinases.

## 1. Introduction

Cancer remains a significant global public health issue and ranks as the second leading cause of mortality worldwide [1]. The International Agency for Research on Cancer (IARC) reported an estimated 20 million new cancer cases and 9.7 million cancer deaths worldwide in 2022 [2]. Despite significant advances in cancer treatment, limitations persist, including the lack of tumor cell selectivity, undesirable side effects, and the development of drug resistance, which can render conventional therapy ineffective against tumor cells [3,4]. Consequently, the discovery of novel small molecules with both potency and selectivity is an ongoing challenge in the field of drug development.

**Figure 1:**
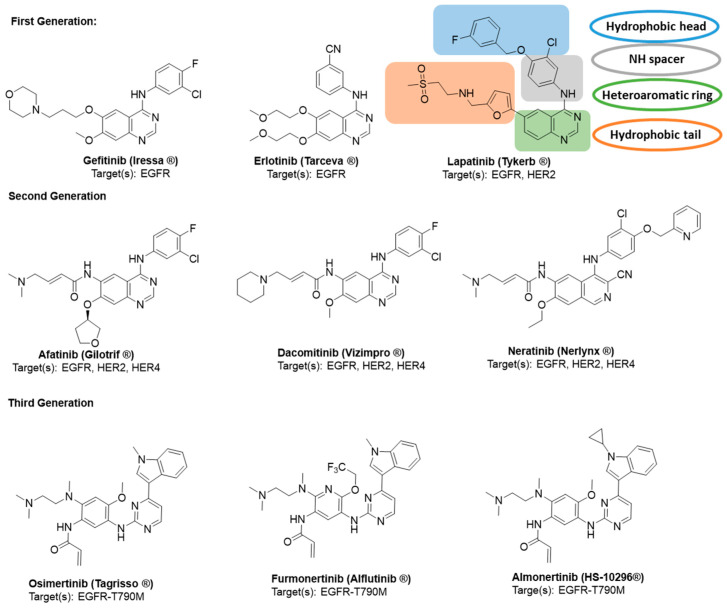
Examples of selective EGFR, dual EGFR/HER2, and mutant EGFR-T790M TKIs [21,26,27].

The dysregulation of receptor tyrosine kinases (RTKs), particularly the epidermal growth factor receptor (EGFR) and human epidermal growth factor receptor 2 (HER2), has been identified as a primary factor contributing to the development of diverse malignancies [5,6]. EGFR is a transmembrane glycoprotein belonging to the ErbB family of RTKs, and it plays a critical role in regulating intracellular signaling cascades through the phosphorylation of tyrosine residues within its kinase domain [7,8,9]. The regulatory effects of EGFR on cell proliferation, progression, and anti-apoptosis signals are mediated through the activation of the RAS-RAF-MAPK, PI3K-AKT, and STAT signaling pathways [10,11,12]. Abnormal stimulation of these signaling pathways, resulting from EGFR overexpression or mutation, leads to uncontrolled cell growth, proliferation, and increased cancer cell adhesion, motility, and metastasis [13,14,15]. EGFR overexpression has been extensively studied due to its involvement in the development of lethal cancers, including non-small-cell lung cancer (NSCLC), and head-and-neck, breast, colon, and ovarian cancer [16,17]. EGFR has been identified as a promising target for anticancer drug development, and EGFR tyrosine kinase inhibitors (TKIs) are used clinically (Figure 1) [18,19,20,21,22]. HER2, a kinase in the ErbB family, also plays a key role in the growth and development of aggressive types of cancer [13]. HER2 overexpression has been associated with poor prognosis and short survival in approximately 20–30% of breast cancer cases [23]. HER2 preferentially forms heterodimers with other members of the ErbB family, including EGFR, thereby activating a network of signaling pathways [24]. The HER2-containing heterodimer exhibits distinct characteristics, including prolonged endocytosis, slow ligand dissociation, reduced receptor degradation, and accelerated recycling. These characteristics contribute to enhanced downstream signaling [24,25]. Consequently, dual EGFR and HER2 inhibition is a promising strategy to potentiate EGFR signaling inhibition and block multiple signaling pathways mediated by HER2 heterodimers, potentially improving anticancer activity. Thus, the current work aims to develop new derivatives with potentially high anticancer activity and dual inhibition of EGFR and HER2 kinases.

Purine is a privileged core found in a diverse range of bioactive compounds, including protein kinase inhibitors [28,29,30,31,32,33,34,35,36,37,38,39,40,41]. Hydrazones have attracted significant interest in medicinal chemistry, in particular for their anticancer properties and kinase-inhibiting potential [42,43,44,45,46,47]. The present study hypothesizes that the hybridization of purine and hydrazone may yield compounds that have substantial anticancer properties through inhibiting both EGFR and HER2 kinases.

**Figure 2:**
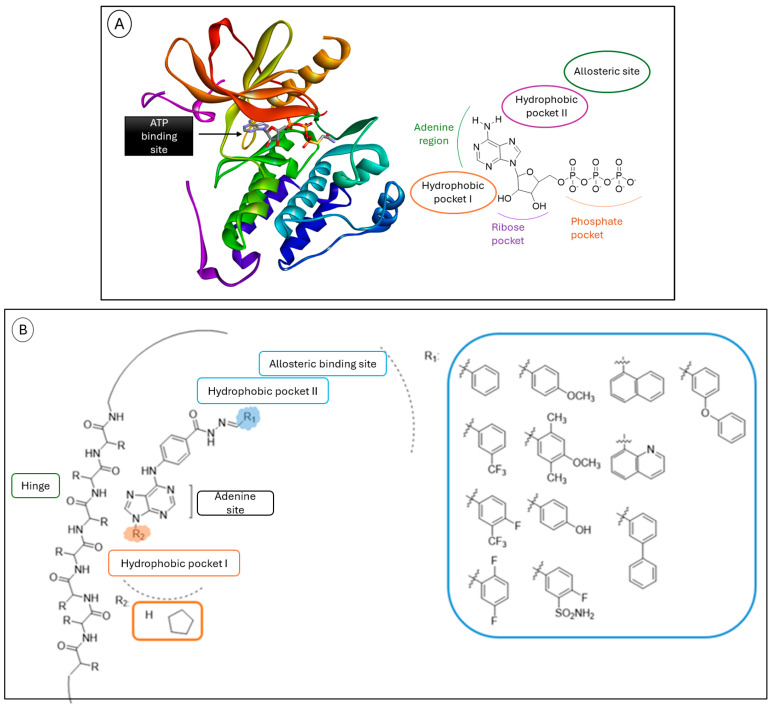
(**A**) Crystal structure of the EGFR kinase domain showing the ATP binding site (PDB ID: 2GS6) [49]; (**B**) the design strategy for purine-containing hydrazone analogs as dual EGFR/HER2-TKIs.

Generally, the ATP-binding site of EGFR can be divided into a set of regions (Figure 2): (1) the adenine binding pocket, which comprises key hydrogen bonds with the adenine ring; (2) the phosphate-binding region; and (3) the hydrophilic ribose pocket. Additionally, there are neighboring pockets to the ATP-binding area that are unoccupied by ATP but serve as targets for the ligand binding. These regions are primarily hydrophobic and can be categorized as hydrophobic pocket I, an allosteric binding site (back pocket), and hydrophobic pocket II [19,48].

The purine-containing hydrazone derivatives in this study have structural similarities to the FDA-approved EGFR-TKIs (Figure 1), along with the presence of the following: (i) A heterocyclic core, a purine nucleus has been utilized based on several considerations. Firstly, purine closely resembles the adenine found in ATP and has the potential to compete with ATP at its binding site in the TK domain. The nitrogen atoms in purine are predicted to participate in hydrogen bonding interactions with the amino acids in the hinge region, hence providing a strong binding affinity. Studies have reported numerous derivatives with a purine scaffold that exhibit significantly improved anticancer efficacy by blocking several receptor protein kinases [32,34,36]. (ii) The NH linker is proposed to interact with the amino acid residues located in the linker region. Studies have emphasized the crucial role of hydrazone linkers in enhancing the antiproliferative activity and improving the target selectivity of anticancer agents towards proteins such as kinases [44,45,46,50,51,52,53,54,55]. Consequently, hydrazones (–NH–N=CH–) have been chosen as linkers for designing the current molecules. (iii) A hydrophobic head (R_1_) is anticipated to bind with the hydrophobic pocket II via hydrophobic interactions, aiming to improve the binding affinity and hence enhance kinase inhibition efficacy. To identify the most appropriate hydrophobic head group that can fit into the hydrophobic pocket II of EGFR and HER2 and achieve the desired dual inhibitory activity, various hydrophobic moieties were introduced. This includes an unsubstituted phenyl group as well as a phenyl substituted with either electron-withdrawing or electron-donating substituents. These distinct groups will be incorporated to investigate the influence of electron-rich and electron-poor characteristics on binding affinity. Bulky hydrophobic groups, including fused aromatic and heterocyclic structures, and polycyclic ring systems (e.g., naphthyl and 3-biphenyl) were also studied to assess the impact of group rigidity vs. flexibility on binding affinity and interaction with the targeted proteins. (iv) A hydrophobic tail group (R_2_) is proposed to occupy hydrophobic region I of the kinase domain. Studies indicate that the incorporation of the cyclopentyl group at the *N*9 position of purine yields compounds with promising antiproliferative effects [31,35]. The structural activity relationship (SAR) study conducted by Yang et al. [32] indicates that among various *N*9 purine substituents (including methyl, cyclopropyl, isopropyl, cyclopentyl, cyclohexyl, phenyl, and 3,5-dimethylbenzene), compounds bearing a cyclopentyl moiety had the most significant antiproliferative activity and inhibitory potential against EGFR. Hence, a cyclopentyl group was chosen for insertion at the *N*9 position of the purine ring, as seen in compounds **6b**, **11b**, **13b**, and **16b**–**24b**.

In this study, a molecular docking analysis will first be conducted to determine the predicted binding affinity of the proposed compounds toward the active sites of EGFR and HER2 kinases. Next, the compounds will be synthesized and assessed for their antiproliferative activities against a range of human cancer cell lines. The derivatives demonstrating noticeable cytotoxic activity will be evaluated for their inhibition of EGFR and HER2-TK, followed by a further assessment of their ability to induce apoptosis and arrest the cell cycle. The most promising candidate will be selected as a representative example to illustrate its potential binding patterns in the active sites of EGFR and HER2 tyrosine kinases.

## 2. Results

### 2.1. In Silico Molecular Docking Study

A molecular docking study was carried out using AutoDock VINA (PyRx) and Discovery Studio Visualizer to explore the binding affinities and potential interactions of the proposed compounds with the active sites of EGFR (PDB ID: 1XKK) and HER2 (PDB ID: 3RCD).

Initially, a series of (*E*)-4-((9*H*-purine-6-yl)amino)-*N*′-(substituted)-(benzylidene/naphthylidene) benzohydrazide derivatives (**6a**–**20a** and **23a**) were docked into the active sites of EGFR and HER2. These derivatives incorporate a diversity of hydrophobic head groups (R_1_). Among these analogs, the highest binding affinities toward the target proteins were obtained by compounds **19a** and **23a** (see Table S1 in the Supplementary Materials). Table 1 presents the molecular docking scores for (*E*)-4-((9*H*-purin-6-yl)amino)-*N*′-([1,1′-biphenyl]-3-ylmethylene)benzohydrazide (**19a**) and (*E*)-4-((9*H*-purin-6-yl)amino)-*N*′-(naphthalen-1-ylmethylene)benzohydrazide (**23a**) toward the EGFR and HER2 active sites, using lapatinib as a reference compound. The data depicted in this table shows that **19a** displayed a score value of −10.3 kcal/mol toward the HER2 active site, suggesting a good affinity for HER2. Furthermore, this compound exhibits the highest affinity among the screened compounds for EGFR, with a binding score value of −11 kcal/mol. This value is comparable to that of lapatinib, which has an EGFR score value of −11.1 kcal/mol. On the other hand, **23a** demonstrated the greatest affinity for HER2, achieving a binding score of −10.9 kcal/mol, superior to that of lapatinib.

Subsequently, the impact of introducing the cyclopentyl group at the *N*9 position of purine was investigated through a molecular docking study. The binding scores of the compounds **6b**, **16b**, **17b**, **18b**, **19b**, **22b**, and **23b** toward the EGFR and HER2 active sites are presented in Table 2. The data in this table demonstrate an improvement in the binding affinity toward EGFR, increasing from −9.8 kcal/mol with compound **6b**, which has an unsubstituted phenyl head group, to −10.6 kcal/mol with compound **16b**, containing a 3-trifluoromethylphenyl group, and further to −11 kcal/mol with compounds **22b** and **17b**, which include a 3-sulfonamide-4-fluorophenyl and 3-trifluoromethyl-4-fluorophenyl moieties, respectively. Furthermore, **22b** demonstrates an excellent binding affinity for HER2, with a binding score of −10.8 kcal/mol, exceeding that of lapatinib, which has a binding score of −10.3 kcal/mol for HER2. The compound bearing a 3-biphenyl hydrophobic head group (**19b**) exhibited the most substantial increase in binding affinity towards EGFR, achieving a binding score of −11.7 kcal/mol. Additionally, the naphthyl-containing compound (**23b**) had the greatest affinity for HER2, with a binding score of −10.9 kcal/mol. Compared with **6b**, compound **18b** did not exhibit a noticeable rise in binding affinity toward EGFR and HER2. It showed a binding score value of −9.9 kcal/mol toward EGFR and HER2.

### 2.2. Synthesis

**Scheme 1:**
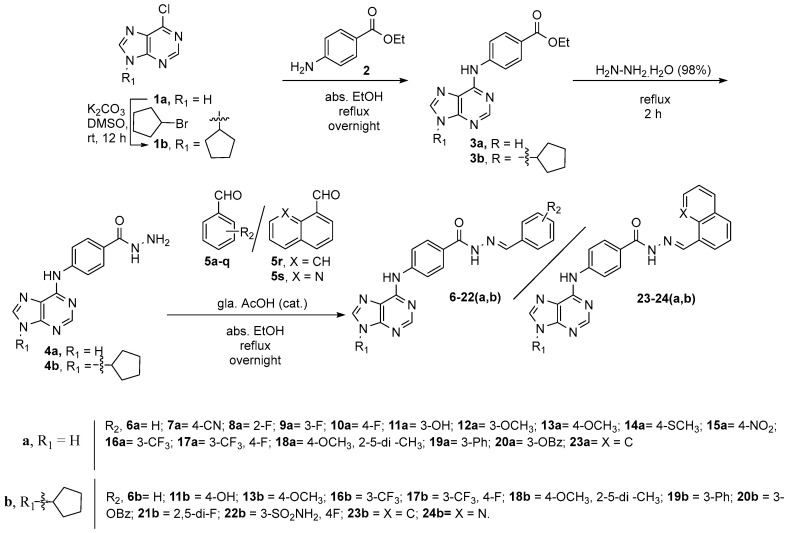
Synthesis of compounds **6**–**24** (**a**,**b**).

The synthesis of (*E*)-4-((7H-purin-6-yl) amino)-*N*′-(substituted)-benzylidenebenzohydrazide, **6a**–**20a**, and (*E*)-4-((7H-purin-6-yl)amino)-*N*′-(naphthalen-1-ylmethylene) benzohydrazide **23a** were accomplished using the synthetic procedure outlined in Scheme 1. The preparation of ethyl-4-((7H-purin-6-yl) amino) benzoate (**3a**) from 6-chloropurine (**1a**) and benzocaine (**2**) was carried out under reflux in absolute ethanol, resulting in an excellent yield (93%). Compound **3a** was then reacted with hydrazine hydrate (Caution: Combustible Liquid, Carcinogen, Target Organ Effect, Toxic by inhalation, Toxic by ingestion, Toxic by skin absorption, Skin sensitizer, Irritant, and Corrosive), obtaining 4-((7H-purin-6-yl) amino) benzohydrazide (**4a**). In the third step, compound **4a** was refluxed with variously substituted aldehydes (**5a**–**o**, **5r**) with a catalytic glacial acetic acid (0.2 mL). This yielded compounds **6a**–**20a** and **23a** with good to excellent yields (70–99%). To obtain **1b**, *N*-alkylation of the 9-position of compound **1a** was performed using cyclopentyl bromide in the presence of K_2_CO_3_ in dry DMSO. The second set of compounds (**6b**, **11b**, **13b**, and **16b**–**24b**) were obtained from **1b** in three steps following the procedure adopted for compound **6a**, with moderate to excellent yields (61–94%).

The synthesized compounds were subjected to structural elucidation using infrared (IR) spectroscopy, proton and carbon nuclear magnetic resonance (^1^H-NMR and ^13^C-NMR) analyses, and mass spectrometry (MS). Additionally, the melting points for the synthesized compounds were detected and indicate their purity. Characteristic IR bands corresponding to the benzylidene hydrazone (-N=CH-) group were observed as sharp peaks in the range of 1616–1635 cm^−1^ for all compounds. In the _1_H-NMR spectra, the disappearance of NH_2_ peaks, which appeared as a singlet at 4.45 and 4.46 ppm with two protons in compounds **4a** and **4b**, respectively, confirmed the conversion of compounds **4** (**a**,**b**) to compounds **6**–**24** (**a**,**b**). Notably, the characteristic three NH peaks for compounds **6a**–**20a** and **23a** were observed as singlets within the ranges of 13.21 to 13.29, 11.62 to 12.06, and 10.12 to 10.18 ppm. However, for compounds **6b**, **11b**, **13b**, and **16b**–**24b**, the two NH peaks were observed in the ranges of 12.02 to 11.54 and 10.22 to 10.18 ppm. Furthermore, a characteristic benzylidene proton peak was observed as a singlet between 8.34 and 8.37 ppm for all the derivatives. The structure of compound **1b** has been validated by two-dimensional NMR spectroscopy (including HSQC and HMBC) and by comparing its melting point and ^13^C-NMR data with the published literature values [56]. Finally, the HRMS mass spectral data confirmed the mass of all compounds.

### 2.3. In Vitro Cytotoxicity Assessment

The newly synthesized compounds **6**–**24** (**a**,**b**) were evaluated for their anticancer activities against a diverse panel of human cancer cell lines. The selected cell lines include A549, a lung cancer cell line that is characterized by its high expression of EGFR; SKOV-3, an ovarian cancer cell line, selected for its overexpression of both EGFR and HER2; and the breast cancer cell line (SKBR-3), characterized by HER2 overexpression. The selectivity of the compounds toward the target-expressed vs. low target-expressed cell lines was also investigated using another ovarian cancer cell line (A2780), which has relatively low HER2 expression. The MTT assay was carried out to assess the antiproliferative efficacy of the compounds by calculating the cell viability percentages for all derivatives, using erlotinib (an EGFR inhibitor) as a reference. Afterwards, the IC_50_ values for the most potent compounds were determined, and lapatinib (a dual EGFR/HER2 inhibitor) was used as a reference drug.

#### 2.3.1. Evaluation of the Cytotoxicity of Compounds **6a**–**20a** and **23a**

**Figure 3:**
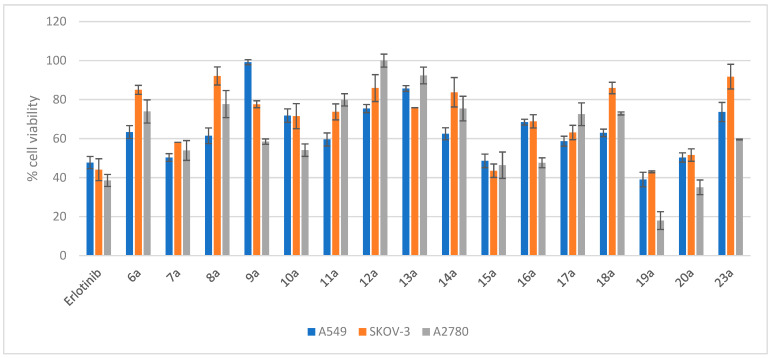
The percentage of cell viability of A549, SKOV-3, and A2780 cells exposed to a 10 μM concentration of **6a**–**20a** and **23a** for 48 h.

According to the findings shown in Figure 3, compounds **7a**, **15a**, and **17a** exhibit superior antiproliferative activity relative to **6a**. The percentage of cell viability resulting from exposure to 10 µM of the assessed compounds dropped from 63% in A549 cells and 85% in SKOV-3 cells with compound **6a**, to 50%, 49%, and 59% in A549 cells, and to 58%, 44%, and 63% in SKOV-3 cells with compounds **7a**, **15a**, and **17a**, respectively. Conversely, the cytotoxic effect toward A549 decreased with compounds **12a** and **13a,** resulting in cell viabilities of 75% and 86%, respectively. Compound **19a** exhibited the greatest efficacy among *N*9-unprotected purine derivatives, achieving cell survival percentages of 39% (A549), 43% (SKOV-3), and 18% (A2780) at a concentration of 10 μM. This performance surpasses that of erlotinib in A549 and A2780 (%cell viability = 48% and 39%, respectively) and is comparable in SKOV-3 (%cell viability = 44%). Furthermore, compound **20a** demonstrated good activity, with a cell survival percentage between 35% and 52%. Treatment with 10 μM of compound **23a** yielded lower potency, with cell viabilities of 74% and 92% for A549 and SKOV-3 cells, respectively.

#### 2.3.2. Evaluation of the Cytotoxicity of Compounds **6b**, **11b**, **13b**, and **16b**–**24b**

**Figure 4:**
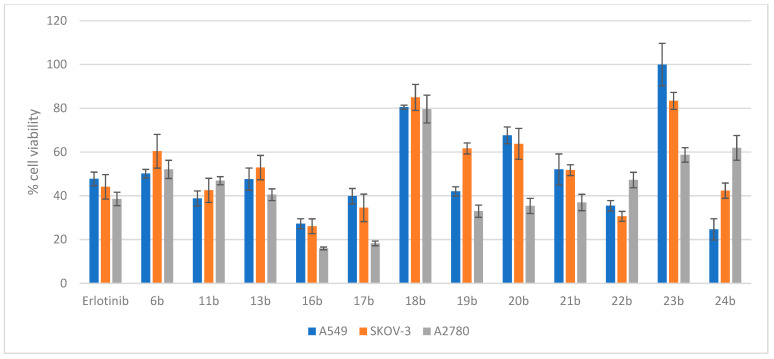
The percentage of cell viability of A549, SKOV-3, and A2780 cells exposed to a 10 μM concentration of **6b**, **11b**, **13b**, and **16b**–**24b** for 48h.

Figure 4 illustrates the percentage of cell viability of A549, SKOV-3, and A2780 following treatment with the *N*9-cyclopentyl purine derivatives (**6b**, **11b**, **13b**, and **16b**–**24b**). A 10 µM concentration of **6b** results in a cell viability percentage of 50% for A549, 60% for SKOV-3, and 52% for A2780. These values have decreased to 27% and 35% for A549, and 26% and 31% for SKOV-3, utilizing the same concentration of compounds **16b** and **22b**, respectively. Compounds **16b** and **22b** showed superior effectiveness compared to the reference drug erlotinib against A549 and SKOV-3. Moreover, the A2780 cell exhibited sensitivity to compound **16b**, showing a cell viability of 16% at a concentration of 10 µM. In comparison to **6b**, compounds **18b** and **23b** exhibited reduced activity, resulting in an 81–100% cell viability range for A549 and SKOV-3 at a concentration of 10 µM.

Cancer cell lines (A549, SKOV-3, and SKBR-3) and a non-cancerous cell line (MRC-5) were utilized to determine the IC_50_ values of the most potent compounds among *N*9-unsubstituted purine derivatives (**19a**), and *N*9-cyclopentyl substituted purine derivatives (**16b** and **22b**). Generally, compounds **19a**, **16b**, and **22b** exhibit promising anticancer efficacy against the selected cancer cell lines (see Table 3). The findings outlined in this table indicate that compound **19a** demonstrates a significant cytotoxicity against all three cancer cell lines, with the lowest IC_50_ values in A549 (IC_50_ = 0.81 ± 0.13 µM) and SKBR-3 (IC_50_ = 1.41 ± 0.36 µM) among the tested compounds and the reference drugs (lapatinib and erlotinib). Compounds **19a**, **16b**, and **22b** exhibit superior antiproliferative activity on SKOV-3 in comparison to the reference drugs, with IC_50_ values of 2.19 ± 0.16, 1.97 ± 0.07, and 1.54 ± 0.47 µM, respectively (lapatinib IC_50_ = 16.95 ± 2.57 µM and erlotinib IC_50_ = 5.18 ± 1.20 µM). Additionally, **19a**, **16b**, and **22b** demonstrate a selectivity index that exceeds that of lapatinib across all cell lines (lapatinib SI range = 2.60–5.14). Among the tested compounds, **19a** exhibits the highest selectivity, especially for A549 (SI = 64.60). Good selectivity has been obtained by **22b**, particularly for SKOV-3 (SI = 39.32). The lowest selectivity index among the tested compounds was achieved by **16b** (SI range = 8.51–18.55); however, it still demonstrates superior performance compared to lapatinib.

### 2.4. In Vitro EGFR and HER2 Kinase Inhibition Assays

The inhibitory activity of the compounds **19a**, **16b**, and **22b** against EGFR and HER2 kinases was then evaluated. In general, all the compounds that were tested exhibited substantial inhibition potency against EGFR and HER2. Table 4 highlights that **22b** exhibits the highest activity against HER2, with an IC_50_ of 0.03 µM, surpassing erlotinib (IC_50_ = 0.05 µM) and comparable to lapatinib (IC_50_ = 0.03 µM). Regarding EGFR, compounds **19a**, **16b**, and **22b** showed superior inhibitory potential relative to lapatinib (IC_50_ = 0.13 µM), with IC_50_ values of 0.08, 0.06, and 0.07 µM, respectively. However, in comparison to erlotinib (IC_50_ = 0.04 µM), the evaluated compounds exhibited lower potency toward EGFR.

### 2.5. Cell Cycle Analysis of Compounds ***19a*** and ***22b***

**Figure 5:**
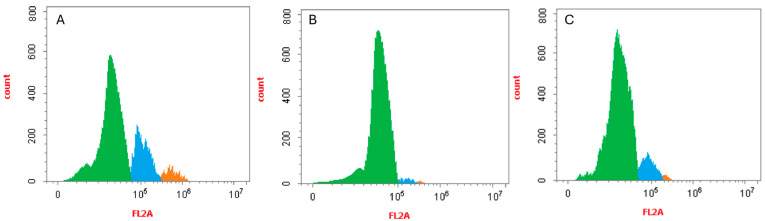
Flow cytometric analysis of cell cycle phases in A549 cells following treatment with compounds **19a** and **22b**. (**A**) control (untreated) A549 cell lines; (**B**) A549 cells treated with compound **19a**; (**C**) A549 cells treated with compound **22b**.

Cell cycle analysis was conducted on the A549 cell line following treatment with the most potent compounds, **19a** and **22b**. The assay results presented in Table 5 indicate a significant increase in the DNA content of cells in the G0–G1 phase, rising from 54.2% in untreated cells to 86.9% and 77.0% following treatment with the IC_50_ concentrations of compounds **19a** and **22b**, respectively. The percentage of DNA in the S phase was 31.4%, decreasing to 9.2% following treatment with **19a** and to 18.6% after exposure to **22b**. Compounds **19a** and **22b** demonstrate a notable reduction in the G2/M phase, decreasing from 14.3% in untreated cells to 3.9% and 4.4%, respectively. The cell cycle distribution index (CDI) was calculated based on the percentages of cells in each phase of the cell cycle. The CDI value was determined using the formula CDI = (G2/M + S)/(G0 − G1), where the values represent the percentages of cells in each phase. The CDI values for the control group and A549 cells treated with compounds **19a** and **22b** were 0.84, 0.15, and 0.29, respectively. A lower CDI value suggests a decreased rate of cell proliferation, implying that the cells may have undergone cell cycle arrest (Figure 5 and Table 5).

### 2.6. Apoptosis Analysis of Compounds ***19a*** and ***22b***

**Figure 6:**
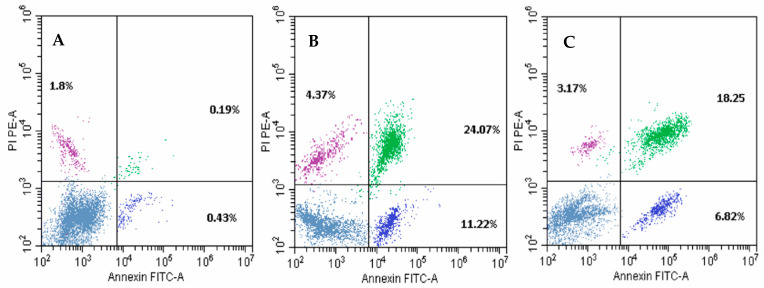
Flow cytometric analysis of apoptosis in A549 cells following treatment with compounds **19a** and **22b**. (**A**) control (untreated) A549 cell lines; (**B**) A549 cells treated with compound **19a**; (**C**) A549 cells treated with compound **22b**.

Flow cytometric evaluation using Annexin V/PI staining was carried out to assess the apoptotic induction capability of compounds **19a** and **22b** in A549 cells. Table 6 and Figure 6 demonstrates that treatment with **19a** and **22b** at their respective IC_50_ concentrations for 24 h resulted in a rise in the percentage of apoptotic cells. In the control (untreated) A549 cells, 97.62% of the cells were alive, with only 0.48% in early apoptosis, 0.17% in late apoptosis, and 1.73% undergoing necrosis. However, the treatment with compound **19a** significantly impacted the A549 cells, reducing the percentage of live cells to 62.30%. This was accompanied by a substantial increase in both early (9.72%) and late (23.13%) apoptosis. Compound **22b** exerted a milder, yet still notable, effect on the A549 cells. The viable cell population decreased to 72.51%, whereas early apoptosis increased to 5.92%, and late apoptosis reached 18.21%.

### 2.7. The Predicted Mode of Interaction of **19a** and **22b** Within the Active Site of EGFR and HER2

Compounds **19a** and **22b** were further investigated to elucidate the potential mechanism of binding to the EGFR and HER2 active sites.

**Figure 7:**
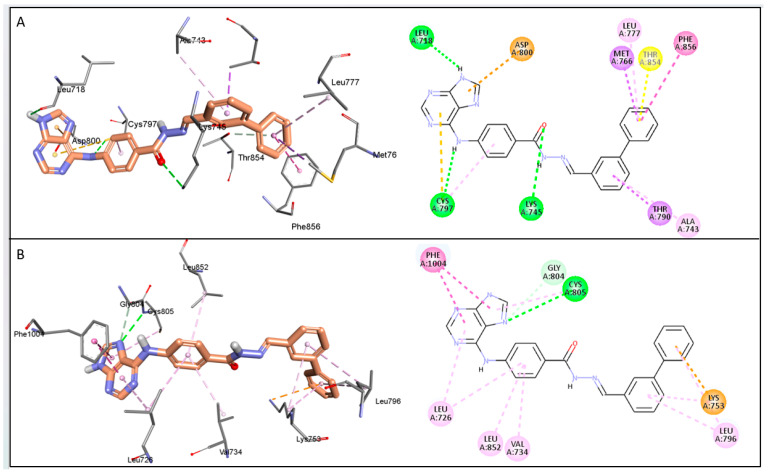
The anticipated interaction pattern of compound **19a** within the kinase domains of (**A**) EGFR and (**B**) HER2.

Compound **19a** was docked inside the active site of EGFR and HER2 kinase, giving binding scores of −11.0 and −10.3 kcal/mol, respectively (Table 1). Compound **19a** binds to the active site of EGFR by creating three hydrogen bonds with Lys745 (distance = 3.02 Å), Cys797 (distance = 2.78 Å), and Leu718 (distance = 2.87 Å). Furthermore, hydrophobic interactions were established with the amino acids Leu777, Asp800, Phe856, and Ala743 (Figure 7A). Compound **19a** engaged the ATP binding site of HER2 by forming a hydrogen bond with Cys805 (distance = 3.07 Å), and exhibited hydrophobic interactions with the amino acid residues Leu796, Val734, Leu852, Leu726, and Lys753 (Figure 7B).

**Figure 8:**
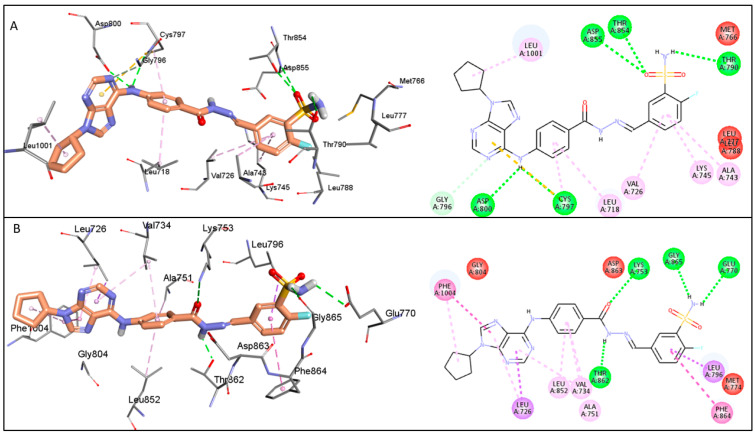
The anticipated interaction pattern of compound **22b** within the kinase domains of (**A**) EGFR and (**B**) HER2.

Figure 8 illustrates the predicted binding interactions between the EGFR or HER2 kinase domain and compound **22b**. It is apparent that **22b** occupies the EGFR active site by creating hydrogen bonds with Thr854 (distance = 3.15 Å), Asp800 (distance = 2.58 Å), Cys797 (distance = 3.08 Å), Asp855 (distance = 2.7 Å), and Thr790 (distance = 3.05 Å). Additionally, hydrophobic interactions have been established with Lys745, Ala743, Val726, and Leu718. Within the HER2 active site, **22b** demonstrated good binding interactions through the development of hydrogen bonds with Lys753 (distance = 3.11 Å), Thr862 (distance = 2.16 Å), Gly865 (distance = 2.48 Å), and Glu770 (distance = 2.66 Å), as well as hydrophobic interactions with the Phe864, Leu852, Val734, and Ala751 amino acid residues.

## 3. Discussion

In this study, a series of purine-containing hydrazone derivatives, **6**–**24** (**a**,**b**), were designed as potential anticancer agents targeting EGFR and HER2-TKs. These analogs exhibit structural similarities to the previously reported and clinically approved EGFR-TKIs, along with the presence of a heterocyclic core, spacer, hydrophobic head (R_1_), and hydrophobic tail (R_2_) group.

Initially, molecular docking studies were conducted to evaluate the binding affinities of mono-substituted purine derivatives (**6a**–**20a** and **23a**) toward the target proteins (EGFR and HER2). Those derivatives contain a variety of hydrophobic head groups (R_1_), including an unsubstituted phenyl, phenyl substituted with an electron-withdrawing group, and phenyl substituted with an electron-donating group, as well as bulky hydrophobic groups (e.g., naphthyl and 3-biphenyl). The aim of incorporating these groups is to identify the optimal hydrophobic head group that may efficiently occupy the hydrophobic pocket II of the EGFR and HER2 kinase domain and display a strong binding affinity to the target proteins, thereby exhibiting good kinase inhibition activity. According to the molecular docking results, among these derivatives, compounds **19a** and **23a** demonstrated the highest affinity for EGFR and HER2. Table 1 highlights the impact of the incorporation of a bulky hydrophobic group (R_1_) on the binding affinity toward the target proteins. Compared with **6a** (having an unsubstituted phenyl head group), the inclusion of a 3-biphenyl moiety (in compound **19a**) led to a noteworthy increase in binding affinity for EGFR, giving a binding score of −11.0 kcal/mol, with good affinity toward HER2 (Table 1). Compound **23a**, which contains a 1-naphthyl group, demonstrated the highest affinity for HER2 among these derivatives, with a score value of −10.9 kcal/mol. This indicates that the bulky nature of these groups may facilitate stronger binding interactions with EGFR and HER2. This is illustrated in Figure 7A, where the 3-biphenyl moiety in **19a** penetrates the hydrophobic pocket and interacts with Ala743. It further extends into the back pocket, generating hydrophobic interactions with the Leu777 and Met766 amino acid residues. Furthermore, a pi–pi (T-shaped) interaction has been established between this moiety and Phe856 in the DFG motif within the activation loop of the EGFR kinase domain.

In an attempt to optimize the binding affinity and interaction with EGFR and HER2, further derivatives have been constructed by inserting the cyclopentyl group at the *N*9 position of the purine ring, yielding compounds **6b**, **11b**, **13b**, and **16b**–**24b**. The inclusion of this group has been suggested with the aim of enhancing the binding affinity towards the EGFR and HER2 kinases via establishing a hydrophobic contact with the hydrophobic pocket in the kinase domain. This could contribute to the optimization of the compound’s inhibitory potential, hence enhancing the anticancer activity. Table 2 presents the docking scores for the selected *N*9-cyclopentyl-purine analog. Generally, it is apparent that this modification enhances the binding affinity to the target proteins in comparison with the free NH purine derivatives (refer to Tables S1 and S2 in the Supplementary Materials). The docking result indicates that the inclusion of the cyclopentyl group at *N*9 of purine could potentially improve the strength of the contact between the compounds and the target proteins. This has been shown in Figure 8, where the cyclopentyl moiety in compound **22b** establishes hydrophobic contact with the Leu1001 and Phe1004 amino acid residues in the active sites of EGFR and HER2, respectively.

A computational study aimed at identifying the optimal head group following incorporating the cyclopentyl tail group reveals that the addition of 4-methoxy-2,5-dimethyl to the phenyl ring (in compound **18b**) does not demonstrate a noteworthy rise in binding affinity for EGFR and HER2 when compared to the compound possessing an unsubstituted phenyl head group (**6b**) (refer to Table 2). Conversely, the compounds containing the 3-trifluoromethylphenyl group (**16b**) and 3-trifluoromethyl-4-fluorophenyl (**17b**) showed a notable improvement in binding affinity for EGFR, with binding scores of −10.6 and −11.0 kcal/mol, respectively. The results highlight the advantage of incorporating the electron-withdrawing groups into the phenyl ring (at R_1_) in improving the binding affinity of the proposed compounds for the target proteins. This observation could arise from the capability of **16b** and **17b** to create additional interaction forces with the hydrophobic pocket of the target protein, in contrast to compound **18b**. The interaction modes of **17b** and **18b** within the active site of EGFR have been illustrated in Figures S3 and S4 in the Supplementary Materials. It is noticeable that the hydrophobic head group in compound **17b** generates five forces of interaction, three of which are hydrophobic contacts between the phenyl ring and Lys745, Val726, and Ala743. The trifluoromethyl carbon establishes two additional interactions with the Met766 and Leu777 residues in the back pocket of EGFR. However, the replacement of this head group with 4-methoxy, 2,5-dimethylphenyl (in compound **18b**) leads to the loss of two forces of interaction with the Ala743 and Leu777 residues. Additionally, the purine ring and the NH aniline in compound **18b** are reoriented from the front pocket, resulting in the disruption of the two hydrogen bonds that had been established with **17b**. The first hydrogen bond was between the *N1* of purine and Cyst797, and the second appeared between the aniline NH and the Asp800 amino acid residue.

The addition of 3-sulfonamide-4-fluoro into the phenyl head group yielded **22b**, which exhibited a binding score of −11.0 kcal/mol toward EGFR, comparable to lapatinib, indicating a substantial binding affinity for EGFR. Furthermore, **22b** displayed an excellent binding affinity for HER2, with a binding score of −10.8 kcal/mol, surpassing that of lapatinib, which has a binding score of −10.3 kcal/mol for HER2. Figure 8 depicts the molecular mechanism that supports this observation. Compound **22b** aligns efficiently with the EGFR and HER2 binding sites. Aniline’s NH group forms two hydrogen bonds with the Asp800 and Cys797 residues in the EGFR kinase domain. In addition, the oxygen atom of the amide and NH group of the hydrazone form two hydrogen bonds in the HER2 active site: one with Thr862 in the hinge region and the other with Lys753. The sulfonamide group in compound **22b** enhances the binding affinity within the active sites of EGFR and HER2 by creating the major hydrogen bonding with the key amino acids in EGFR, including Thr854 (in the hinge region), Asp855 (in the DFG motif), and Thr790 (in the gatekeeper). Furthermore, sulfonamide establishes two hydrogen bonds with Glu770 and Gly865 (in the DFG motif) within the HER2 active site.

The insertion of the cyclopentyl at *N*9 of the purine ring in compound **19a** (which bears a 3-biphenyl head group) produces compound **19b**, which exhibits a higher binding affinity toward EGFR and HER2, attaining binding scores of −11.7 and −10.6 kcal/mol, respectively. On the other hand, **23b**, which contains a 1-naphthyl group, had the greatest affinity for HER2, demonstrating a binding score of −10.9 kcal/mol. The results obtained confirm the favorable effect of incorporating the bulky hydrophobic groups (at R_1_) in improving the binding affinity of the proposed compounds for the target proteins.

Compounds **6**–**24** (**a**,**b**) were synthesized according to a method outlined in Scheme 1 (in the Section 2.2), and the structural clarification was performed using IR spectroscopy, ^1^H-NMR and ^13^C-NMR analysis, and mass spectrometry (MS).

Subsequently, the synthesized compounds were evaluated for their antiproliferative efficacy against a varied panel of human cancer cell lines, which exhibited different levels of target protein (EGFR and HER2) expression. The results illustrated in Figure 3 and Figure 4 indicate that the synthesized compounds exhibit diverse anticancer activity against the selected cell lines. The initial objective was to investigate the cytotoxic effects of the *N*9-unprotected purine derivatives (**6a**–**20a** and **23a**), which possessed different substituents on R_1_ (the hydrophobic head group) to elucidate the structure–activity relationship (SAR). The results provided in Figure 3 indicate that incorporating electron-withdrawing groups (EWGs) into the phenyl ring at R_1_, including 4-cyano (**7a**), 4-nitro (**15a**), and 3-trifluoromethyl-4-fluoro (**17a**), enhances the antiproliferative activity toward A549 and SKOV-3 compared with the unsubstituted phenyl in compound (**6a**). Conversely, the phenyl substituted with electron-donating groups (EDGs), such as 3-methoxy (**12a**) and 4-methoxy-2,5-dimethyl (**18a**), exhibited no significant change or negative impacts on activity across the tested cell lines. Importantly, compound **19a**, containing a bulky hydrophobic head group (3-biphenyl), demonstrated the greatest potency among these derivatives, with cell survival percentages between approximately 18% and 43% at a concentration of 10 μM. It is noteworthy to mention that the meta-substituted phenyl ring (in compound **19a**) exhibited preferentially higher activity than the two fused aromatic rings (in **23a**). Figure 3 shows that the A549 and SKOV-3 cells exhibited cell viabilities of 74% and 92%, respectively, after treatment with 10 μM of compound **23a**. This observation may be attributed to the rotatability of the 3-biphenyl moiety, which provides conformational flexibility and improved adaptability of the molecule, resulting in more effective interactions with the active site of the target protein and increased binding affinity, thereby enhancing the antiproliferative activity against the cell line that overexpresses this protein. In contrast, the rigid planar structure of the naphthyl group has limited flexibility, which could result in steric hindrance and restrict its capacity to optimize the interactions with the active site of the target protein.

Afterwards, the impact of adding a cyclopentyl group at the *N*9 position of the purine ring on the compounds’ antiproliferative properties was explored. The findings of the cell viability investigation (shown in Figure 4) demonstrated that this modification generally afforded more potent compounds (**6b**, **11b**, **13b**, and **16b**–**24b**) than *N*9 unfunctionalized purine analogs, except for the compound that contained a 3-biphenyl hydrophobic head group (**19b**). A notable enhancement in cytotoxicity was observed following the incorporation of a cyclopentyl moiety into the compound containing a 3-trifluoromethylphenyl hydrophobic head group. The cell viability percentages of A549 and SKOV-3 decreased significantly from 68% and 69% (with **16a**, containing free *N*9-purine) to 27% and 26% (with **16b**, bearing *N*9-cyclopentyl moiety), respectively, after cells were treated with 10 μM of the compound. Hence, the impact of introducing a cyclopentyl group at the *N*9 position of the purine ring was found to be significant for the bioactivity of the synthesized molecules.

A similar trend (EWG > EDG) has been observed in the *N*9-cyclopentyl purine derivatives, which are compounds bearing EWGs on the phenyl ring at R_1_, exhibiting superior cell growth inhibition activity when compared to compounds containing EDGs (see Figure 4). The most substantial cytotoxic potency among the N9-cyclopentyl purine derivatives has been observed with compounds **16b** and **22b**. These findings agree with the results of the molecular docking study (see Table S2 in the Supplementary Materials).

The introduction of the cyclopentyl group at the *N*9 position of the purine ring in **19a**, the most potent compound among *N*9-unprotected derivatives, yielded compound **19b**. The molecular docking study shows that **19b** exhibited a greater binding affinity for EGFR and HER2 kinases in comparison to **19a** (refer to Table 1 and Table 2). Unexpectedly, **19b** demonstrated lower antiproliferative activity than **19a** (refer to Table 3). The possible reason for this may be the relative size of **19b** (compared to other analogs), which may restrict its ability to bind effectively within the active site of the kinase as a result of steric hindrance. In addition, one of the drawbacks of molecular docking is that it overestimates binding scores because of the increased number of contacts in larger molecules [57,58]. Furthermore, the poor membrane permeability of large molecules may obstruct their ability to reach their intended target in sufficient concentrations to produce biological effects [59,60]. Interestingly, among the most potent compounds, **22b** displays reduced growth inhibition activity against A2780, with a cell viability of 47%, in contrast to **19a** (cell viability = 18%) and **16b** (cell viability = 16%), at a concentration of 10 μM, suggesting the possible involvement of **19a** and **16b** in the inhibition of other critical signaling pathways. This also indicates that the selectivity of **22b** for EGFR- and HER2-overexpressing cell lines may be attributed to the ability of the sulfonamide group in **22b** to form multiple hydrogen bonds inside the kinase domains of EGFR and HER2 (see Figure 8). This hydrogen bond is often critical to maintaining the specificity of a ligand toward a particular protein. In the case of **19a**, 3-biphenyl forms hydrophobic interactions, which are often less selective than hydrogen bonds (Figure 7) [61].

After obtaining the cell survival results, the most active compounds, **19a**, **16b**, and **22b**, were selected to determine their IC_50_ value against three cancer cell lines, A549, SKOV-3, and SKBR-3 cells, as well as a normal human embryonic lung cell line (MRC-5). The cytotoxicity assessment results for compounds **19a**, **16b**, and **22b**, as presented in Table 3, demonstrated substantial activity. Compound **19a** exhibited IC_50_ values of 0.81 ± 0.13, 2.19 ± 0.16, and 1.41 ± 0.36 μM against A549, SKOV-3, and SKBR-3 cells, respectively, demonstrating remarkable efficacy in inhibiting cell proliferation. Furthermore, compound **19a** exhibited superior potency compared to the reference drugs lapatinib and erlotinib. The *N*9-cyclopentyl purine derivatives (**16b** and **22b**) also displayed notable cytotoxicity. Compound **16b** had IC_50_ values of 2.60 ± 0.35, 1.97 ± 0.07, and 4.30 ± 0.94 μM, while compound **22b** had IC_50_ values of 3.95 ± 0.61, 1.54 ± 0.47, and 3.80 ± 1.40 μM toward the A549, SKOV-3, and SKBR-3 cell lines, respectively. These values were superior to lapatinib and comparable to erlotinib (as shown in Table 3). The noticeable rise in activity after the incorporation of 3-trifluoromethyl-phenyl (in compound **16b**) can be linked to its capability for stronger binding through hydrophobic interactions within the kinase domain, along with the high lipophilic character of this group, which may enhance its permeability across the cell membrane, resulting in enhanced antiproliferative activity. The docking study correlated the enhanced activity of **22b** to its sulfonamide moiety, which served as a site for hydrogen bonding in the active regions of the EGFR and HER2 kinase domains (see Figure 8).

To further assess the selective cytotoxicity of the most promising compounds (**19a**, **16b**, and **22b**) toward cancer cells versus normal cells, they were evaluated on a normal human embryonic lung cell line (MRC-5). The data in Table 3 indicate that these compounds displayed reduced cytotoxicity towards MRC-5 cells in comparison to cancer cell lines, suggesting a favorable safety profile.

Following the initial screening to identify compounds with potential antiproliferative effects, compounds **19a**, **16b**, and **22b** were chosen for further investigation to elucidate their mechanism of action against cancer cells. The findings, presented in Table 4, showed that compounds **19a**, **16b**, and **22b** showed excellent inhibitory activity against EGFR kinase, surpassing the potency of lapatinib with IC_50_ values of 0.08, 0.06, and 0.07 μM, respectively (lapatinib’s IC_50_ = 0.13 μM). However, erlotinib demonstrated higher inhibitory potential against EGFR with an IC_50_ value of 0.04 μM. Additionally, compound **22b** (bearing a 3-sulfonamide-4-fluoro moiety) had the most promising suppression activity against HER2, with an IC_50_ value of 0.03 μM, which is comparable to lapatinib (IC_50_ = 0.03 μM). Conversely, the 3-trifluoromethyl-containing compound (**16b**) exhibited the highest inhibitory potential against EGFR (IC_50_ = 0.06 μM). Considering the results of the kinase inhibition study and the cytotoxicity evaluation, the results were in good agreement for compounds **16b** and **22b**. Compound **16b** exhibits greater cytotoxic activity than **22b** on A549 cells, which overexpress EGFR, with an IC_50_ value of 2.60 ± 0.35 μM for **16b** and 3.95 ± 0.61 μM for **22b**. Nevertheless, **22b** exhibited higher potency on SKBR-3 (which has a high level of HER2), with an IC_50_ value of 3.80 ± 1.40 μM (whereas **16b** had an IC_50_ of 4.30 ± 0.94 μM). Although **19a** displayed the highest cytotoxic efficacy against A549 and SKBR-3 cells, **16b** and **22b** demonstrated greater inhibitory activity in the kinase study, suggesting that **19a** may be engaged in multiple signaling pathways. These results suggest that compounds **19a**, **16b**, and **22b** exhibit potent inhibitory activity against both EGFR and HER2, making them a promising candidate series for further investigation as a potential anticancer agents.

Compounds **19a** and **22b** were chosen for further investigation to evaluate their impact on the progression of the cell cycle in the A549 cell line. The results, presented in Table 5, revealed an increase in the percentage of DNA content in the G0–G1 phase from 54.23% in the untreated control to 86.88% following treatment with compound **19a**. Conversely, the proportion of DNA content in the S phase and G2/M phase declined from 31.40% to 9.21%, and from 14.36% to 3.90%, respectively. These findings reveal that compound **19a** induced cell cycle arrest at the G1 phase. In the case of compound **22b**, 77.04% of cells were in the G0–G1 phase, 18.60% in the S phase, and 4.35% in the G2/M phase. This indicates that compound **22b** arrests the cell cycle at the G1 phase, exhibiting a less pronounced effect compared to compound **19a**.

An Annexin V-FITC/PI assay was performed to evaluate the capacity of compounds **19a** and **22b** to induce apoptosis in A549. Treatment with **19a** and **22b** at their IC_50_ concentrations led to a substantial increase in apoptotic cell percentage. As summarized in Table 6, in control A549 cells, 97.62% of the cells were alive. However, the treatment with compound **19a** significantly impacted the A549 cells, reducing the percentage of live cells to 62.3%. Compound **22b** exerted a milder, yet still notable, effect on the A549 cells, where the live cell population decreased to 72.51%. The increase in early and late cellular apoptosis confirmed that compounds **19a** and **22b** induced a pronounced apoptotic effect. Both tested compounds (**19a** and **22b**) appear to have a greater effect on triggering late-stage apoptosis.

The remarkable results achieved for **19a** and **22b**, with considerable cytotoxicity and kinase inhibition activity, along with apoptosis induction and cell cycle arrest, highlight their potential as prospective candidates for anticancer therapy.

Structure–activity relationship (SAR)

**Figure 9:**
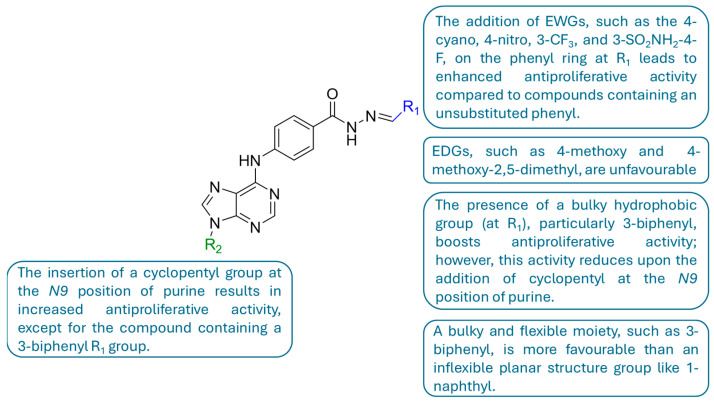
Structure–activity relationship of the targeted compounds.

The cytotoxicity study results indicated that the synthesised compounds showed diverse antiproliferative activity against the tested cell lines (the SAR has been illustrated in Figure 9). The incorporation of electron-withdrawing groups (EWGs) into the phenyl ring at R_1_, such as 4-cyano, 4-nitro, and 3-trifluoromethyl-4-fluoro, leads to enhanced antiproliferative activity compared to the unsubstituted phenyl-containing compound. Conversely, the phenyl substituted with electron-donating groups (EDGs), such as 3-methoxy and 4-methoxy-2,5-dimethyl, exhibited no significant change or negative impacts on activity. Importantly, the study’s findings highlight the beneficial effects of incorporating a bulky hydrophobic group (R_1_), in particular 3-biphenyl, on the cell growth inhibition potency. It is noteworthy to mention that the compound bearing a 3-biphenyl moiety exhibited preferentially higher antiproliferative activity compared to the compound containing two fused aromatic rings, e.g., 1-naphthyl. The insertion of a cyclopentyl group at the *N*9 position of the purine ring typically yielded more potent compounds compared to the unfunctionalized *N*9 purine analogs, with the exception of the compound that had a 3-biphenyl hydrophobic head group.

## 4. Materials and Methods

### 4.1. In Silico Molecular Docking Studies

**Figure 10:**
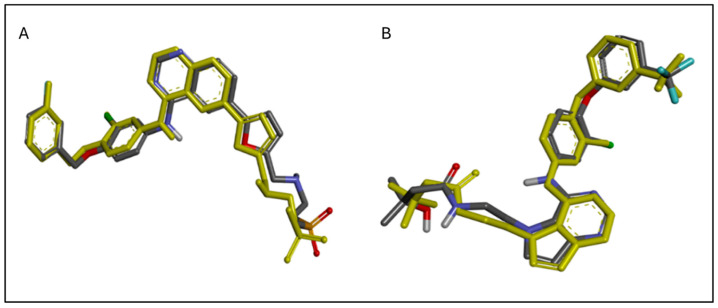
Three-dimensional illustration of the superimposition of the co-crystallized ligands (yellow) (**A**) lapatinib and (**B**) TAK-285, and the docked pose (gray) within the active site of (**A**) EGFR (PDB ID = 1XKK) and (**B**) HER2 (PDB ID: 3RCD).

The docking study was conducted using the AutoDock VINA (PyRx) virtual screening software (version 0.9.9), AutoDock Tools (version 1.5.7), and BIOVIA Discovery Studio Visualizer 2021 (version 24.1.0.23298). First, the kinase domains of EGFR and HER2 complexed with lapatinib (PDB ID: 1XKK) and TAK-285 (PDB ID: 3RCD), respectively, via refs. [62,63], were downloaded from the Protein Data Bank. Second, the protein and co-crystallized ligand were prepared by eliminating water molecules, small molecules, and ions, adding polar hydrogen atoms and charges, and energy minimization. Afterward, the docking setup was validated by self-docking of lapatinib within the binding site of EGFR, giving an energy score of −11.10 kcal/mol with an RMSD value (root mean square deviation) of 0.984 Å, and self-docking of TAK-285 within the binding site of HER2, giving an energy score of −10 kcal/mol with an RMSD value of 0.572 Å (Figure 10). Finally, all designed compounds were prepared and docked to the kinase domains of EGFR and HER2 to evaluate their binding affinities for the target proteins. Discovery Studio Visualizer was used to illustrate the anticipated interaction mechanism between the proposed compounds and target proteins.

### 4.2. Chemistry

All solvents and reagents utilized in the study were purchased from commercial suppliers (Thermo Fisher Scientific (Waltham, MA, USA), Sigma-Aldrich (St. Louis, MO, USA), and Tokyo Chemical Industry (Tokyo, Japan)), and used without further purification. The reaction progress was monitored using the pre-coated TLC sheets DC-Fertigplatten^®^ SIL G-25/UV254 (Macherey-Nagel, Düren, Germany) under UV light. Column chromatography was performed using Merck Silica Gel (Merck KGaA, Darmstadt, Germany) 60 (35–70) as the stationary phase. A Barnstead electrothermal digital melting point apparatus (model IA9100, BIBBY Scientific Limited, Staffordshire, UK) was used to determine the melting points. A Jasco FT/IR-6600 spectrometer (Tokyo, Japan) was used for recording infrared (IR) data. A Bruker 700 MHz nuclear magnetic resonance (NMR) spectrometer (Zurich, Switzerland) was used to obtain the NMR spectroscopic data. Chemical shifts are expressed as δ values (ppm) using tetramethyl silane (TMS) as the internal reference. Signals are indicated by the following abbreviations: s = singlet, d = doublet, t = triplet, q = quartet, p = pentet, m = multiple, br = broad, dd = doublet of doublet, ddd = doublet of doublet of doublet, td = triplet of doublet, dt = doublet of triplet, and qd = quartet of doublet. ^1^H-NMR, ^13^C-NMR, and 2D NMR were taken in deuterated dimethyl sulfoxide (DMSO-d_6_). Mass spectrometry used an Agilent 6320 ion trap mass spectrometer (MS) equipped with an ESI ion source (Agilent Technologies, Palo Alto, CA, USA) and Time-of-flight (TOF) MS, Waters, Xevo G2-XS.

Synthesis of 6-chloro-9-cyclopentyl-9H-purine (**1b**).

A mixture of 6-chloro-9H-purine (0.5 g, 3.2 mmol) and potassium carbonate (K_2_CO_3_, 1.3 g, 9.6 mmol) was stirred for 45 min at room temperature in anhydrous DMSO (20 mL) under a nitrogen atmosphere. Cyclopentyl bromide (1.6 mL, 16 mmol) was added dropwise, and stirring was continued at room temperature overnight (16 h). Upon completion of the reaction, as measured by TLC (n-hexane/ethyl acetate 3:2), the reaction mixture was poured into ice-cold water (30 mL) and extracted with ethyl acetate (3 × 20 mL). The combined organic layers were dried over Na_2_SO_4_, filtered, and the solvent was evaporated in vacuo. The crude product was purified by column chromatography (SiO_2_) using n-hexane/ethyl acetate (3:2) and gave a white solid (**1b**, 56%). R_f_ = 0.52 (n-hexane/ethyl acetate 3:2), Mp. 96–97 °C (literature = 95–98 °C) [56]. ^1^H-NMR (700 MHz, DMSO-d_6_) δ 8.79 (s, 1H), 8.78 (s, 1H), 5.00 (p, *J* = 7.5 Hz, 1H), 2.23–2.19 (m, 2H), 2.08–2.03 (m, 2H), 1.95–1.86 (m, 2H), and 1.77–1.68 (m, 2H) ppm. ^13^C-NMR (176 MHz, DMSO-d_6_) δ 152.2, 151.6, 149.4, 146.6, 131.7, 56.8, 32.2, and 24.0 ppm. Mass (ESI): *m*/*z* = 223 [M + H]^+^.

Synthesis of Ethyl-4-((7H-purin-6-yl)amino)benzoate (**3a**). A mixture of 6-chloro-7H-purine (**1**, 1.54 g, 10 mmol) and ethyl-4-aminobenzoate (**2**, 1.65 g, 10 mmol) in absolute ethanol (60 mL) was heated under reflux. The progression of the reaction was monitored using TLC. Upon completion of the reaction after 24 h, the reaction mixture was cooled to room temperature. The precipitated product was filtered under vacuum, washed with an excess of cold ethanol (3 × 30 mL), and dried overnight, yielding a pale-yellow solid (93%). Mp. 259–260 °C. FT-IR (KBr): ν (cm^−1^) = 2980, 2780, 1718, 1659, 1623, 1602, 1509, 1491, 1444, 1385, 1366, 1310, 1286, 1216, 1180, 1129, 1105, 1022, 943, 855, 765, 691, 615, and 549 cm^−1^. ^1^H-NMR (700 MHz, DMSO-d_6_) δ 11.63 (br s, 1H), 8.91 (s, 1H), 8.80 (s, 1H), 8.15 (d, *J* = 8.8 Hz, 2H), 8.00 (d, *J* = 8.8 Hz, 2H), 4.32 (q, *J* = 7.1 Hz, 2H), and 1.33 (t, *J* = 7.1 Hz, 3H) ppm. ^13^C-NMR (176 MHz, DMSO-d_6_) δ 165.7, 150.1, 149.9, 149.8, 143.5, 143.2, 130.6, 125.3, 120.4, 113.9, 61.0, and 14.7 ppm. Mass (ESI): *m*/*z* = 284 [M + H]^+^.

Synthesis of Ethyl-4-((9-cyclopentyl-9H-purin-6-yl)amino)benzoate (**3b**). A mixture of 6-chloro-9-cyclopentyl-purine (**1b**, 1.83 g, 8.2 mmol) and ethyl-4-aminobenzoate (1.35 g, 8.2 mmol) in absolute ethanol (60 mL) was refluxed for 24 h. The reaction progress was monitored by TLC. After completion of the reaction, the reaction mixture was cooled to room temperature (25 °C), and the precipitate obtained was filtered under vacuum, washed with an excess amount of cold ethanol (3 × 30 mL), and dried overnight, yielding a white solid (**3b**, 2.8 g, 97%). R_f_ = 0.39 (ethyl acetate: n-hexane; 7:3), Mp. 198–199 °C. ^1^H-NMR (700 MHz, DMSO-d_6_) δ 10.49 (s, 1H), 8.67 (s, 1H), 8.55 (s, 1H), 8.17 (d, *J* = 8.7 Hz, 2H), 7.95 (d, *J* = 8.7 Hz, 2H), 4.97 (p, *J* = 7.5 Hz, 1H), 4.31 (q, *J* = 7.1 Hz, 2H), 2.24–2.19 (m, 2H), 2.09–2.04 (m, 2H), 1.96–1.87 (m, 2H), 1.77–1.69 (m, 2H), and 1.33 (t, *J* = 7.1 Hz, 3H) ppm. ^13^C-NMR (176 MHz, DMSO-d_6_) δ 165.9, 152.1, 151.4, 150.1, 144.6, 141.3, 130.4, 123.8, 119.9, 119.8, 60.8, 56.4, 32.3, 24.0, and 14.7 ppm. Mass (ESI): *m*/*z* = 352 [M + H]^+^.

Synthesis of 4-((7H-Purin-6-yl) amino) benzohydrazide (**4a**).

4-((7H-Purin-6-yl)amino)benzohydrazide (**4a**) was prepared by refluxing ethyl-4-((7H-purin-6-yl) amino) benzoate (**3a**, 1 g, 3.50 mmol) in 98% hydrazine monohydrate (25 mL) for 2 h. The reaction mixture was cooled to room temperature, and the precipitate obtained was filtered under a vacuum, washed with an excess amount of cold water, and dried overnight, affording a white solid (74%). Mp. 379–380 °C. FT-IR (KBr): ν (cm^−1^) = 3316, 3190, 2976, 2818, 1625, 1613, 1504, 1470, 1348, 1320, 1235, 1179, 943, 897, 744, 675, 556, and 500 cm^−1^. ^1^H-NMR (700 MHz, DMSO-d_6_) δ 12.59 (brs, 1H), 10.03 (s, 1H), 9.64 (s, 1H), 8.45 (s, 1H), 8.34 (s, 1H), 8.08 (d, *J* = 8.3 Hz, 2H), 7.81 (d, *J* = 8.8 Hz, 2H), and 4.46 (s, 2H) ppm. ^13^C-NMR (176 MHz, DMSO-d_6_) δ 166.2, 152.1, 151.6, 143.0, 141.1, 127.9, 127.0, and 119.7 ppm. Mass (ESI): *m*/*z* = 270 [M + H]^+^.

Synthesis of 4-((9-Cyclopentyl-9H-purin-6-yl)amino)benzohydrazide (**4b**). A suspension of ethyl 4-((9-cyclopentyl-9H-purin-6-yl)amino)benzoate (**3b**, 1.5 g, 4.2 mmol) in 98% hydrazine monohydrate (30 mL) was heated to reflux for 2 h. The reaction mixture was cooled to room temperature, and the precipitate obtained was filtered under vacuum, washed with an excess amount of cold water (3 × 30 mL), and dried overnight, obtaining a white solid (1.23 g, 87%). Mp. 296–297 °C. ^1^H-NMR (700 MHz, DMSO-d_6_) δ 10.10 (s, 1H), 9.64 (s, 1H), 8.47 (s, 1H), 8.45 (s, 1H), 8.08 (d, *J* = 8.2 Hz, 2H), 7.81 (d, *J* = 6.8 Hz, 2H), 4.93 (p, *J* = 7.0 Hz, 1H), 4.45 (s, 2H, -NH_2_), 2.22–2.28 (m, 2H), 2.08–2.03 (m, 2H), 1.95–1.88 (m, 2H), and 1.76–1.69 (m, 2H) ppm. ^13^C-NMR (176 MHz, DMSO-d_6_) δ 166.1, 152.1, 151.9, 150.3, 142.9, 141.2, 127.8, 127.1, 120.9, 119.9, 56.1, 32.4, and 24.1 ppm. Mass (ESI): *m*/*z* = 338 [M + H]^+^.

General procedure for the preparation of **6**–**24** (**a**,**b**). A mixture of 4-((7H-purin-6-yl) amino) benzohydrazide (**4a**, 0.1 g, 0.372 mmol) and substituted aldehyde (1.2 equiv.) was dissolved in absolute ethanol (10 mL). A catalytic amount of glacial acetic acid (0.2 mL) was added to the reaction mixture and refluxed overnight. The precipitate formed was filtered, washed with cold ethanol (3 × 30 mL), and dried under a vacuum.

(*E*)-4-((7H-Purin-6-yl) amino)-*N*′-benzylidenebenzohydrazide (**6a**). White solid (99%), Mp. 304–305 °C. FT-IR (KBr): ν (cm^−1^) = 3370, 3296, 3104, 2972, 2812, 1649, 1631, 1616, 1586, 1535, 1509, 1466, 1416, 1397, 1360, 1317, 1286, 1242, 1197, 1119, 1088, 1042, 955, 935, 916, 904, 852, 826, 799, 749, 683, 648, 563, and 504 cm^−1^. ^1^H-NMR (700 MHz, DMSO-d_6_) δ 13.28 (brs, 1H), 11.75 (s, 1H), 10.15 (s, 1H), 8.48 (s, 2H), 8.35 (s, 1H), 8.18 (d, *J* = 8.3 Hz, 2H), 7.92 (d, *J* = 8.3 Hz, 2H), 7.75 (d, *J* = 7.3 Hz, 2H), and 7.49–7.44 (m, 3H) ppm. ^13^C-NMR (176 MHz, DMSO-d_6_) δ 163.2, 152.2, 151.9, 151.2, 147.6, 143.7, 140.9, 134.9, 130.4, 129.3, 128.7, 127.4, 126.9, 120.3, and 119.8 ppm. Mass (ESI): *m*/*z* = 358 [M + H]^+^; 380 [M + Na]^+^. HRMS (ESI): Calc. for C_19_H_16_N_7_O^+^, 358.1411; found 358.1425 [M + H]^+^.

(*E*)-4-((7H-Purin-6-yl) amino)-*N*′-(4-cyanobenzylidene)benzohydrazide (**7a**). White solid (92%), Mp. 333–334 °C. FT-IR (KBr): ν (cm^−1^) = 3515, 3359, 3196, 3096, 2992, 2814, 2562, 2219, 1671, 1633, 1590, 1573, 1532, 1503, 1480, 1414, 1359, 1297, 1276, 1238, 1190, 1142, 1088, 945, 934, 842, 679, 642, and 556 cm^−1^. ^1^H-NMR (700 MHz, DMSO-d_6_) δ 13.29 (brs, 1H), 12.00 (s, 1H), 10.17 (s, 1H), 8.53 (s, 1H), 8.48 (s, 1H), 8.36 (s, 1H), 8.18 (d, *J* = 7.8 Hz, 2H), and 7.93 (s, 6H) ppm. ^13^C-NMR (176 MHz, DMSO-d_6_) δ 166.2, 163.3, 152.1, 151.8, 151.3, 145.5, 144.0, 141.0, 139.4, 133.2, 128.8, 128.0, 126.6, 119.8, 119.1, and 112.2 ppm. Mass (ESI): *m*/*z* = 383 [M + H]^+^. HRMS (ESI): Calc. for C_20_H_15_N_8_O^+^, 383.1363; found 383.1378 [M + H]^+^.

(*E*)-4-((7H-Purin-6-yl)amino)-*N*′-(2-fluorobenzylidene)benzohydrazide (**8a**). White solid (88%), Mp. 312–313 °C. FT-IR (KBr): ν (cm^−1^) = 3427, 2085, 2816, 2559, 1684, 1635, 1613, 1588, 1501, 1480, 1466, 1413, 1357, 1270, 1239, 1187, 938, 753, and 645 cm^−1^. ^1^H-NMR (700 MHz, DMSO-d_6_) δ 13.29 (brs, 1H), 11.89 (s, 1H), 10.16 (s, 1H), 8.73 (s, 1H), 8.48 (s, 1H), 8.36 (s, 1H), 8.18 (d, *J* = 8.3 Hz, 2H), 7.98 (t, *J* = 7.6 Hz, 1H), 7.93 (d, *J* = 8.3 Hz, 2H), 7.51 (dd, *J* = 7.1 Hz, 1H), and 7.33–7.30 (m, 2H, Aromatic CH) ppm. ^13^C-NMR (176 MHz, DMSO-d_6_) δ 163.1, 161.2 (^1^*J*c–_F_ = 249.5 Hz), 152.2, 151.9, 151.2, 143.9, 140.9, 140.2, 132.3 (^3^*J*c–_F_ = 7.4 Hz), 128.7, 126.7 (^4^*J*c–_F_ = 2.6 Hz), 125.4, 122.4 (^3^*J*c–_F_ = 9.8 Hz), 120.3, 119.8, and 116.4 (^2^*J*c–_F_ = 20.6 Hz) ppm. Mass (ESI): *m*/*z* = 376 [M + H]^+^; 398 [M + Na]^+^. HRMS (ESI): Calc. for C_19_H_15_FN_7_O^+^, 376.1317; found 376.1333 [M + H]^+^.

(*E*)-4-((7H-Purin-6-yl)amino)-*N*′-(3-fluorobenzylidene)benzohydrazide (**9a**). White solid (85%), Mp. 339–340 °C. FT-IR (KBr): ν (cm^−1^) = 3392, 3220, 3096, 3063, 2978, 2813, 2549, 1644, 1624, 1591, 1525, 1505, 1480, 1415, 1358, 1325, 1277, 1240, 1189, 1122, 1066, 933, 909, 850, 791, 766, 789, 640, and 618 cm^−1^. ^1^H-NMR (700 MHz, DMSO-d_6_) δ 13.29 (brs, 1H), 11.87 (s, 1H), 10.16 (s, 1H), 8.48 (s, 2H), 8.35 (s, 1H), 8.18 (d, *J* = 8.4 Hz, 2H), 7.92 (d, *J* = 8.3 Hz, 2H), 7.59 (d, *J* = 7.6 Hz, 1H), 7.57–7.50 (m, 2H, Aromatic CH), and 7.29 (t, *J* = 7.1 Hz, 1H) ppm. ^13^C-NMR (176 MHz, DMSO-d_6_) δ 163.3, 162.9(^1^*J*c–_F_ = 243.7 Hz), 152.2, 151.9, 151.2, 146.2, 143.9, 140.9, 137.5 (^3^*J*c–_F_ = 7.6 Hz), 131.4 (^3^*J*c–_F_ = 8.3 Hz), 128.7, 126.7, 123.8, 120.3, 119.8, 117.1 (^2^*J*c–_F_ = 21.1 Hz), and 113.4 (^2^*J*c–_F_ = 22.6 Hz) ppm. Mass (ESI): *m*/*z* = 376 [M + H]^+^; 398 [M + Na]^+^. HRMS (ESI): Calc. for C_19_H_15_FN_7_O^+^, 376.1317; found 376.1331 [M + H]^+^.

(*E*)-4-((7H-Purin-6-yl)amino)-*N*′-(4-fluorobenzylidene)benzohydrazide (**10a**). White solid (94%), Mp. 324–325 °C. FT-IR (KBr): ν (cm^−1^) = 3466, 3394, 3220, 3072, 2795, 2562, 1645, 1631, 1593, 1574, 1537, 1508, 1486, 1469, 1414, 1352, 1301, 1246, 1229, 1210, 1191, 1155, 1081, 1068, 915, 837, 794, 643, and 608 cm^−1^. ^1^H-NMR (700 MHz, DMSO-d_6_) δ 13.28 (brs, 1H), 11.77 (s, 1H), 10.14 (s, 1H), 8.48 (s, 2H), 8.36 (s, 1H), 8.17 (d, *J* = 7.9 Hz, 2H), 7.92 (d, *J* = 8.3 Hz, 2H), 7.81 (t, *J* = 6.9 Hz, 2H-Aromatic CH), and 7.32 (dd, *J* = 8.5 Hz, 2H-Aromatic CH) ppm. ^13^C-NMR (176 MHz, DMSO-d_6_) δ 163.5 (^1^*J*c–_F_ = 248 Hz), 163.2, 152.1, 151.3, 151.1, 146.5, 143.7, 140.8, 131.5 (^4^*J*c–_F_ = 1.6 Hz), 129.6 (^3^*J*c–_F_ = 8.4 Hz), 128.7, 126.9, 120.3 (^2^*J*c–_F_ = 32 Hz), 119.8, and 116.3 (^2^*J*c–_F_ = 22 Hz) ppm. Mass (ESI): *m*/*z* = 376 [M + H]^+^; 398 [M + Na]^+^. HRMS (ESI): Calc. for C_19_H_15_FN_7_O^+^, 376.1317; found 376.1328 [M + H]^+^.

(*E*)-4-((7H-Purin-6-yl)amino)-*N*′-(3-hydroxybenzylidene)benzohydrazide (**11a**). White solid (70%), Mp. 336–337 °C. FT-IR (KBr): ν (cm^−1^) = 3406, 3240, 3076, 2762, 2676, 2585, 1644, 1632, 1596, 1580, 1525, 1504, 1486, 1463, 1415, 1327, 1271, 1240, 1194, 1118, 1070, 944, 849, 758, 683, and 640 cm^−1^. ^1^H-NMR (700 MHz, DMSO-d_6_) δ 13.28 (brs, 1H), 11.70 (s, 1H), 10.14 (brs, 1H), 9.64 (s, 1H), 8.48 (s, 1H), 8.38 (s, 1H), 8.36 (s, 1H), 8.17 (d, *J* = 8.2 Hz, 2H), 7.91 (d, *J* = 8.4 Hz, 2H), 7.27 (t, *J* = 7.8 Hz, 1H), 7.22 (s, 1H), 7.11 (d, *J* = 7.5 Hz, 1H), and 6.84 (dd, *J* = 8.1, 2.4 Hz, 1H) ppm. ^13^C-NMR (176 MHz, DMSO-d_6_) δ 163.1, 158.1, 152.2, 151.9, 151.2, 147.7, 143.7, 140.8, 136.2, 130.3, 128.7, 127.0, 120.3, 119.7, 119.2, 117.7, and 112.9 ppm. Mass (ESI): *m*/*z* = 374 [M + H]^+^; 396 [M + Na]^+^. HRMS (ESI): Calc. for C_19_H_16_N_7_O_2_^+^, 376.1360; found 374.1373 [M + H]^+^.

(*E*)-4-((7H-Purin-6-yl)amino)-*N*′-(3-methoxybenzylidene)benzohydrazide (**12a**). White solid (79%), Mp. 257–258 °C. FT-IR (KBr): ν (cm^−1^) = 3421, 3216, 3073, 2994, 2832, 2568, 1629, 1593, 1578, 1530, 1508, 1479, 1413, 1359, 1298, 1288, 1236, 1183, 1036, 932, 847, 793, 757, 689, and 647 cm^−1^. ^1^H-NMR (700 MHz, DMSO-d_6_) δ 13.25 (brs, 1H), 11.76 (s, 1H), 10.13 (brs, 1H), 8.48 (s, 1H), 8.45 (s, 1H), 8.36 (s, 1H), 8.16 (d, *J* = 8.3 Hz, 2H), 7.92 (d, *J* = 8.3 Hz, 2H), 7.39 (t, *J* = 7.9 Hz, 1H), 7.30 (d, *J* = 8.1 Hz, 1H), 7.29 (s, 1H), 7.03 (d, *J* = 7.8 Hz, 1H), and 3.83 (s, 3H) ppm. ^13^C-NMR (176 MHz, DMSO-d_6_) δ 163.2, 160.0, 152.1, 147.5, 143.7, 141.1, 136.4, 130.4, 128.7, 126.9, 120.4, 119.7, 116.5, 111.6, and 55.6 ppm. Mass (ESI): *m*/*z* = 388 [M + H]^+^; 410 [M + Na]^+^. HRMS (ESI): Calc. for C_20_H_18_N_7_O_2_^+^, 388.1516; found 388.1523 [M + H]^+^.

(*E*)-4-((7H-Purin-6-yl)amino)-*N*′-(4-methoxybenzylidene)benzohydrazide (**13a**). White solid (70%), Mp. 304–305 °C. FT-IR (KBr): ν (cm^−1^) = 3476, 3390, 3325, 3311, 3098, 2992, 2819, 2689, 2575, 1663, 1616, 1588, 1509, 1476, 1412, 1361, 1305, 1280, 1247, 1193, 1192, 1172, 1134, 1030, 934, 834, 796, 646, 578, and 514 cm^−1^. ^1^H-NMR (700 MHz, DMSO-d_6_) δ 13.27 (br, 1H), 11.62 (s, 1H), 10.12 (brs, 1H), 8.48 (s, 1H), 8.42 (s, 1H), 8.36 (s, 1H), 8.16 (d, *J* = 8.3 Hz, 2H), 7.91 (d, *J* = 8.3 Hz, 2H), 7.69 (d, *J* = 8.2 Hz, 2H), 7.04 (d, *J* = 8.2 Hz, 2H), and 3.83 (s, 3H) ppm. ^13^C-NMR (176 MHz, DMSO-d_6_) δ 163.0, 161.2, 152.2, 151.8, 151.3, 147.5, 143.6, 140.9, 129.0, 128.6, 127.5, 127.1, 119.8, 114.8, and 55.7 ppm. Mass (ESI): *m*/*z* = 388 [M + H]^+^; 410 [M + Na]^+^. HRMS (ESI): Calc. for C_19_H_15_FN_7_O_1_^+^, 388.1516; found 388.1525 [M + H]^+^.

(*E*)-4-((7H-Purin-6-yl)amino)-*N*′-(4-(methylthio)benzylidene)benzohydrazide (**14a**). Yellow solid (86%), Mp. 317–318 °C. FT-IR (KBr): ν (cm^−1^) = 3447, 3392, 3100, 2812, 1664, 1616, 1589, 1515, 1509, 1470, 1408, 1357, 1301, 1283, 1238, 1188, 937, 856, 672, and 507 cm^−1^. ^1^H-NMR (700 MHz, DMSO-d_6_) δ 13.28 (brs, 1H), 11.71 (s, 1H), 10.14 (s, 1H), 8.48 (s, 1H), 8.43 (s, 1H), 8.36 (s, 1H), 8.16 (d, *J* = 8.3 Hz, 2H), 7.91 (d, *J* = 8.3 Hz, 2H), 7.68 (d, *J* = 7.9 Hz, 2H), 7.34 (d, *J* = 8.0 Hz, 2H), and 2.53 (s, 3H) ppm. ^13^C-NMR (176 MHz, DMSO-d_6_) δ 163.1, 152.2, 151.9, 151.2, 147.2, 143.7, 141.2, 140.9, 131.3, 128.7, 127.9, 127.0, 126.1, 120.3, 119.8, and 14.7 ppm. Mass (ESI): *m*/*z* = 404 [M + H]^+^; 426 [M + Na]^+^. HRMS (ESI): Calc. for C_20_H_18_N_7_OS^+^, 404.1288; found 404.1302 [M + H]^+^.

(*E*)-4-((7H-Purin-6-yl)amino)-*N*′-(4-nitrobenzylidene)benzohydrazide (**15a**). Yellow solid (97%), Mp. 349–350 °C. FT-IR (KBr): ν (cm^−1^) = 3470, 3392, 3229, 3079, 2819, 1652, 1629, 1590, 1509, 1480, 1463, 1418, 1345, 1315, 1290, 1247, 1191, 940, 643, and 458 cm^−1^. ^1^H-NMR (700 MHz, DMSO-d_6_) δ 13.28 (br, 1H), 12.06 (s, 1H), 10.17 (brs, 1H), 8.57 (brs, 1H), 8.48 (s, 1H), 8.36 (s, 1H), 8.32 (d, *J* = 8.3 Hz, 2H), 8.18 (d, *J* = 6.9 Hz, 2H), 8.01 (d, *J* = 8.3 Hz, 2H), and 7.94 (d, *J* = 8.3 Hz, 2H) ppm. ^13^C-NMR (176 MHz, DMSO-d_6_) δ 163.4, 152.1, 151.9, 151.3, 148.2, 145.0, 144.0, 141.3, 140.9, 130.0, 129.0, 128.3, 126.5, 124.5, and 119.8 ppm. Mass (ESI): *m*/*z* = 403 [M + H]^+^; 425 [M + Na]^+^. HRMS (ESI): Calc. for C_19_H_15_N_8_O_3_^+^, 403.1262; found 403.1274 [M + H]^+^. HRMS (ESI): Cal. for C_19_H_15_N_8_O_3_^+^, 403.1262; found 403.1274 [M + H]^+^.

(*E*)-4-((7H-Purin-6-yl)amino)-*N*′-(3-(trifluoromethyl)benzylidene)benzohydrazide (**16a**). White solid (80%), Mp. 268–269 °C. FT-IR (KBr): ν (cm^−1^) = 3660, 3378, 3220, 3058, 2994, 2820, 2723, 2568, 1629, 1593, 1577, 1535, 1509, 1480, 1412, 1357, 1328, 1282, 1236, 1216, 1165, 1127, 1071, 932, 908, 849, 800, 759, 698, and 647 cm^−1^. ^1^H-NMR (700 MHz, DMSO-d_6_) δ 13.29 (brs, 1H), 11.96 (s, 1H), 10.17 (s, 1H), 8.56 (s, 1H), 8.48 (s, 1H), 8.36 (s, 1H), 8.19 (d, *J* = 8.4 Hz, 2H), 8.09 (s, 1H), 8.04 (d, *J* = 7.7 Hz, 1H), 7.93 (d, *J* = 8.3 Hz, 2H), 7.81 (d, *J* = 7.7 Hz, 1H), and 7.72 (t, *J* = 7.8 Hz, 1H) ppm. ^13^C-NMR (176 MHz, DMSO-d_6_) δ 163.4, 152.2, 151.9, 151.2, 145.8, 143.9, 140.9, 136.1, 131.5, 130.5, 130.1 (^2^*J*c–_F_ = 31.7 Hz), 128.8, 126.6, 126.8, 125.3, 122.9 (^1^*J*c–_F_ = 271.0 Hz), 120.3, and 119.8 ppm. Mass (ESI): *m*/*z* = 426 [M + H]^+^; 448 [M + Na]^+^; 464 [M + K]^+^. HRMS (ESI): Calc. for C_20_H_15_F_3_N_7_O^+^, 426.1285; found 426.1298 [M + H]^+^.

(*E*)-4-((7H-Purin-6-yl)amino)-*N*′-(4-fluoro-3-(trifluoromethyl)benzylidene) benzohydrazide (**17a**). White solid (80%), Mp. 307–308 °C. FT-IR (KBr): ν (cm^−1^) = 3222, 3081, 2994, 2822, 2568, 1658, 1626, 1593, 1577, 1529, 1501, 1479, 1413, 1359, 1323, 1283, 1242, 1163, 1138, 1125, 1053, 931, 849, and 645 cm^−1^. ^1^H-NMR (700 MHz, DMSO-d_6_) δ 13.28 (brs, 1H), 11.97 (s, 1H), 10.16 (s, 1H), 8.54 (s, 1H), 8.48 (s, 1H), 8.36 (s, 1H), 8.18 (d, *J* = 7.9 Hz, 2H), 8.13 (d, *J* = 6.9 Hz, 2H), 7.92 (d, *J* = 8.3 Hz, 2H), and 7.64 (dd, *J* = 8.8 Hz, 1H) ppm. ^13^C-NMR (176 MHz, DMSO-d_6_) δ 163.4, 159.8 (^1^*J*c–_F_ = 249.7 Hz, C-F), 152.1, 151.8, 151.3, 144.8, 143.9, 140.9, 134.0 (^3^*J*c–_F_ = 8.4 Hz), 132.3, 128.8, 126.6, 125.7 (^3^*J*c–_F_ = 3.5 Hz), 122.9 (^1^*J*c–_F_ = 272.3 Hz, CF_3_), 120.3, 119.7, 118.4 (^2^*J*c–_F_ = 20.8 Hz), and 117.6 (^2^*J*c–_F_ = 32.0 Hz) ppm. Mass (ESI): *m*/*z* = 444 [M + H]^+^; 466 [M + Na]^+^. HRMS (ESI): Calc. for C_20_H_14_F_4_N_7_O^+^, 444.1190; found 444.1208 [M + H]^+^.

(*E*)-4-((7H-Purin-6-yl)amino)-*N*′-(4-methoxy-2,5-dimethylbenzylidene) benzohydrazide (**18a**). Yellow solid (93%), Mp. 306–307 °C. FT-IR (KBr): ν (cm^−1^) = 3404, 3219, 3089, 2926, 2826, 1635, 1616, 1592, 1532, 1503, 1480, 1411, 1362, 1321, 1280, 1238, 1187, 1096, 931, 908, and 645 cm^−1^. ^1^H-NMR (700 MHz, DMSO-d_6_) δ 13.28 (brs, 1H), 11.58 (s, 1H), 10.13 (brs, 1H), 8.67 (s, 1H), 8.48 (s, 1H), 8.36 (s, 1H), 8.16 (d, *J* = 7.9 Hz, 2H), 7.92 (d, *J* = 8.2 Hz, 2H), 7.66 (s, 1H), 6.83 (s, 1H), 3.83 (s, 3H), 2.43 (s, 3H), and 2.16 (s, 3H) ppm. ^13^C-NMR (176 MHz, DMSO-d_6_) δ 162.8, 159.0, 152.2, 151.9, 151.3, 146.2, 143.6, 140.9, 136.8, 128.6, 128.1, 127.2, 124.7, 124.0, 119.8, 112.8, 55.8, 19.3, and 16.1 ppm. Mass (ESI): *m*/*z* = 416 [M + H]^+^; 438 [M + Na]^+^. HRMS (ESI): Calc. for C_22_H_22_N_7_O_2_^+^, 416.1829; found 416.1845 [M + H]^+^.

(*E*)-4-((7H-purin-6-yl)amino)-*N*′-([1,1′-biphenyl]-3-ylmethylene)benzohydrazide (**19a**). White solid (82%), Mp. 367–368 °C. FT-IR (KBr): ν (cm^−1^) = 3429, 3223, 2822, 1628, 1589, 1578, 1476, 1411, 1359, 1291, 1239, 1184, 930, 852, 754, 696, 644, and 500 cm^−1^. ^1^H-NMR (700 MHz, DMSO-d_6_) δ 13.26 (brs, 1H), 11.82 (s, 1H), 10.13 (s, 1H), 8.54 (s, 1H), 8.46 (s, 1H), 8.34 (s, 1H), 8.16 (d, *J* = 8.3 Hz, 2H), 7.97 (s, 1H), 7.91 (d, *J* = 8.3 Hz, 2H), 7.75 (d, *J* = 7.6 Hz, 1H), 7.73–7.69 (m, 3H-Aromatic CH), 7.56 (t, *J* = 7.7 Hz, 1H), 7.50 (t, *J* = 7.6 Hz, 2H), and 7.40 (t, *J* = 7.4 Hz, 1H) ppm. ^13^C-NMR (176 MHz, DMSO-d_6_) δ 163.2, 152.2, 151.9, 151.2, 147.4, 143.8, 141.2, 140.9, 140.1, 135.6, 130.0, 129.5, 128.7, 128.2, 127.3, 126.9, 126.4, 125.7, 120.3, and 119.8 ppm. Mass (ESI): *m*/*z* = 434 [M + H]^+^; 456 [M + Na]^+^. HRMS (ESI): Calc. for C_25_H_20_N_7_O^+^, 434.1724; found 434.1739 [M + H]^+^.

(*E*)-4-((7H-purin-6-yl) amino)-*N*′-(3-(benzyloxy) benzylidene) benzohydrazide (**20a**). White solid (78%), Mp. 258–259 °C. FT-IR (KBr): ν (cm^−1^) = 3059, 1635, 1487, 1371, 1284, 1184, 1048, 907, 843, 786, 726, 685, 637, and 604 cm^−1^. ^1^H-NMR (700 MHz, DMSO-d_6_) δ 13.21 (br, 1H), 11.77 (s, 1H), 10.12 (s, 1H), 8.48 (s, 1H), 8.45 (brs, 1H), 8.36 (s, 1H), 8.17 (d, *J* = 8.4 Hz, 2H), 7.92 (d, *J* = 8.2 Hz, 2H), 7.50 (d, *J* = 7.5 Hz, 2H), 7.45–7.31 (m, 6H), 7.10 (d, *J* = 8.1 Hz, 1H), and 5.18 (s, 2H) ppm. ^13^C-NMR (176 MHz, DMSO-d_6_) δ 163.2, 159.1, 152.1, 147.4, 143.8, 141.3, 137.4, 136.4, 130.4, 128.9, 128.7, 128.3, 128.1, 126.9, 120.4, 119.7, 117.1, 112.9, and 69.7 ppm. Mass (ESI): *m*/*z* = 464 [M + H]^+^. HRMS (ESI): Calc. for C_26_H_22_N_7_O_2_^+^, 464.1829; found 464.1846 [M + H]^+^.

(*E*)-4-((7H-Purin-6-yl)amino)-*N*′-(naphthalen-1-ylmethylene) benzohydrazide (**23a**). White solid (82%), Mp. 325–326 °C. FT-IR (KBr): ν (cm^−1^) = 3389, 3212, 3089, 3046, 3011, 2829, 2715, 2562, 1630, 1609, 1596, 1548, 1528, 1480, 1416, 1364, 1314, 1288, 1265, 1237, 1186, 1085, 933, 914, 845, 779, 761, 685, 640, and 604 cm^−1^. ^1^H-NMR (700 MHz, DMSO-d_6_) δ 13.29 (brs, 1H), 11.86 (s, 1H), 10.18 (s, 1H), 9.14 (s, 1H), 8.90 (d, *J* = 8.5 Hz, 1H), 8.50 (s, 1H), 8.37 (s, 1H), 8.21 (d, *J* = 8.3 Hz, 2H), 8.04 (dd, *J* = 8.5, 4.2 Hz, 2H), 7.98 (d, *J* = 8.1 Hz, 2H), 7.96 (d, *J* = 7.1 Hz, 1H), 7.70 (t, *J* = 7.7 Hz, 1H), and 7.63 (q, *J* = 6.9 Hz, 2H) ppm. ^13^C-NMR (176 MHz, DMSO-d_6_) δ 163.1, 152.2, 151.9, 151.2, 147.4, 143.8, 140.9, 134.0, 130.9, 130.6, 130.2, 129.2, 128.7, 128.0, 127.7, 126.9, 126.7, 126.0, 124.7, 120.3, and 119.9 ppm. Mass (ESI): *m*/*z* = 408 [M + H]^+^. HRMS (ESI): Calc. for C_23_H_18_N_7_O^+^, 408.1567; found 408.1584 [M + H]^+^.

(*E*)-*N*′-benzylidene-4-((9-cyclopentyl-9H-purin-6-yl)amino)benzohydrazide (**6b**). White solid (94%), Mp. 263–264 °C. ^1^H-NMR (700 MHz, DMSO-d_6_) δ 11.75 (s, 1H), 10.20 (s, 1H), 8.50 (s, 1H), 8.48 (s, 1H), 8.47 (s, 1H), 8.17 (d, *J* = 8.3 Hz, 2H), 7.92 (d, *J* = 8.3 Hz, 2H), 7.75 (d, *J* = 7.3 Hz, 2H), 7.52–7.40 (m, 3H), 4.95 (p, *J* = 7.6 Hz, 1H), 2.23–2.18 (m, 2H), 2.10–2.05 (m, 2H), 1.96–1.89 (m, 2H), and 1.78–1.69 (m, 2H) ppm. ^13^C-NMR (176 MHz, DMSO-d_6_) δ 163.1, 152.0, 151.9, 150.3, 147.6, 143.6, 141.3, 134.9, 130.4, 129.3, 128.7, 127.4, 127.0, 121.0, 119.9, 56.1, 32.4, and 24.1 ppm. Mass (ESI): *m*/*z* = 426 [M + H]^+^. HRMS (ESI): Cal. for C_24_H_24_N_7_O^+^, 426.2037; found 426.2049 [M + H]^+^.

(*E*)-4-((9-cyclopentyl-9H-purin-6-yl)amino)-*N*′-(4-hydroxybenzylidene)benzohydrazide (**11b**). White solid (62%), Mp. 184–185 °C. ^1^H-NMR (700 MHz, DMSO-d_6_) δ 11.54 (s, 1H), 10.18 (s, 1H), 9.92 (s, 1H), 8.49 (s, 1H), 8.46 (s, 1H), 8.36 (s, 1H), 8.15 (d, *J* = 7.4 Hz, 2H), 7.90 (d, *J* = 7.4 Hz, 2H), 7.57 (d, *J* = 7.8 Hz, 2H), 6.85 (d, *J* = 8.0 Hz, 2H), 4.94 (p, 1H), 2.22–2.18 (m, 2H), 2.09–2.04 (m, 2H), 1.92–1.90 (m, 2H), and 1.74–1.71 (m, 2H) ppm. ^13^C-NMR (176 MHz, DMSO-d_6_) δ 162.9, 159.7, 152.0, 151.9, 150.3, 148.0, 143.4, 141.3, 129.2, 128.5, 127.3, 125.9, 121.0, 119.9, 116.1, 56.1, 32.4, and 24.0 ppm. Mass (ESI): *m*/*z* = 442 [M + H]^+^. HRMS (ESI): Calc. for C_24_H_24_N_7_O_2_^+^, 442.1986; found 442.1999 [M + H]^+^.

(*E*)-4-((9-cyclopentyl-9H-purin-6-yl)amino)-*N*′-(4-methoxybenzylidene)benzohydrazide (**13b**). White solid (76%), Mp. 249–250 °C. ^1^H-NMR (700 MHz, DMSO-d_6_) δ 11.62 (s, 1H), 10.19 (s, 1H), 8.49 (s, 1H), 8.46 (s, 1H), 8.42 (s, 1H), 8.16 (d, *J* = 8.3 Hz, 2H), 7.90 (d, *J* = 8.2 Hz, 2H), 7.69 (d, *J* = 8.2 Hz, 2H), 7.04 (d, *J* = 8.1 Hz, 2H), 4.94 (p, *J* = 7.7 Hz, 1H), 3.82 (s, 3H), 2.29–2.18 (m, 2H), 2.09–2.04 (m, 2H), 1.93– 1.88 (m, 2H), and 1.75–1.70 (m, 2H) ppm. ^13^C-NMR (176 MHz, DMSO-d_6_) δ 163.0, 161.2, 152.0, 151.9, 150.3, 147.5, 143.5, 141.3, 129.0, 128.6, 127.5, 127.2, 121.0, 119.9, 114.8, 56.1, 55.7, 32.4, and 24.0 ppm. Mass (ESI): *m*/*z* = 556 [M + H]^+^. HRMS (ESI): Calc. for C_25_H_26_N_7_O_2_^+^, 456.2142; found 456.2148 [M + H]^+^.

(*E*)-4-((9-cyclopentyl-9H-purin-6-yl)amino)-*N*′-(3-(trifluoromethyl)benzylidene) benzohydrazide (**16b**). White solid (77%), Mp. 242–243 °C. ^1^H-NMR (700 MHz, DMSO-d_6_) δ 11.96 (s, 1H), 10.22 (s, 1H), 8.56 (s, 1H), 8.50 (s, 1H), 8.47 (s, 1H), 8.18 (d, *J* = 8.2 Hz, 2H), 8.09 (s, 1H), 8.04 (d, *J* = 7.4 Hz, 1H), 7.93 (d, *J* = 8.2 Hz, 2H), 7.81 (d, *J* = 7.7 Hz, 1H), 7.72 (t, *J* = 7.8 Hz, 1H), 4.95 (p, *J* = 7.9 Hz, 1H), 2.23–2.18 (m, 2H), 2.09–2.04 (m, 2H), 1.96–1.89 (m, 2H), and 1.77–1.69 (m, 2H) ppm. ^13^C-NMR (176 MHz, DMSO-d_6_) δ 163.4, 152.0, 151.9, 150.4, 145.8, 143.8, 141.3, 136.1, 131.5, 130.5, 130.1 (^2^*J*_C–F_ = 32 Hz), 128.8, 126.8 (^3^*J*_C–F_ = 2 Hz), 126.6, 124.5 (^1^*J*c–_F_ = 272 Hz), 123.3 (^3^*J*_C–F_ = 4 Hz), 121.0, 119.9, 56.1, 32.4, and 24.1 ppm. Mass (ESI): *m*/*z* = 494 [M + H]^+^. HRMS (ESI): Calc. for C_25_H_23_F_3_N_7_O_1_^+^, 494.1911; found 494.1926 [M + H]^+^.

(*E*)-4-((9-cyclopentyl-9H-purin-6-yl)amino)-*N*′-(4-fluoro-3-(trifluoromethyl) benzylidene) benzohydrazide (**17b**). White solid (61%), Mp. 256–257 °C. ^1^H-NMR (700 MHz, DMSO-d_6_) δ 11.97 (s, 1H), 10.21 (s, 1H), 8.54 (brs, 1H), 8.50 (s, 1H), 8.47 (s, 1H), 8.18 (d, *J* = 8.3 Hz, 2H), 8.13 (brs, 2H), 7.92 (d, *J* = 8.2 Hz, 2H), 7.64 (dd, *J* = 8.8 Hz, 1H), 4.94 (p, *J* = 7.6 Hz, 1H), 2.23–2.18 (m, 2H), 2.09–2.04 (m, 2H), 1.93–1.90 (m, 2H), and 1.76–1.70 (m, 2H) ppm. ^13^C-NMR (176 MHz, DMSO-d_6_) δ 163.4, 159.8 (^1^*J*_C–F_ = 257 Hz), 152.0, 151.9, 150.4, 144.8, 143.8, 141.3, 134.0 (^3^*J*_C–F_ = 8 Hz), 132.2 (^3^*J*_C-F_ = 3 Hz), 128.8, 126.7, 125.7 (^3^*J*_C–F_ = 4 Hz), 122.9 (^1^*J*_C–F_ = 271 Hz), 121.0, 119.9, 118.4 (^2^*J*_C–F_ = 21 Hz), 117.6 (^2,3^*J*_C–F_ = 33 & 13 Hz), 56.1, 32.4, and 24.0 ppm. Mass (ESI): *m*/*z* = 512 [M + H]^+^. HRMS (ESI): Calc. for C_25_H_22_F_4_N_7_O^+^, 512.1816; found 512.1836 [M + H]^+^.

(*E*)-4-((9-cyclopentyl-9H-purin-6-yl)amino)-*N*′-(4-methoxy-2,5-dimethylbenzylidene) benzohydrazide (**18b**) White solid (93%), Mp. 287–288 °C. ^1^H-NMR (700 MHz, DMSO-d_6_) δ 11.57 (s, 1H), 10.18 (s, 1H), 8.67 (s, 1H), 8.49 (s, 1H), 8.46 (s, 1H), 8.16 (d, *J* = 8.3 Hz, 2H), 7.91 (d, *J* = 8.2 Hz, 2H), 7.67 (s, 1H), 6.83 (s, 1H), 4.94 (p, *J* = 7.6 Hz, 1H), 3.83 (s, 3H), 2.44 (s, 3H), 2.23–2.19 (m, 2H), 2.16 (s, 3H), 2.09–2.04 (m, 2H), 1.94–1.89 (m, 2H), and 1.75–1.71 (m, 2H) ppm. ^13^C-NMR (176 MHz, DMSO-d_6_) δ 162.8, 159.0, 152.0, 151.9, 150.3, 146.2, 143.5, 141.3, 136.8, 128.5, 128.1, 127.3, 124.7, 124.0, 121.0, 119.9, 112.8, 56.1, 55.8, 32.4, 24.0, 19.3, and 16.1 ppm. Mass (ESI): *m*/*z* = 484 [M + H]^+^. HRMS (ESI): Calc. for C_27_H_30_N_7_O_2_^+^, 484.2455; found 484.2466 [M + H]^+^.

(*E*)-*N*′-((1,1′-biphenyl)-3-ylmethylene)-4-((9-cyclopentyl-9H-purin-6-yl)amino)benzohydrazide (**19b**). White solid (82%), Mp. 223–224 °C. ^1^H-NMR (700 MHz, DMSO-d_6_) δ 11.84 (s, 1H), 10.21 (s, 1H), 8.56 (s, 1H), 8.50 (s, 1H), 8.47 (s, 1H), 8.18 (d, *J* = 8.3 Hz, 2H), 7.99 (s, 1H), 7.93 (d, *J* = 8.1 Hz, 2H), 7.78–7.373 (m, 4H-Aromatic CH), 7.58 (t, *J* = 7.6 Hz, 1H), 7.52 (t, *J* = 7.6 Hz, 2H), 7.43 (t, *J* = 7.4 Hz, 1H), 4.95 (p, *J* = 7.2 Hz, 1H), 2.23–2.19 (m, 2H), 2.09–2.04 (m, 2H), 1.96–1.89 (m, 2H), and 1.78–1.71 (m, 2H) ppm. ^13^C-NMR (176 MHz, DMSO-d_6_) δ 163.2, 152.0, 151.9, 150.3, 147.4, 143.7, 141.3, 141.2, 140.1, 135.6, 130.0, 129.5, 128.7, 128.2, 127.2, 127.0, 126.4, 125.7, 121.0, 119.9, 56.1, 32.4, and 24.1 ppm. Mass (ESI): *m*/*z* = 502 [M + H]^+^. HRMS (ESI): Calc. for C_30_H_28_N_7_O^+^, 502.2350; found 502.2370 [M + H]^+^.

(*E*)-*N*′-(3-(benzyloxy)benzylidene)-4-((9-cyclopentyl-9H-purin-6-yl)amino) benzohydrazide (**20b**). White solid (71%), Mp. 214–215 °C. ^1^H-NMR (700 MHz, DMSO-d_6_) δ 11.76 (s, 1H), 10.20 (s, 1H), 8.49 (s, 1H), 8.47 (s, 1H), 8.44 (s, 1H), 8.17 (d, *J* = 8.2 Hz, 2H), 7.91 (d, *J* = 8.2 Hz, 2H), 7.50 (d, *J* = 7.5 Hz, 2H), 7.46–7.29 (m, 6H-Aromatic CH), 7.10 (d, *J* = 8.0 Hz, 1H), 5.18 (s, 2H), 4.95 (p, *J* = 7.7 Hz, 1H), 2.23–2.18 (m, 2H), 2.09–2.04 (m, 2H), 1.96–1.87 (m, 2H), and 1.79–1.69 (m, 2H) ppm. ^13^C-NMR (176 MHz, DMSO-d_6_) δ 173.4, 159.1, 152.0, 151.9, 150.3, 147.4, 141.3, 137.4, 136.4, 130.4, 128.9, 128.7, 128.3, 128.1, 127.0, 121.0, 120.4, 119.9, 117.1, 112.9, 69.7, 56.1, 32.4, and 24.1 ppm. Mass (ESI): *m*/*z* = 532 [M + H]^+^. HRMS (ESI): Calc. for C_31_H_30_N_7_O_2_^+^, 532.2455; found 532.2476 [M + H]^+^.

(*E*)-4-((9-cyclopentyl-9H-purin-6-yl)amino)-*N*′-(2,5-difluorobenzylidene) benzohydrazide (**21b**). White solid (75%), Mp. 289–290 °C. ^1^H NMR (700 MHz, DMSO-d_6_) δ 11.98 (s, 1H), 10.22 (s, 1H), 8.68 (brs, 1H), 8.50 (s, 1H), 8.47 (s, 1H), 8.18 (d, *J* = 8.4 Hz, 2H), 7.93 (d, *J* = 6.9 Hz, 2H), 7.64 (s, 1H), 7.42–7.39 (m, 1H), 7.36 (brs, 1H), 4.94 (p, *J* = 7.5 Hz, 1H), 2.23–2.19 (m, 2H), 2.11–2.03 (m, 2H), 1.95–1.88 (m, 2H), and 1.77–1.70 (m, 2H). ^13^C NMR (176 MHz, DMSO-d_6_) δ 163.2, 158.8 (^1^*J*_C–F_ = 241 Hz), 157.3 (^1^*J*_C–F_ = 246 Hz), 152.0, 151.9, 150.4, 143.9, 141.3, 139.2, 128.8, 126.6, 124.1 (^2^*J*_C–F_ = 12 and ^3^*J*_C–F_ = 8 Hz), 121.0, 119.9, 118.8 (^2^*J*_C–F_ = 25 and ^3^*J*_C–F_ = 12 Hz), 118.4 (^2^*J*_C–F_ = 24 and ^3^*J*_C–F_ = 9 Hz), 112.0 (^2^*J*_C–F_ = 26 and ^3^*J*_C–F_ = 3 Hz), 56.1, 32.4, and 24.0 ppm. Mass (ESI): *m*/*z* = 462 [M + H]^+^. HRMS (ESI): Calc. for C_24_H_22_F_2_N_7_O^+^, 462.1848; found 462.1862 [M + H]^+^.

(*E*)-5-((2-(4-((9-cyclopentyl-9H-purin-6-yl)amino)benzoyl)hydrazineylidene)methyl)-2-fluorobenzenesulfonamide (**22b**). White solid (80%), Mp. 244–245 °C. ^1^H-NMR (700 MHz, DMSO-d_6_) δ 11.89 (s, 1H), 10.21 (s, 1H), 8.51 (s, 1H), 8.50 (s, 1H), 8.47 (s, 1H), 8.22 (d, *J* = 6.7 Hz, 1H), 8.18 (d, *J* = 8.3 Hz, 2H), 7.97 (s, 1H), 7.92 (d, *J* = 8.2 Hz, 2H), 7.81 (s, 2H), 7.54 (dd, *J* = 9.1 Hz, 1H), 4.94 (p, *J* = 7.7 Hz, 1H), 2.23–2.18 (m, 2H), 2.09–2.04 (m, 2H), 1.95–1.89 (m, 2H), and 1.77–1.70 (m, 2H) ppm. ^13^C-NMR (176 MHz, DMSO-d_6_) δ 163.3, 159.0 (^1^*J*_C–F_ = 256 Hz), 152.0, 151.9, 150.4, 145.2, 143.8, 141.3, 133.7, 132.7 (^2^*J*_C–F_ = 15.2 Hz), 131.6, 128.7, 126.8, 126.4, 121.0, 119.9, 118.2 (^2^*J*_C–F_ = 22 Hz), 56.1, 32.4, and 24.0 ppm. Mass (ESI): *m*/*z* = 523 [M + H]^+^. HRMS (ESI): Calc. for C_24_H_24_FN_8_O_3_S^+^, 523.1671; found 523.1686 [M + H]^+^.

(*E*)-4-((9-cyclopentyl-9H-purin-6-yl)amino)-*N*′-(naphthalen-1-ylmethylene)benzohydrazide (**23b**). White solid (80%), Mp. 251–252 °C. ^1^H-NMR (700 MHz, DMSO-d_6_) δ 11.85 (s, 1H), 10.22 (s, 1H), 9.13 (s, 1H), 8.90 (d, *J* = 8.5 Hz, 1H), 8.51 (s, 1H), 8.48 (s, 1H), 8.20 (d, *J* = 8.3 Hz, 2H), 8.04 (br, 2H), 7.97–7.95 (m, 3H-Aromatic CH), 7.70 (dd, *J* = 7.9 Hz, 1H), 7.64–7.61 (m, 2H-Aromatic CH), 4.95 (p, *J* = 7.3 Hz, 1H), 2.22–2.19 (m, 2H), 2.11–2.04 (m, 2H), 1.96–1.89 (m, 2H), and 1.76–1.70 (m, 2H) ppm. ^13^C-NMR (176 MHz, DMSO-d_6_) δ 163.1, 152.0, 151.9, 150.4, 147.4, 143.7, 141.3, 134.0, 130.9, 130.6, 130.1, 129.2, 128.7, 128.0, 127.7, 127.0, 126.7, 126.0, 124.7, 121.0, 120.0, 56.1, 32.4, and 24.1 ppm. Mass (ESI): *m*/*z* = 476 [M + H]^+^. HRMS (ESI): Calc. for C_28_H_26_N_7_O^+^, 476.2193; found 476.2213 [M + H]^+^.

(*E*)-4-((9-cyclopentyl-9H-purin-6-yl)amino)-*N*′-(quinolin-8-ylmethylene)benzohydrazide (**24b**). White solid (87%), Mp. 263–264 °C. ^1^H-NMR (700 MHz, DMSO-d_6_) δ 12.02 (s, 1H), 10.21 (s, 1H), 9.79 (s, 1H), 9.02 (s, 1H), 8.51 (s, 1H), 8.47 (s, 2H), 8.42 (d, *J* = 7.2 Hz, 1H), 8.18 (d, *J* = 8.3 Hz, 2H), 8.10 (d, *J* = 8.0 Hz, 1H), 8.00 (d, *J* = 8.1 Hz, 2H), 7.75 (t, *J* = 7.7 Hz, 1H), 7.65 (dd, *J* = 8.4, 4.1 Hz, 1H), 4.95 (p, *J* = 7.7 Hz, 1H), 2.22–2.18 (m, 2H), 2.09–2.04 (m, 2H), 1.95–1.87 (m, 2H), and 1.76–1.69 (m, 2H) ppm. ^13^C-NMR (176 MHz, DMSO-d_6_) δ 163.1, 152.0, 151.9, 150.8, 150.3, 145.8, 144.5, 143.7, 141.3, 137.1, 131.8, 130.3, 128.8, 128.5, 127.0, 126.9, 126.0, 122.3, 121.0, 119.9, 56.1, 32.4, and 24.1 ppm. Mass (ESI): *m*/*z* = 477 [M + H]^+^. HRMS (ESI): Calc. for C_27_H_25_N_8_O^+^, 477.2146; found 477.2165 [M + H]^+^.

### 4.3. Biological Evaluation

#### 4.3.1. Materials and Consumables

The RPMI-1640 and McCoy’s 5A culture mediums, penicillin/streptomycin concentrate, fetal bovine serum (FBS), trypsin-EDTA, phosphate-buffered saline (PBS), 3-(4,5-dimethylthiazol-2-yl)-2,5-Diphenyltetrazolium Bromide (MTT), and dimethyl sulfoxide (DMSO) used in biological experiments were purchased from Sigma Aldrich (St. Louis, MO, USA). Trypsin, propranolol, and penicillin/streptomycin were purchased from Thermo Fischer. Sterile consumables and plasticware for the cell culture, including flasks, well plates, petri dishes, serological pipettes, and other sterile disposables, were purchased from Gibco (Waltham, MA, USA), Star Labs (Washington, DC, USA), and CoStar (Washington, DC, USA).

#### 4.3.2. Cell Maintenance

The in vitro cell survival/cytotoxicity (IC_50_) of compounds **6**–**24** (**a**,**b**) was evaluated against a panel of cancer cell lines, including A549, SKOV-3, A2780, and SKBR-3, as well as a normal cell line, MRC-5. The cell lines utilized in this study were obtained from the American Type Culture Collection (ATCC) and grown using RPMI-1640 (for A549, SKOV-3, and A2780) or McCoy’s 5A (for SKBR-3) media supplemented with 10% *v*/*v* fetal bovine serum and 1% *v*/*v* penicillin/streptomycin antibiotics (20.000 units). Cells were passaged regularly at around 80% confluency using Triplex and maintained in 75 cm^2^ flasks at 37 °C in a humidified atmosphere containing 5% CO_2_.

#### 4.3.3. Instrumentation and Data Processing

Absorbance measurements were carried out in 96-well plates using a FLUOStar Omega microplate reader (BMG LABTECH, Ortenberg, Germany), with the resultant data analyzed using Microsoft Excel and GraphPad Prism 10.2.3. Flow cytometry was carried out using a Beckman Coulter Cytoflex (Beckman Coulter, Inc., Brea, CA, USA), and data were processed using FlowJo_v10.10.0.

#### 4.3.4. In Vitro Cytotoxicity Assay (MTT)

Approximately 10 × 10^3^ cells were seeded per well of the 96-well plates using drug-free media and incubated for 24 h. The media was subsequently removed, and the cells were exposed to either fresh media as a negative control, lapatinib as a positive control, or the compounds under assessment, with concentrations ranging from 0.01 to 100 μM in culture media. After 48 h of drug exposure, the supernatant was aspirated, and the cells were washed with 100 μL of PBS. The MTT assay was performed by creating a stock solution of MTT at a concentration of 5 mg/mL in PBS. From this stock, a solution of MTT in the cell culture media was prepared to achieve a final concentration of 0.5 mg/mL. Subsequently, 100 μL of the final solution was added to each well, followed by incubation for 4 h at 37.5 °C. The MTT solution was thereafter removed, and the generated formazan crystals were dissolved by adding 100 μL of propanol/DMSO (50:50) per well. Absorbance was measured at 570 nm, and IC_50_ values for each compound were calculated by comparison against negative untreated controls.

#### 4.3.5. In Vitro Enzyme Inhibition Assay

The enzyme inhibitory activities of the compounds against EGFR and HER2 enzymes were assessed using the EGFR Kinase Assay Kit (Catalog # 40321) and HER2 Kinase Assay Kit (Catalog # 40721), following the manufacturer’s (BPS Bioscience, San Diego, CA, USA) protocol.

#### 4.3.6. Cell Cycle Assay

In a flat-bottom 6-well plate, 3 × 10^5^ A549 cells were seeded per well and incubated at 37 °C for 24 h to allow attachment. Afterward, triplicate wells were exposed to the IC_50_) concentration of the compounds under investigation (0.81 µM for **19a** and 3.95 µM for **22b**) in cell culture media. Following a 24 h incubation period, the supernatant was removed by suction, and adherent cells were washed with PBS and collected with trypsin. Additionally, negative control (untreated) samples were also prepared. The cells were resuspended in 2 mL of 80% *v*/*v* of cold methanol for 45 min to achieve fixation. After centrifugation, the methanol was discarded, and the resulting cell pellets were washed and stained at room temperature for 30 min in the dark using propidium iodide PI (10% *v*/*v* of a 1 mg/mL stock solution in PBS) and RNAse (15% *v*/*v* of a 1 mg/mL stock solution in PBS). The cells were analyzed using flow cytometry, reading histograms in the red channel adjusted to PI (Em. 617 nm, Ex. 535 nm).

#### 4.3.7. Apoptosis Analysis

Briefly, in flat-bottom 6-well plates, a total of 3 × 10^5^ A549 cells were seeded per well and maintained at 37 °C for 24 h in a CO_2_ humidified atmosphere. Next, the cells were subjected to treatment with the equipotent concentration (1xIC_50_) of the tested compounds in the cell culture media. Following a 24 h incubation period, the supernatant was removed by suction, and attached cells were washed with PBS and collected using trypsin. Following the quenching of trypsin with culture media, the cells were resuspended as a single cell suspension in Annexin V binding buffer with 1% (*v*/*v*) propidium iodide and 1% (*v*/*v*) Annexin V-FITC. Negative (untreated) control samples were also included in the experiment. The samples were analyzed using flow cytometry, reading PI at Em. 617 nm, Ex. 535 nm, and Annexin-V-FITC at Em. 520 nm, Ex. 488 nm, while collecting 20,000 events/sample.

#### 4.3.8. Statistical Analysis

The statistical significance of biological experiments was calculated by GraphPad Prism 10.2.3, using unpaired sample *t*-tests to compare the populations treated with the tested compound against the reference drug (lapatinib) unless otherwise stated. Significance levels are represented as * for *p* < 0.05, ** for *p* < 0.01, *** for *p* < 0.001, and **** for *p* < 0.0001.

## 5. Conclusions

In this study, new purine-containing hydrazone derivatives **6**–**24** (**a**,**b**) were designed, synthesized, and screened for their cytotoxicity potential against a panel of human cancer cell lines, including A549, SKOV-3, A2780, and SKBR-3, as well as normal cells (MRC-5), using erlotinib and lapatinib as standard drugs. The cytotoxicity assay results indicate that the incorporation of a bulky and flexible hydrophobic head group, such as 3-biphenyl, is more advantageous for cytotoxic activity compared to a planar structure group like naphthyl. Furthermore, the insertion of an electron-withdrawing group at the meta position of the phenyl ring (at R_1_), such as 3-trifluoromethyl or 3-sulfonamide-4-fluoro, is more favorable than the introduction of an electron-donating group. The protection of the *N*9 position of the purine ring with a cyclopentyl moiety generally yields compounds exhibiting enhanced growth inhibition activity, except **19b**. Among the evaluated compounds, **19a**, **16b**, and **22b** displayed noteworthy efficacy against A549, SKOV-3, and SKBR-3 cells, surpassing the clinically approved lapatinib. Furthermore, these derivatives showed low cytotoxicity against the normal MRC-5 cell line, validating the safety margin of these derivatives. Additionally, those derivatives exhibited enhanced EGFR kinase inhibition activity relative to lapatinib. Compound **22b** displayed approximately an equivalent suppression potency to lapatinib against HER2 kinase. Compounds **19a** and **22b** arrest the cell cycle at the G1 phase and induce apoptosis in the A549 cell line, which is characterized by EGFR overexpression. The docking simulation study indicated that compounds **19a** and **22b** displayed good binding affinity to the ATP pocket of both EGFR and HER2, with binding scores comparable to that of lapatinib. Compound **19a** interacts with the EGFR active site by establishing three hydrogen bonds with Lys745, Leu718, and Cys797. Moreover, it binds to HER2 by creating a hydrogen bond with Cys805, along with hydrophobic interactions with the amino acid residues Leu796, Val734, Leu852, Leu726, and Lys753. The expected binding interactions between the EGFR/HER2 kinase domain and compound **22b** indicate that **22b** occupies the EGFR active site by forming hydrogen bonds with Thr854, Asp800, Cys797, Thr790, and Asp855. On HER2, **22b** develops hydrogen bonds with the amino acid residues Lys753, Thr862, Glu770, and Gly865.

An overview of the results indicates that **19a**, **16b**, and **22b** are novel effective anticancer agents demonstrating favorable safety profiles towards normal cells along with significant inhibitory effects toward EGFR and HER2 kinases. All of these advantages together suggest that these derivatives may serve as promising lead compounds for the future development of more effective anticancer candidates targeting EGFR and HER2.

## Figures and Tables

**Table 1:** The binding scores of lapatinib, **6a**, **19a**, and **23a** within the active sites of EGFR and HER2 kinases.

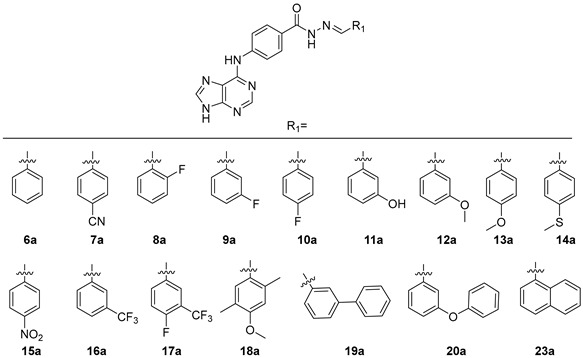					
Compound	Binding score (kcal/mol)		Compound	Binding score (kcal/mol)	
	EGFR	HER2		EGFR	HER2
**6a**	−9.2	−8.9	**23a**	−10.2	−10.9
**19a**	−11.0	−10.3	Lapatinib	−11.1	−10.3

**Table 2:** The binding scores of lapatinib, **6b**, **16b**, **22b**, **17b**, **18b**, **19b**, and **23b** within the active sites of EGFR and HER2 kinases.

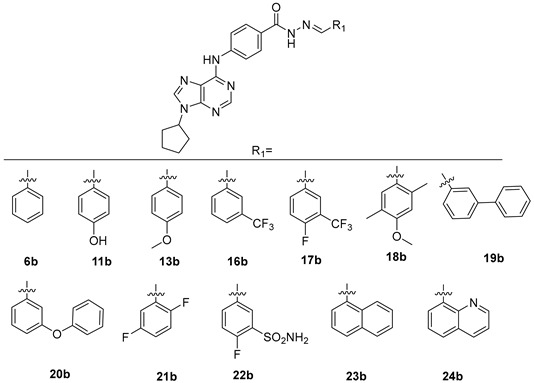					
Compound	Binding score (kcal/mol)		Compound	Binding score (kcal/mol)	
	EGFR	HER2		EGFR	HER2
**6b**	−9.8	−9.6	**19b**	−11.7	−10.6
**16b**	−10.6	−10	**22b**	−11.0	−10.8
**17b**	−11.0	−10	**23b**	−10.3	−10.9
**18b**	−9.9	−9.9	Lapatinib	−11.1	−10.3

**Table 3:** In vitro cytotoxicity assessment of **19a**, **16b**, **22b**, and **19b** against various human cancer/normal cell lines.

Compound No.	Cytotoxicity (IC_50_) in µM			
	A549 (SI) **	SKOV-3 (SI) **	SKBR-3 (SI) **	MRC-5
**19a**	0.810 ± 0.139 **** (64.6)	2.192 ± 0.164 ** (23.88)	1.414 ± 0.362 *** (37.03)	52.360 ± 0.283
**16b**	2.604 ± 0.351 *** (14.07)	1.975 ± 0.07 ** (18.55)	4.308 ± 0.948 ** (8.51)	36.650 ± 0.200
**22b**	3.950 ± 0.615 **** (15.34)	1.541 ± 0.470 ** (39.32)	3.801 ± 1.401 ** (15.94)	60.590 ± 0.329
**19b**	1.844 ± 0.082	15.645 ± 1.475	3.197 ± 0.899	ND
Lapatinib	11.570 ± 0.249 (3.8)	16.950 ± 2.570 (2.6)	8.540 ± 0.640 (5.14)	43.940 ± 0.420
Erlotinib	2.420 ± 0.784 (8.0)	5.180 ± 1.200 (3.7)	5.120 ± 0.879 (3.8)	19.360 ± 0.138

The values represent the mean ± SD of three independent experiments; ** The selectivity index (SI) is expressed as the ratio IC_50_ against MRC-5/IC_50_ against the cancer cell line; ND = not detected. (Significance determined by unpaired sample *t*-test where, ** indicates *p* < 0.01, *** indicates *p* < 0.001, and **** indicates *p* < 0.0001 levels of significance).

**Table 4:** In vitro kinase inhibition activity assessment of **19a**, **16b**, and **22b**, against EGFR and HER2.

Compound No.	Protein Kinase Inhibition IC_50_ (µM) ± SEM	
	EGFR	HER2
**19a**	0.081 ± 0.003 **	0.059 ± 0.002
**16b**	0.062 ± 0.002 ****	0.045 ± 0.010
**22b**	0.074 ± 0.002 ****	0.033 ± 0.015
Lapatinib	0.130 ± 0.002	0.037 ± 0.016
Erlotinib	0.038 ± 0.002	0.052 ± 0.004

IC_50_ values ± SD of three independent experiments. (Significance determined by unpaired sample *t*-test where, ** indicates *p* < 0.01, and **** indicates *p* < 0.0001 levels of significance).

**Table 5:** The effect of compounds **19a** and **22b** on cell cycle progression of A549 cells

Compound/Cell Line	DNA Content (%)			Cell Cycle Distribution Index (CDI)/Comment
	%G0–G1	%S	%G2/M	
Cont. A549	54.23 ± 0.92	31.40 ± 1.12	14.36 ± 0.91	0.84
**19a**/A549	86.88 ± 0.95 ****	9.21 ± 1.43 ****	3.90 ± 1.22 ***	0.15 Cell cycle arrest@G1
**22b**/A549	77.04 ± 1.85 ***	18.60 ± 2.00 **	4.35 ± 0.29 ***	0.29 Cell cycle arrest@G1

Data is represented as the mean ± SD of three independent experiments. (Significance determined by Unpaired sample *t*-test where ** indicates *p* < 0.01, *** indicates *p* < 0.001, and **** indicates *p* < 0.0001 levels of significance).

**Table 6:** The effect of compounds **19a** and **22b** on the distribution of the apoptotic cells in A549 cells.

Compound/Cell Line	Apoptosis			Necrosis
	Total	Early	Late	
Cont. A549	2.38 ± 0.31	0.48 ± 0.10	0.17 ± 0.02	1.72 ± 0.23
**19a**/A549	37.70 ± 1.40 ****	9.72 ± 1.06 ***	23.13 ± 0.73 ****	4.84 ± 0.34
**22b**/A549	27.48 ± 0.57 ****	5.92 ± 0.74 ***	18.21 ± 0.12 ****	3.35 ± 0.49

Data is represented as the mean ± SD of three independent experiments. (Significance determined by unpaired sample *t*-test where and *** indicates *p* < 0.001, **** indicates *p* < 0.0001 levels of significance).

## Data Availability

Data are given in the supporting file and are available upon request.

## Supplementary Materials

The following supporting information can be downloaded at https://www.mdpi.com/article/10.3390/ph18071051/s1: ^1^H-NMR, ^13^C-NMR, and mass spectra.
