# Supplementary material for: Purine–Hydrazone Scaffolds as Potential Dual EGFR/HER2 Inhibitors
Source: Pharmaceuticals (Basel). 2025 Jul 17;18(7):1051. doi: 10.3390/ph18071051 (PMC12299904; doi:10.3390/ph18071051)
Supplement: Supplementary file 1 [file pharmaceuticals-18-01051-s001.zip › pharmaceuticals-3731498-supplementary.pdf]

Purine-hydrazone scaffolds as potent dual EGFR/HER2 inhibitors

[1]School of Pharmacy, University of Birmingham, Edgbaston, Birmingham, B15 2TT, UK; [2]Department of Pharmaceutical Chemistry, College of Pharmacy, King Saud University, Riyadh 11451, Saudi Arabia.

Content	Page no.	Content	Page no.	Content	Page no.
Structures of the compounds **6a-20a** and **23a**	4	^{1}H-NMR spectra of compound **8a**	26	Mass spectra of compound **13a**	48
Structures of the compounds **6b, 11b,13b,16b-24b**	5	^{13}C-NMR spectra of compound **8a**	27	HRMS spectra of compound **13a**	49
^{1}H-NMR spectra of compound **1b**	6	Mass spectra of compound **8a**	28	^{1}H-NMR spectra of compound **14a**	50
^{13}C-NMR spectra of compound **1b**	7	HRMS spectra of compound **8a**	29	^{13}C-NMR spectra of compound **14a**	51
^{1}H-NMR spectra of compound **3a**	8	^{1}H-NMR spectra of compound **9a**	30	Mass spectra of compound **14a**	52
^{13}C-NMR spectra of compound **3a**	9	^{13}C-NMR spectra of compound **9a**	31	HRMS spectra of compound **14a**	53
Mass spectra of compound **3a**	10	Mass spectra of compound **9a**	32	^{1}H-NMR spectra of compound **15a**	54
^{1}H-NMR spectra of compound **3b**	11	HRMS spectra of compound **9a**	33	^{13}C-NMR spectra of compound **15a**	55
^{13}C-NMR spectra of compound **3b**	12	^{1}H-NMR spectra of compound **10a**	34	Mass spectra of compound **15a**	56
^{1}H-NMR spectra of compound **4a**	13	^{13}C-NMR spectra of compound **10a**	35	HRMS spectra of compound **15a**	57
^{13}C-NMR spectra of compound **4a**	14	Mass spectra of compound **10a**	36	^{1}H-NMR spectra of compound **16a**	58
Mass spectra of compound **4a**	15	HRMS spectra of compound **10a**	37	^{13}C-NMR spectra of compound **16a**	59
^{1}H-NMR spectra of compound **4b**	16	^{1}H-NMR spectra of compound **11a**	38	Mass spectra of compound **16a**	60
^{13}C-NMR spectra of compound **4b**	17	^{13}C-NMR spectra of compound **11a**	39	HRMS spectra of compound **16a**	61
^{1}H-NMR spectra of compound **6a**	18	Mass spectra of compound **11a**	40	^{1}H-NMR spectra of compound **17a**	62
^{13}C-NMR spectra of compound **6a**	19	HRMS spectra of compound **11a**	41	^{13}C-NMR spectra of compound **17a**	63
Mass spectra of compound **6a**	20	^{1}H-NMR spectra of compound **12a**	42	Mass spectra of compound **17a**	64
HRMS spectra of compound **6a**	21	^{13}C-NMR spectra of compound **12a**	43	HRMS spectra of compound **17a**	65
^{1}H-NMR spectra of compound **7a**	22	Mass spectra of compound **12a**	44	^{1}H-NMR spectra of compound **18a**	66
^{13}C-NMR spectra of compound **7a**	23	HRMS spectra of compound **12a**	45	^{13}C-NMR spectra of compound **18a**	67
Mass spectra of compound **7a**	24	^{1}H-NMR spectra of compound **13a**	46	Mass spectra of compound **18a**	68
HRMS spectra of compound **7a**	25	^{13}C-NMR spectra of compound **13a**	47	HRMS spectra of compound **18a**	69

Content	Page no.	Content	Page no.	114Content	Page no.
^{1}H-NMR spectra of compound **19a**	70	Mass spectra of compound **13b**	92	^{1}H-NMR spectra of compound **19b**	114
^{13}C-NMR spectra of compound **19a**	71	HRMS spectra of compound **13b**	93	^{13}C-NMR spectra of compound **19b**	115
Mass spectra of compound **19a**	72	^{1}H-NMR spectra of compound **16b**	94	Mass spectra of compound **19b**	116
HRMS spectra of compound **19a**	73	^{13}C-NMR spectra of compound **16b**	95	HRMS spectra of compound **19b**	117
^{1}H-NMR spectra of compound **20a**	74	Mass spectra of compound **16b**	96	^{1}H-NMR spectra of compound **20b**	118
^{13}C-NMR spectra of compound **20a**	75	HRMS spectra of compound **16b**	97	^{13}C-NMR spectra of compound **20b**	119
Mass spectra of compound **20a**	76	^{1}H-NMR spectra of compound **21b**	98	Mass spectra of compound **20b**	120
HRMS spectra of compound **20a**	77	^{13}C-NMR spectra of compound **21b**	99	HRMS spectra of compound **20b**	121
^{1}H-NMR spectra of compound **23a**	78	Mass spectra of compound **21b**	100	^{1}H-NMR spectra of compound **23b**	122
^{13}C-NMR spectra of compound **23a**	79	HRMS spectra of compound **21b**	101	^{13}C-NMR spectra of compound **23b**	123
Mass spectra of compound **23a**	80	^{1}H-NMR spectra of compound **22b**	102	Mass spectra of compound **23b**	124
HRMS spectra of compound **23a**	81	^{13}C-NMR spectra of compound **22b**	103	HRMS spectra of compound **23b**	125
^{1}H-NMR spectra of compound **6b**	82	Mass spectra of compound **22b**	104	^{1}H-NMR spectra of compound **24b**	126
^{13}C-NMR spectra of compound **6b**	83	HRMS spectra of compound **22b**	105	^{13}C-NMR spectra of compound **24b**	127
Mass spectra of compound **6b**	84	^{1}H-NMR spectra of compound **17b**	106	Mass spectra of compound **24b**	128
HRMS spectra of compound **6b**	85	^{13}C-NMR spectra of compound **17b**	107	HRMS spectra of compound **24b**	129
^{1}H-NMR spectra of compound **11b**	86	Mass spectra of compound **17b**	108		
^{13}C-NMR spectra of compound **11b**	87	HRMS spectra of compound **17b**	109		
Mass spectra of compound **11b**	88	^{1}H-NMR spectra of compound **18b**	110		
HRMS spectra of compound **11b**	89	^{13}C-NMR spectra of compound **18b**	111		
^{1}H-NMR spectra of compound **13b**	90	Mass spectra of compound **18b**	112		
^{13}C-NMR spectra of compound **13b**	91	HRMS spectra of compound **18b**	113		

Structures of the compounds **6a-20a** and **23a**

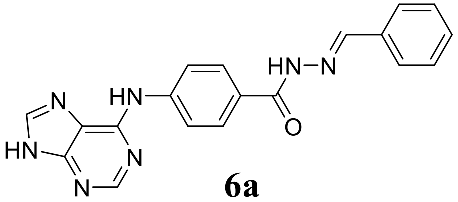
6a

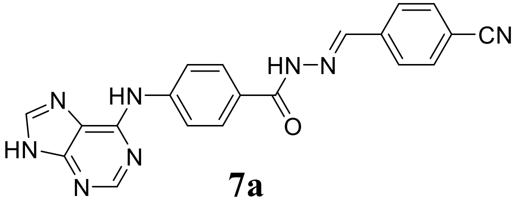
7a

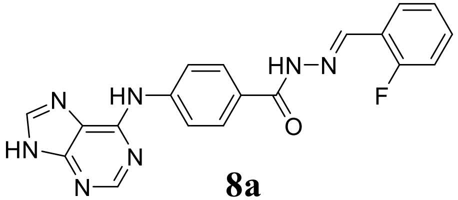
8a

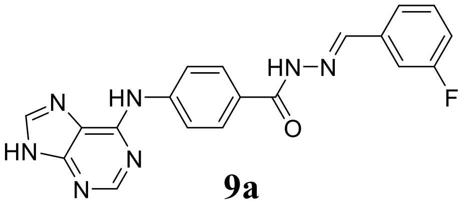
9a

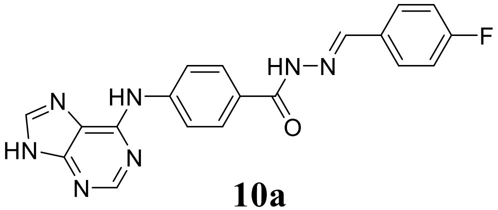
10a

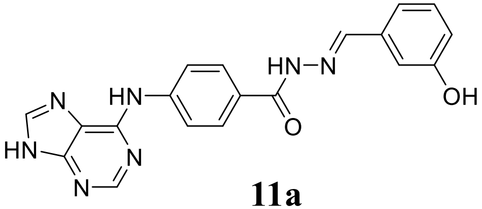
11a

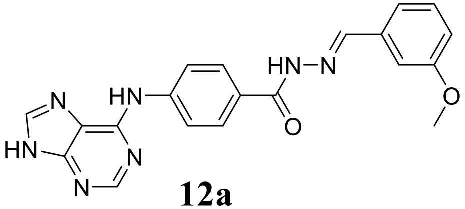
12a

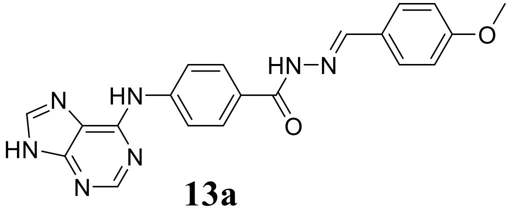
13a

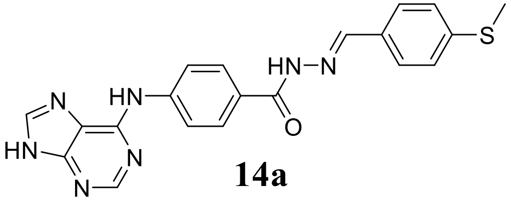
14a

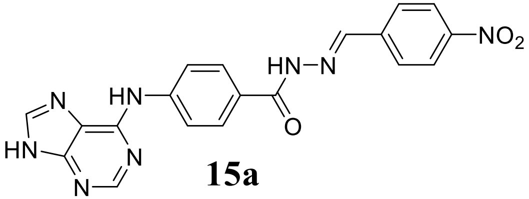
15a

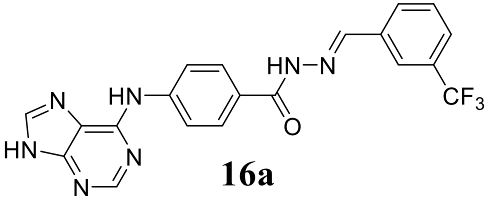
16a

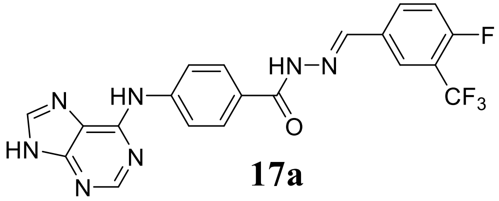
17a

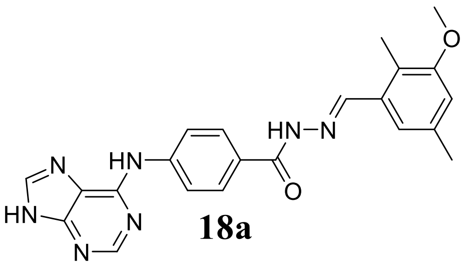
18a

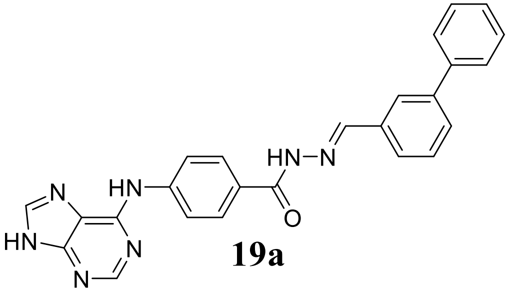
19a

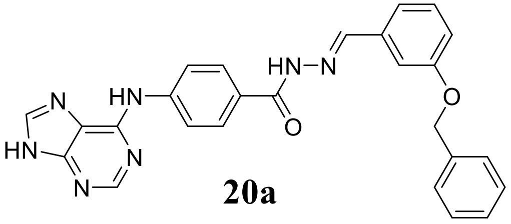
20a

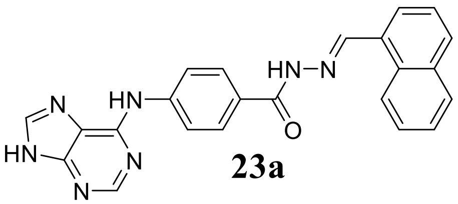
23a

Structures of the compounds **6b,11b,13b** and **16b-24b**

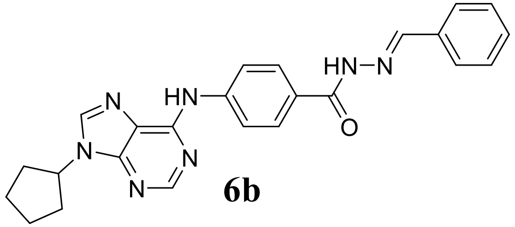

6b

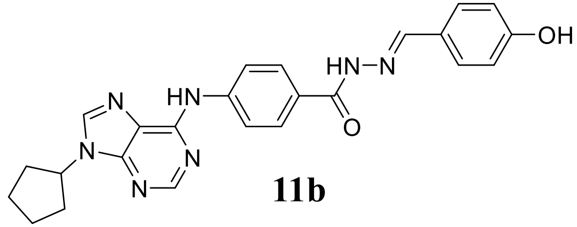

11b

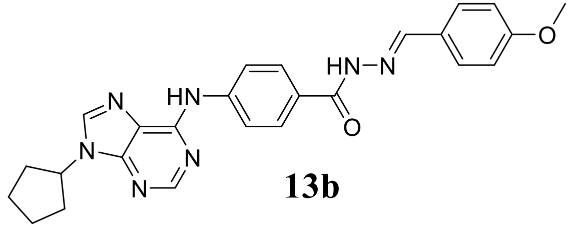

13b

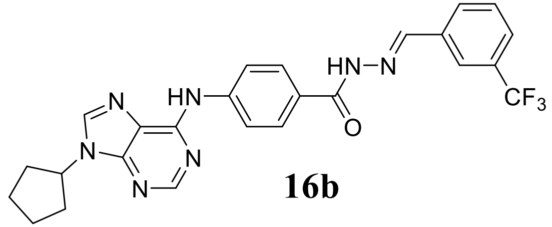

16b

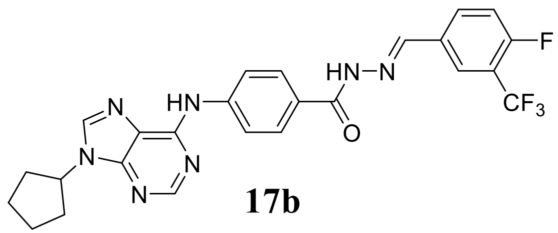

17b

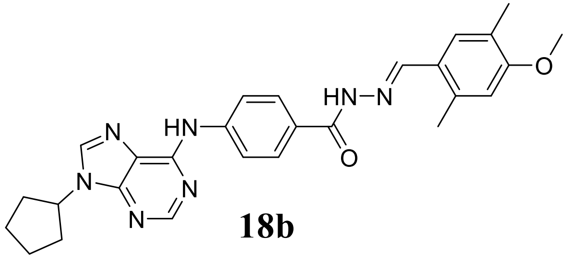

18b

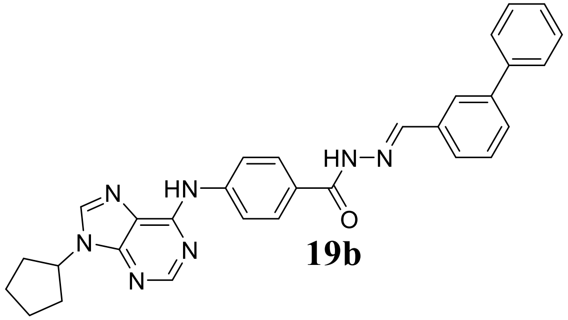

19b

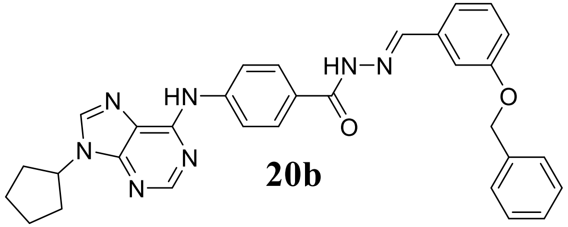

20b

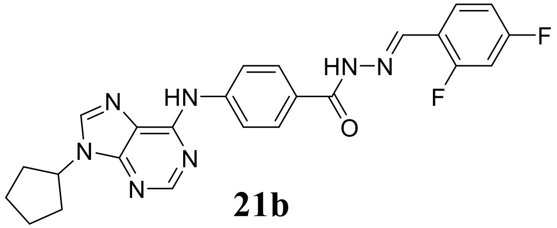

21b

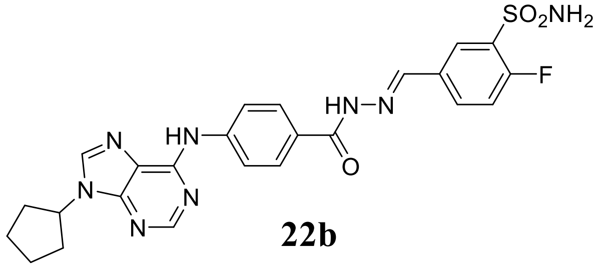

22b

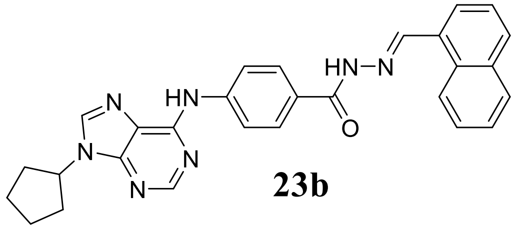

23b

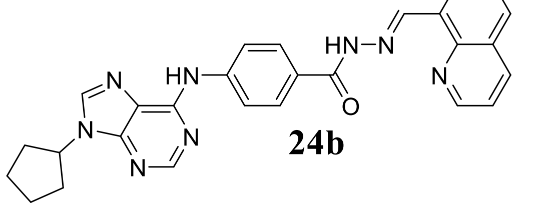

24b

^{1}H-NMR spectra of compound **1b**

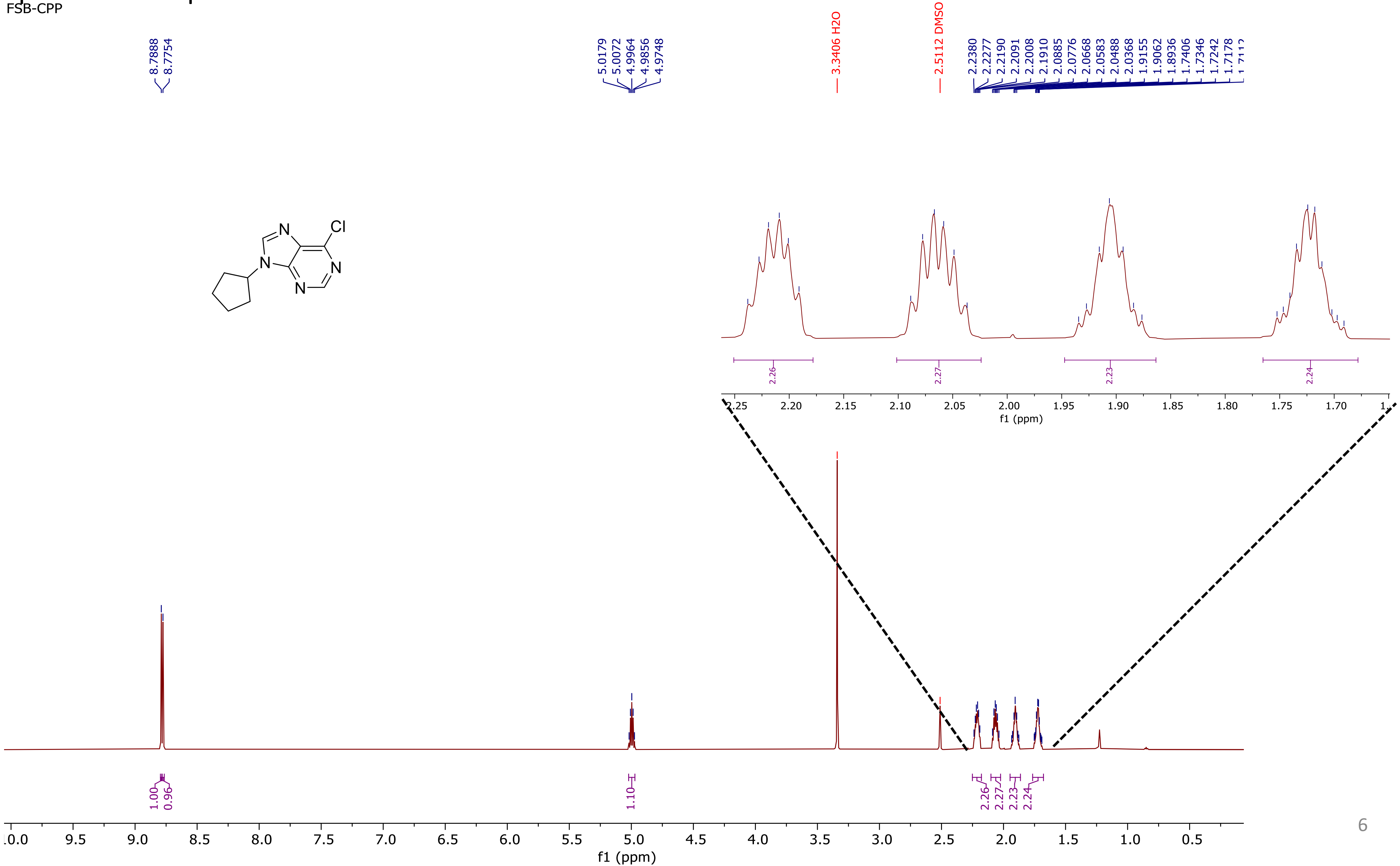

^{13}C-NMR spectra of compound **1b**

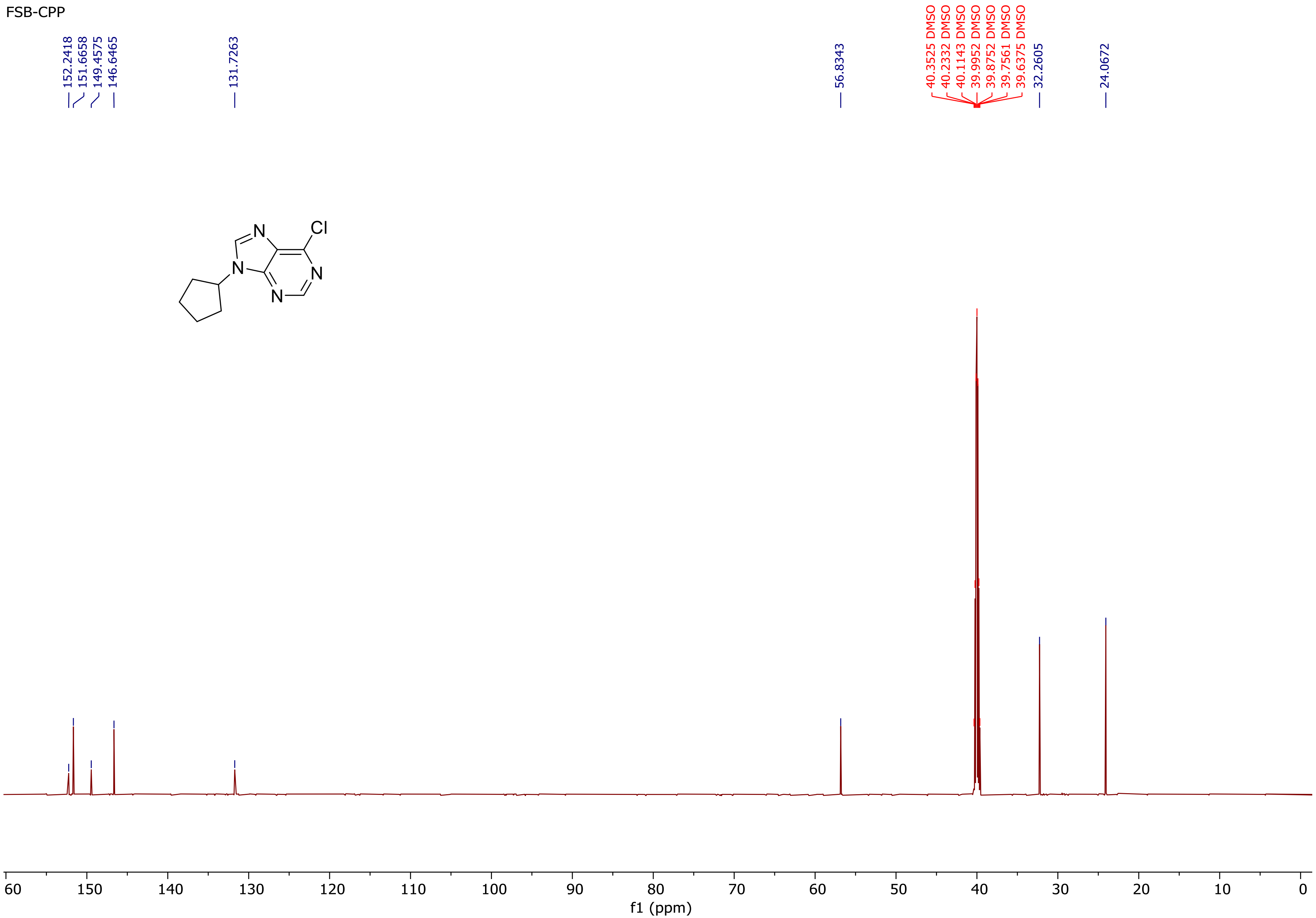

^{1}H-NMR spectra of compound **3a**

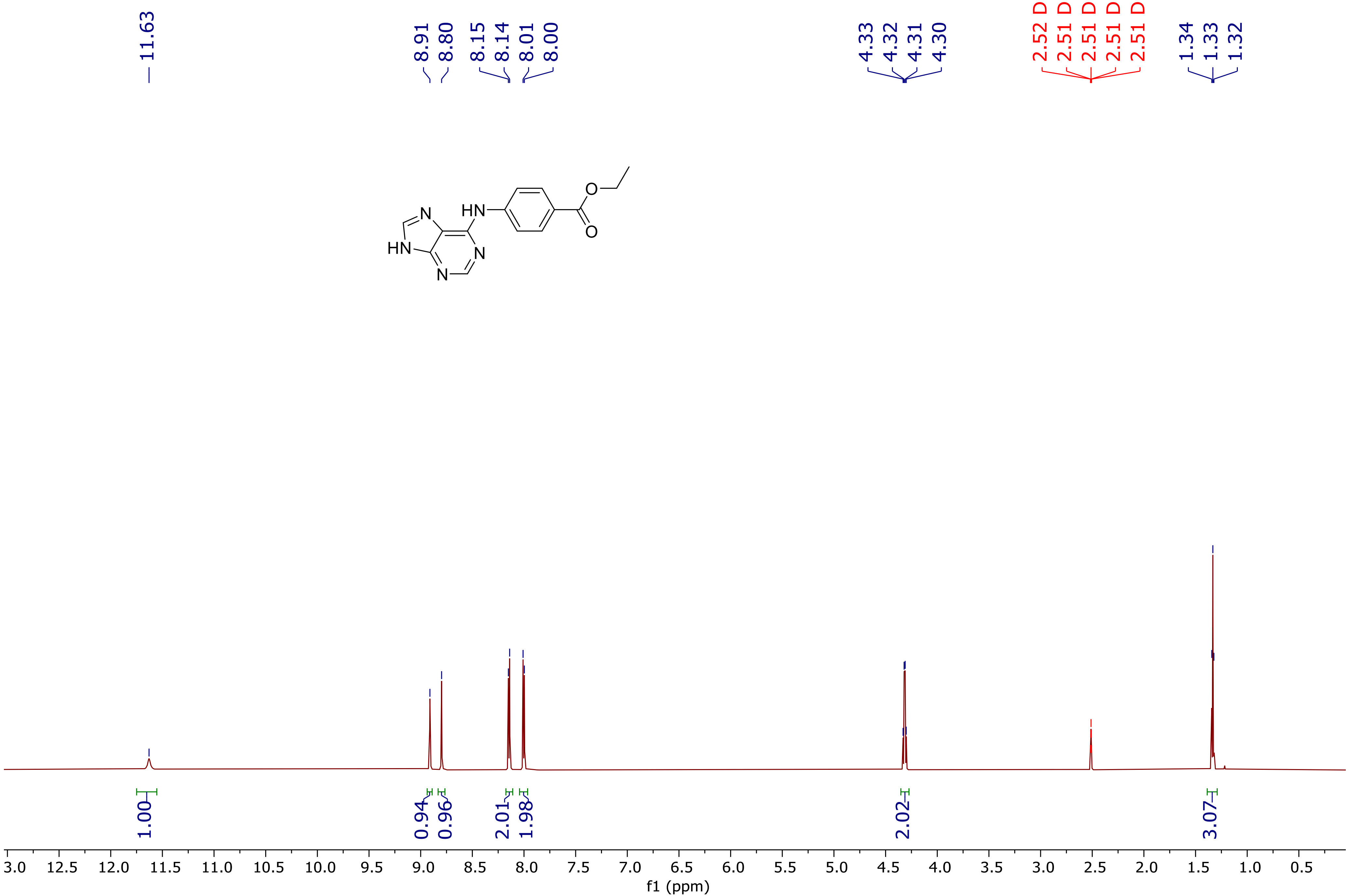

3

^{13}C-NMR spectra of compound **3a**

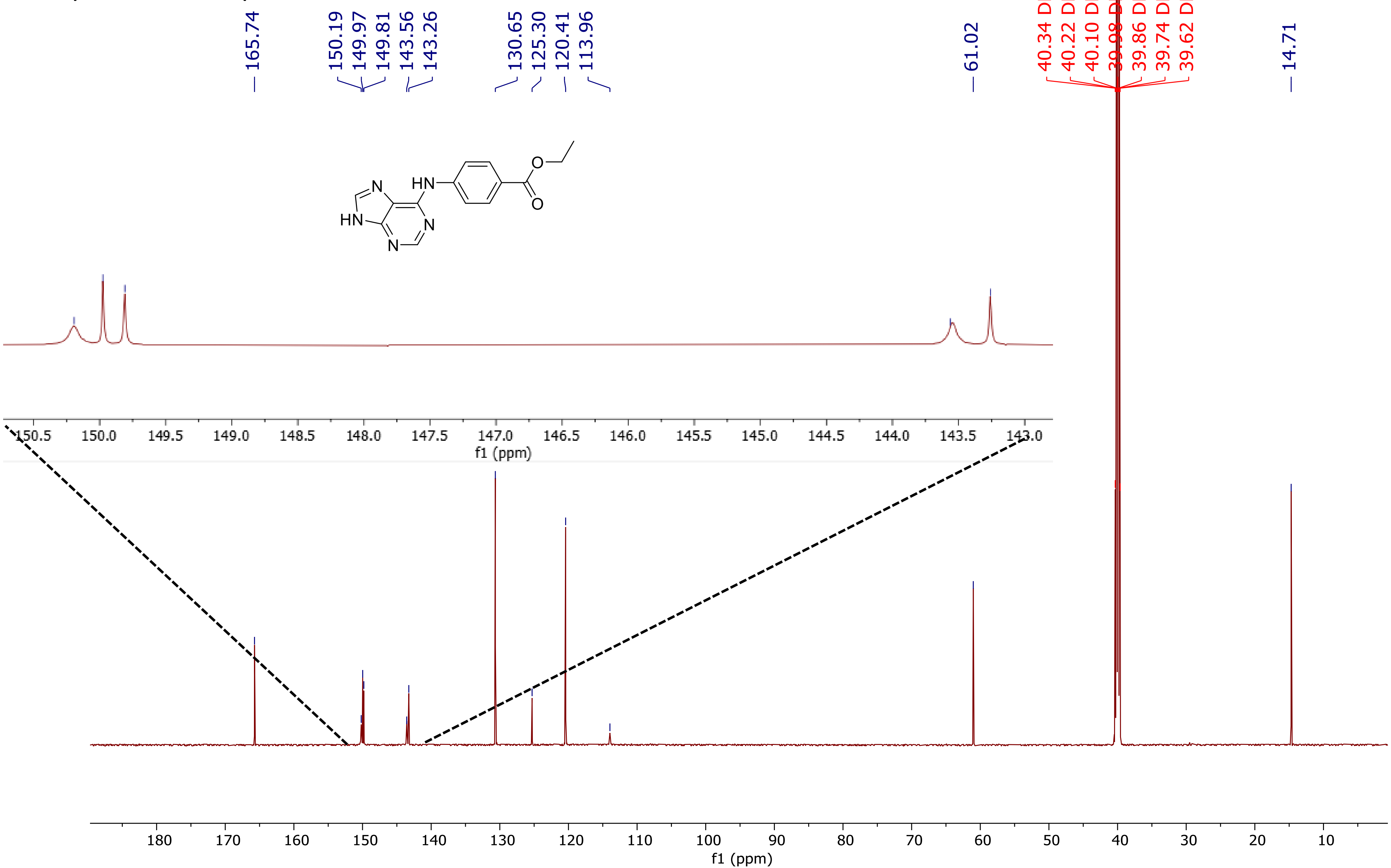

Mass spectra of compound **3a**

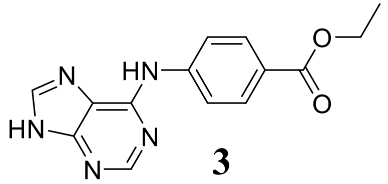

3

Chemical Formula: $C_{14}H_{13}N_5O_2$
Molecular Weight: 283

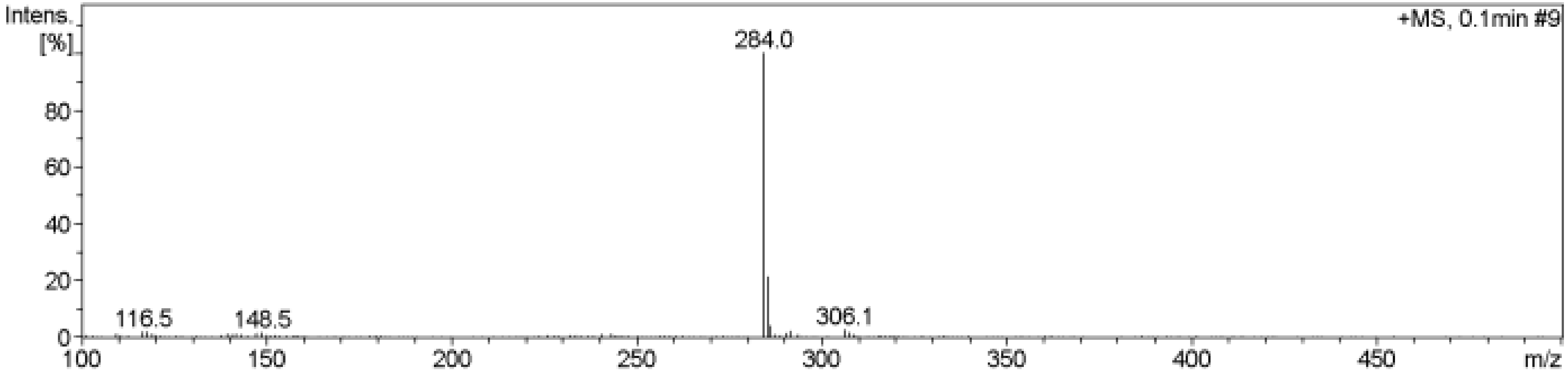

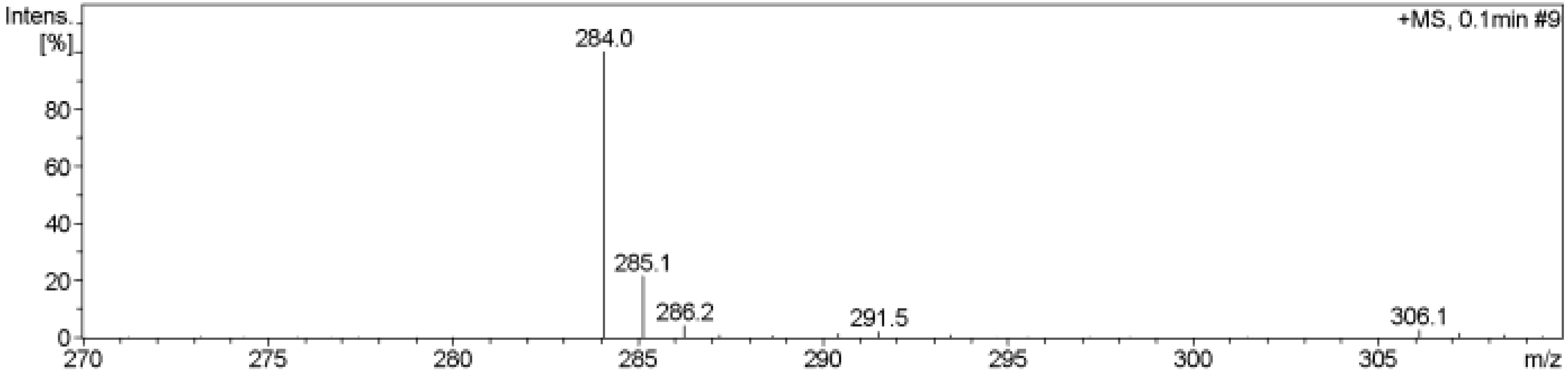

^{1}H-NMR spectra of compound **3b**

FSB-CPBenz

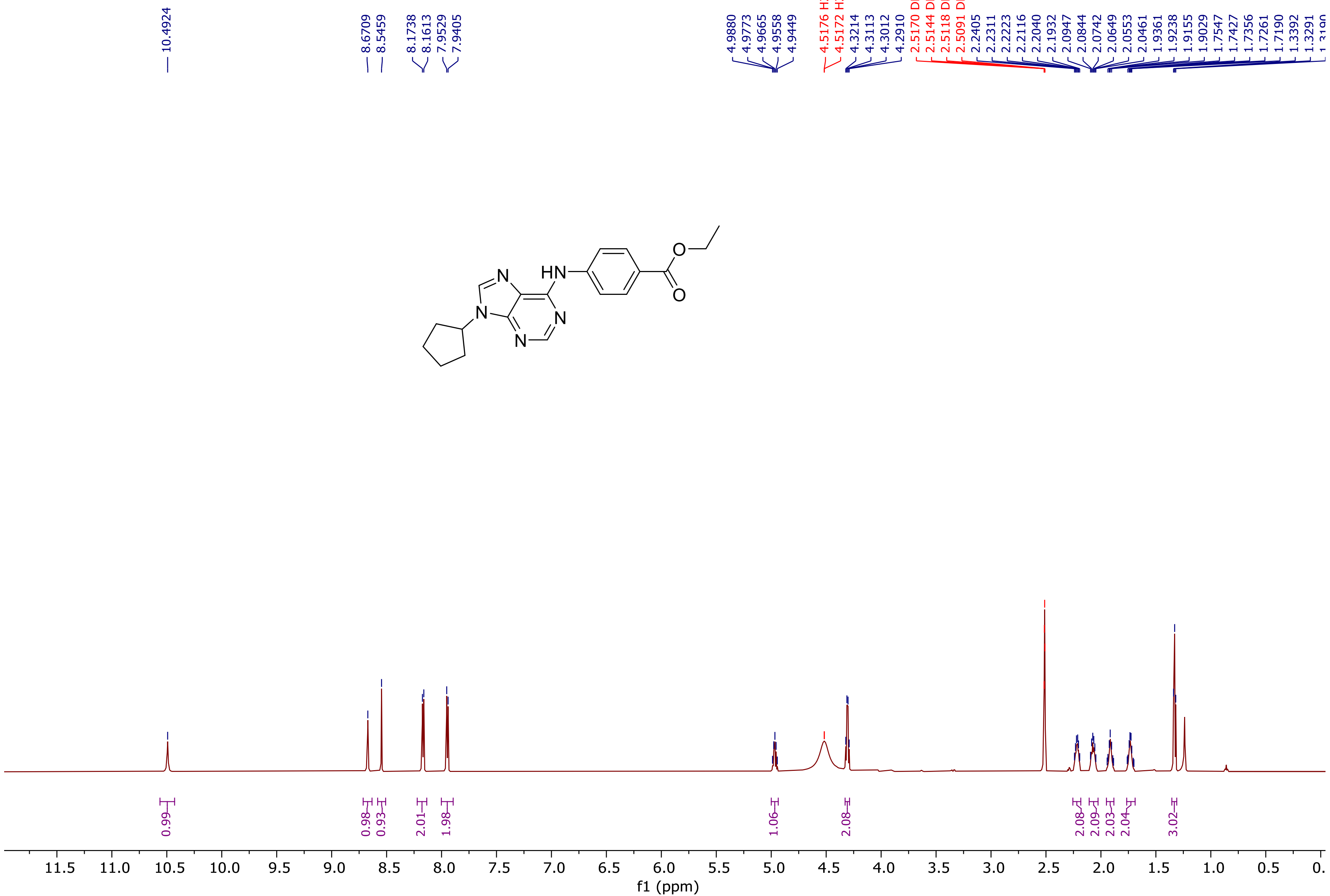

^{13}C-NMR spectra of compound **3b**

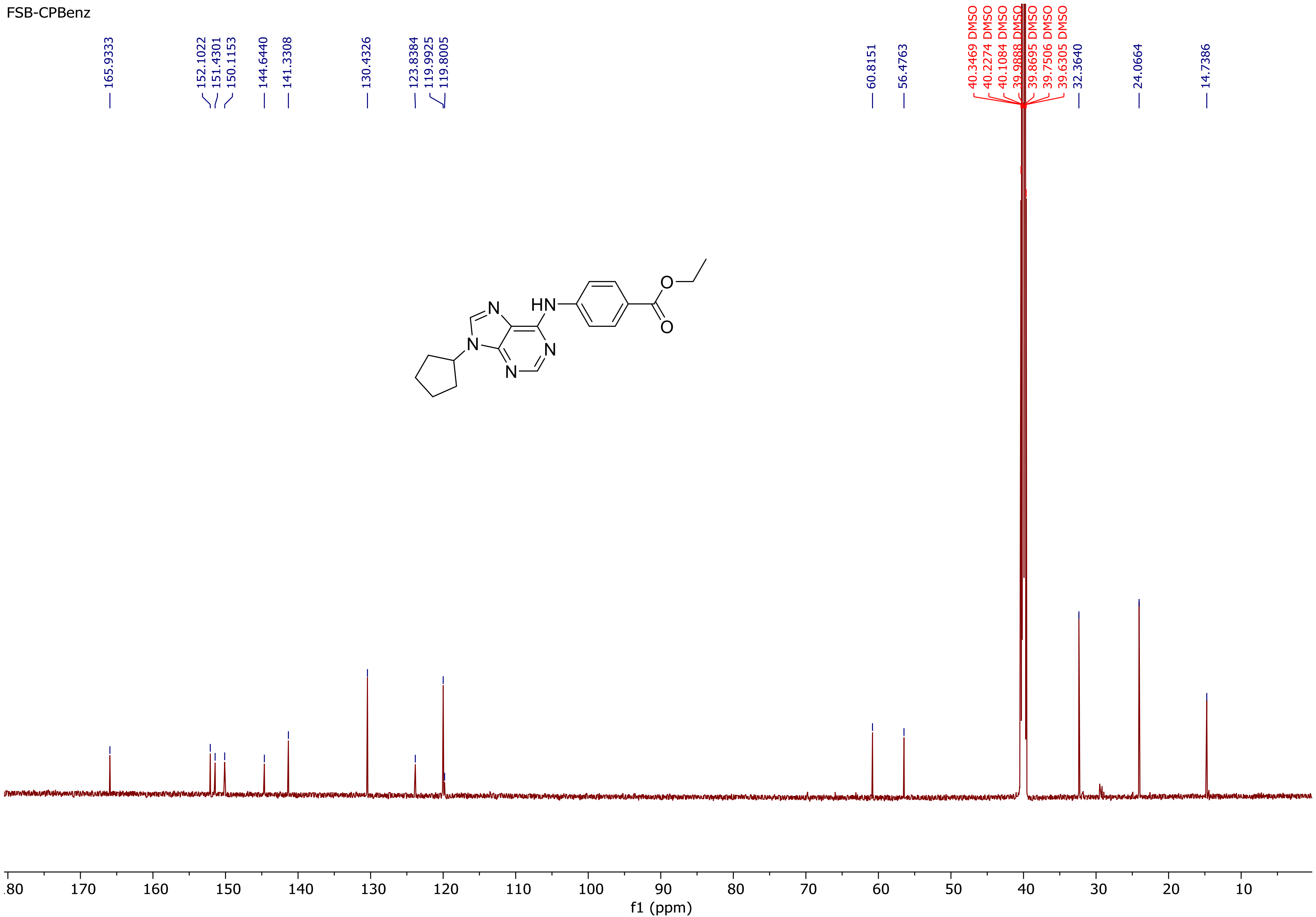

^{1}H-NMR spectra of compound **4a**

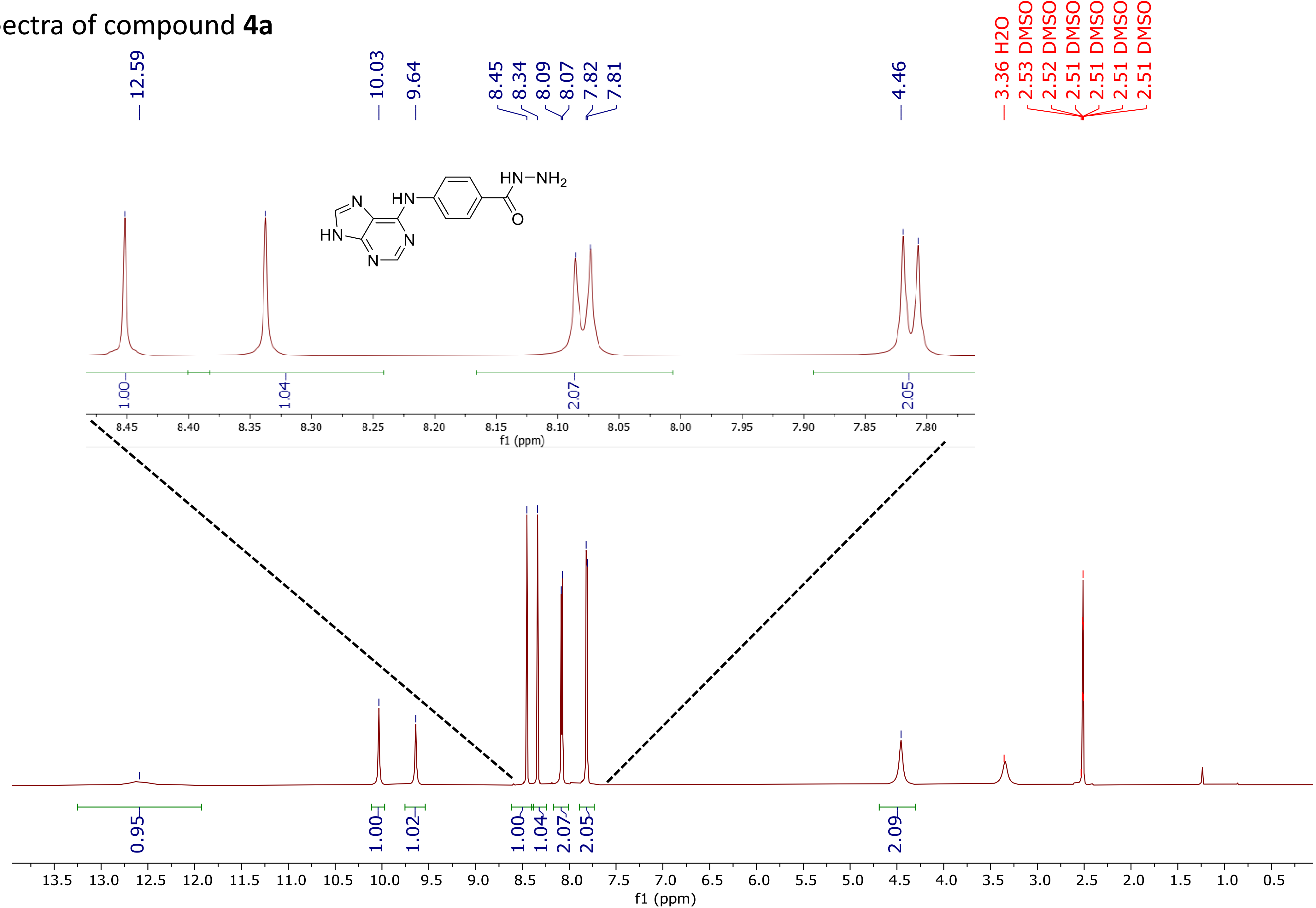

^{13}C-NMR spectra of compound **4a**

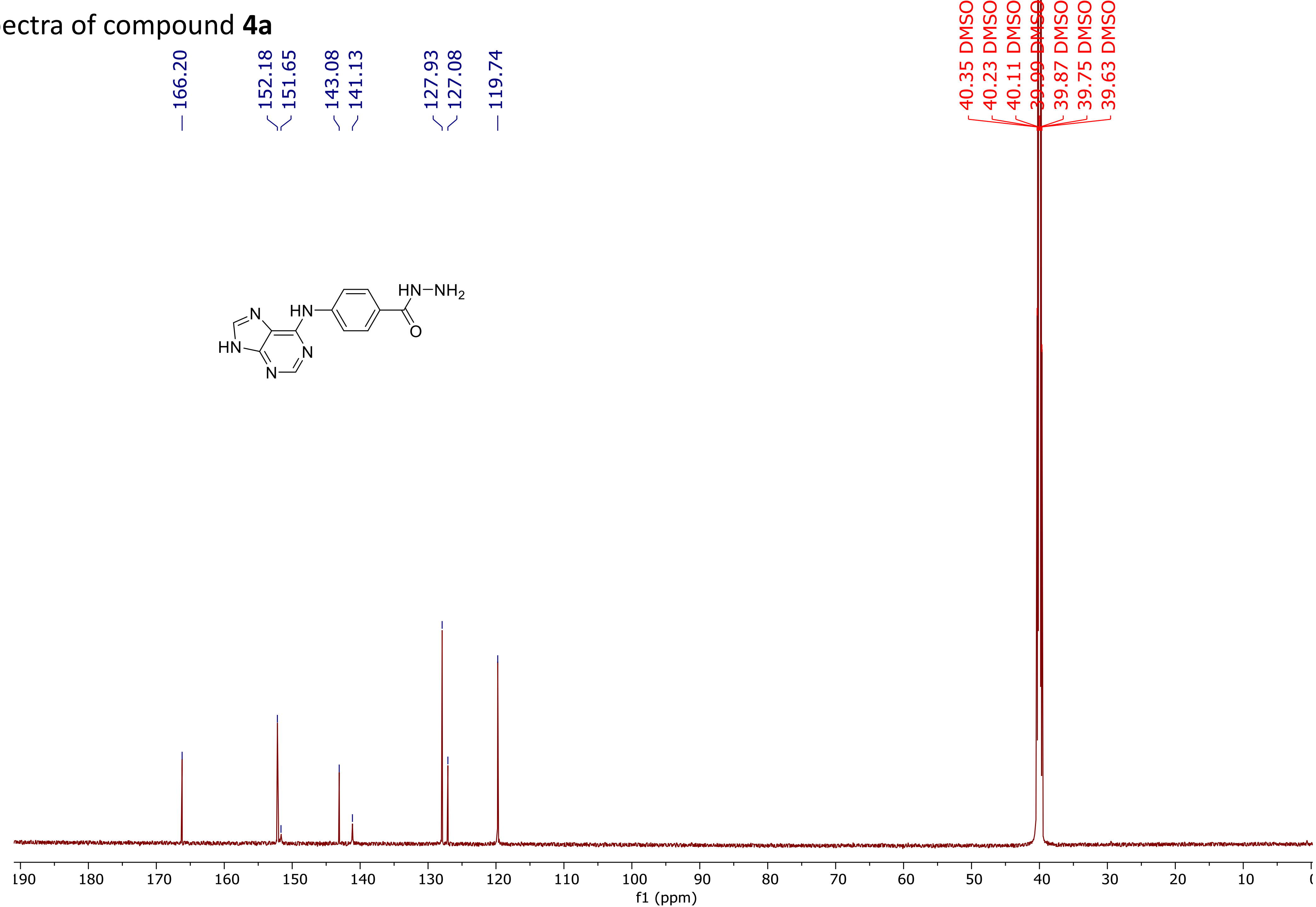

Mass spectra of compound **4a**

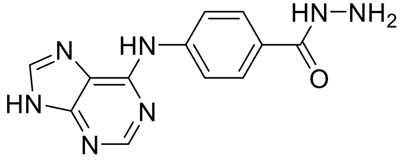

Chemical Formula: $C_{12}H_{11}N_7O$
Molecular Weight: 269

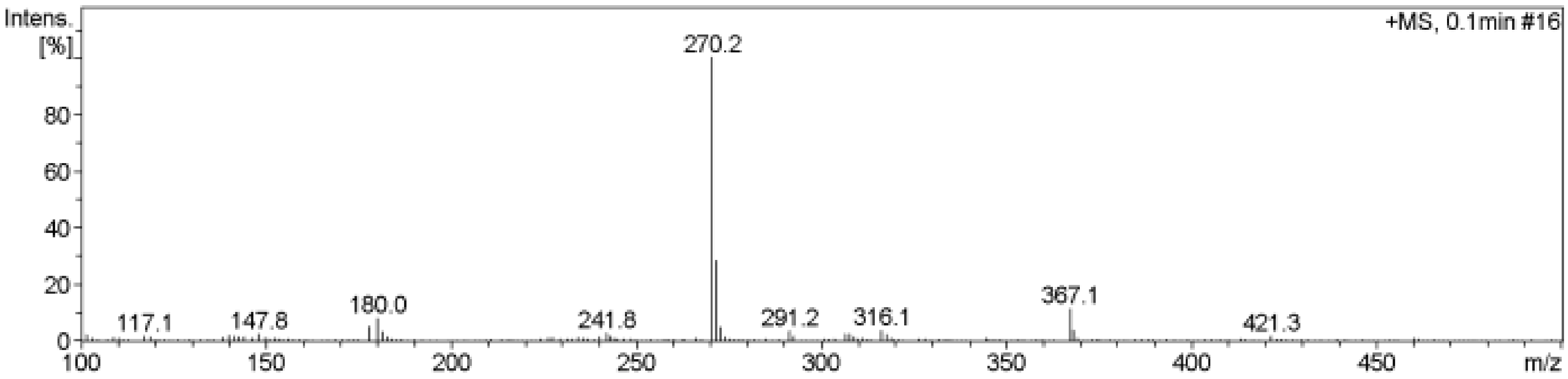

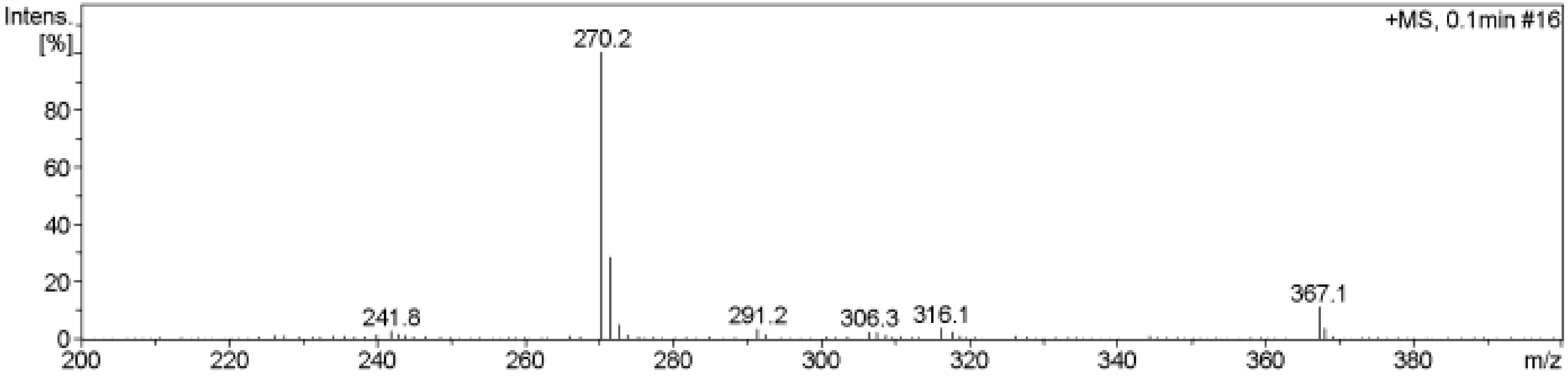

^{1}H-NMR spectra of compound **4b**

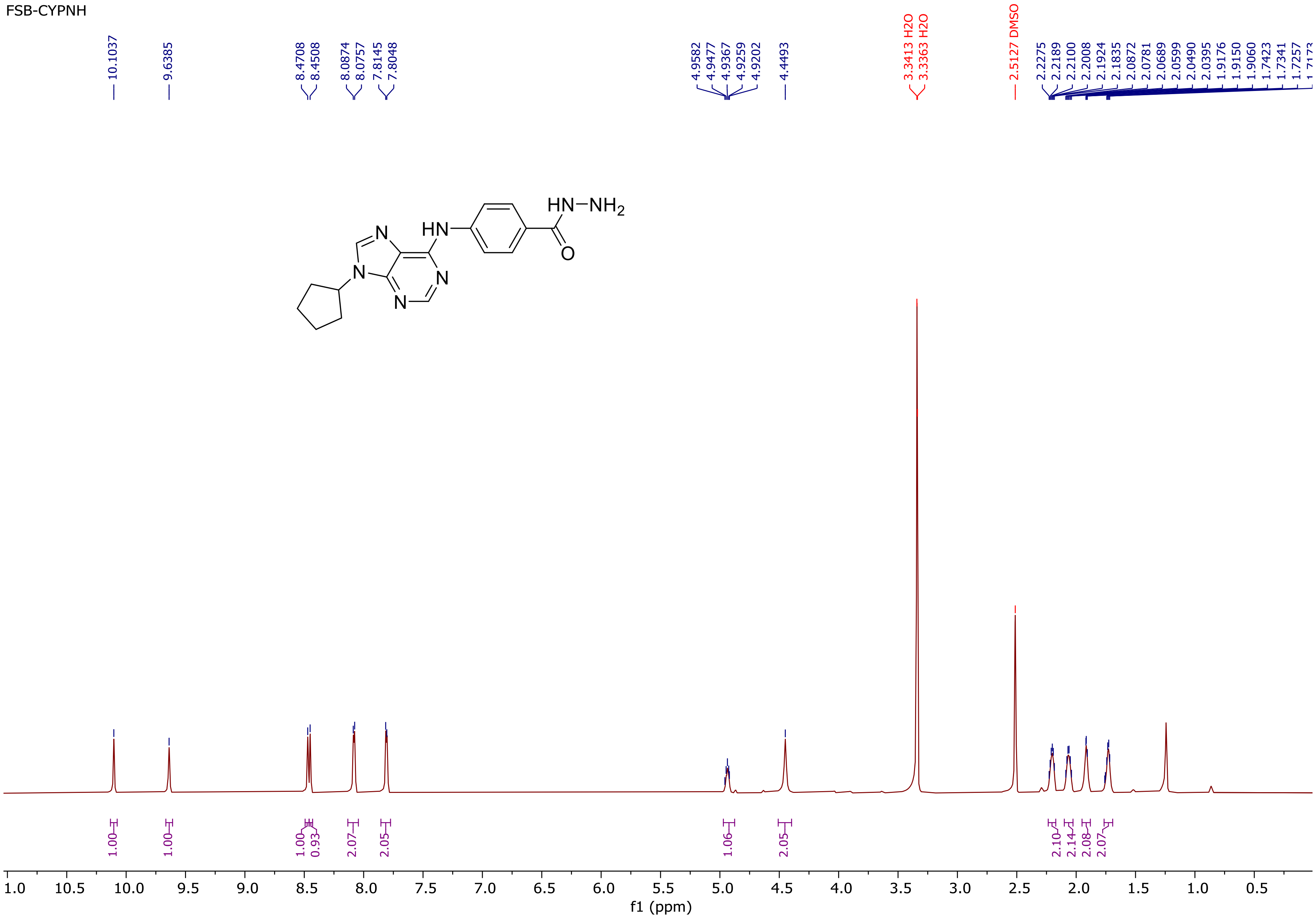

^{13}C-NMR spectra of compound **4b**

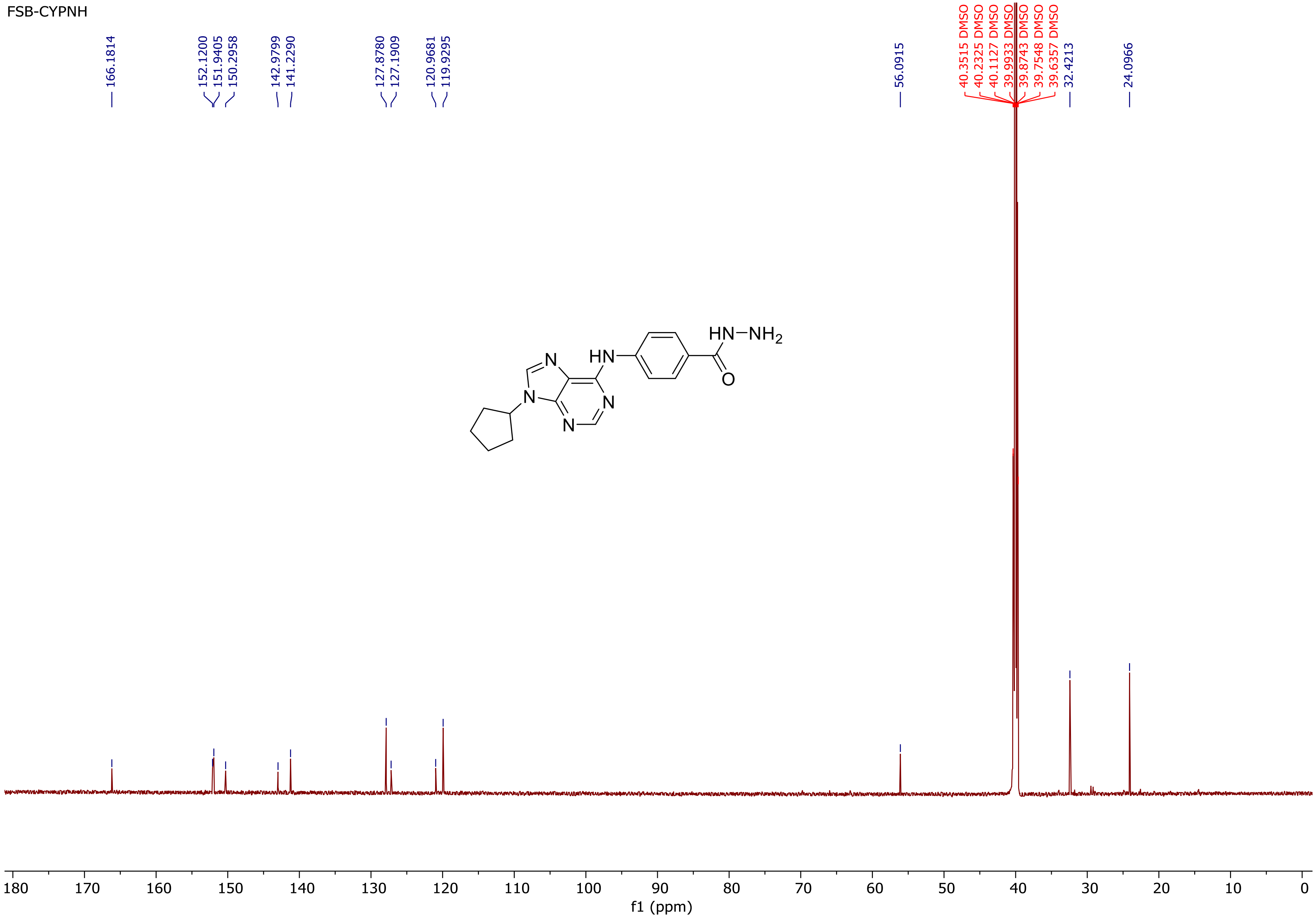

^{1}H-NMR spectra of compound **6a**

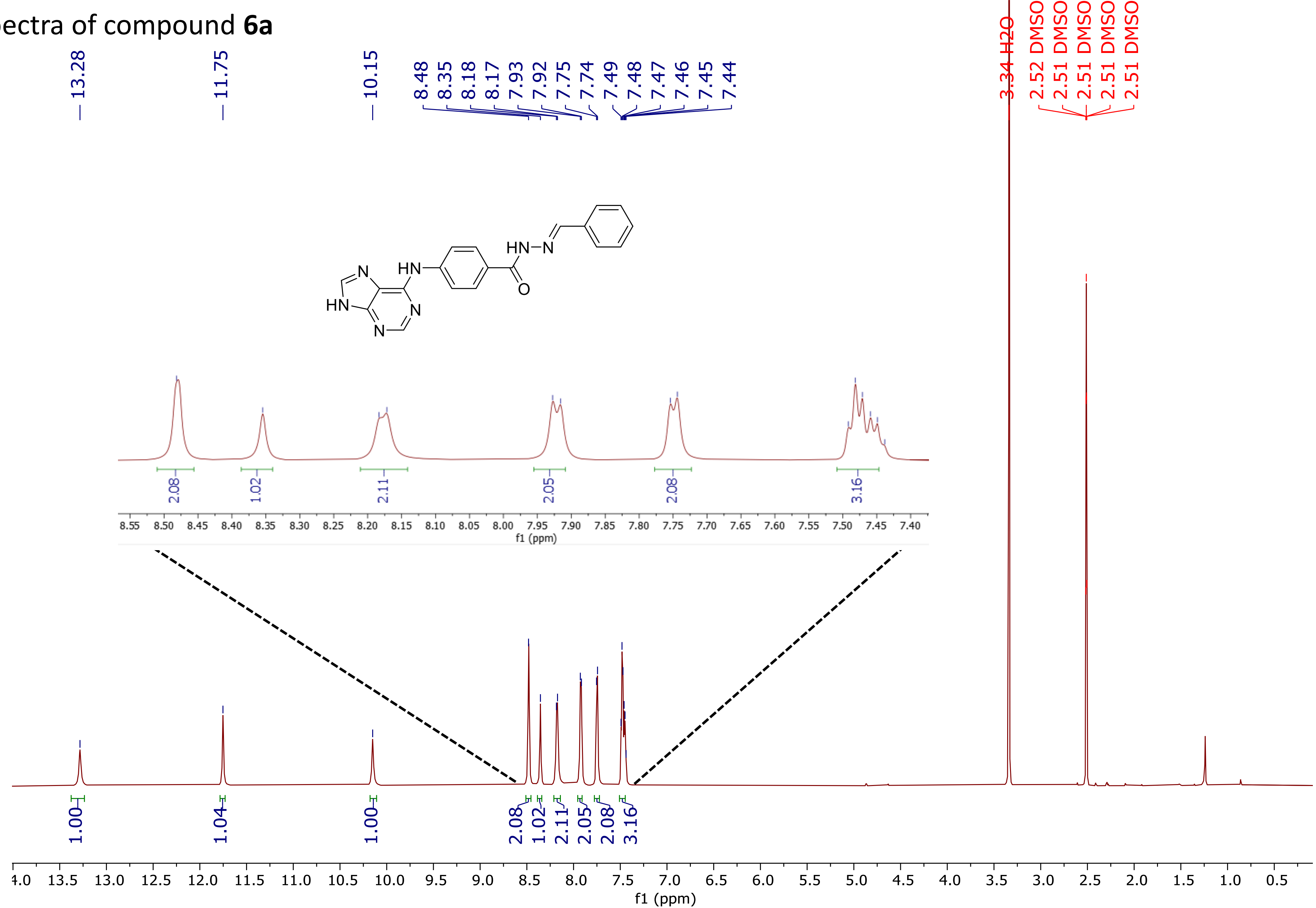

^{13}C-NMR spectra of compound **6a**

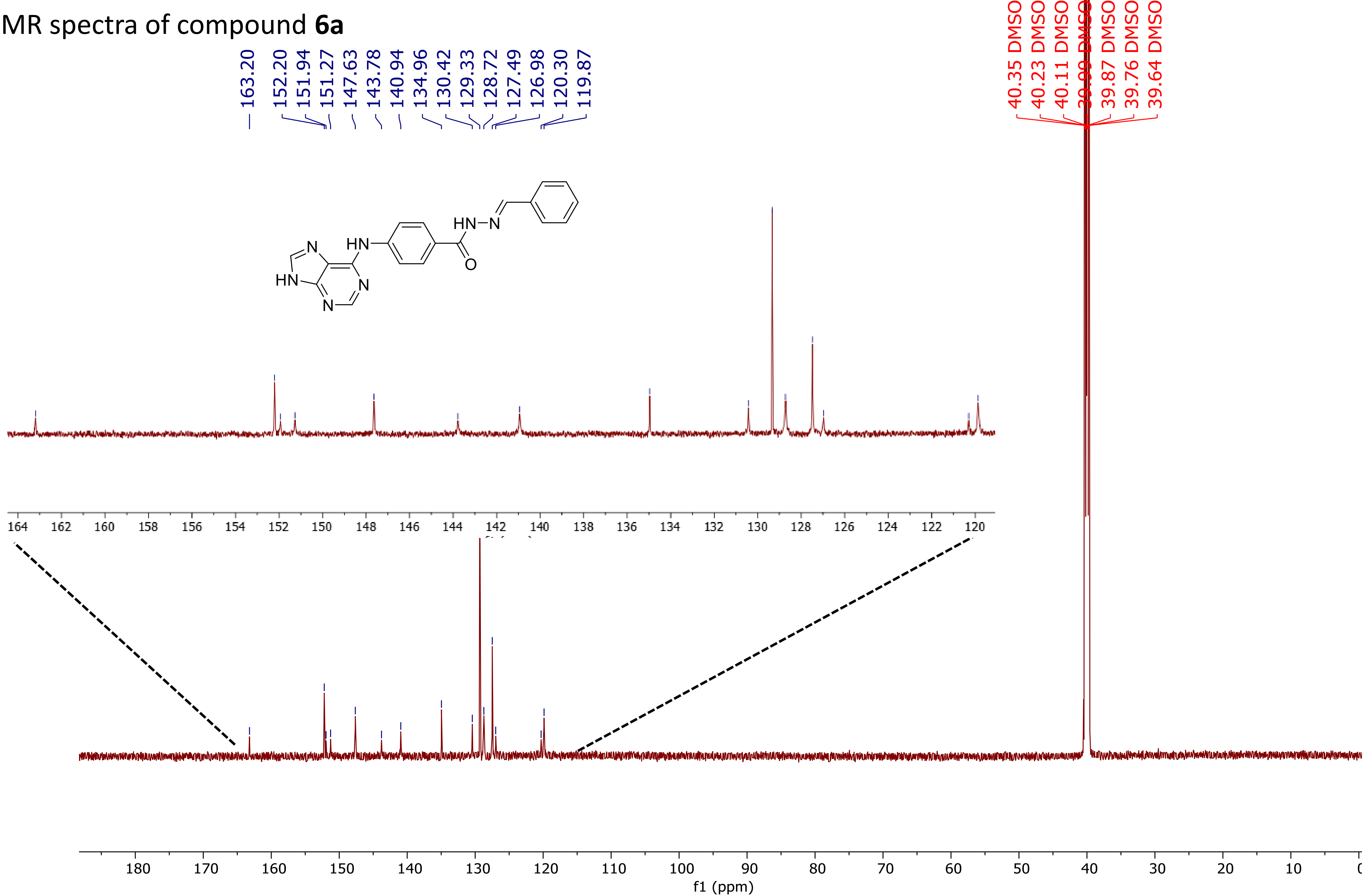

Mass spectra of compound **6a**

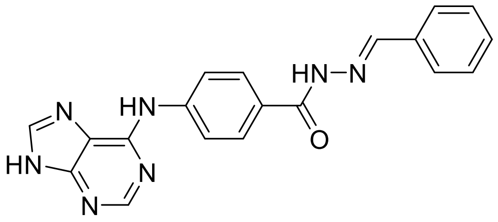

Chemical Formula: $C_{19}H_{15}N_7O$
Molecular Weight: 357

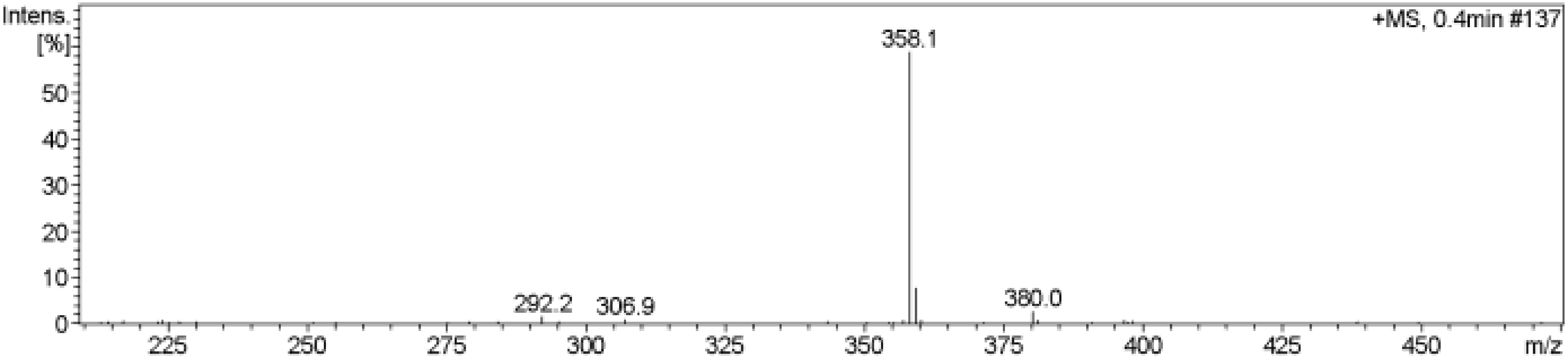

HRMS spectra of compound **6a**

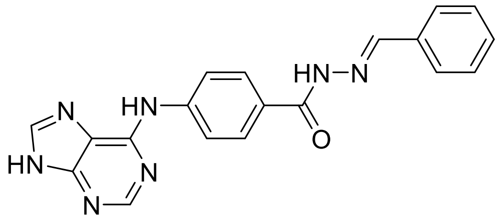

Chemical Formula: $C_{19}H_{15}N_7O$
Exact Mass: 357.1338

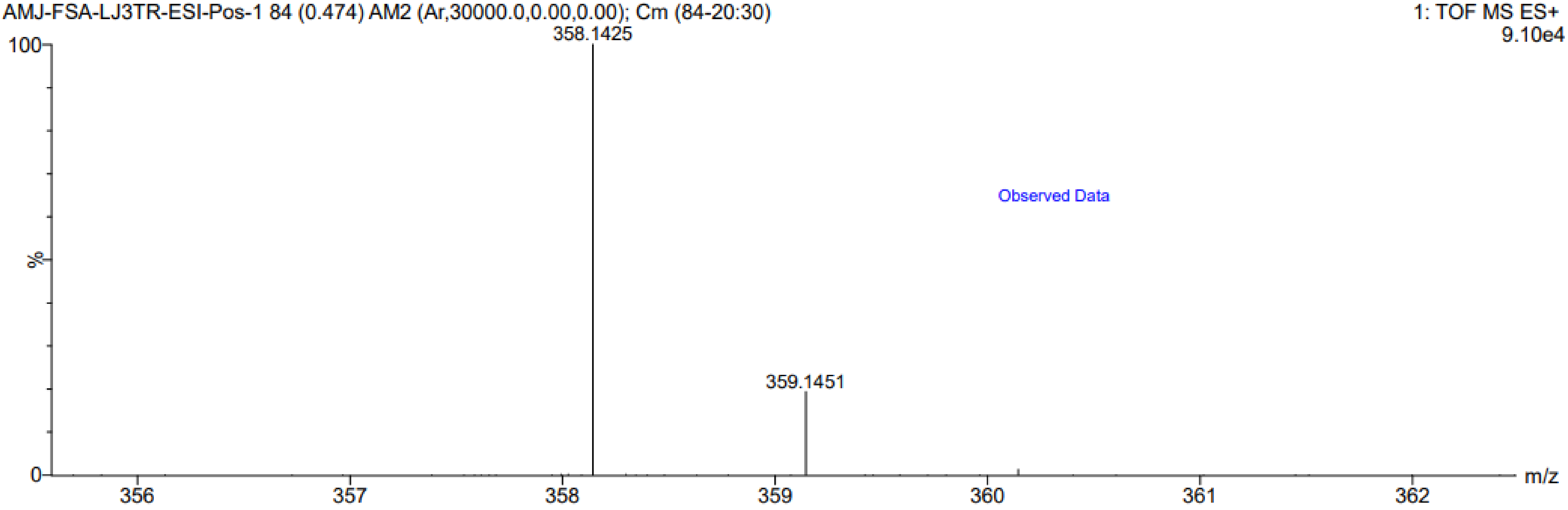

^{1}H-NMR spectra of compound **7a**

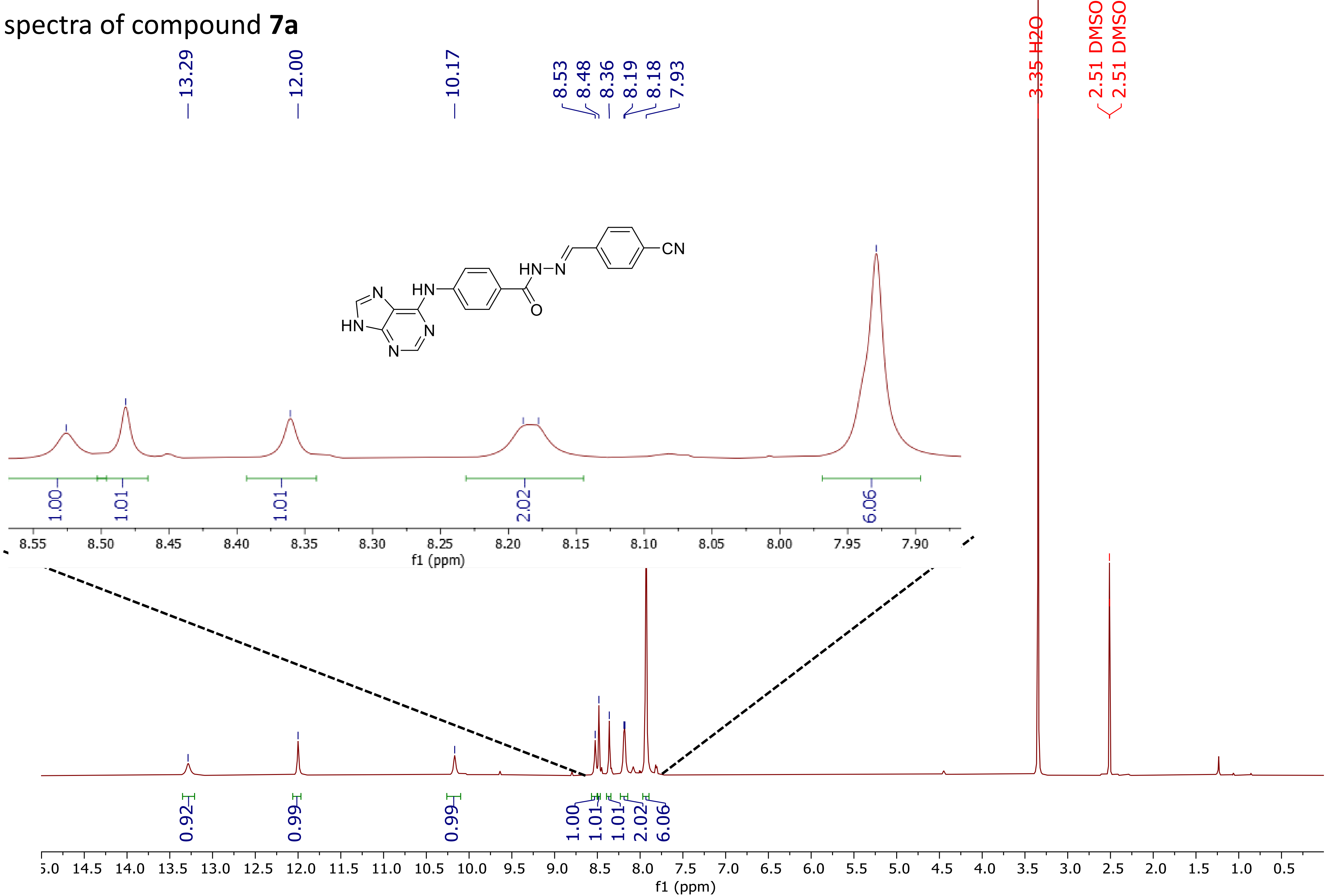

^{13}C-NMR spectra of compound **7a**

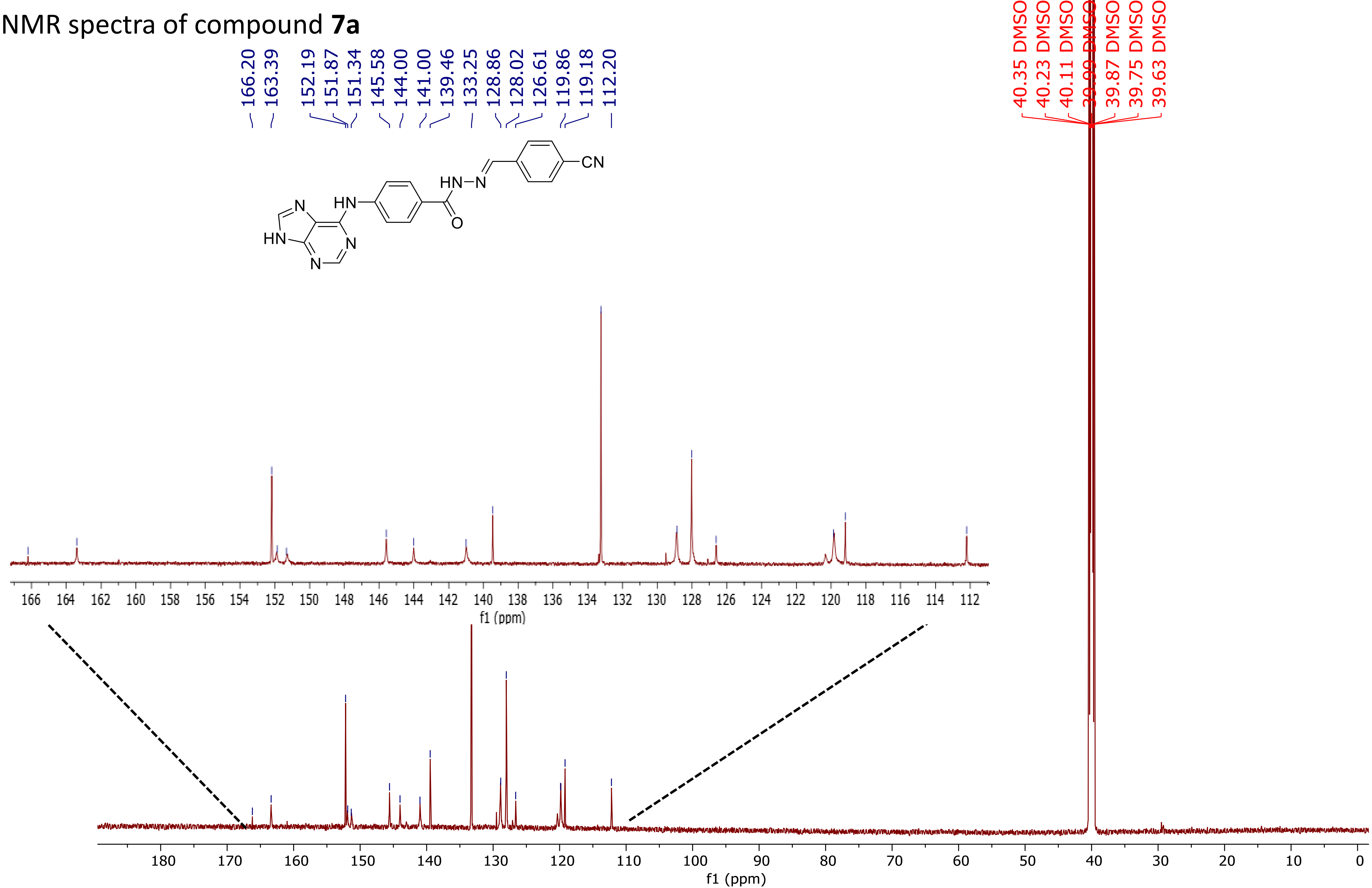

Mass spectra of compound **7a**

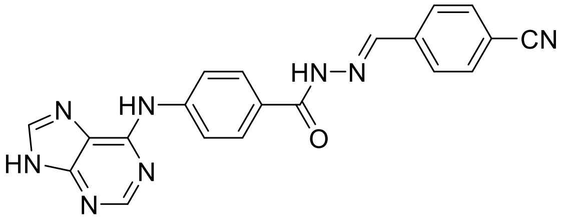

Chemical Formula: $C_{20}H_{14}N_8O$
Molecular Weight: 382

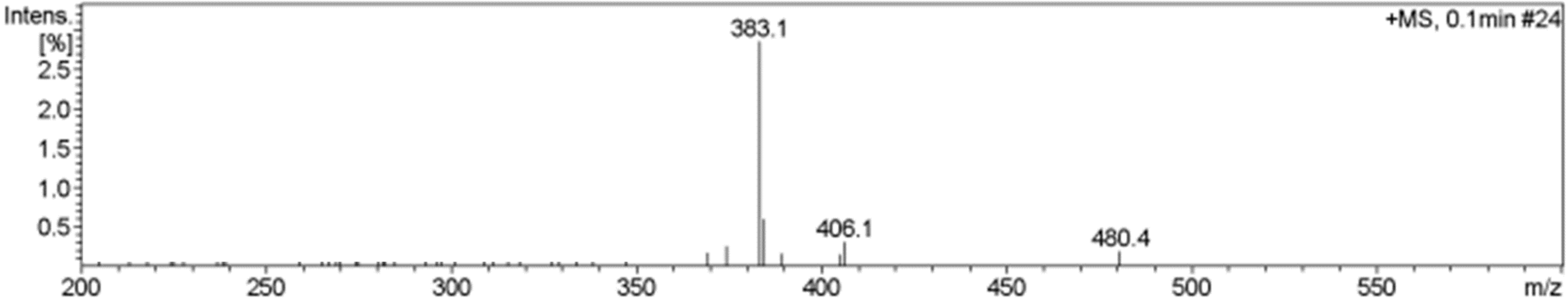

HRMS spectra of compound **7a**

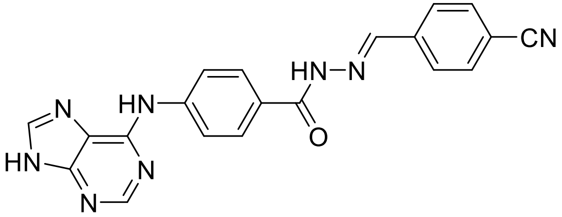

Chemical Formula: $C_{20}H_{14}N_8O$
Exact Mass: 382.1291

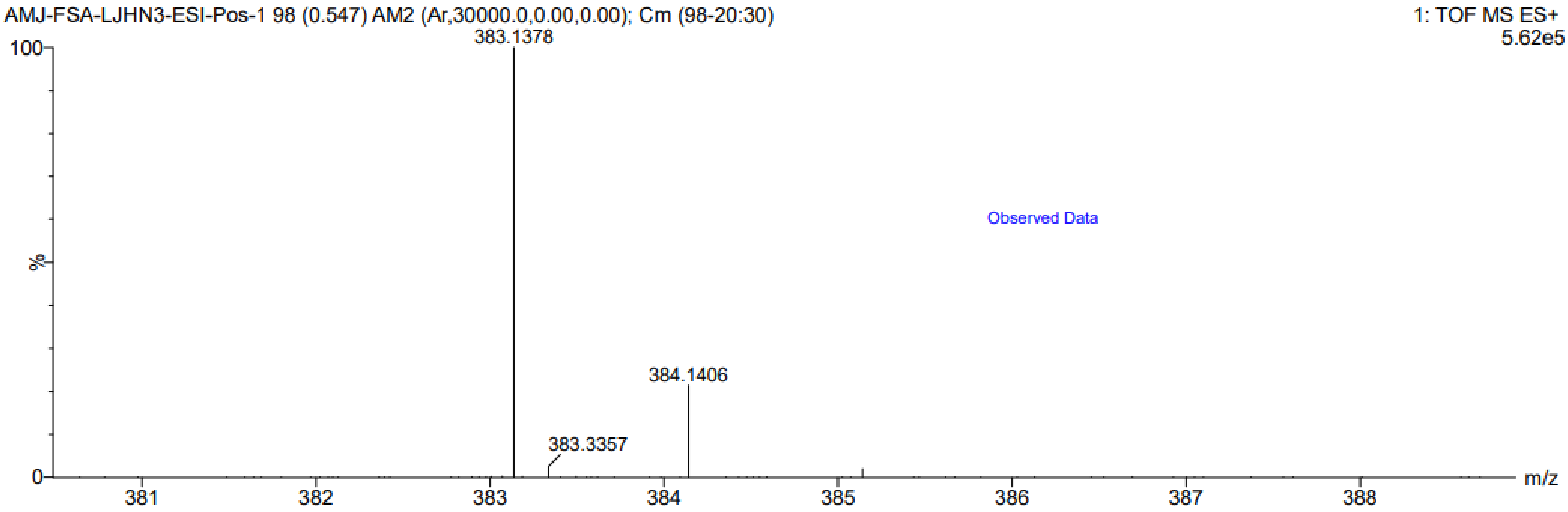

^{1}H-NMR spectra of compound **8a**

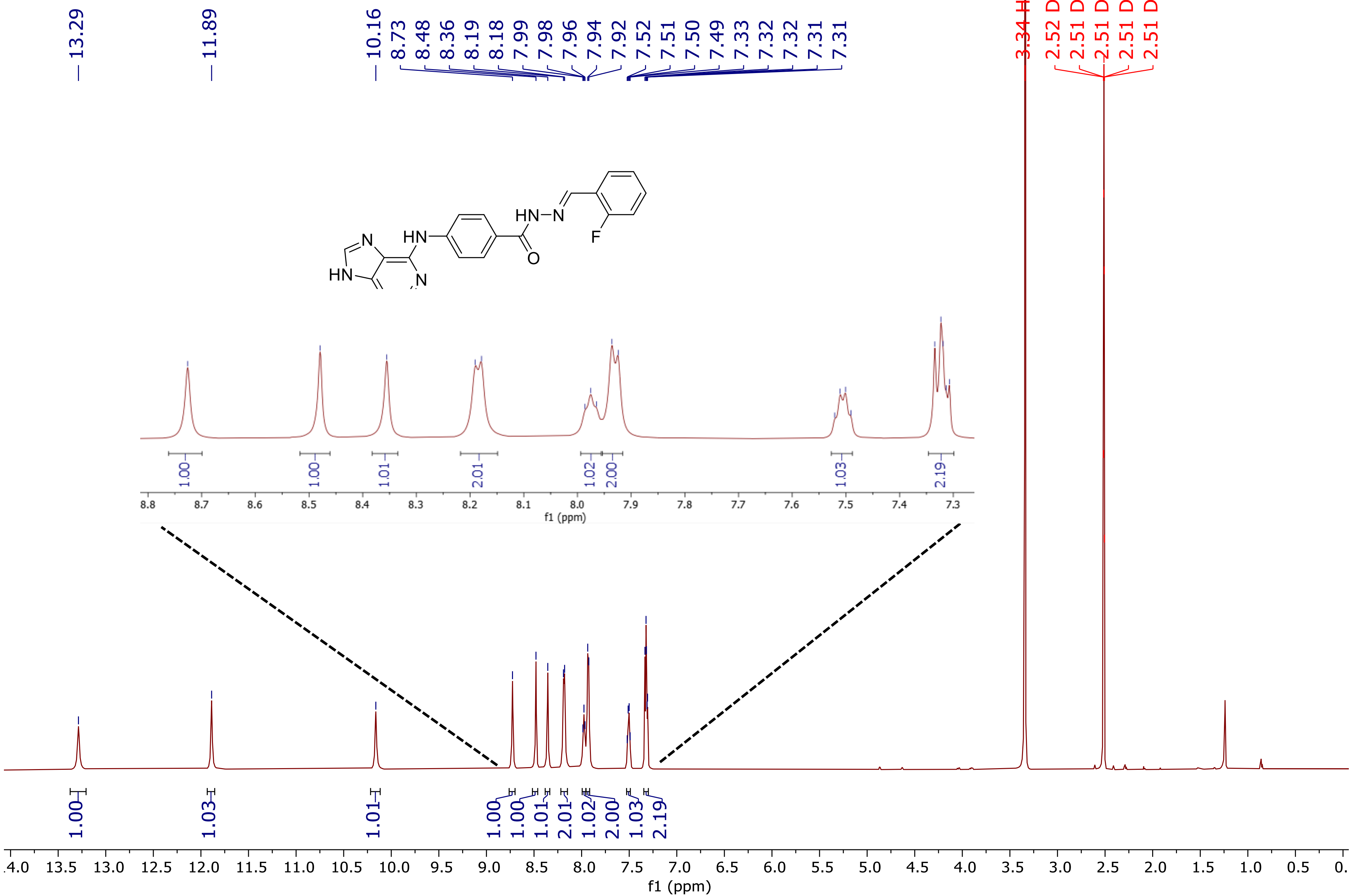

^{13}C-NMR spectra of compound **8a**[5f]

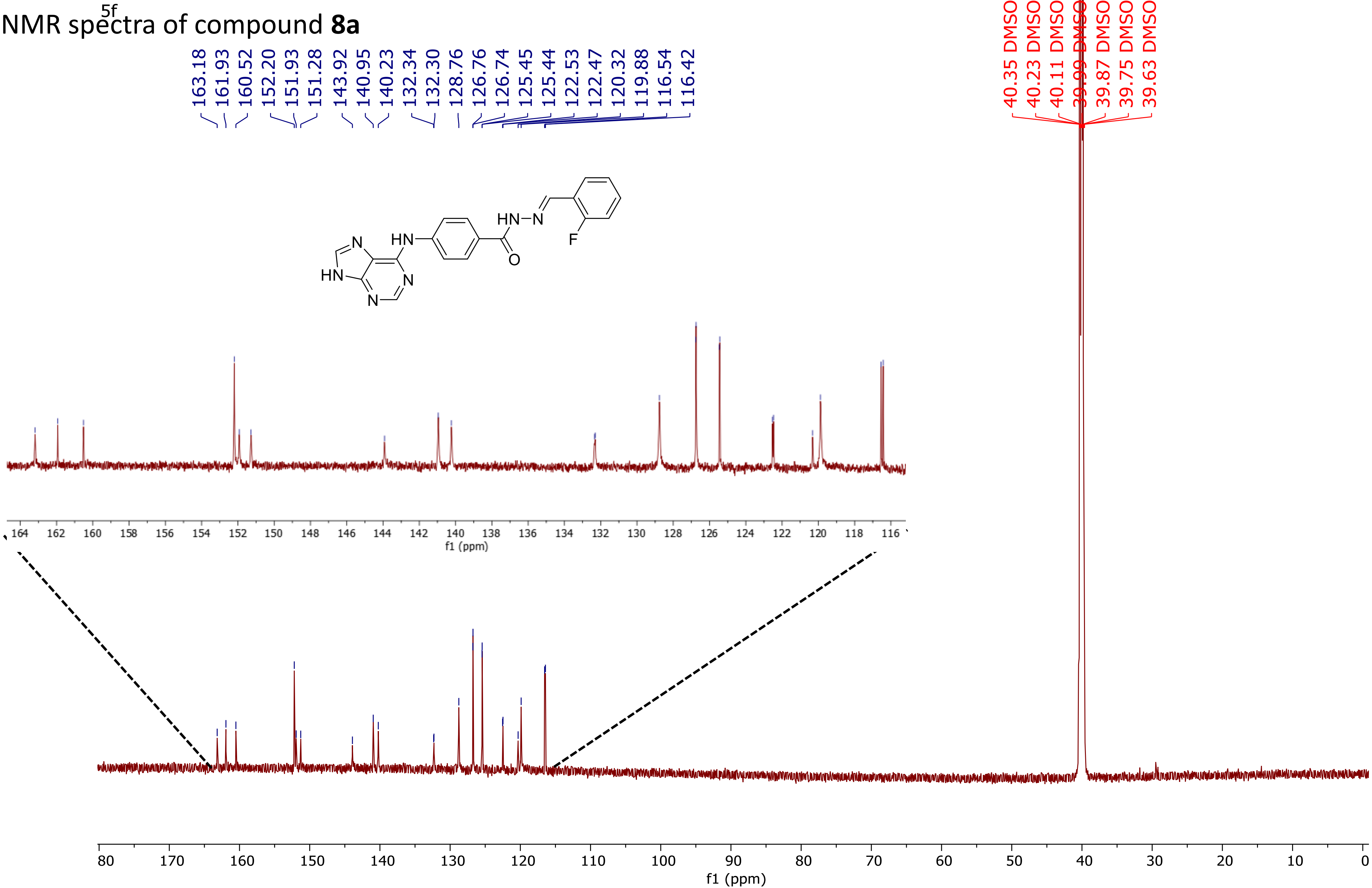

Mass spectra of compound **8a**

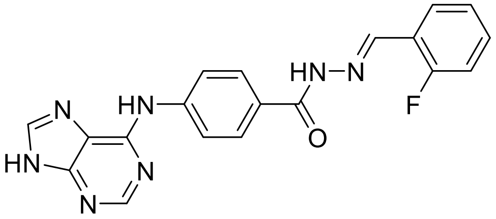

Chemical Formula: $C_{19}H_{14}FN_7O$
Molecular Weight: 375

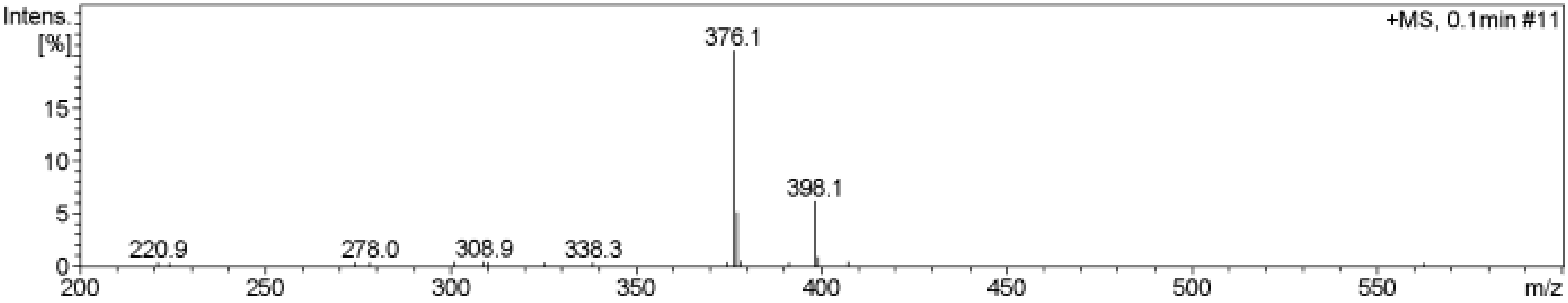

HRMS spectra of compound **8a**

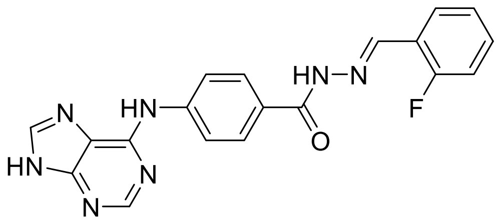

Chemical Formula: $C_{19}H_{14}FN_7O$
Exact Mass: 375.1244

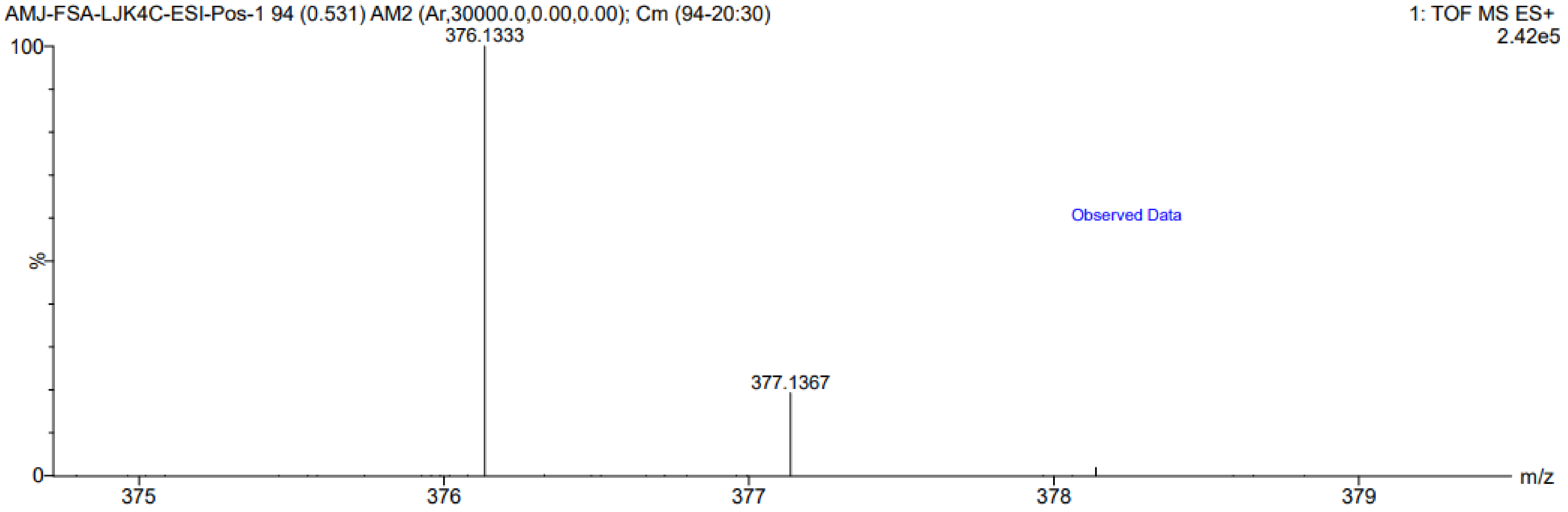

^{1}H-NMR spectra of compound **9a**

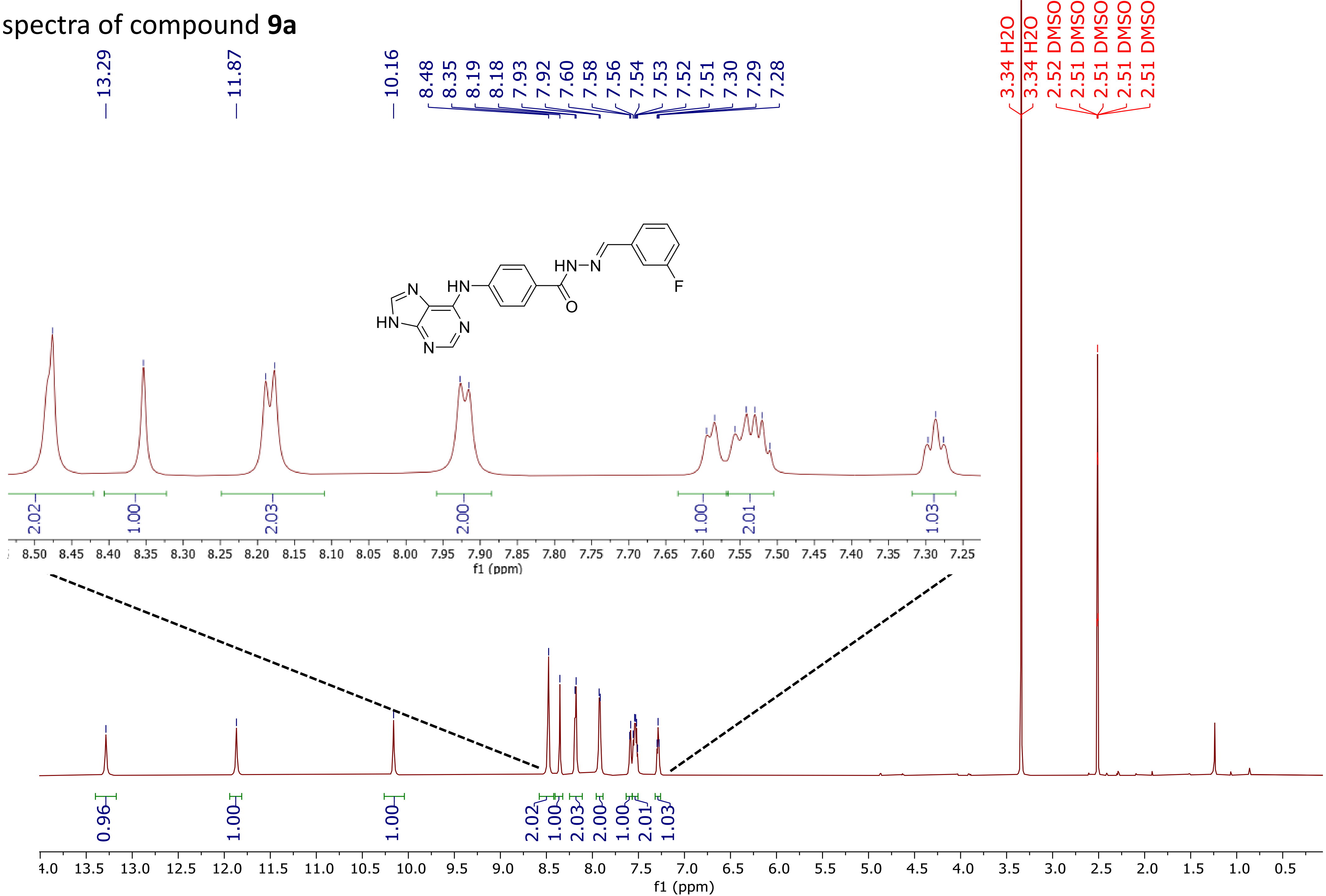

^{13}C-NMR spectra of compound **9a**

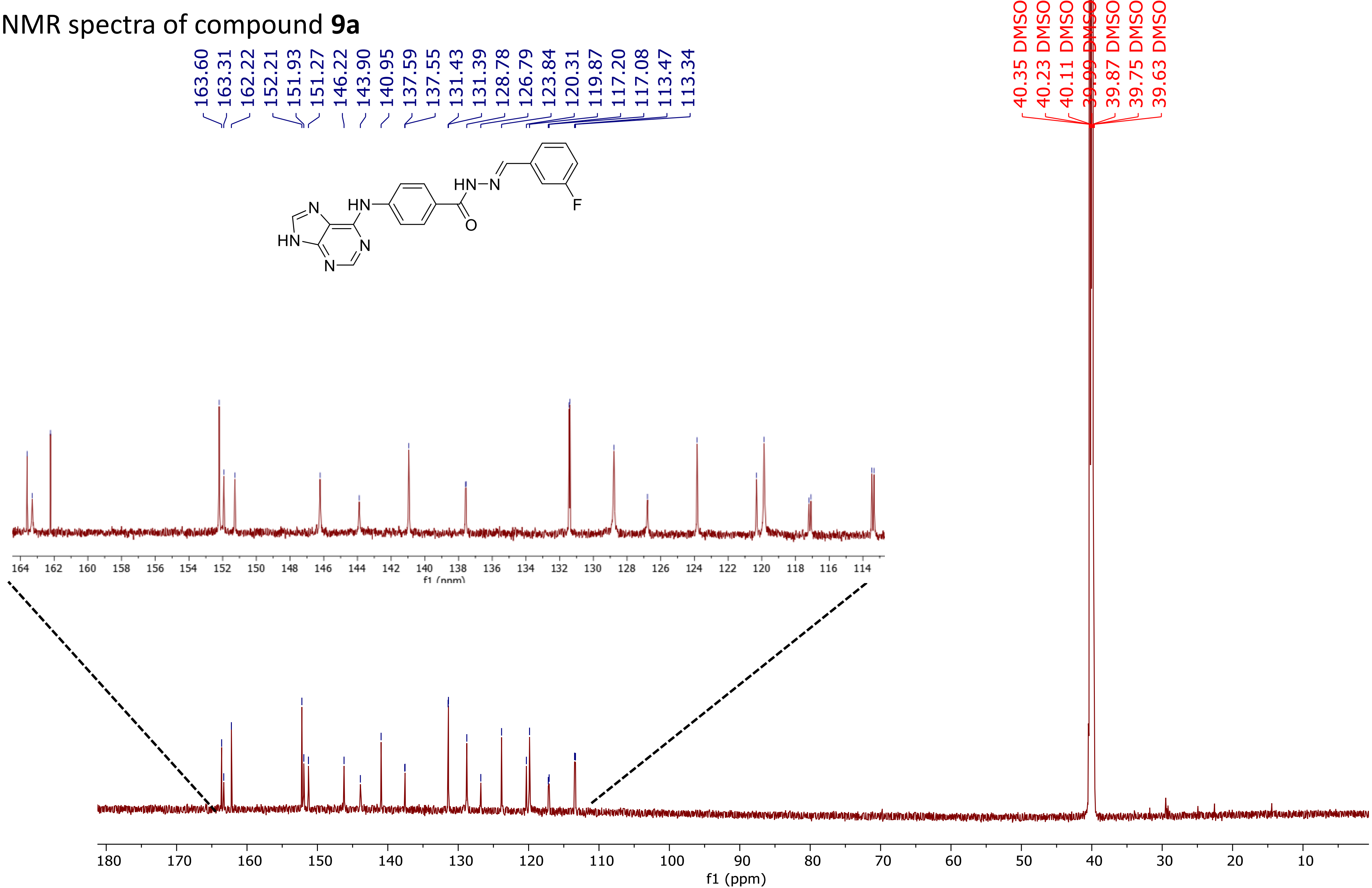

Mass spectra of compound **9a**

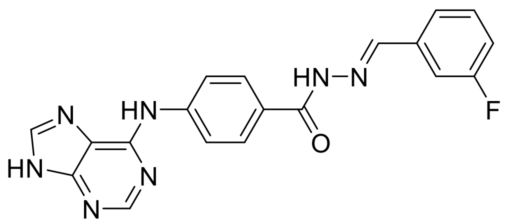

Chemical Formula: $C_{19}H_{14}FN_7O$
Molecular Weight: 375

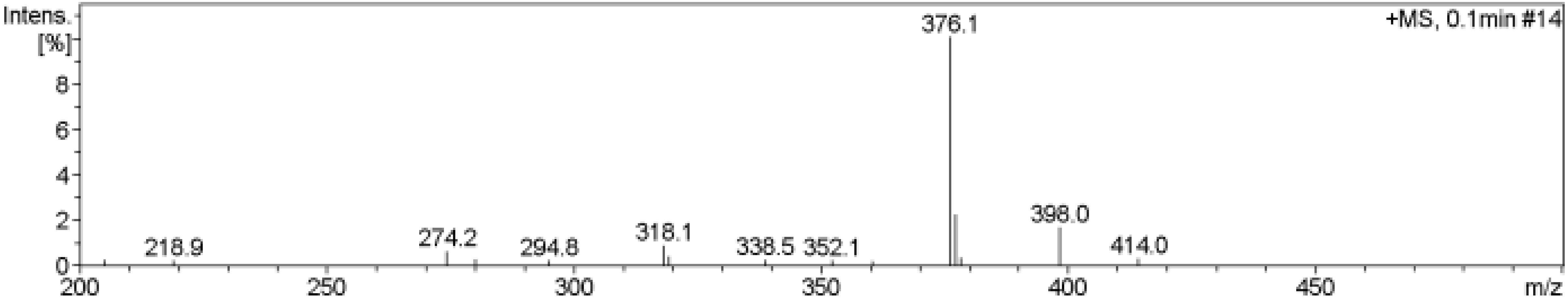

Mass spectra of compound **9a**

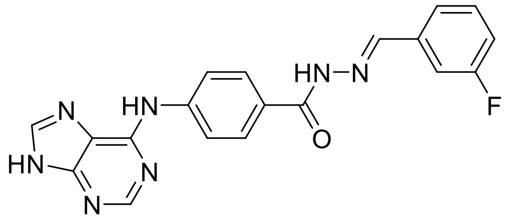

Chemical Formula: $C_{19}H_{14}FN_7O$
Exact Mass: 375.1244

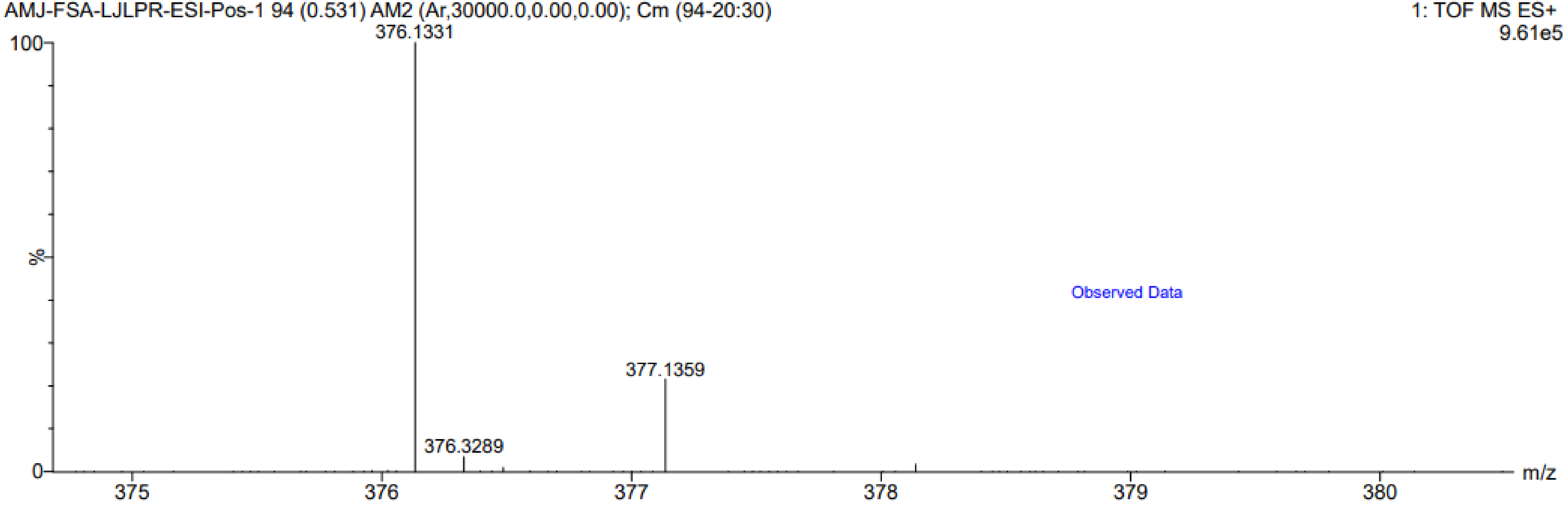

^{1}H-NMR spectra of compound **10a**

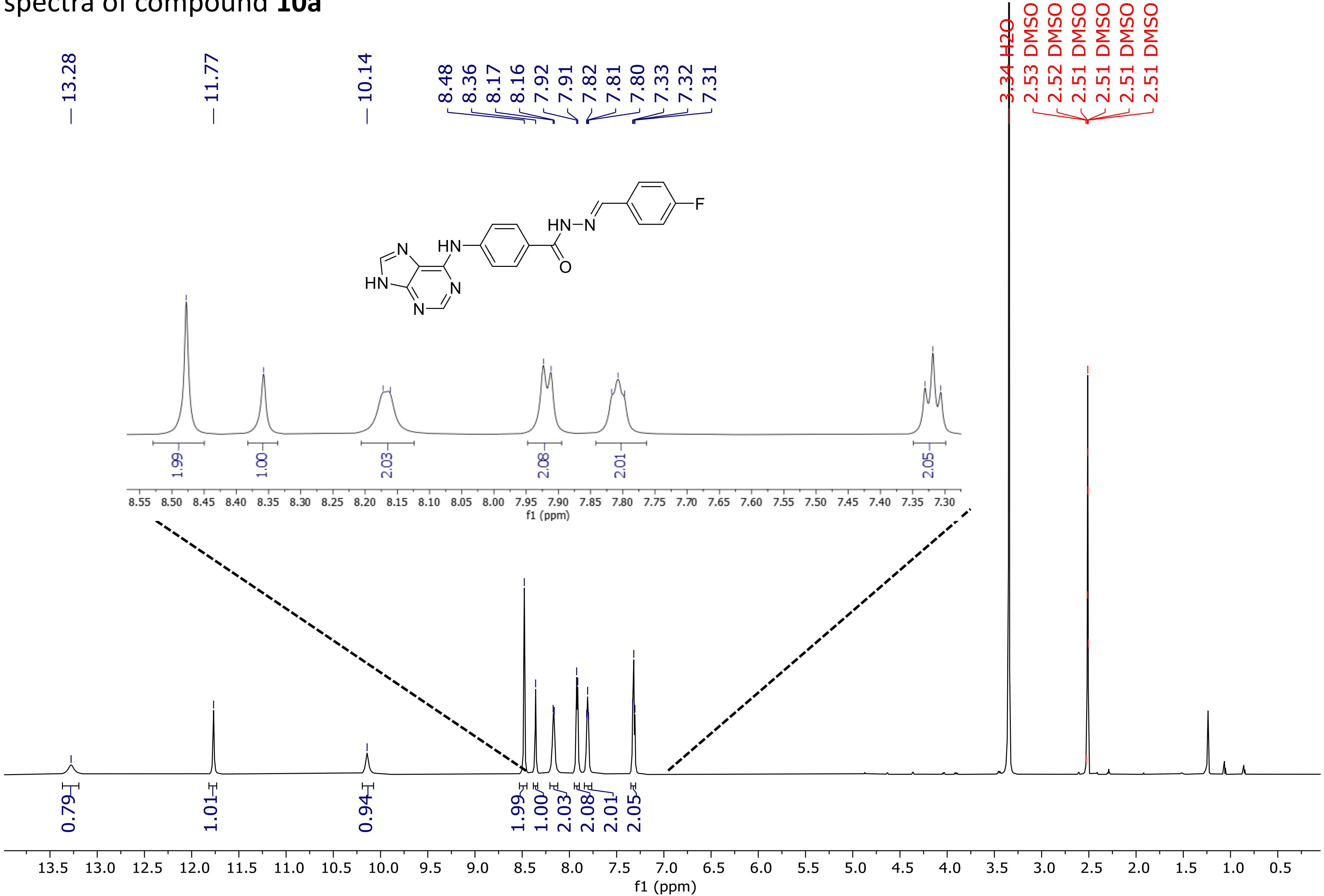

^{13}C-NMR spectra of compound **10a**

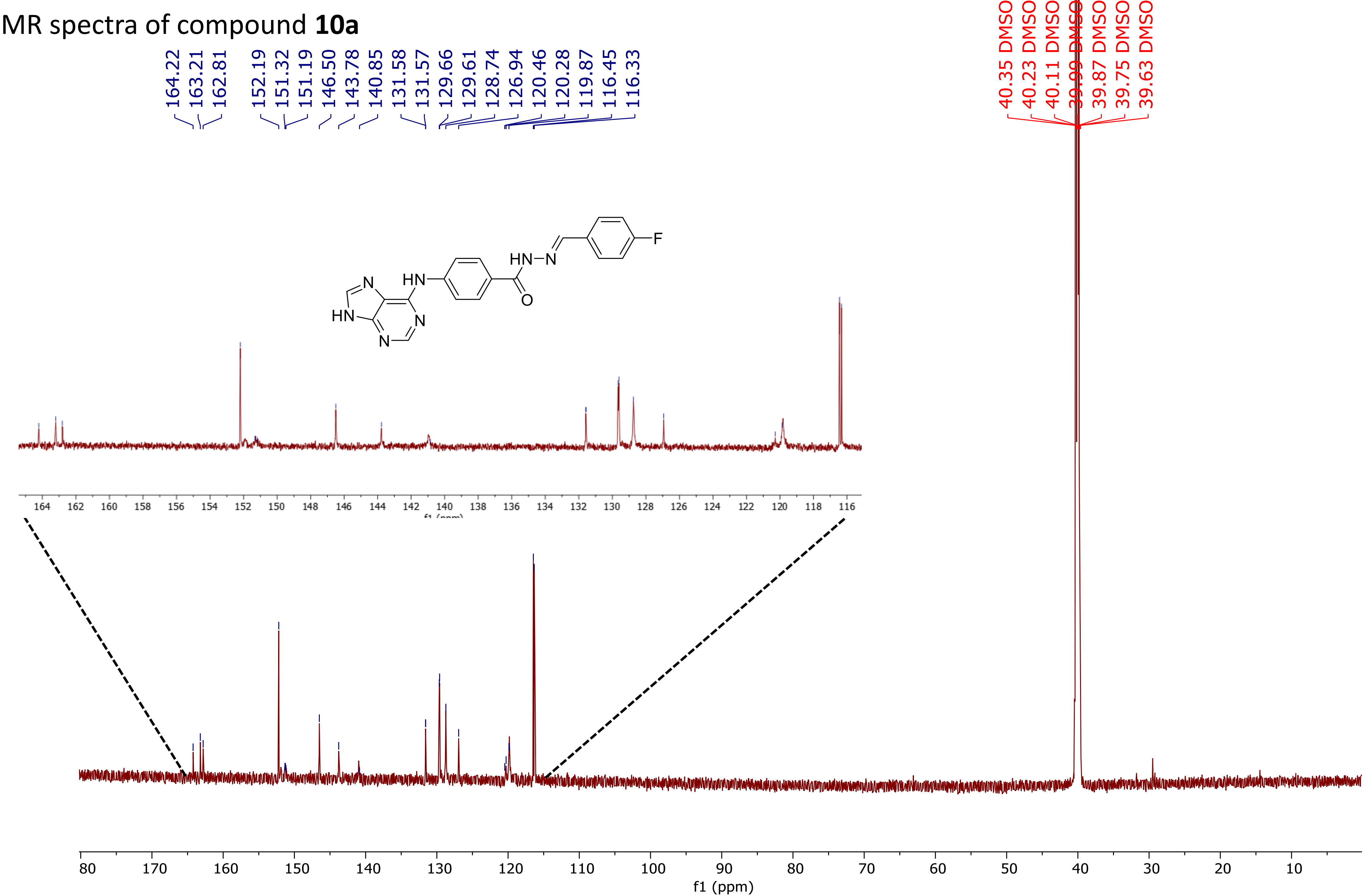

Mass spectra of compound **10a**

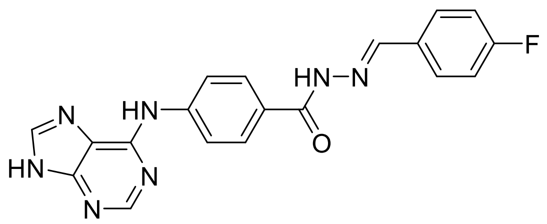

Chemical Formula: $C_{19}H_{14}FN_7O$
Molecular Weight: 375

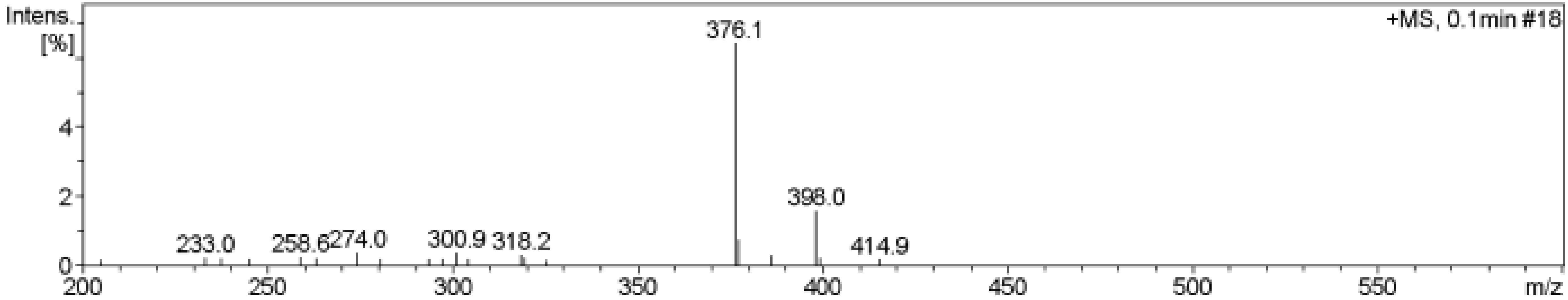

Mass spectra of compound **10a**

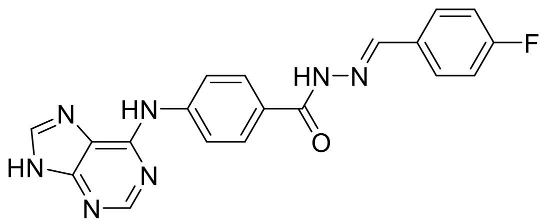

Chemical Formula: $C_{19}H_{14}FN_7O$
Exact Mass: 375.1244

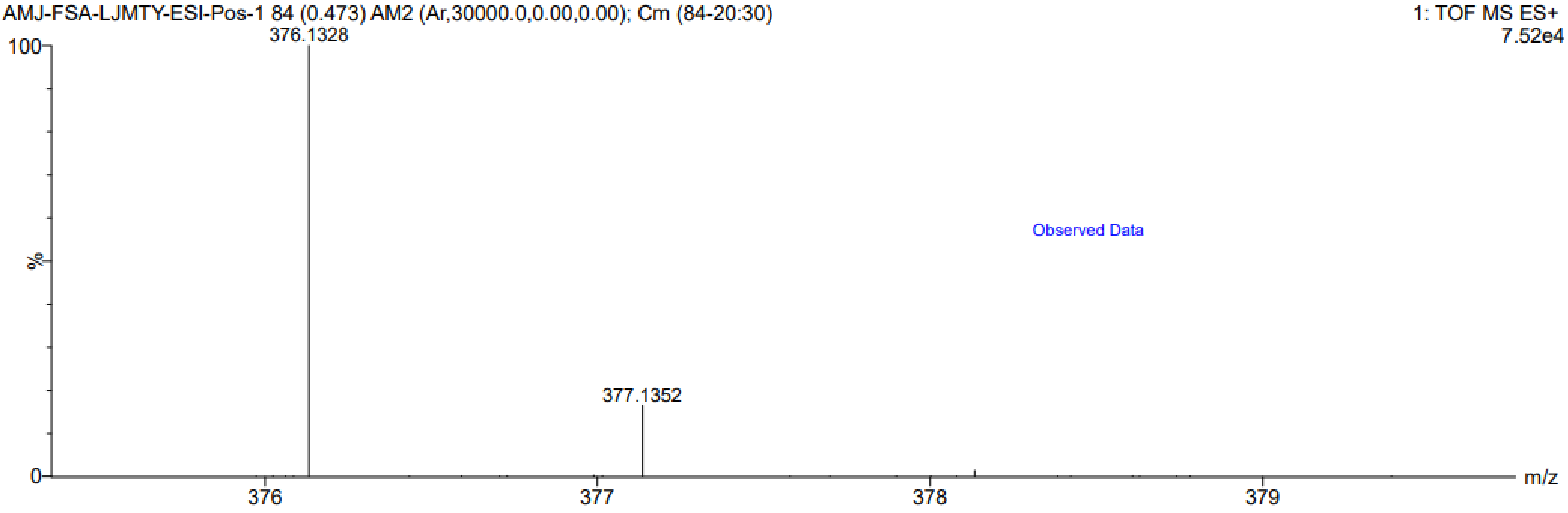

^{1}H-NMR spectra of compound **11a**

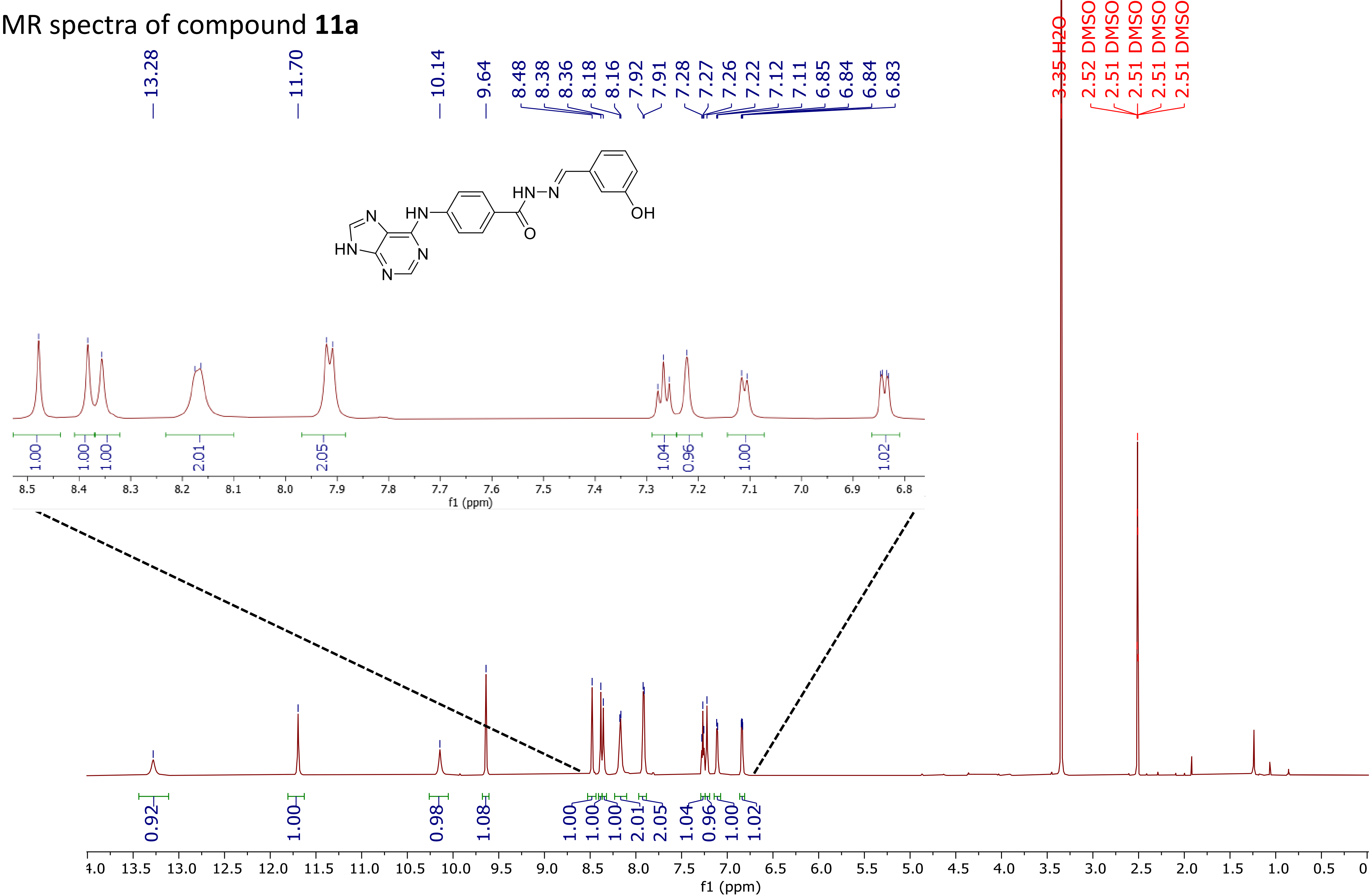

^{13}C-NMR spectra of compound **11a**

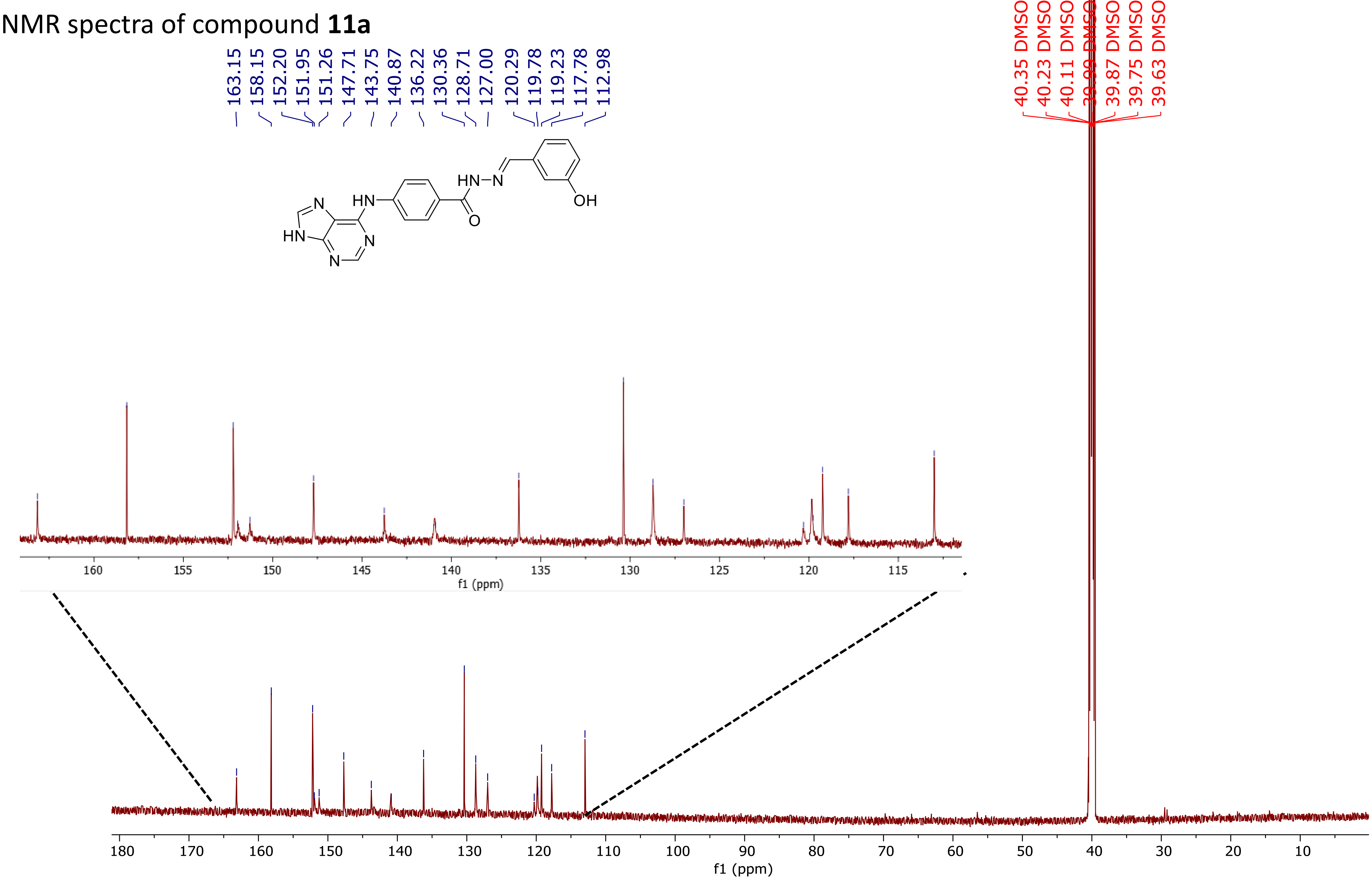

Mass spectra of compound **11a**

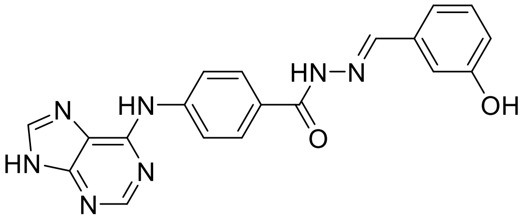

Chemical Formula: $C_{19}H_{15}N_7O_2$
Molecular Weight: 373

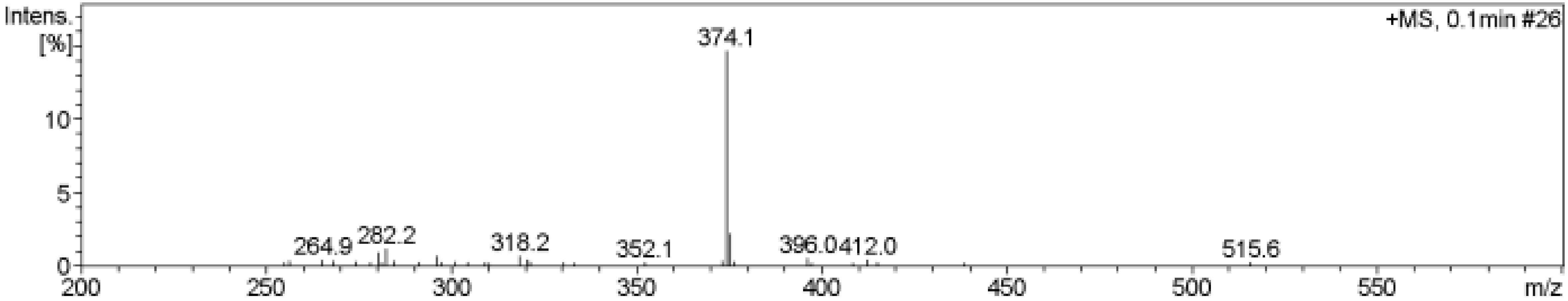

HRMS spectra of compound **11a**

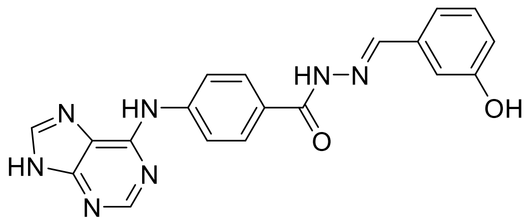

Chemical Formula: $C_{19}H_{15}N_7O_2$
Exact Mass: 373.1287

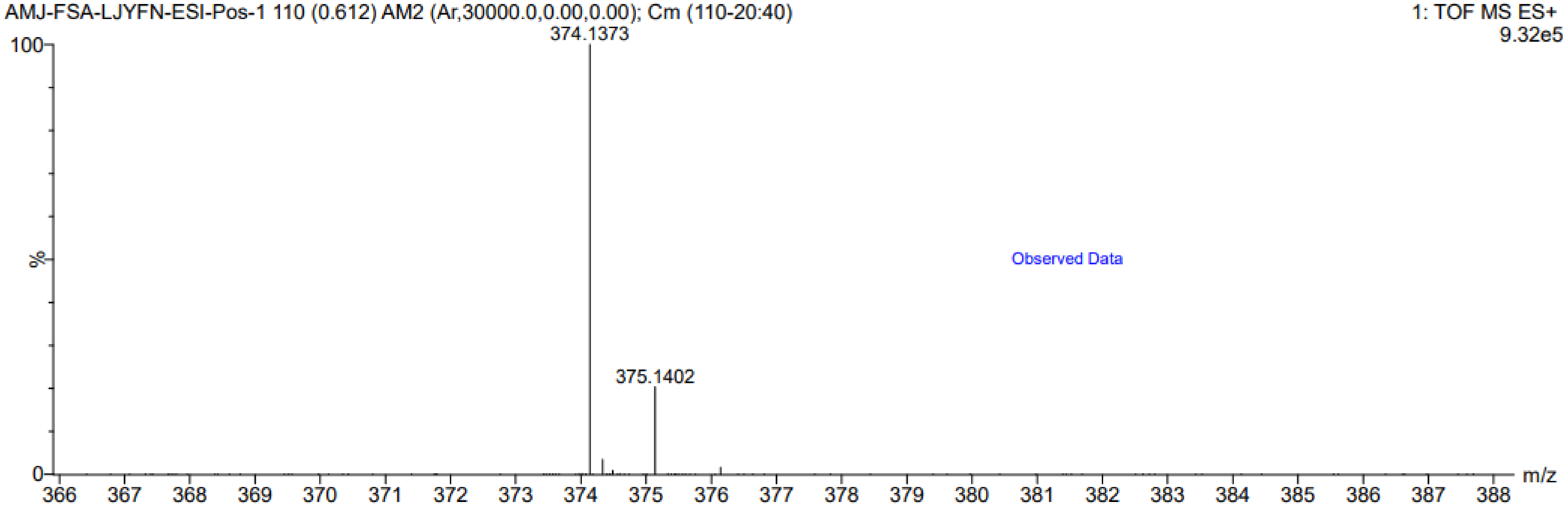

^{1}H-NMR spectra of compound **12a**

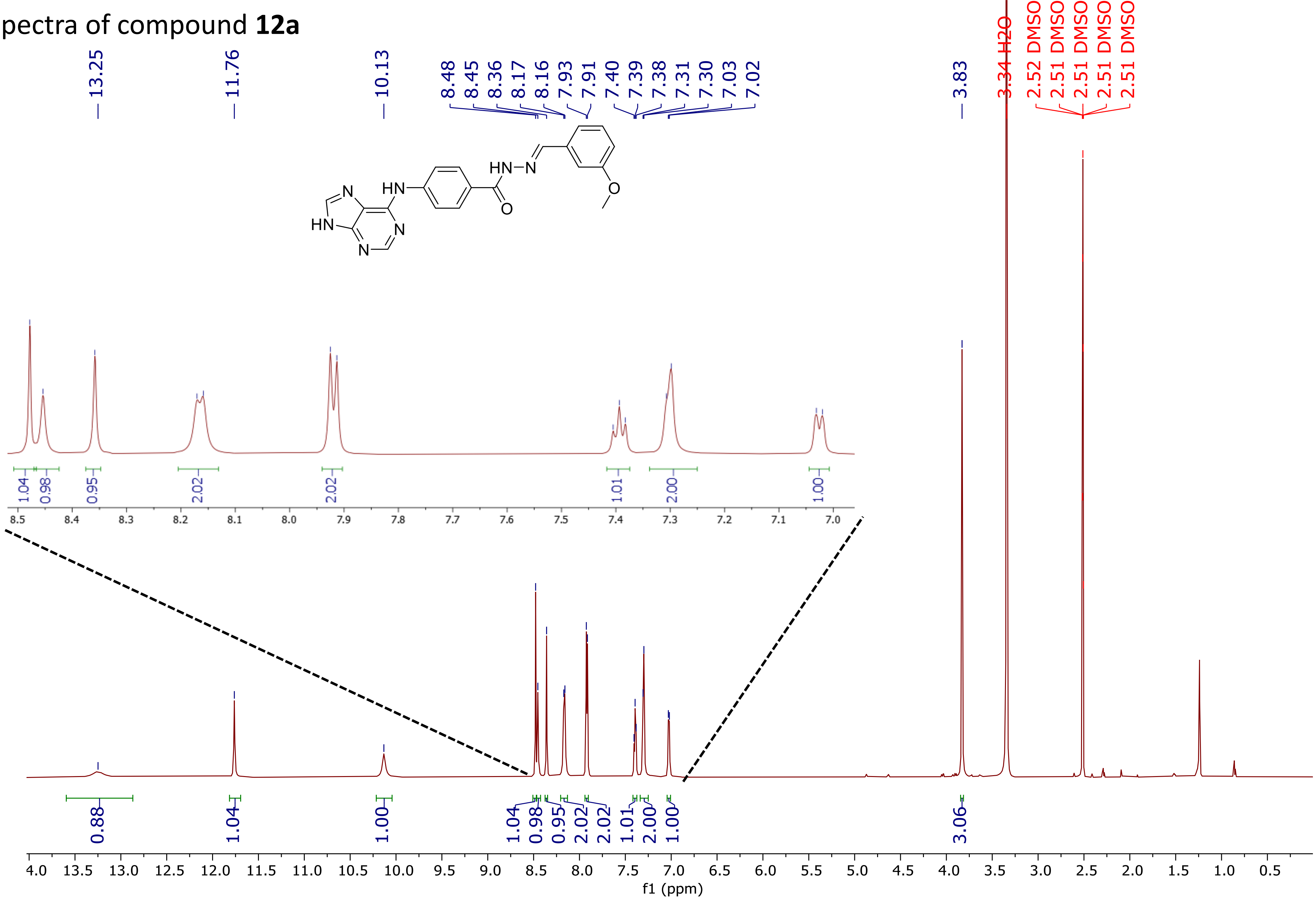

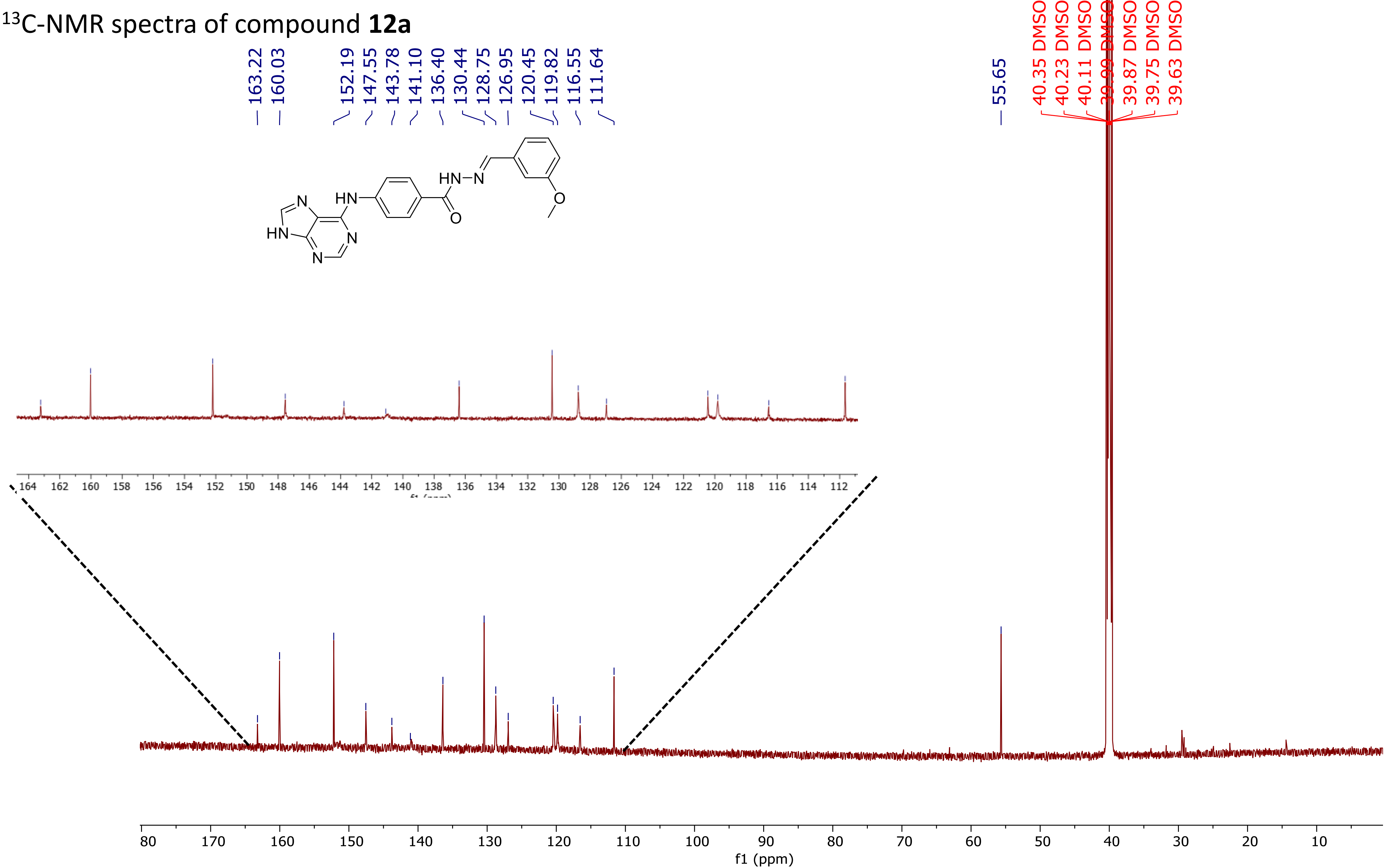

163.22
160.03
152.19
147.55
143.78
141.10
136.40
130.44
128.75
126.95
120.45
119.82
116.55
111.64
55.65
40.35 DMSO
40.23 DMSO
40.11 DMSO
39.99 DMSO
39.87 DMSO
39.75 DMSO
39.63 DMSO
HN
N
N
N
HN
HN
N
O
O
f1 (ppm)

Mass spectra of compound **12a**

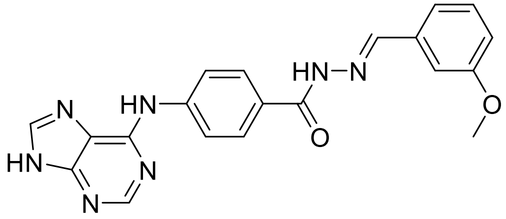

Chemical Formula: $C_{20}H_{17}N_{7}O_{2}$
Molecular Weight: 387

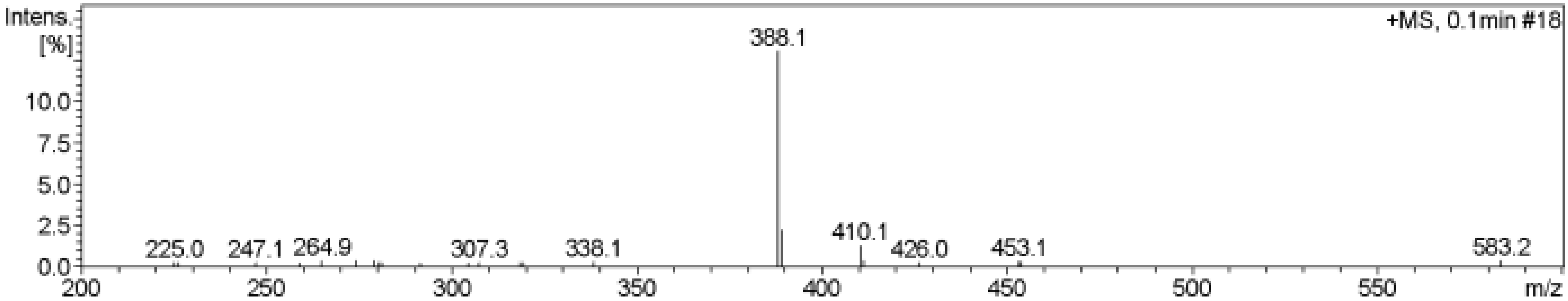

HRMS spectra of compound **12a**

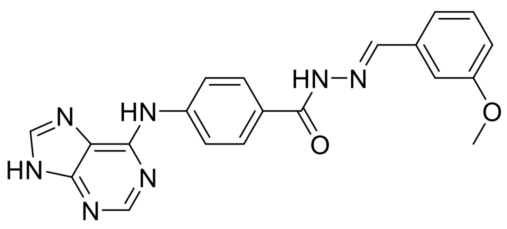

Chemical Formula: $C_{20}H_{17}N_7O_2$
Molecular Weight: 387

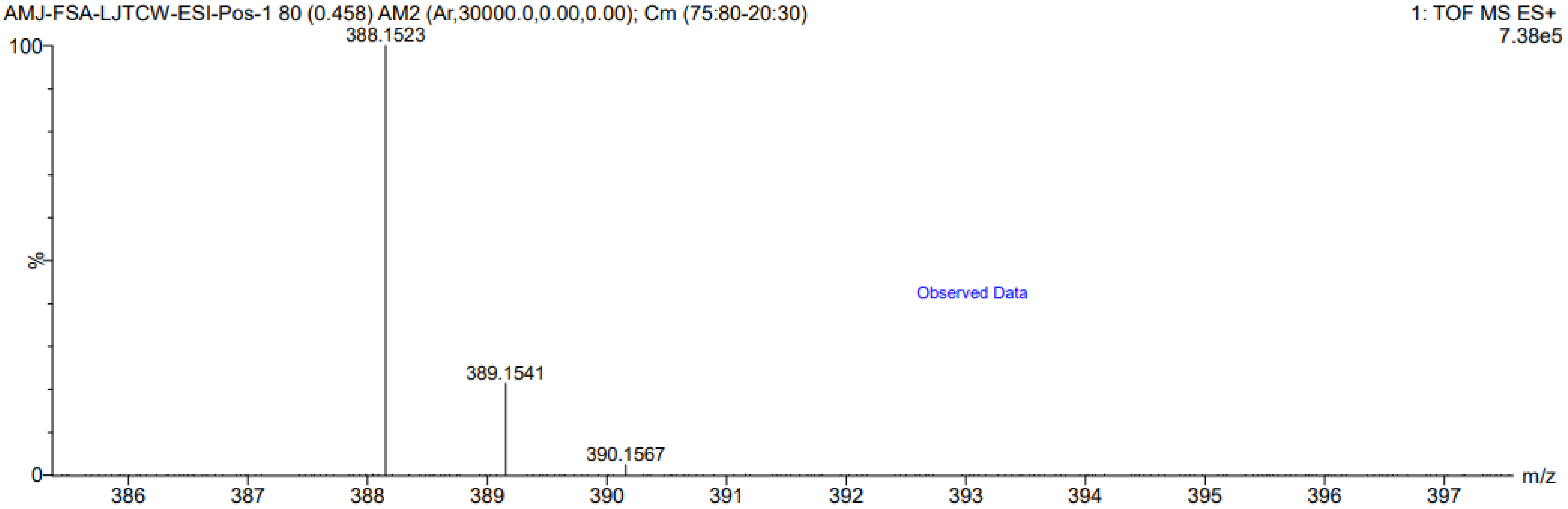

^{1}H-NMR spectra of compound **13a**

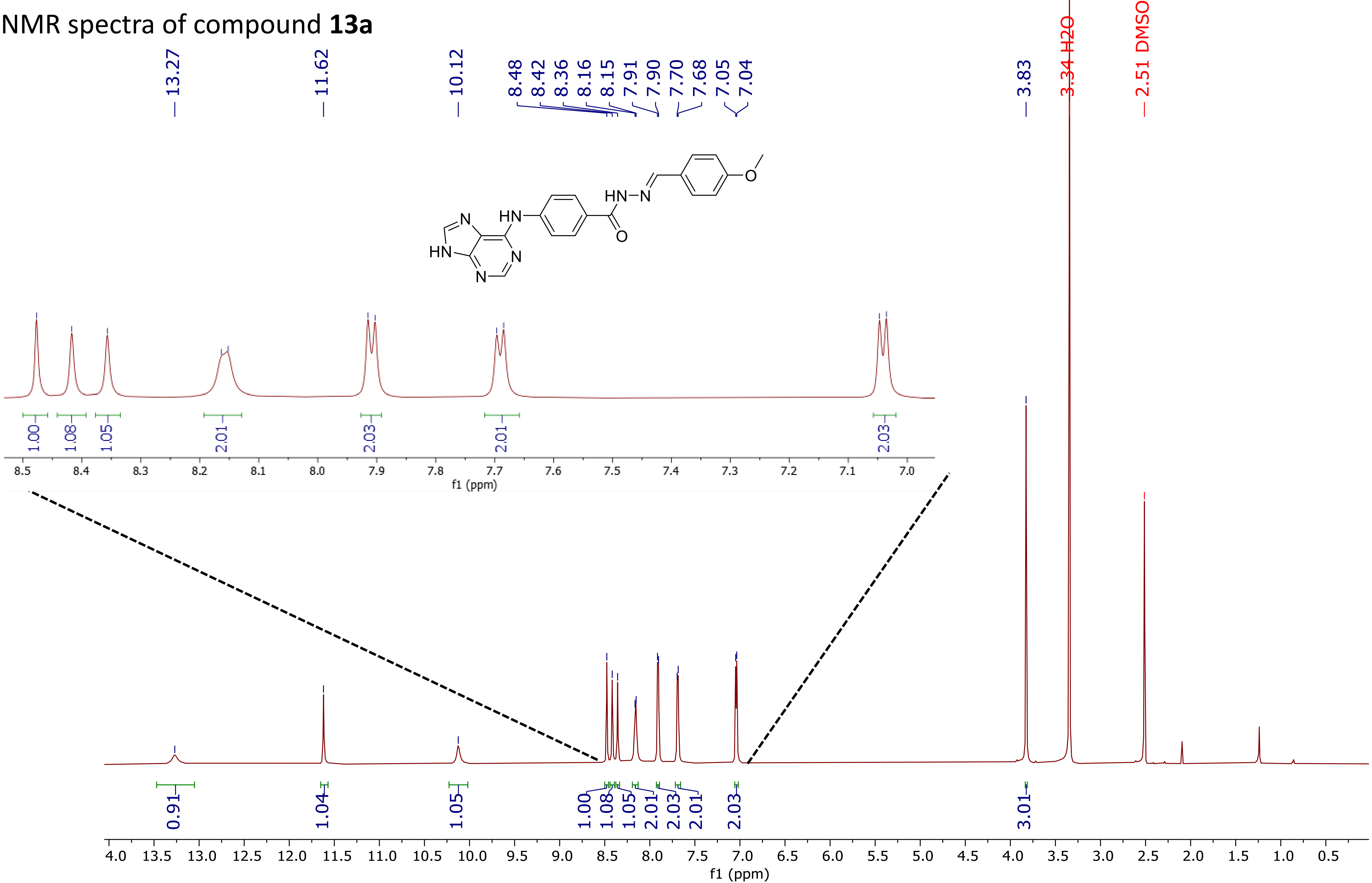

^{13}C-NMR spectra of compound **13a**

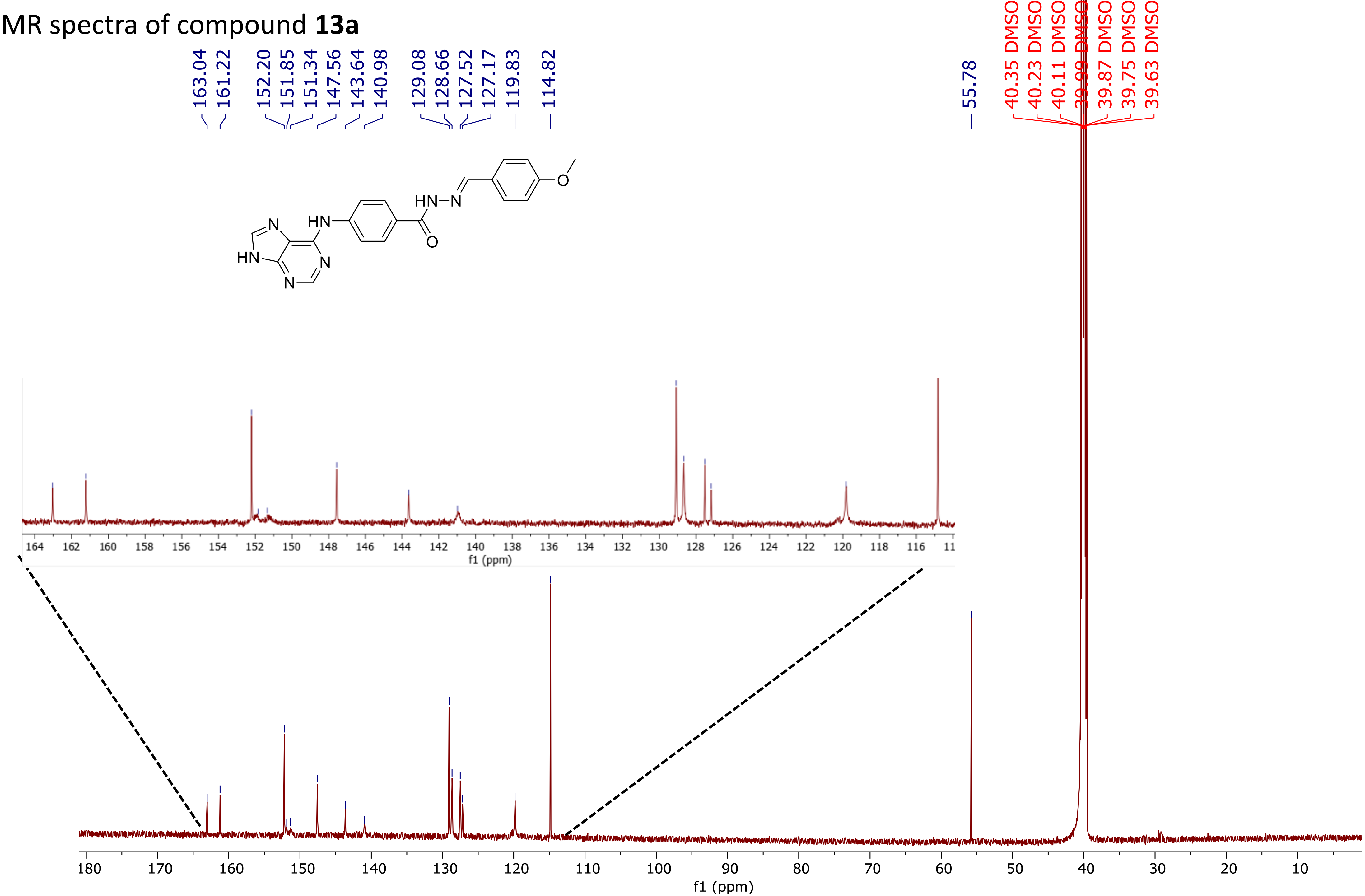

Mass spectra of compound **13a**

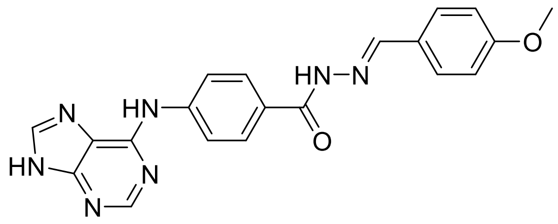

Chemical Formula: $C_{20}H_{17}N_7O_2$
Molecular Weight: 387

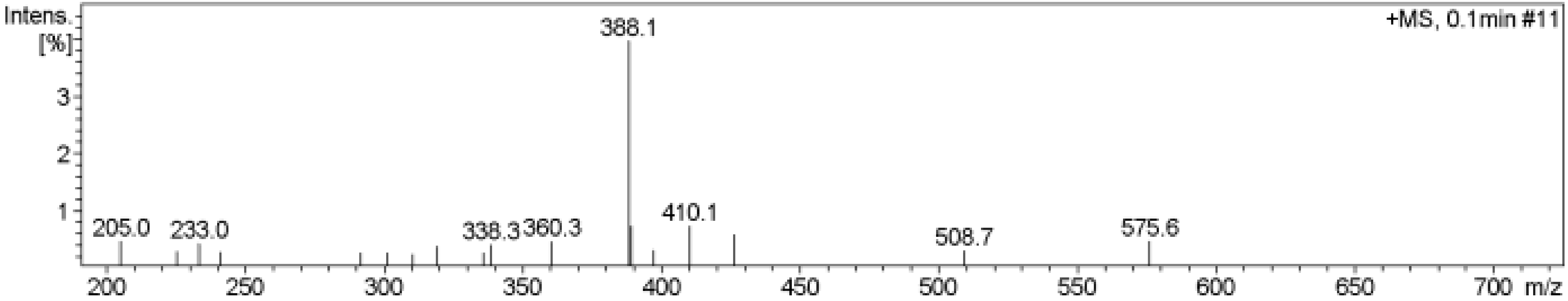

HRMS spectra of compound **13a**

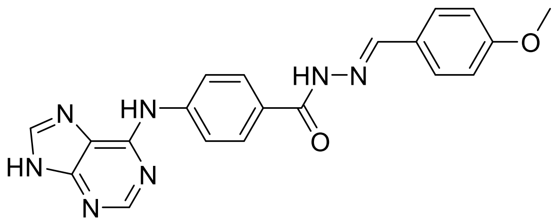

Chemical Formula: $C_{20}H_{17}N_7O_2$
Molecular Weight: 387

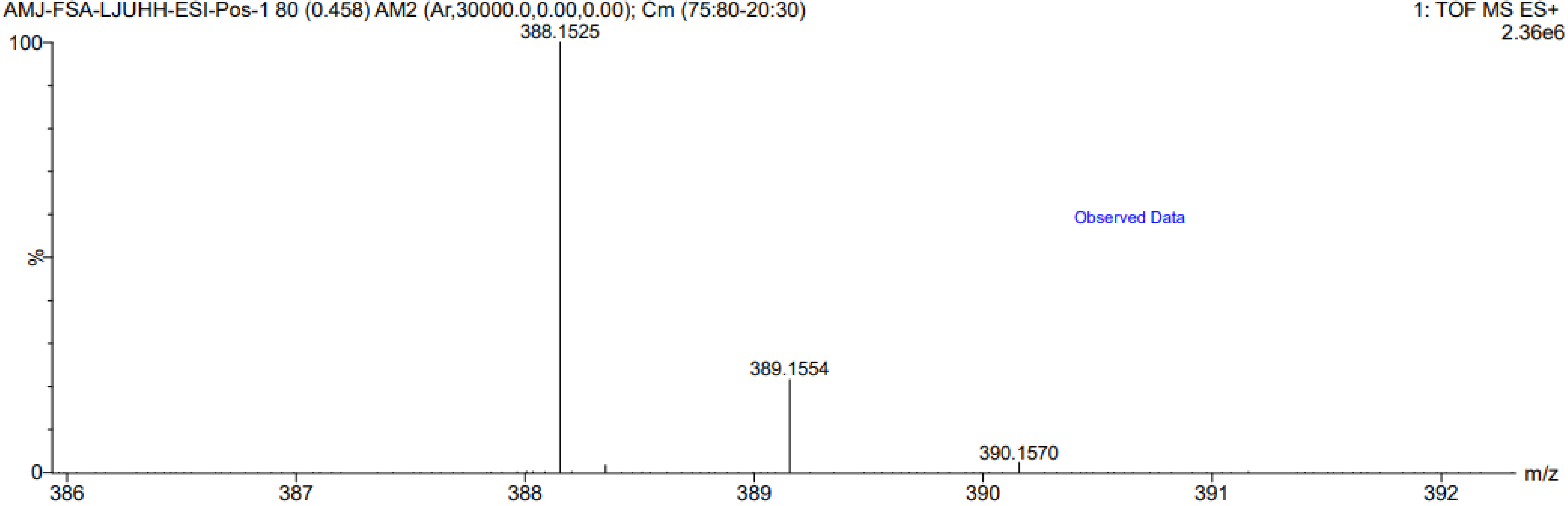

^{1}H-NMR spectra of compound **14a**

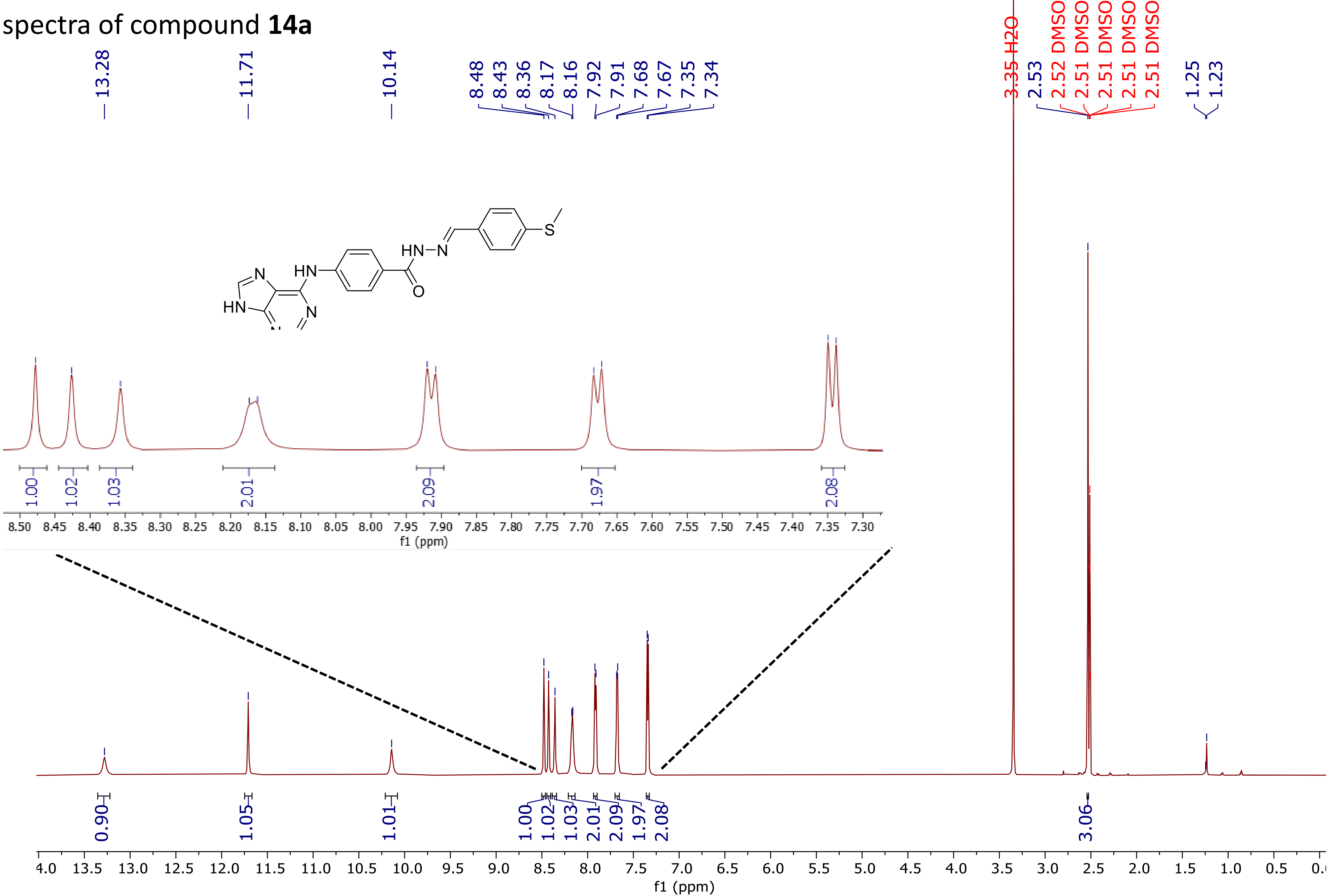

^{13}C-NMR spectra of compound **14a**

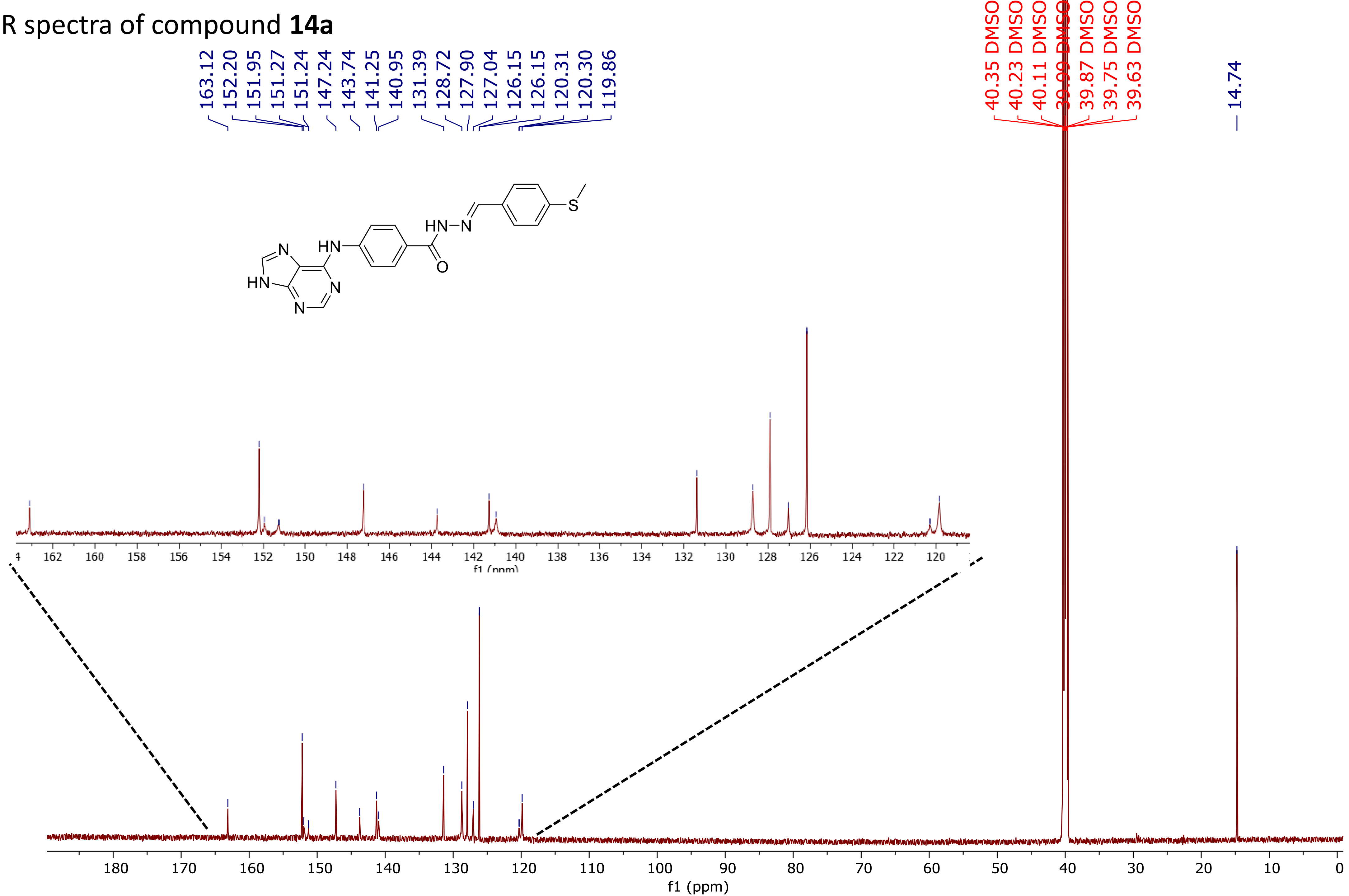

Mass spectra of compound **14a**

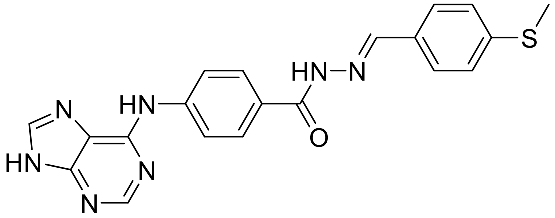

Chemical Formula: $C_{20}H_{17}N_7OS$
Molecular Weight: 403

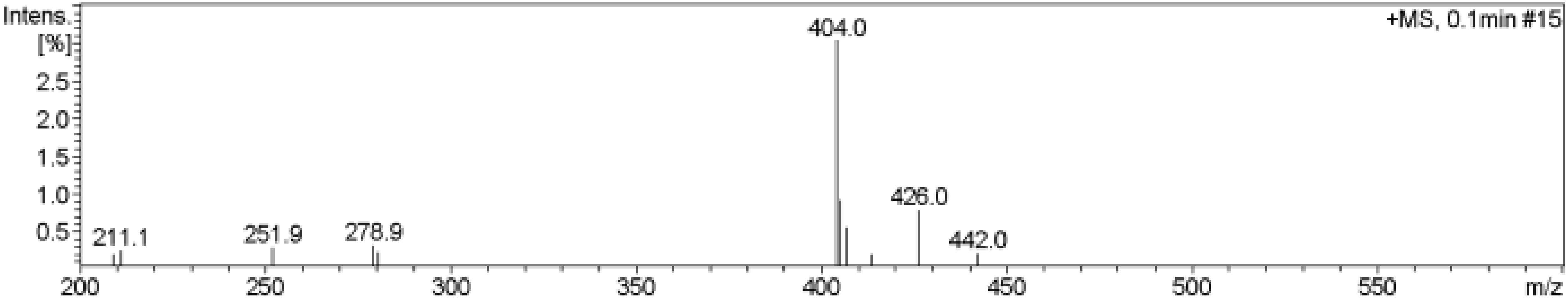

HRMS spectra of compound **14a**

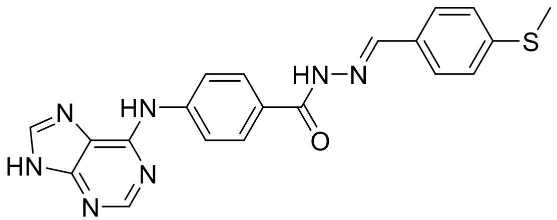

Chemical Formula: $C_{20}H_{17}N_7OS$
Molecular Weight: 403

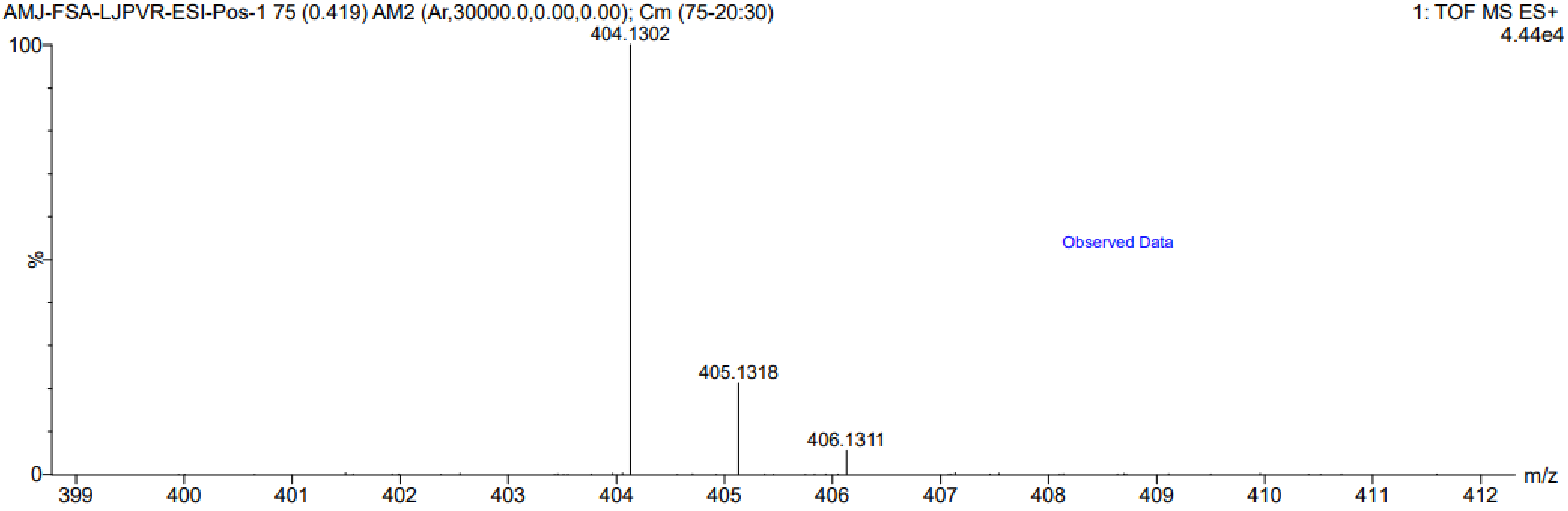

^{1}H-NMR spectra of compound **15a**

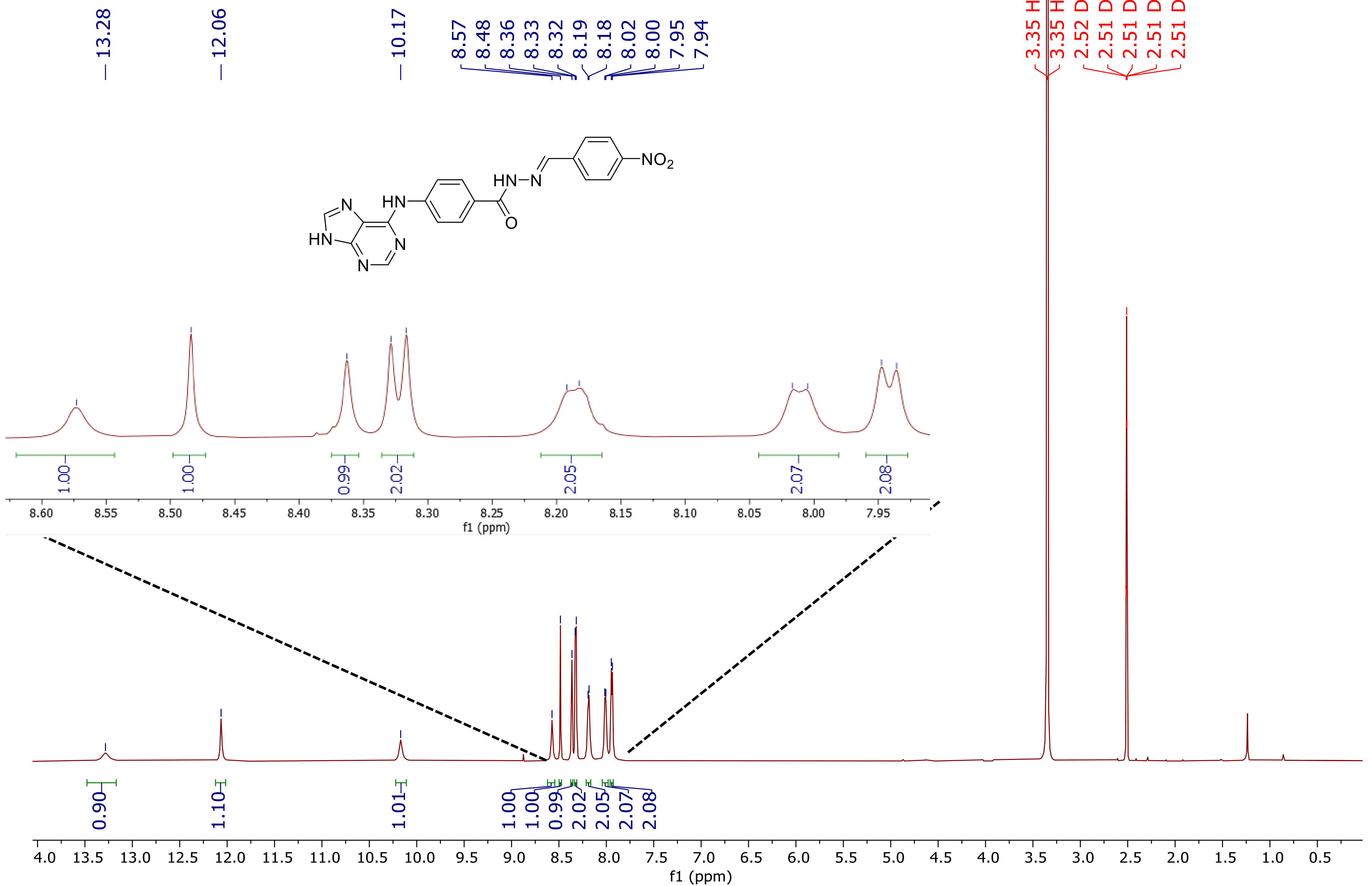

^{13}C-NMR spectra of compound **15a**

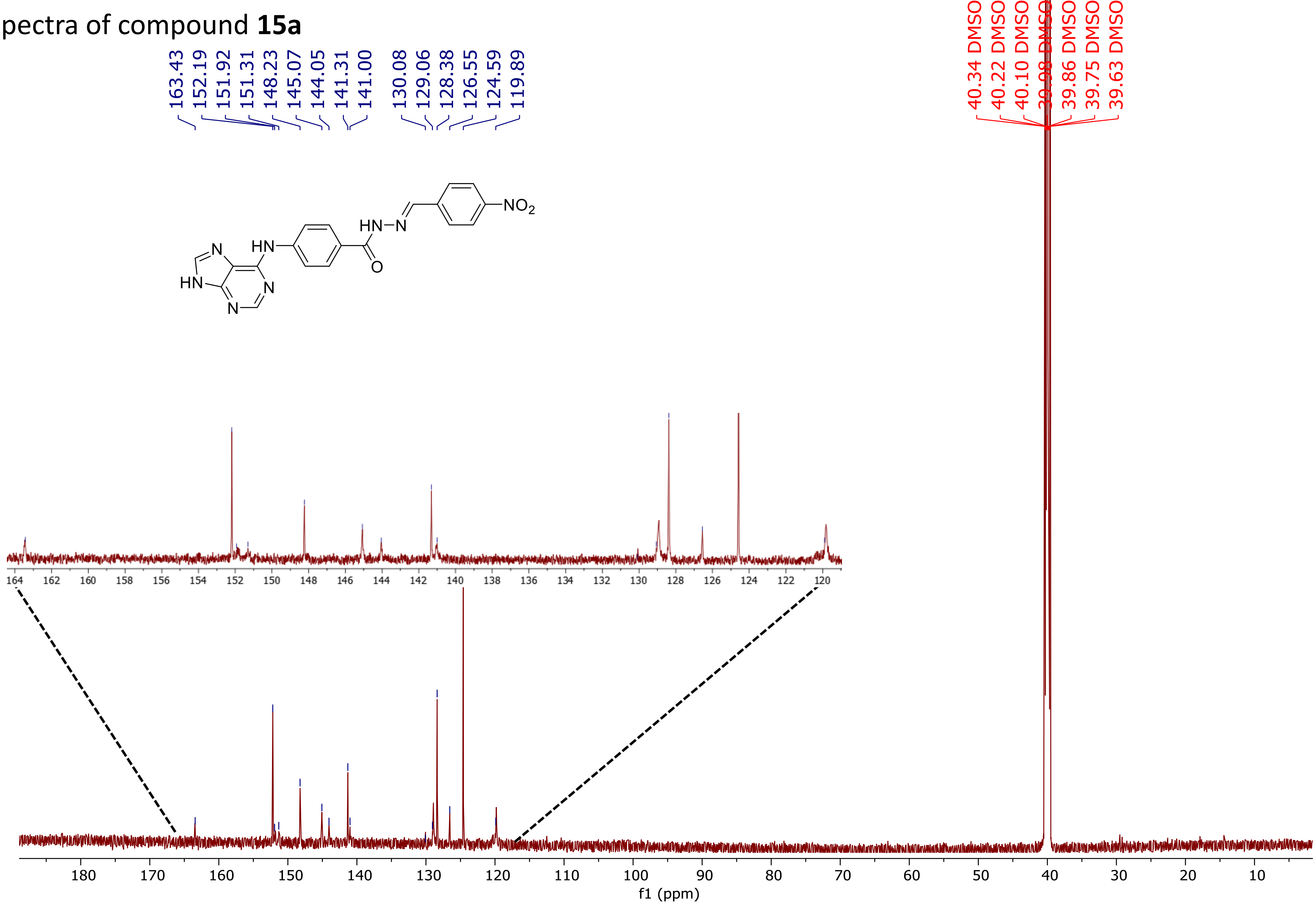

Mass spectra of compound **15a**

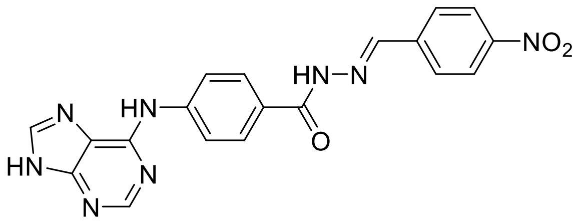

Chemical Formula: $C_{19}H_{14}N_8O_3$
Molecular Weight: 402

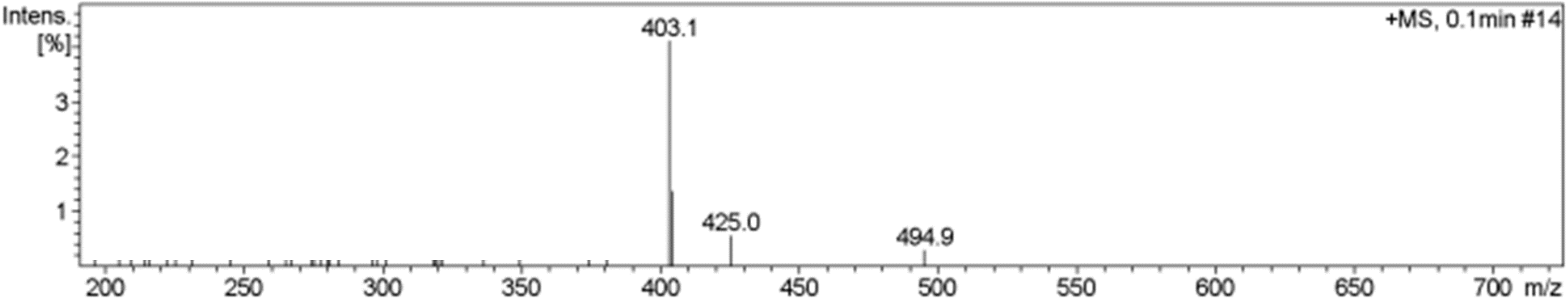

HRMS spectra of compound **15a**

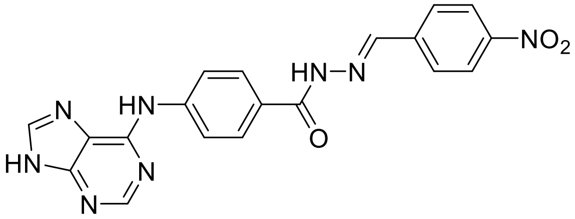

Chemical Formula: $C_{19}H_{14}N_8O_3$
Exact Mass: 402.1189

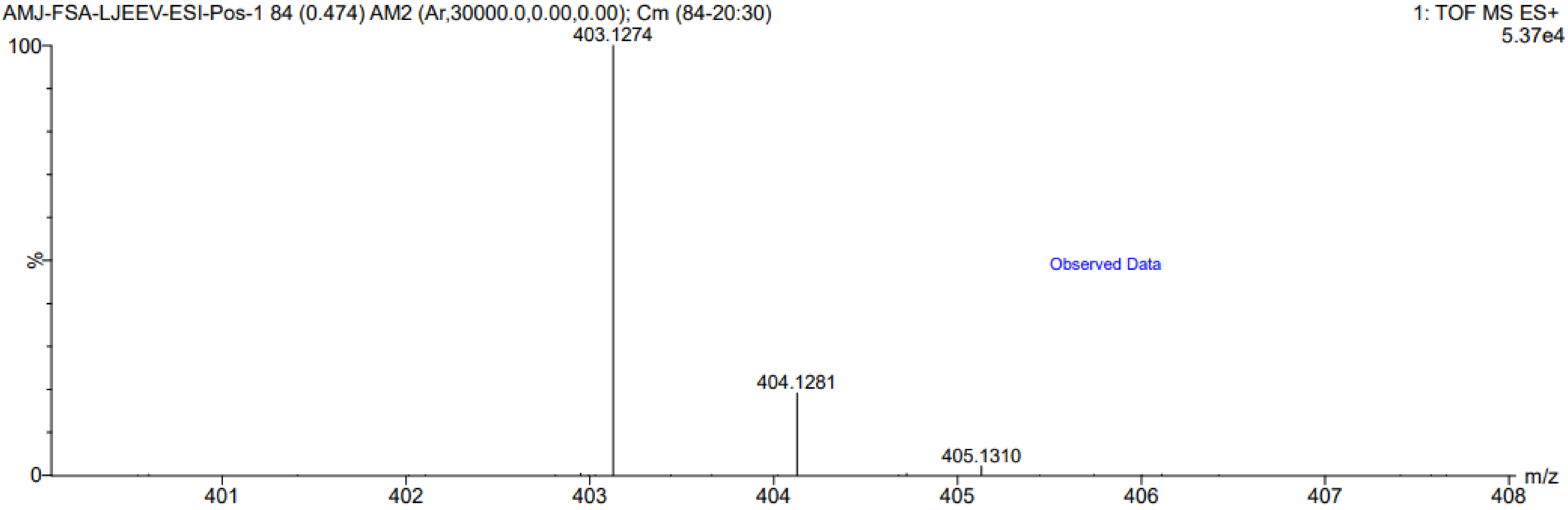

^{1}H-NMR spectra of compound **16a**

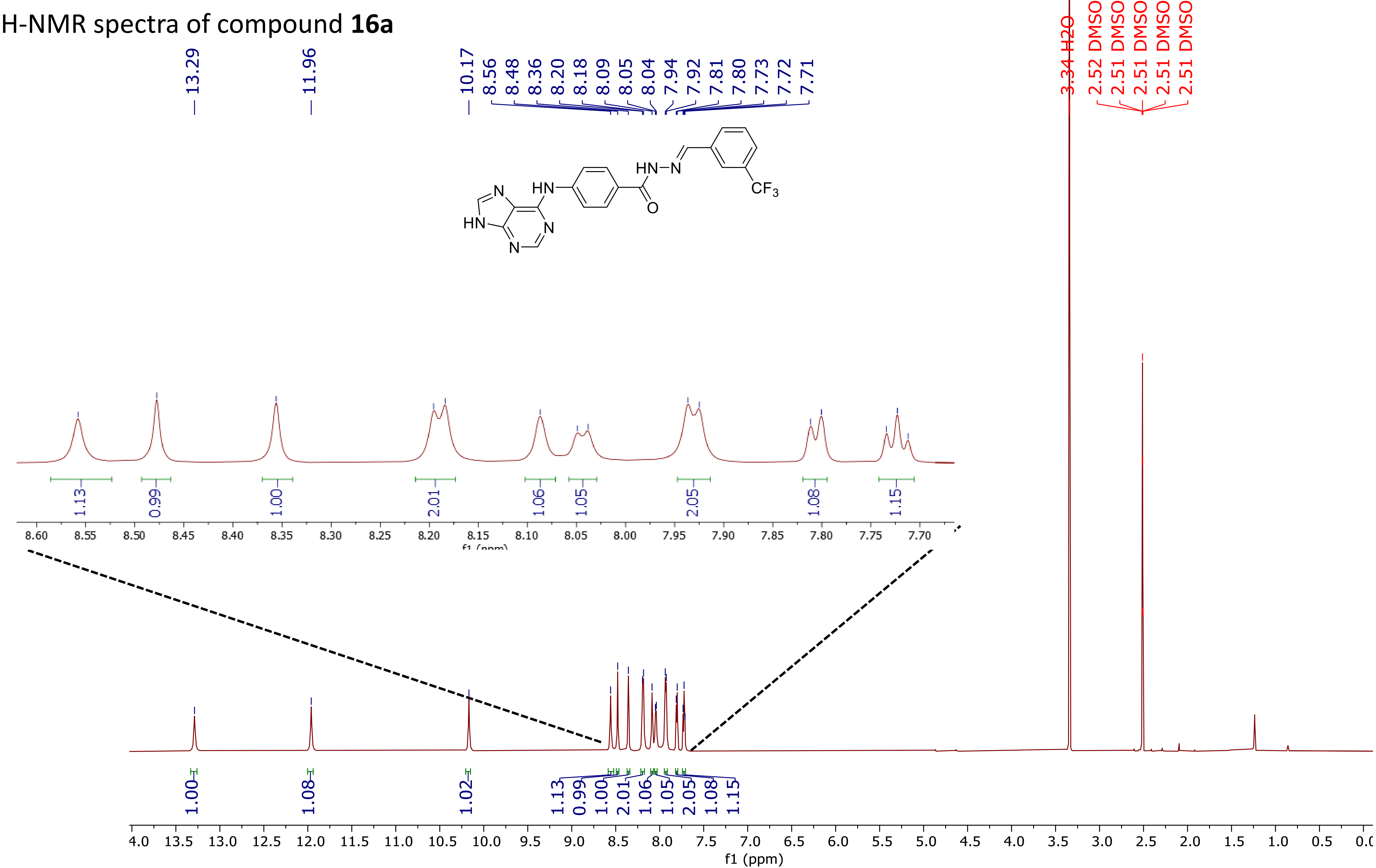

^{13}C-NMR spectra of compound **16a**

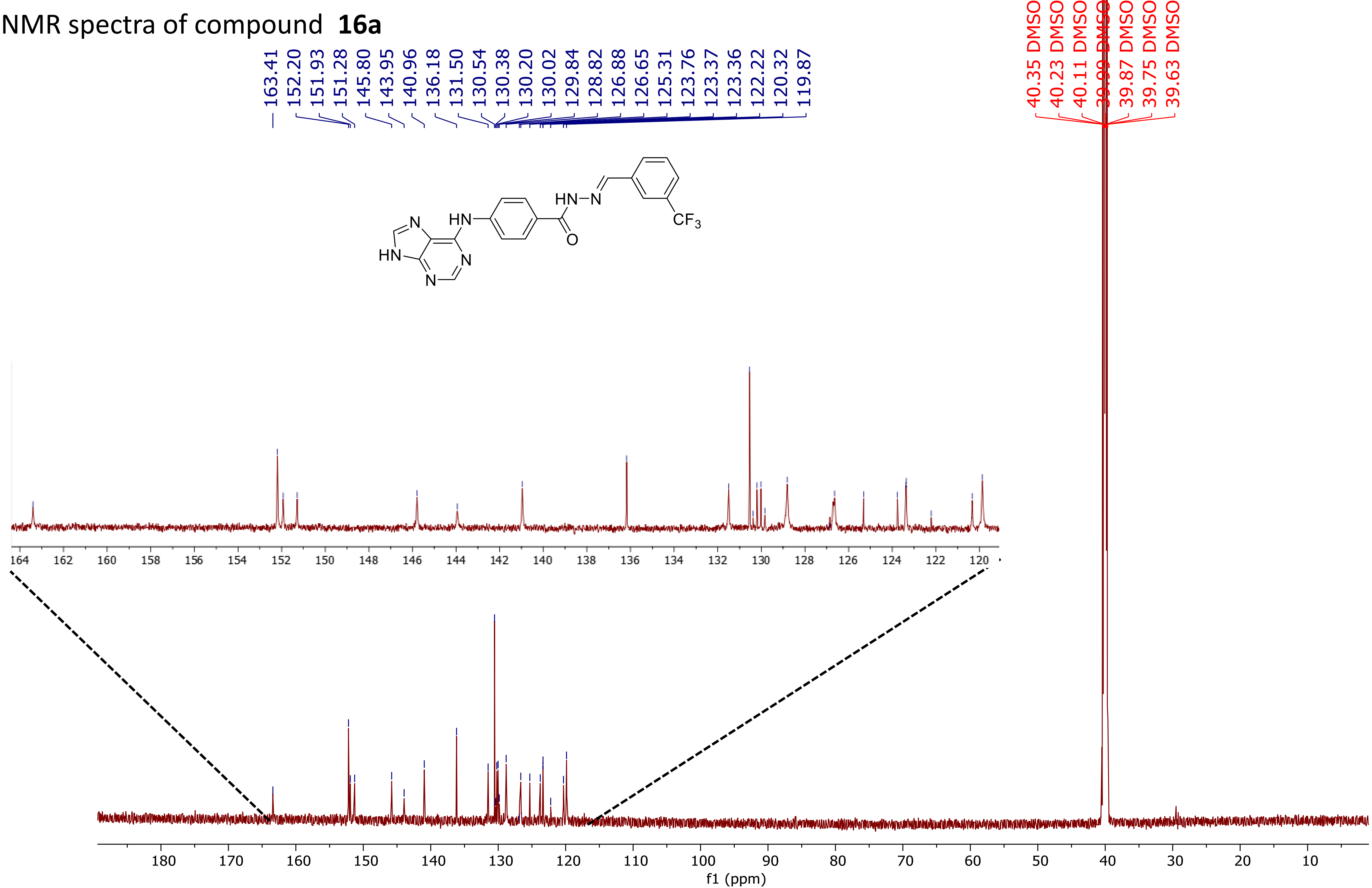

Mass spectra of compound **16a**

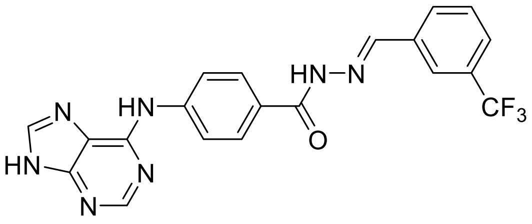

Chemical Formula: $C_{20}H_{14}F_3N_7O$
Molecular Weight: 425

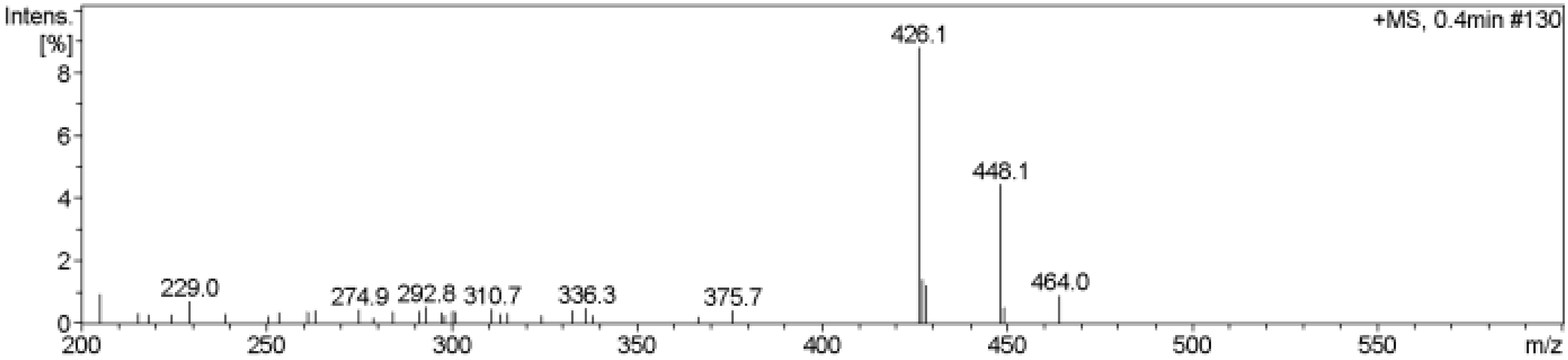

HRMS spectra of compound **16a**

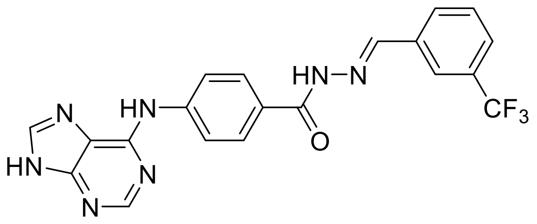

Chemical Formula: $C_{20}H_{14}F_3N_7O$
Molecular Weight: 425.3752

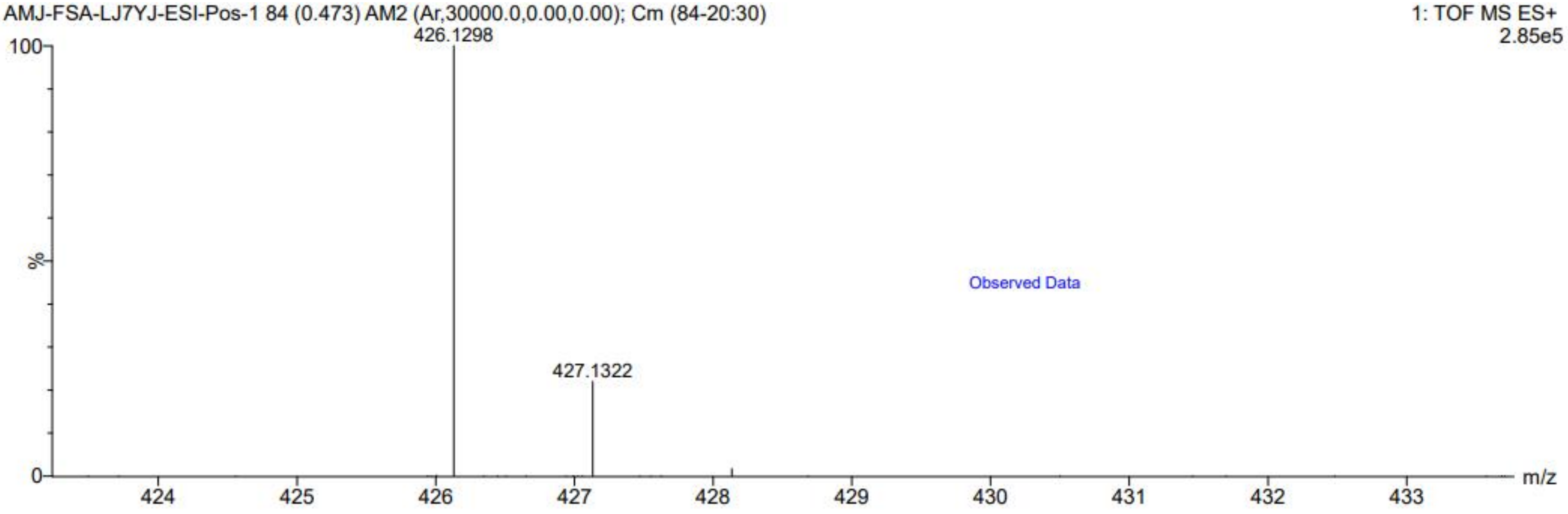

^{1}H-NMR spectra of compound **17a**

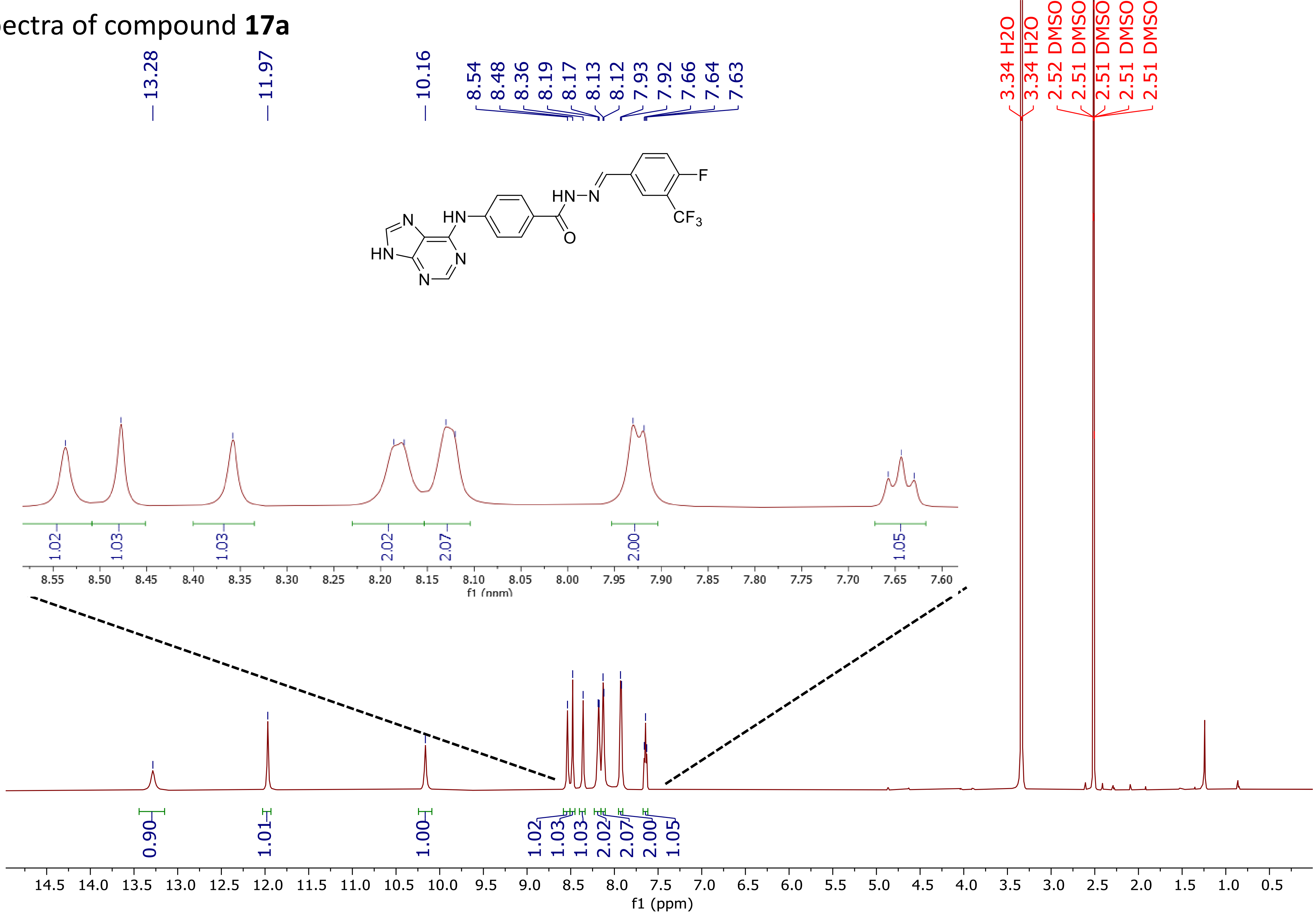

^{13}C-NMR spectra of compound **17a**

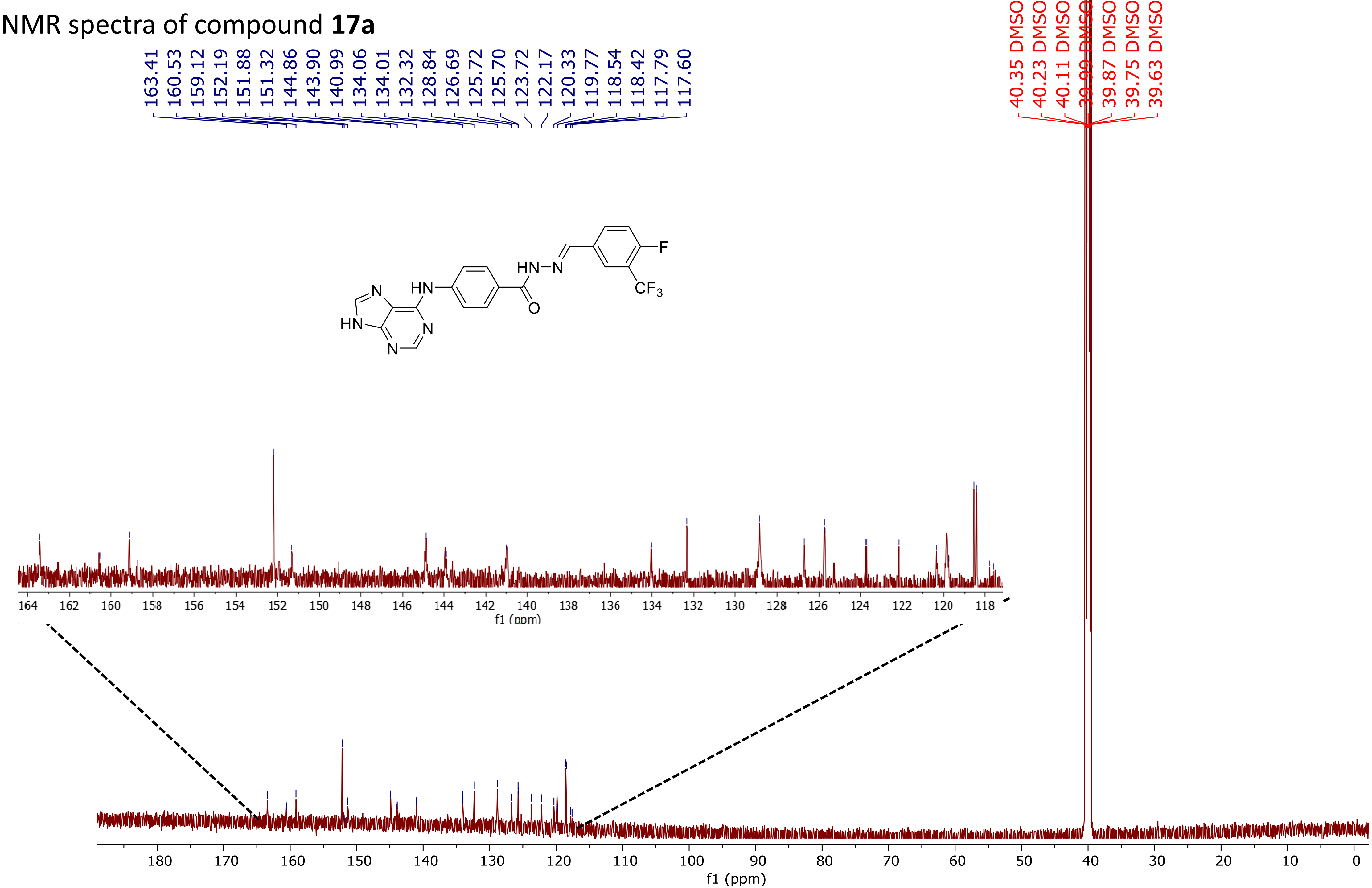

Mass spectra of compound **17a**

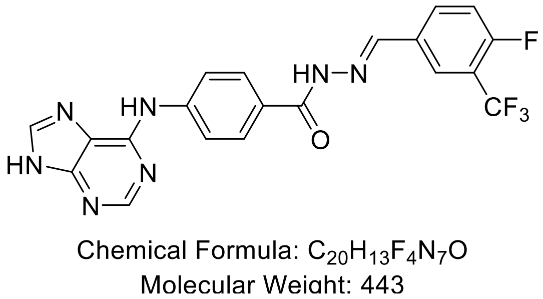

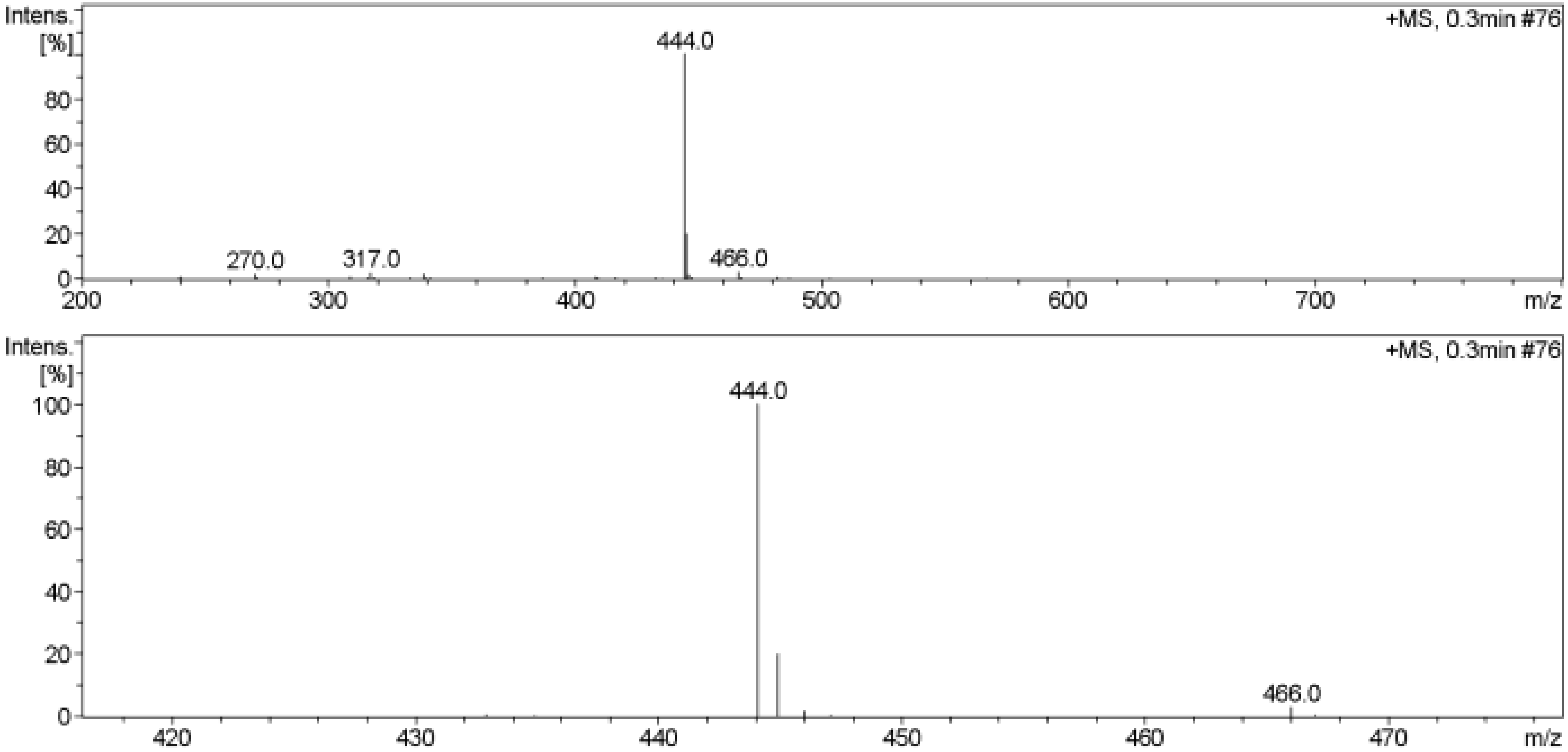

Mass spectra of compound **17a**

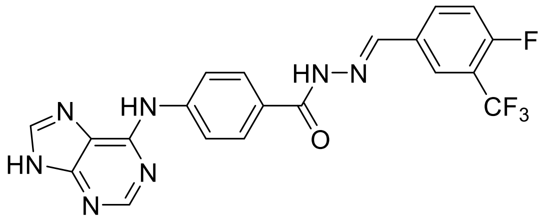

Chemical Formula: $C_{20}H_{13}F_4N_7O$
Molecular Weight: 443.3656

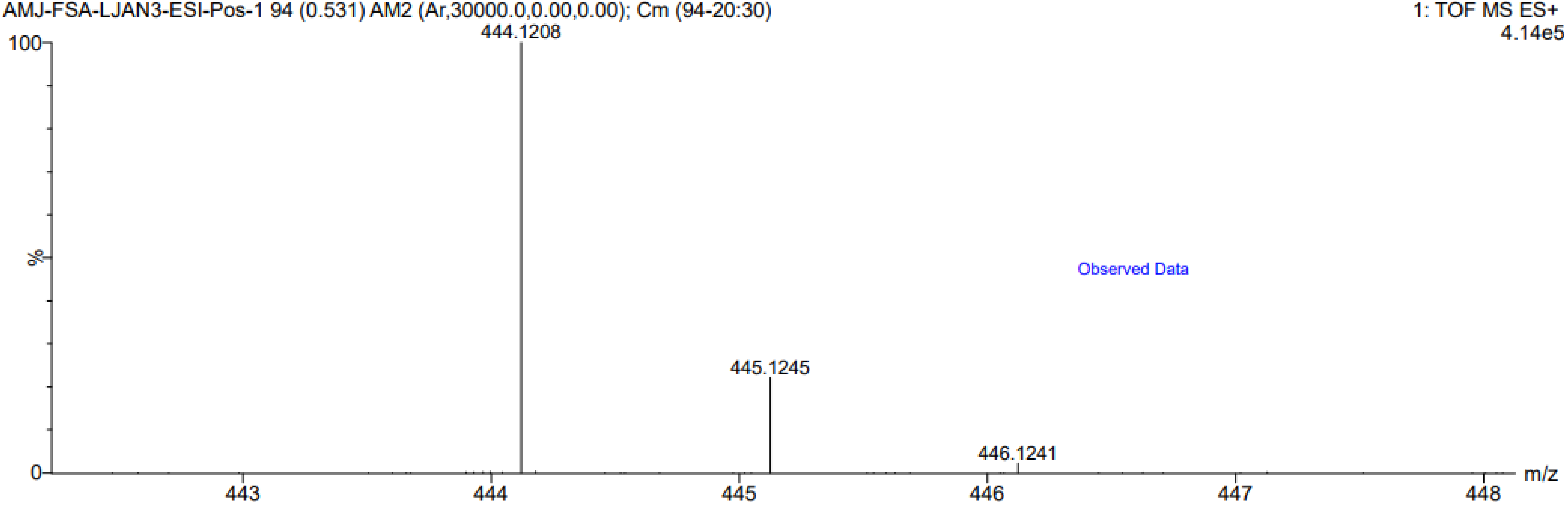

¹H-NMR spectra of compound **18a**

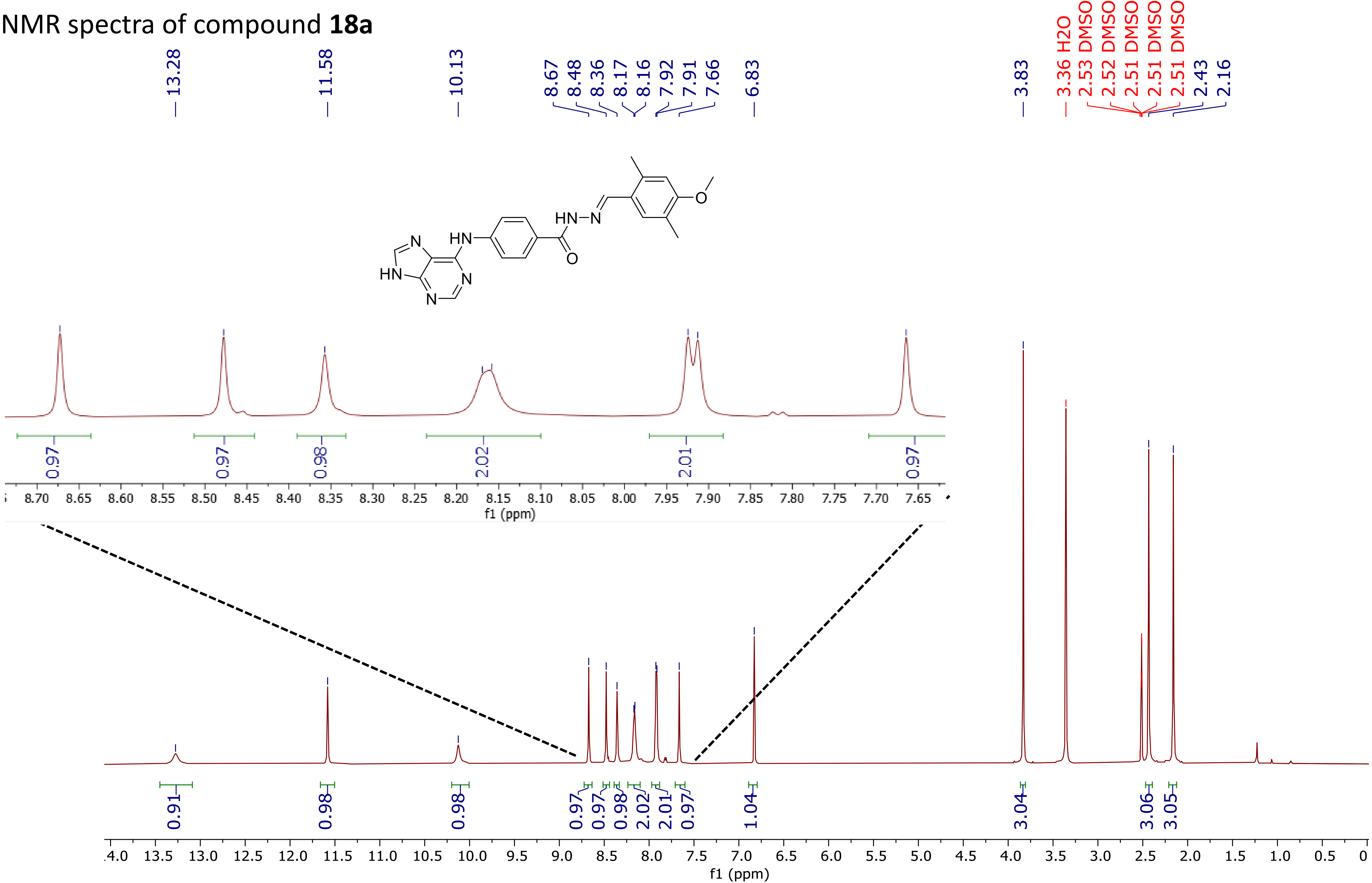

^{13}C-NMR spectra of compound **18a**

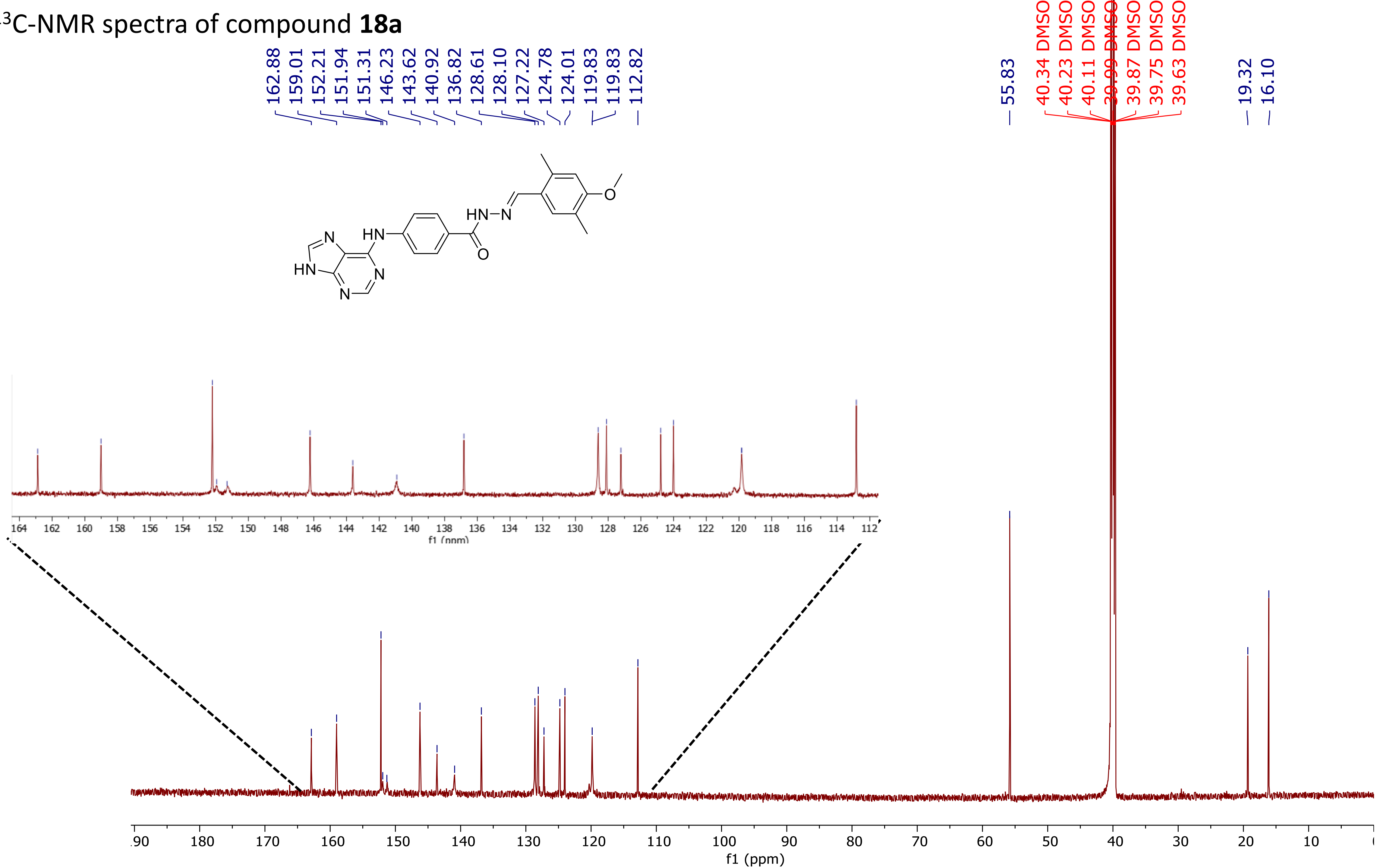

Mass spectra of compound **18a**

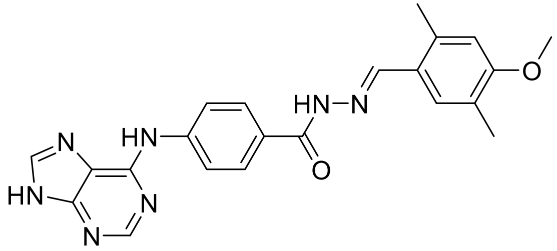

Chemical Formula: $C_{22}H_{21}N_7O_2$
Molecular Weight: 415

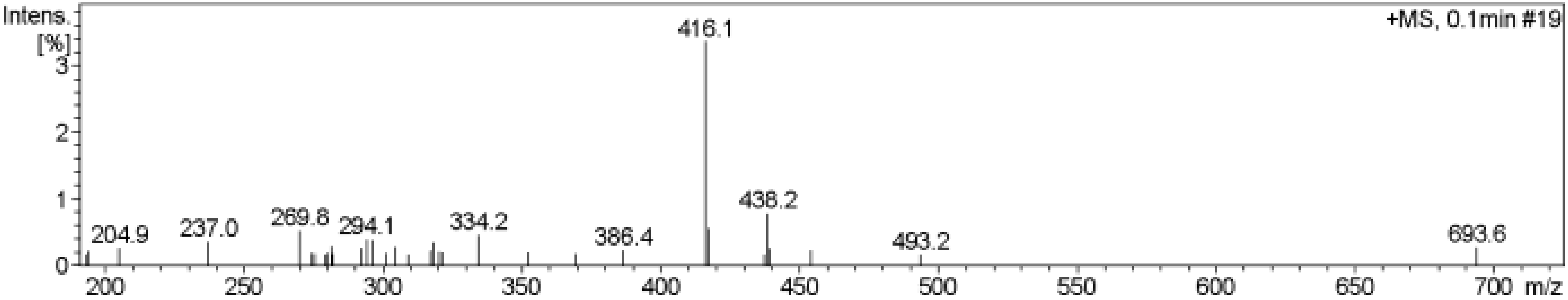

HRMS spectra of compound **18a**

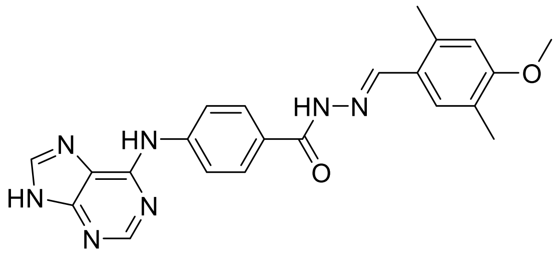

Chemical Formula: $C_{22}H_{21}N_7O_2$
Molecular Weight: 415

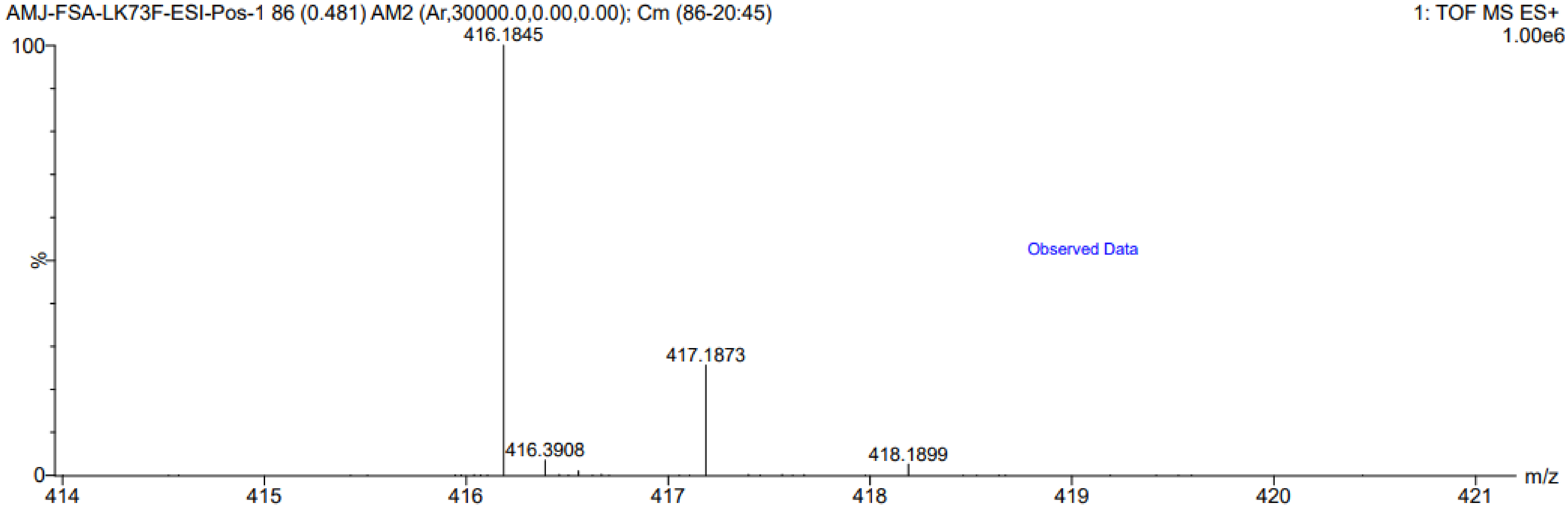

^{1}H-NMR spectra of compound **19a**

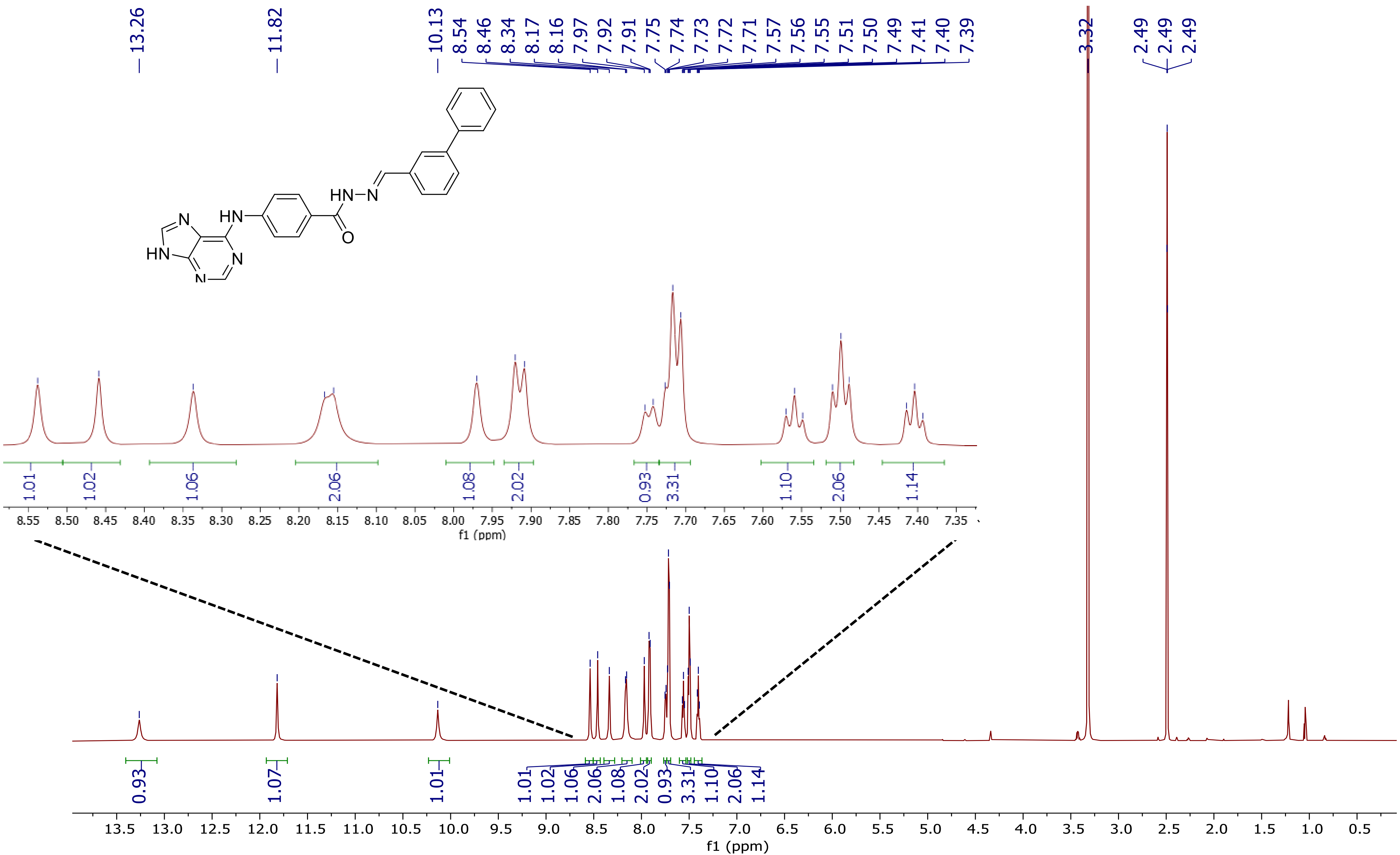

^{13}C-NMR spectra of compound **19a**

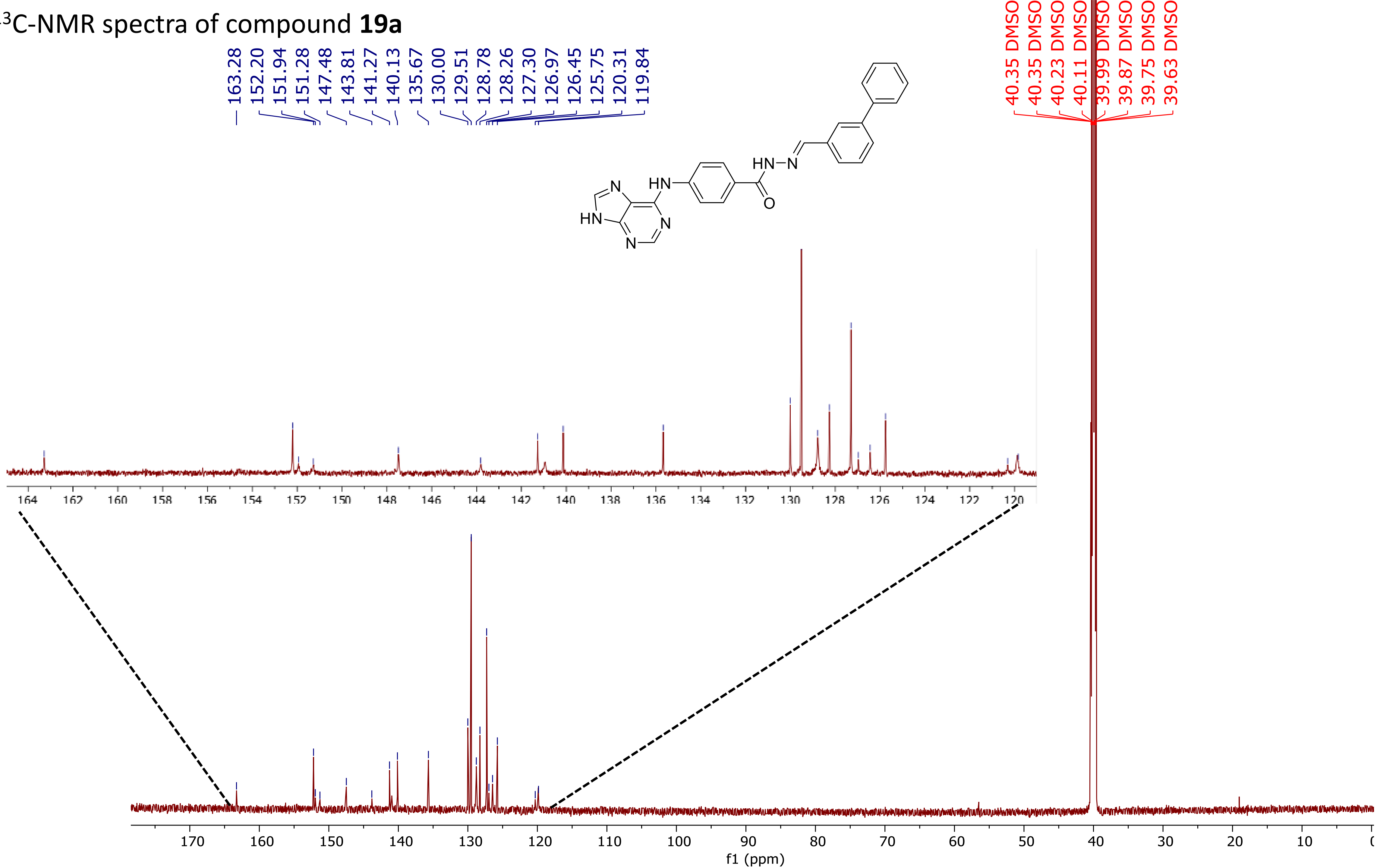

Mass spectra of compound **19a**

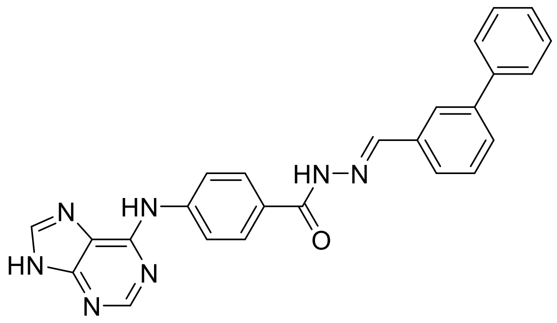

Chemical Formula: $C_{25}H_{19}N_7O$
Molecular Weight: 433

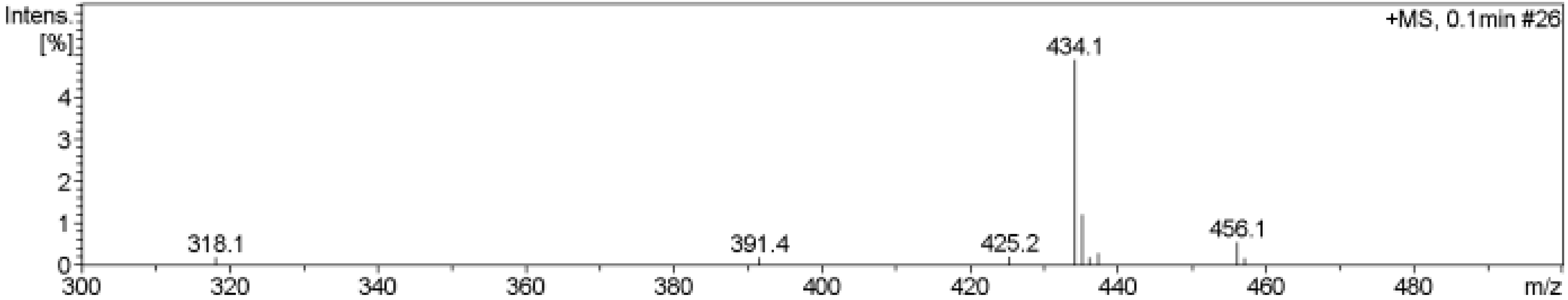

HRMS spectra of compound **19a**

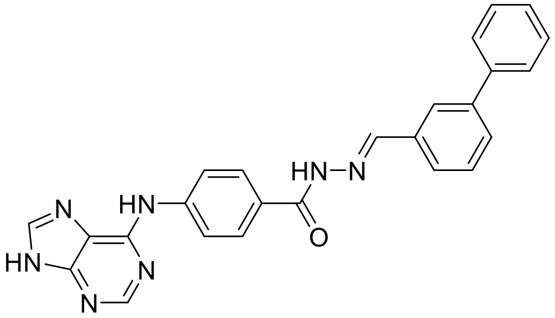

Chemical Formula: $C_{25}H_{19}N_7O$
Molecular Weight: 433

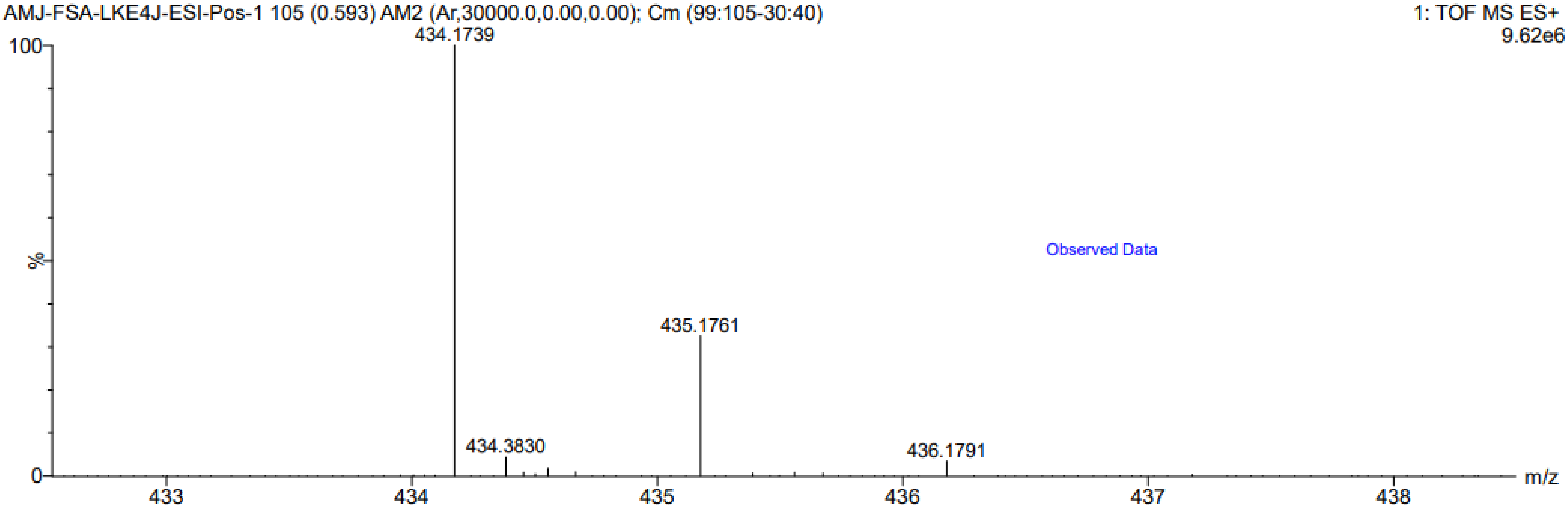

^{1}H-NMR spectra of compound **20a**

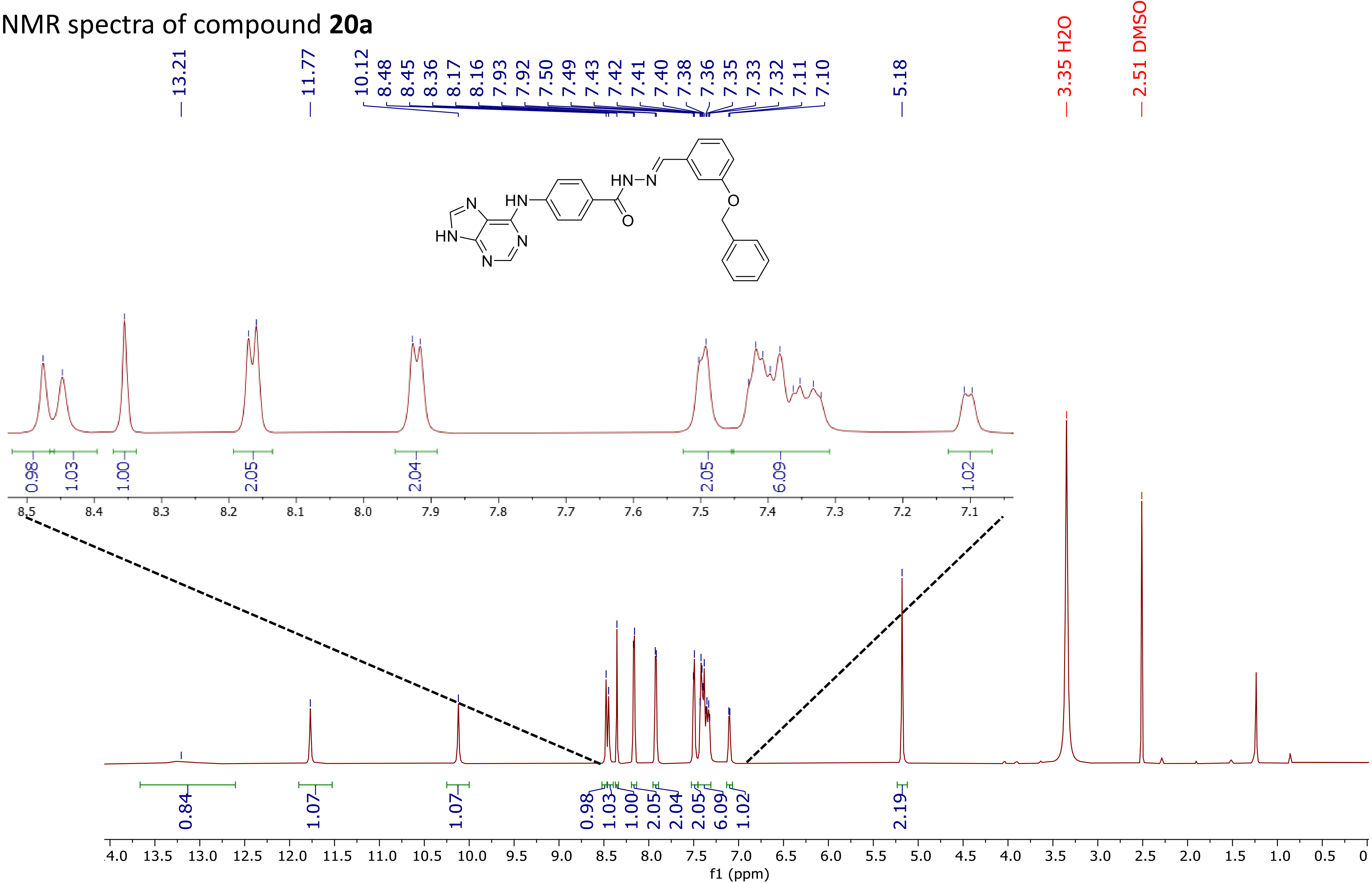

^{13}C-NMR spectra of compound **20a**

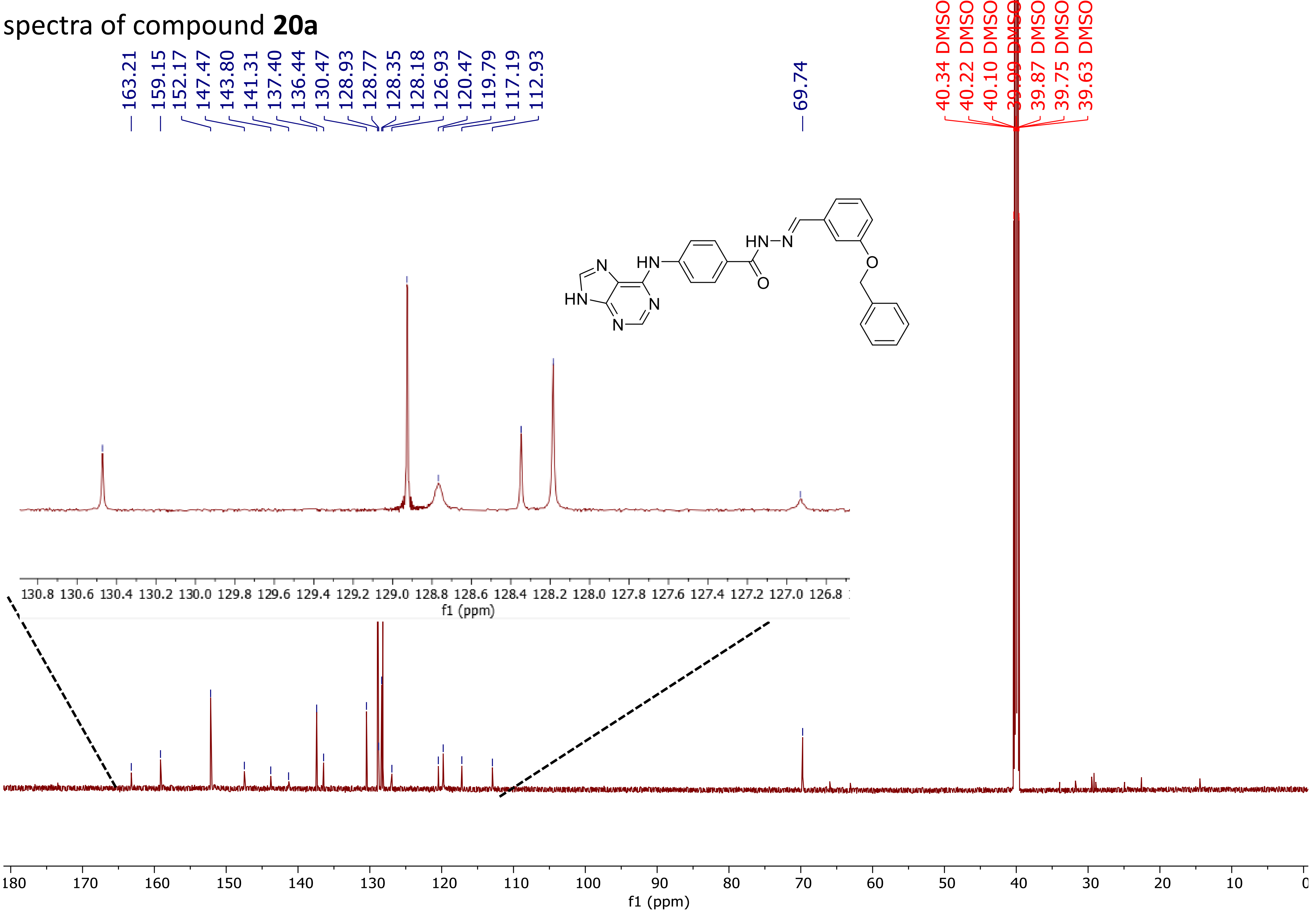

Mass spectra of compound **20a**

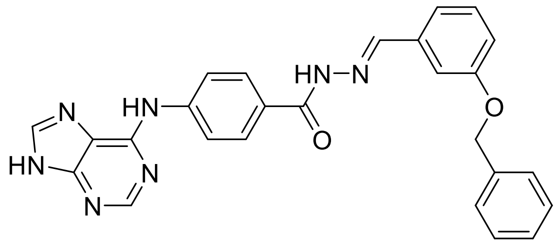

Chemical Formula: $C_{26}H_{21}N_7O_2$
Molecular Weight: 464

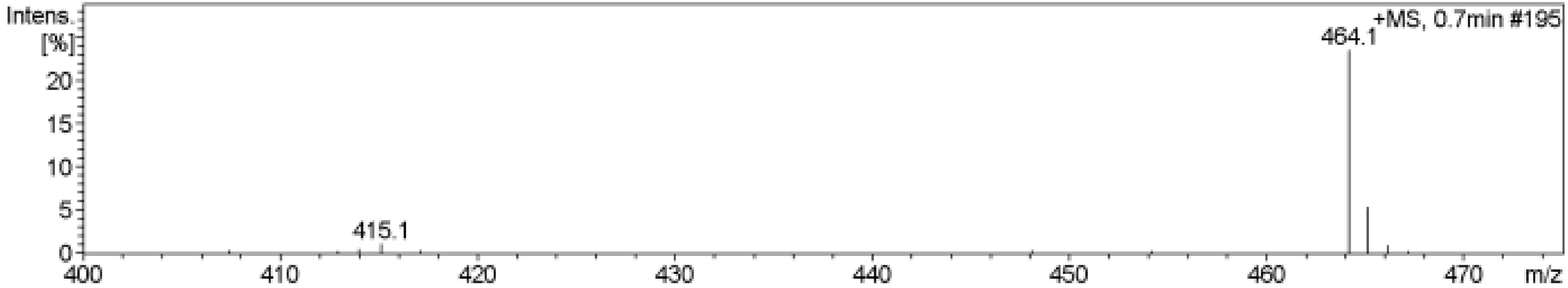

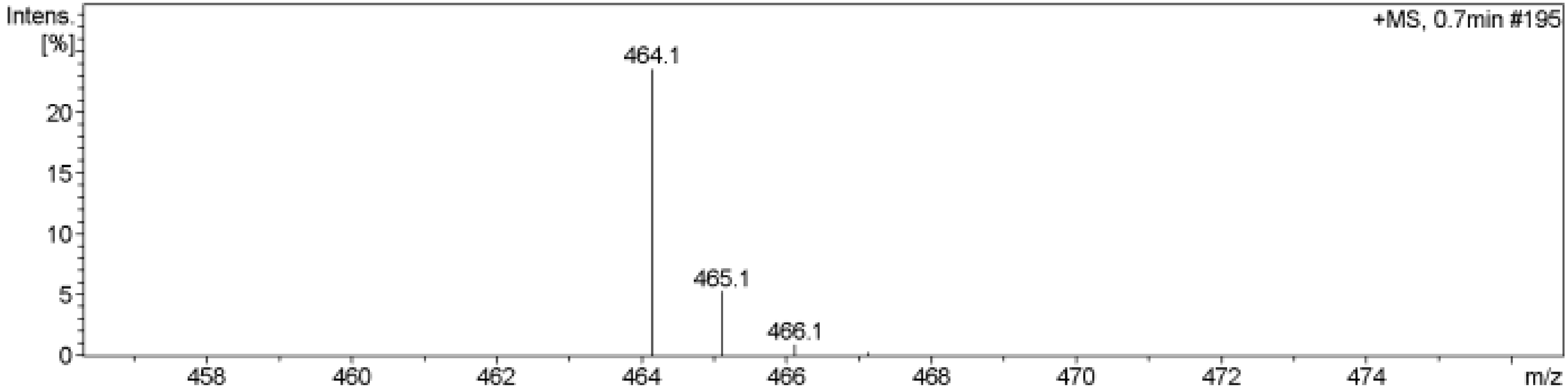

HRMS spectra of compound **20a**

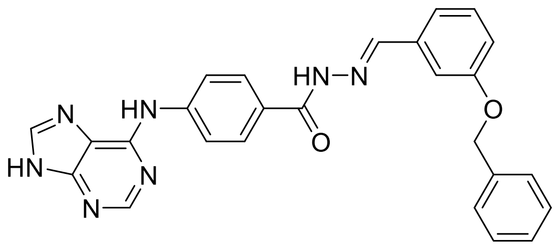

Chemical Formula: $C_{26}H_{21}N_7O_2$
Molecular Weight: 464

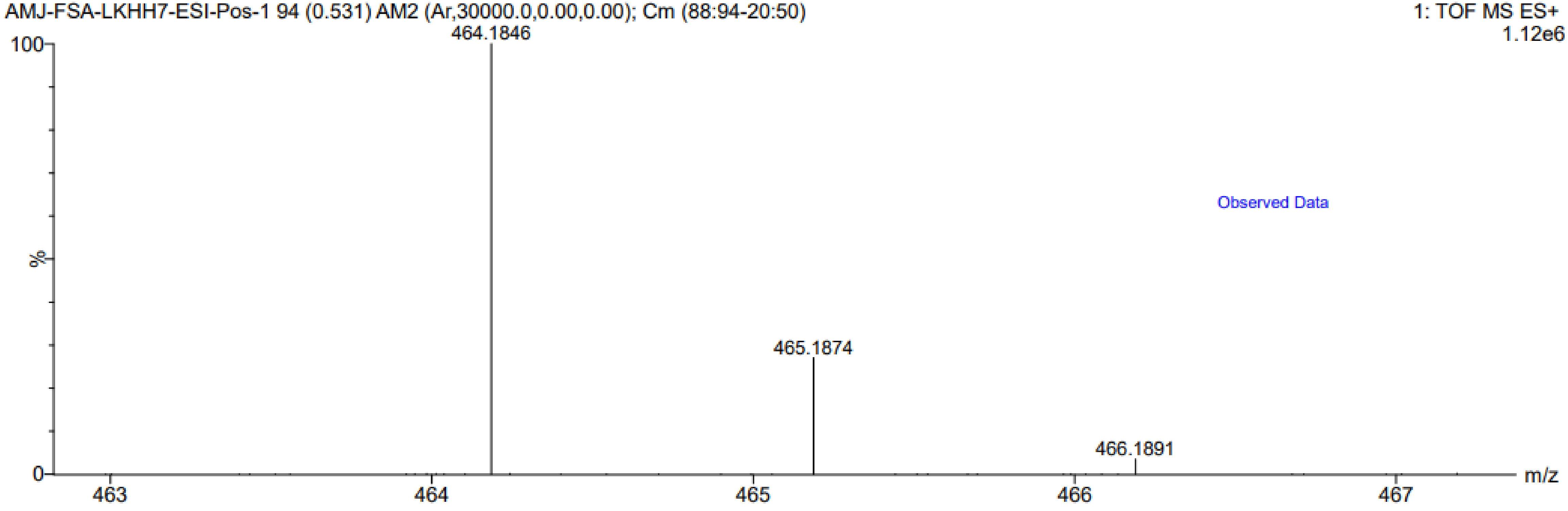

^{1}H-NMR spectra of compound **23a**

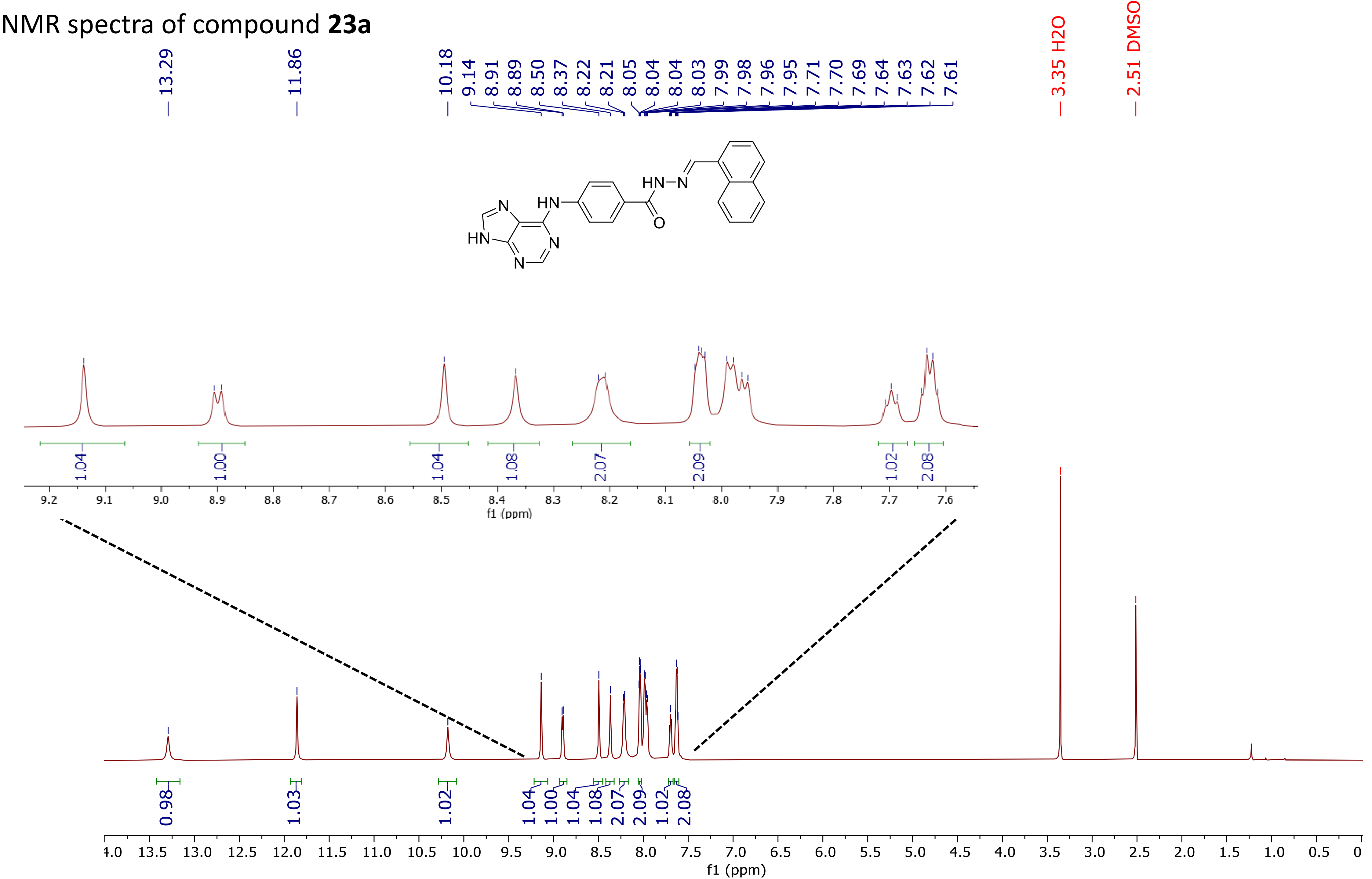

^{13}C-NMR spectra of compound **23a**

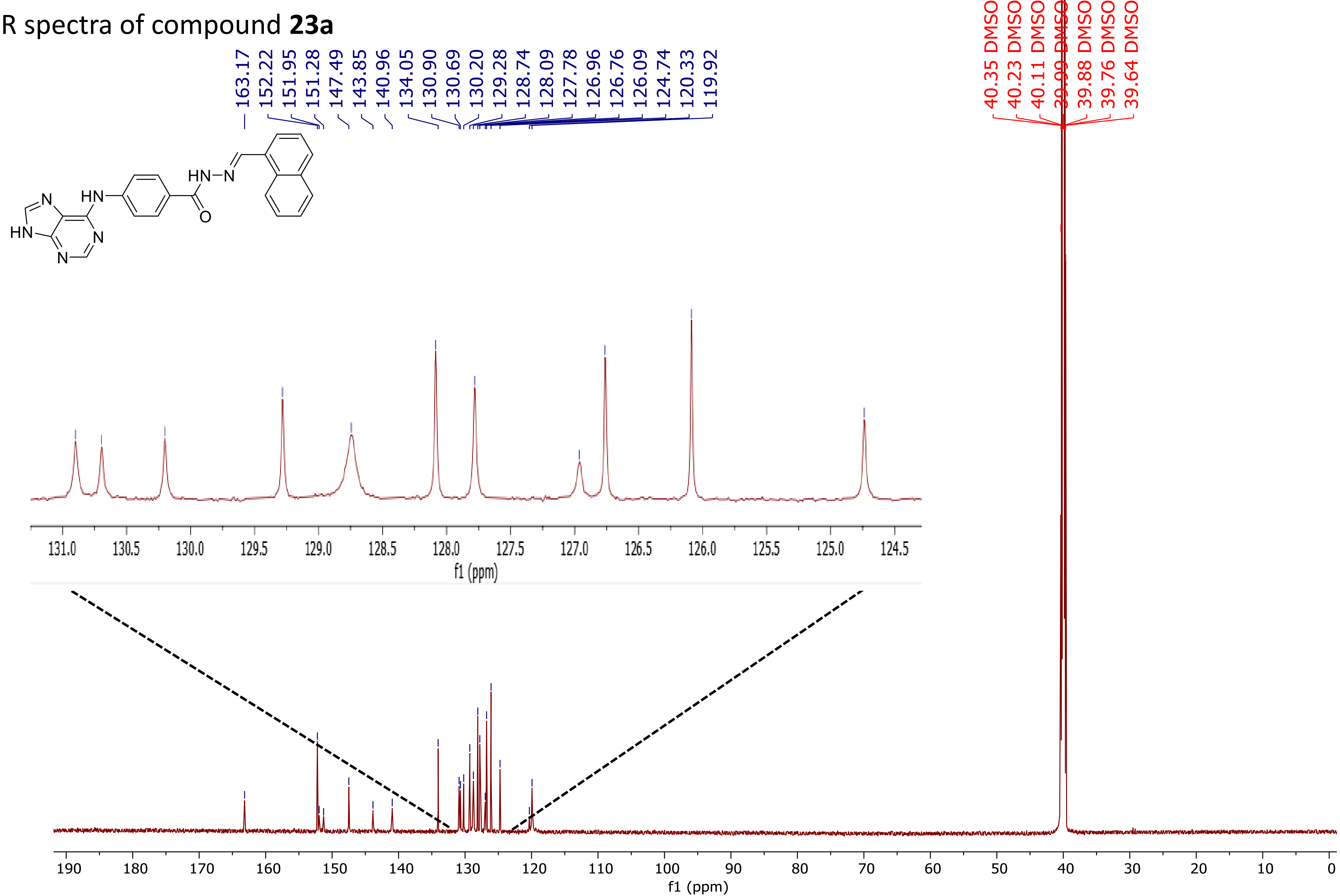

Mass spectra of compound **23a**

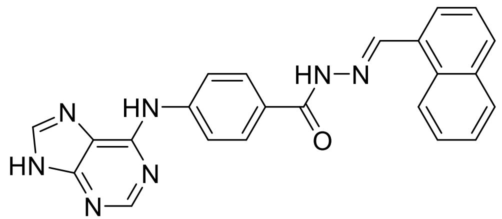

Chemical Formula: $C_{23}H_{17}N_7O$
Molecular Weight: 407

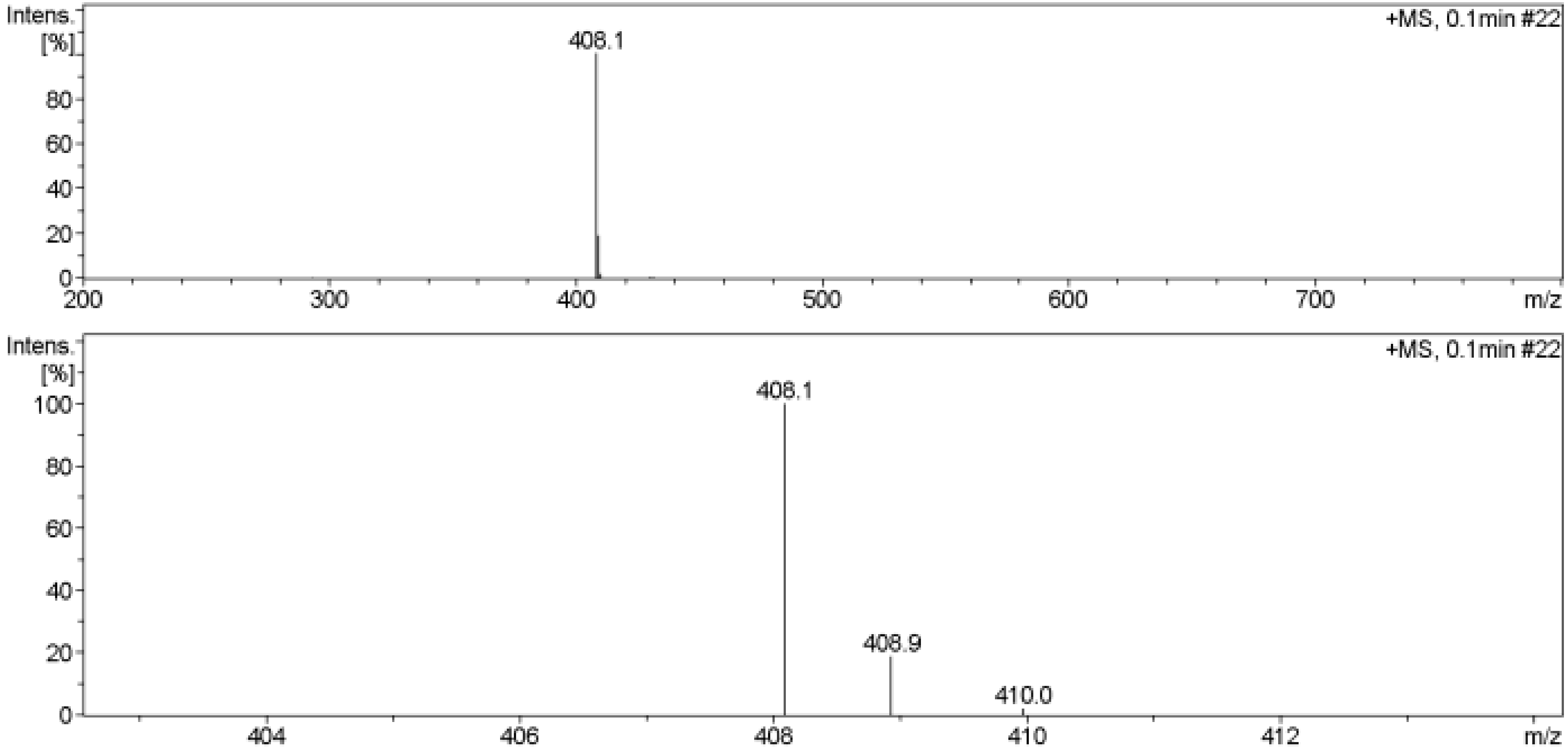

HRMS spectra of compound **23a**

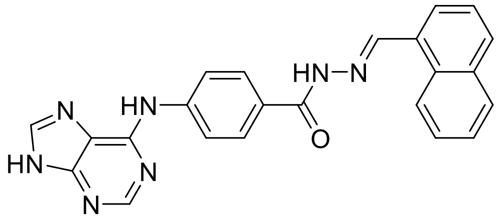

Chemical Formula: $C_{23}H_{17}N_7O$
Molecular Weight: 407

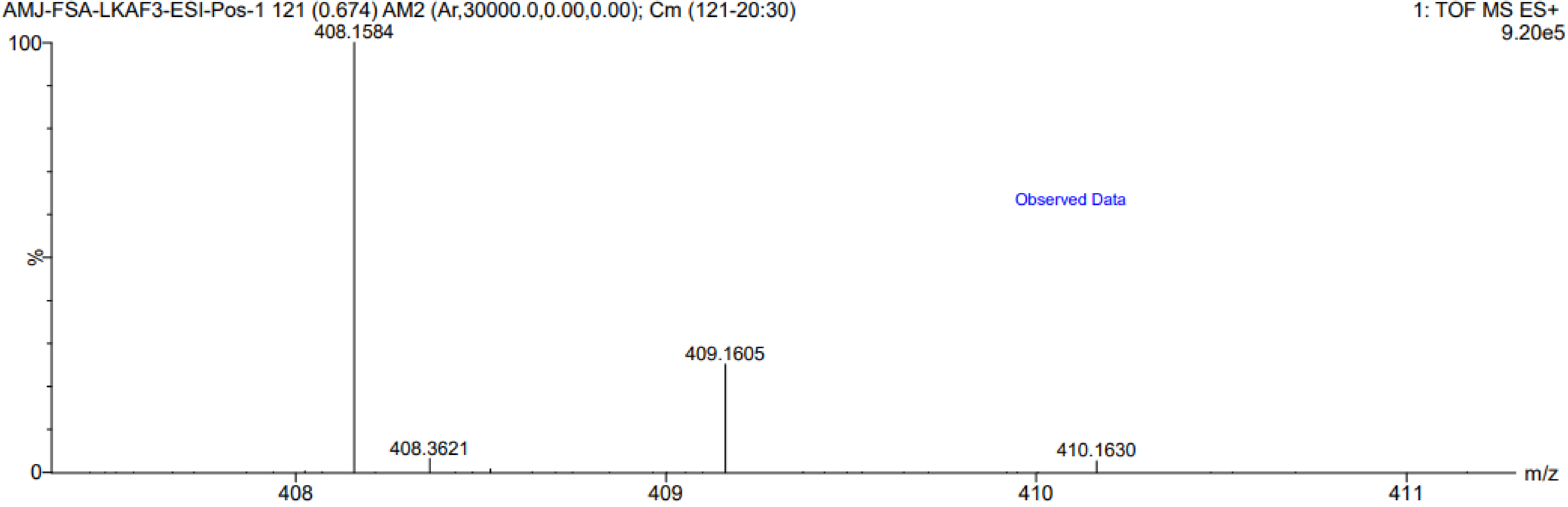

^{1}H-NMR spectra of compound **6b**

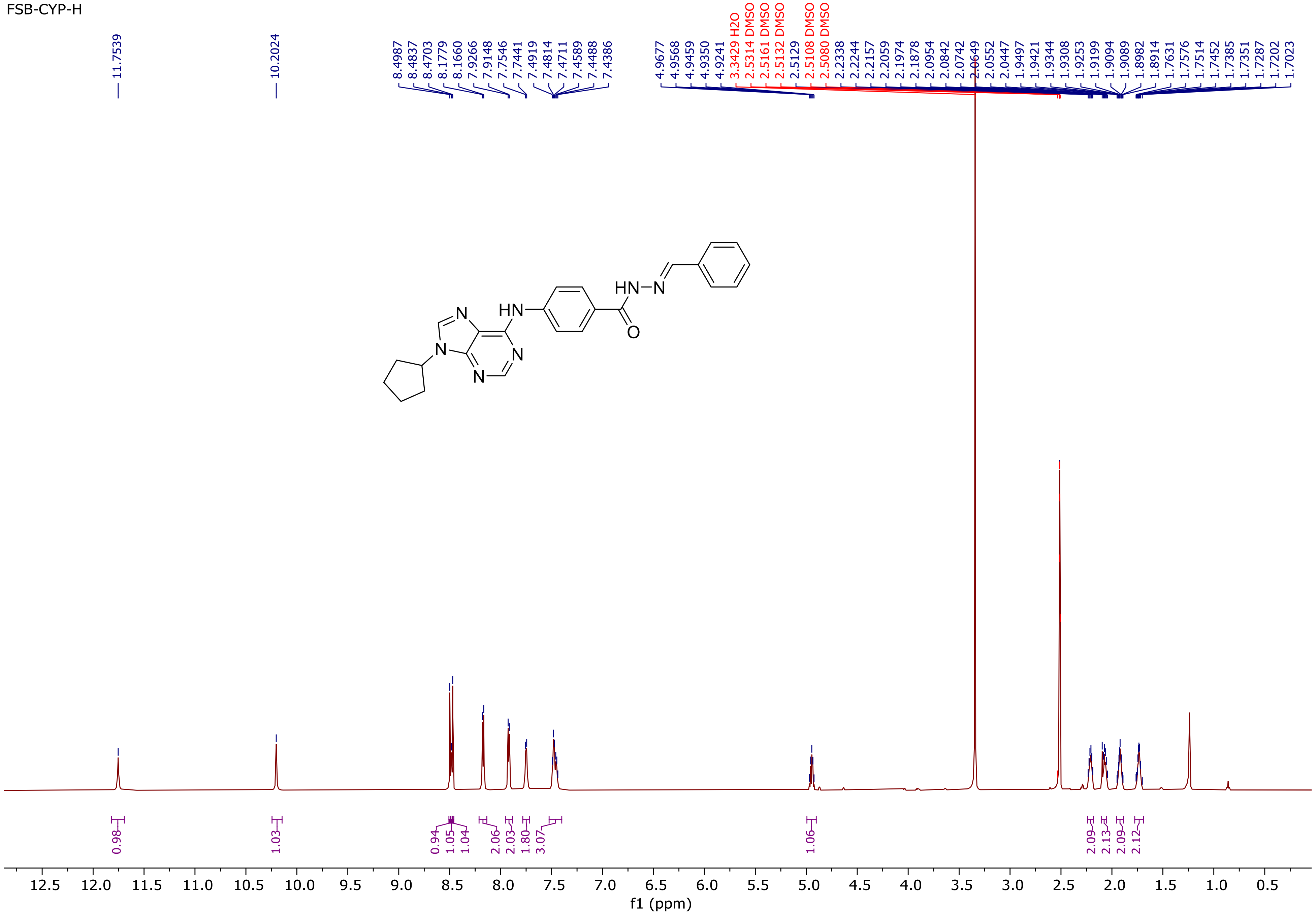

^{13}C-NMR spectra of compound **6b**

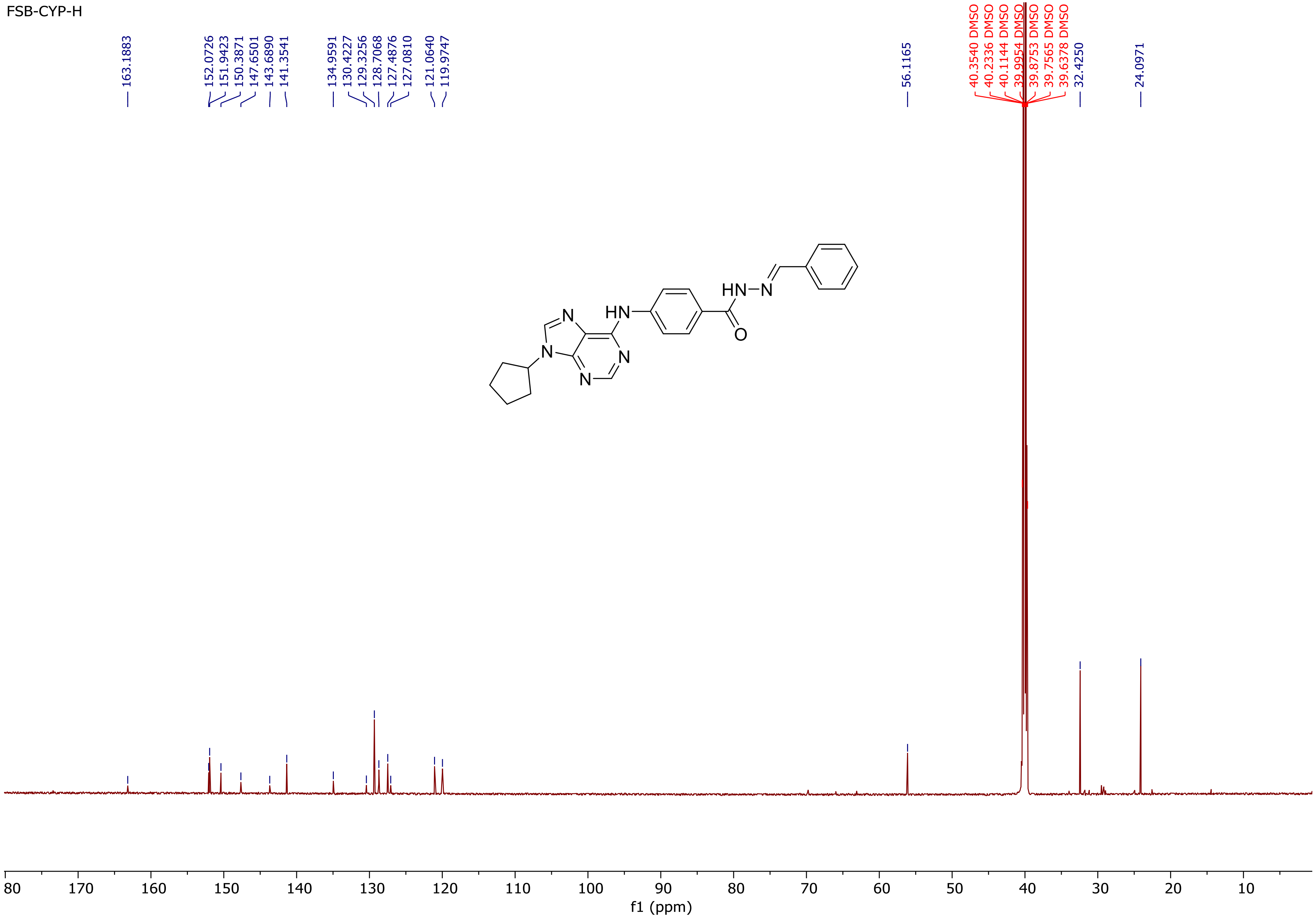

Mass spectra of compound **6b**

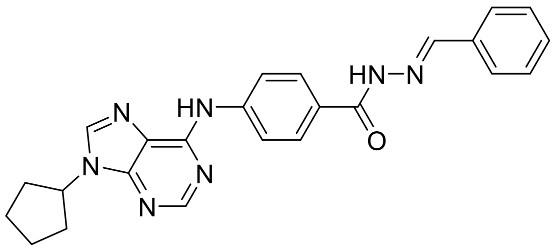

Chemical Formula: $C_{24}H_{23}N_7O$
Exact Mass: 425.1964

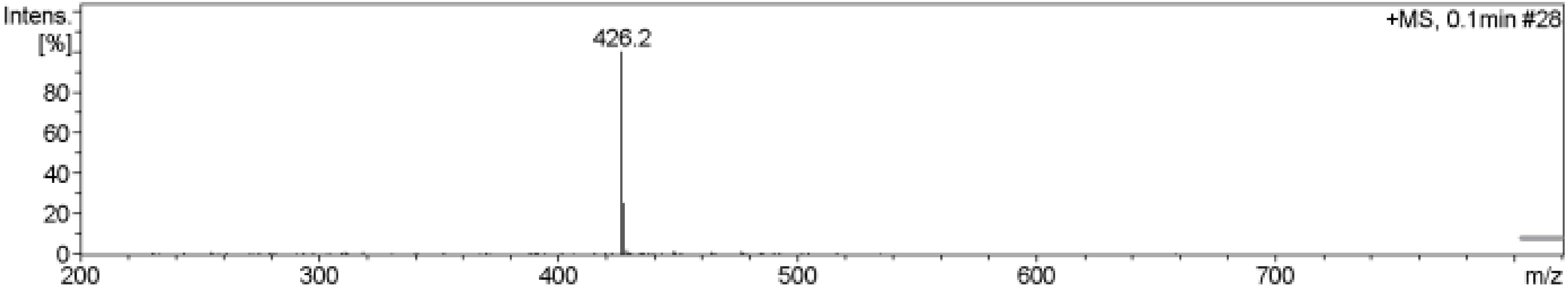

HRMS spectra of compound **6b**

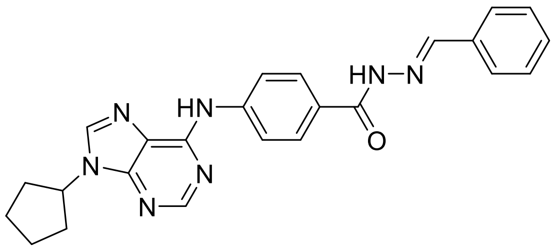

Chemical Formula: $C_{24}H_{23}N_7O$
Exact Mass: 425.1964

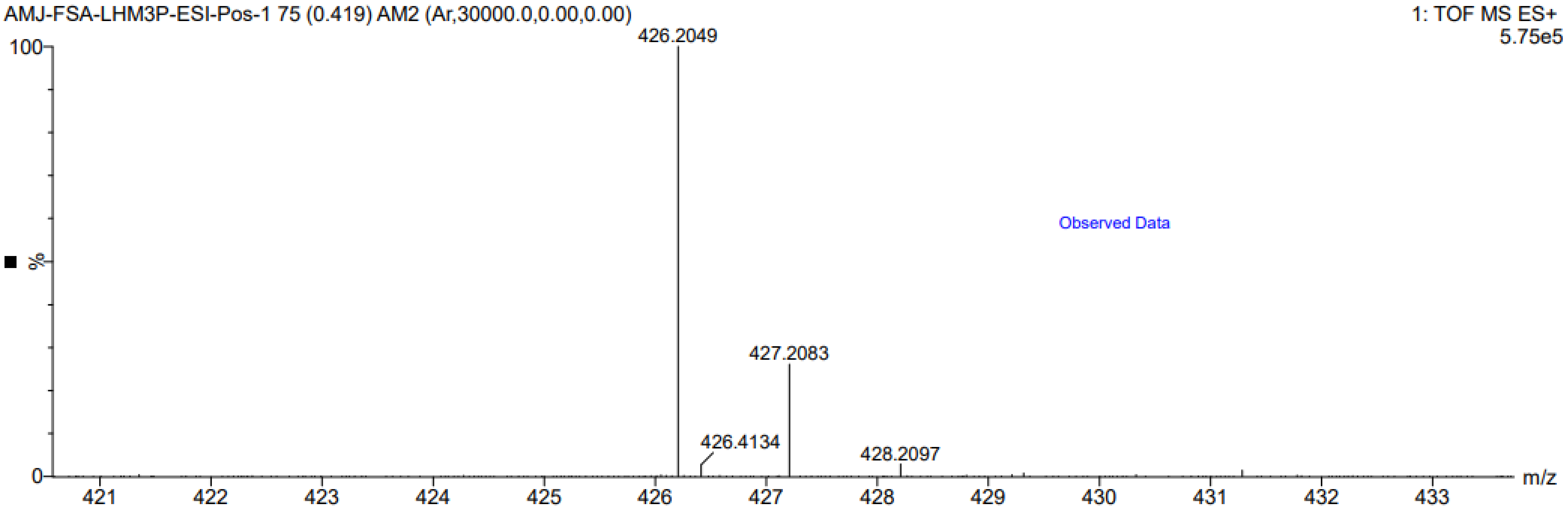

^{1}H-NMR spectra of compound **11b**

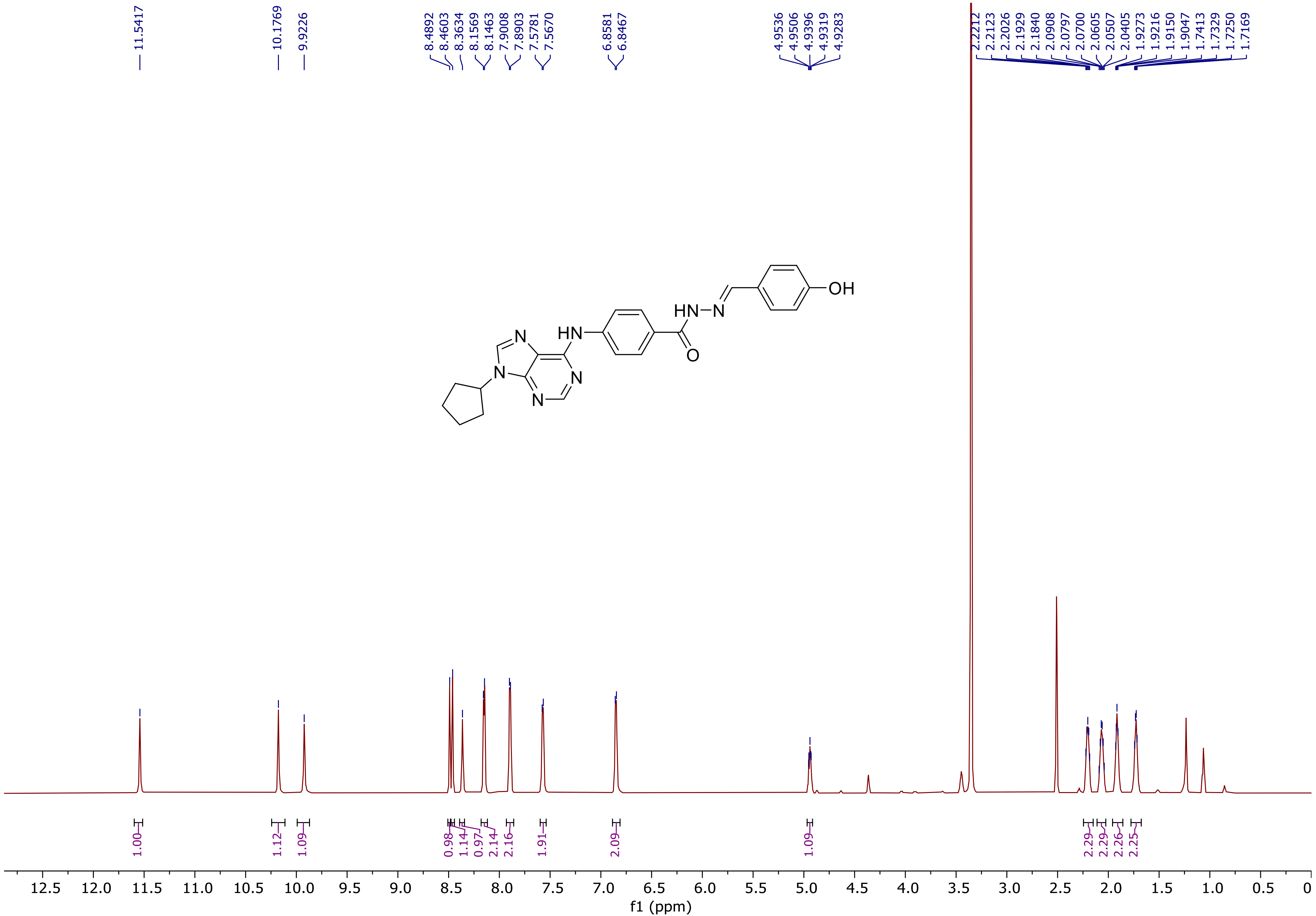

^{13}C-NMR spectra of compound **11b**

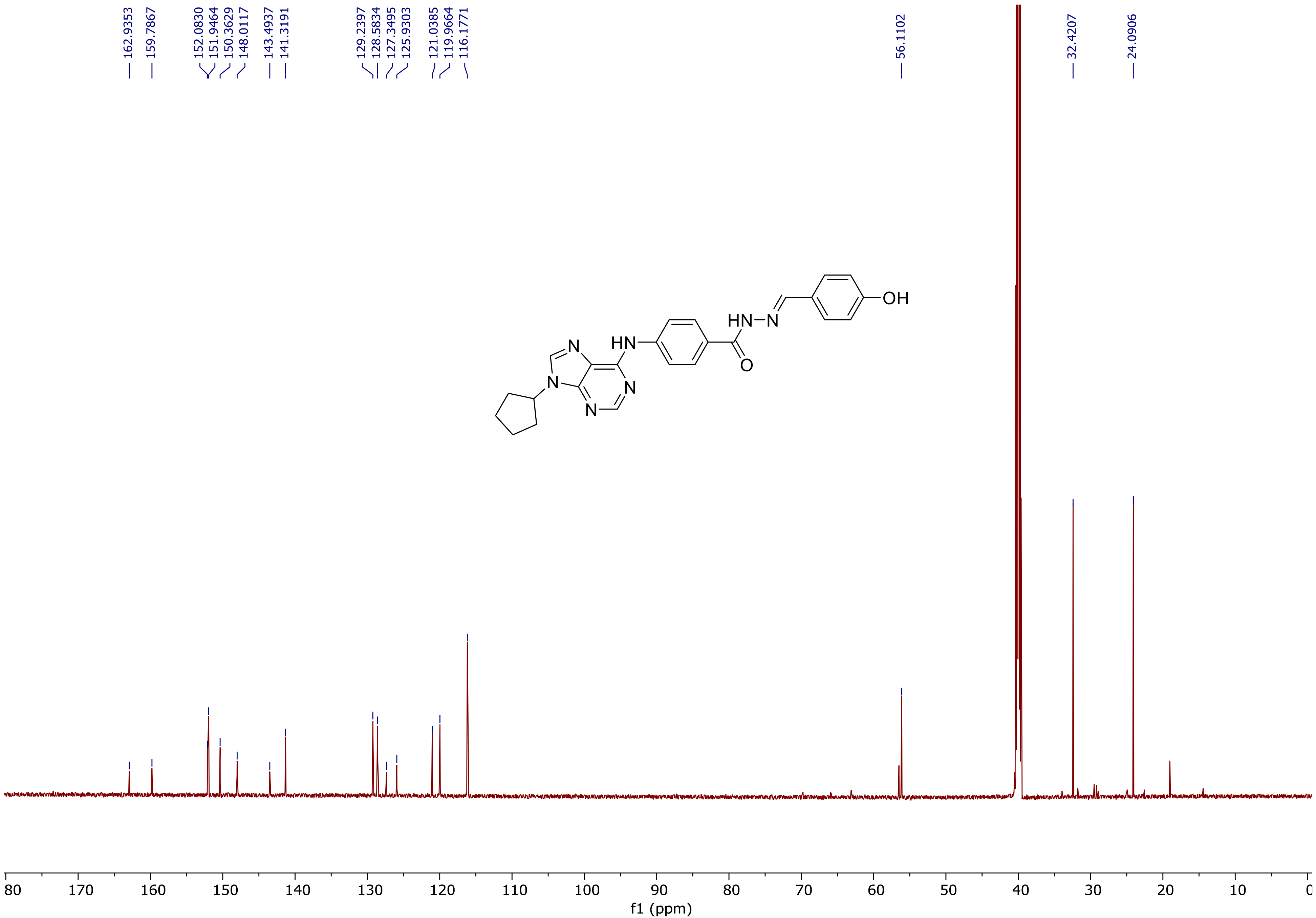

Mass spectra of compound **11b**

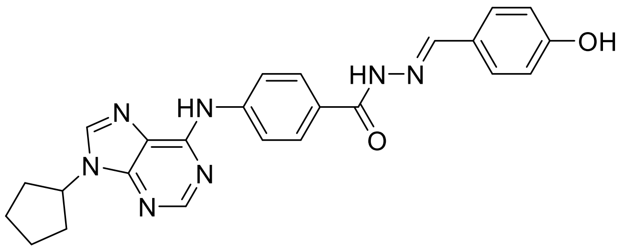

Chemical Formula: $C_{24}H_{23}N_7O_2$
Exact Mass: 441.1913

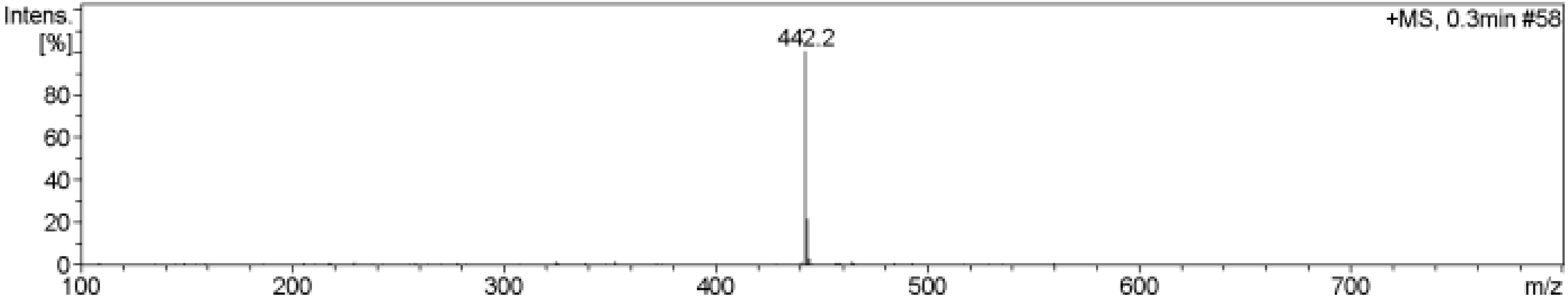

HRMS spectra of compound **11b**

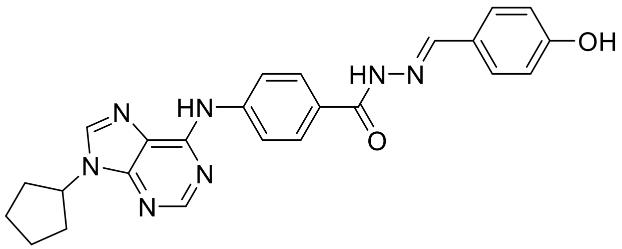

Chemical Formula: $C_{24}H_{23}N_7O_2$
Exact Mass: 441.1913

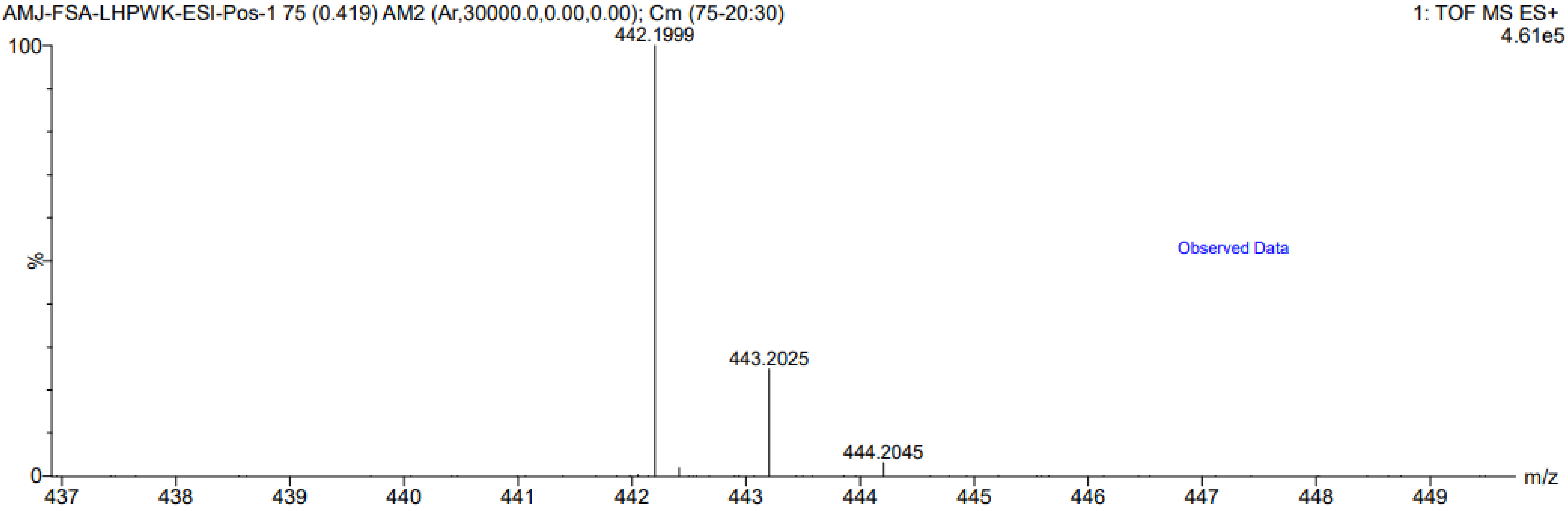

^{1}H-NMR spectra of compound **13b**

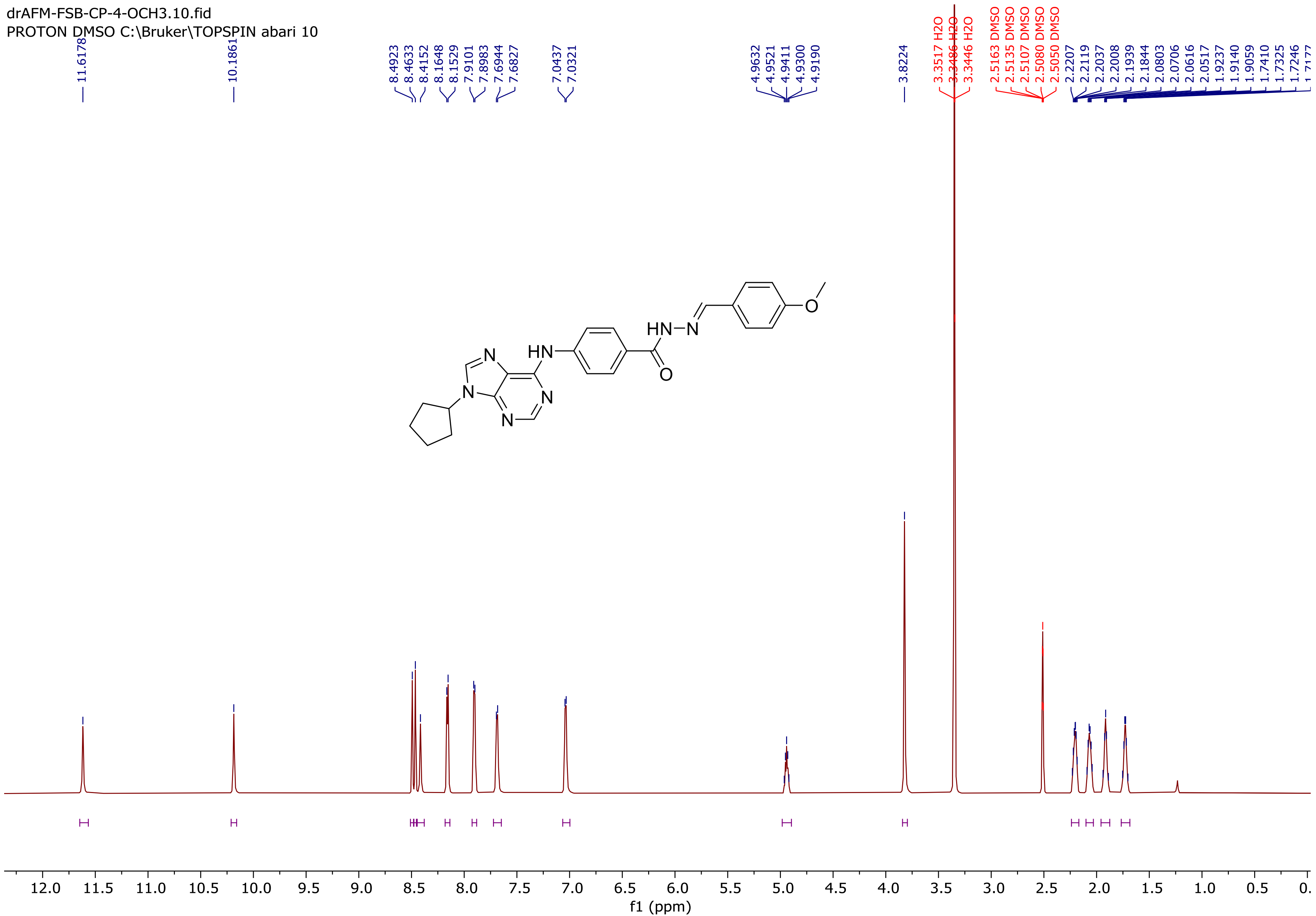

^{13}C-NMR spectra of compound **13b**

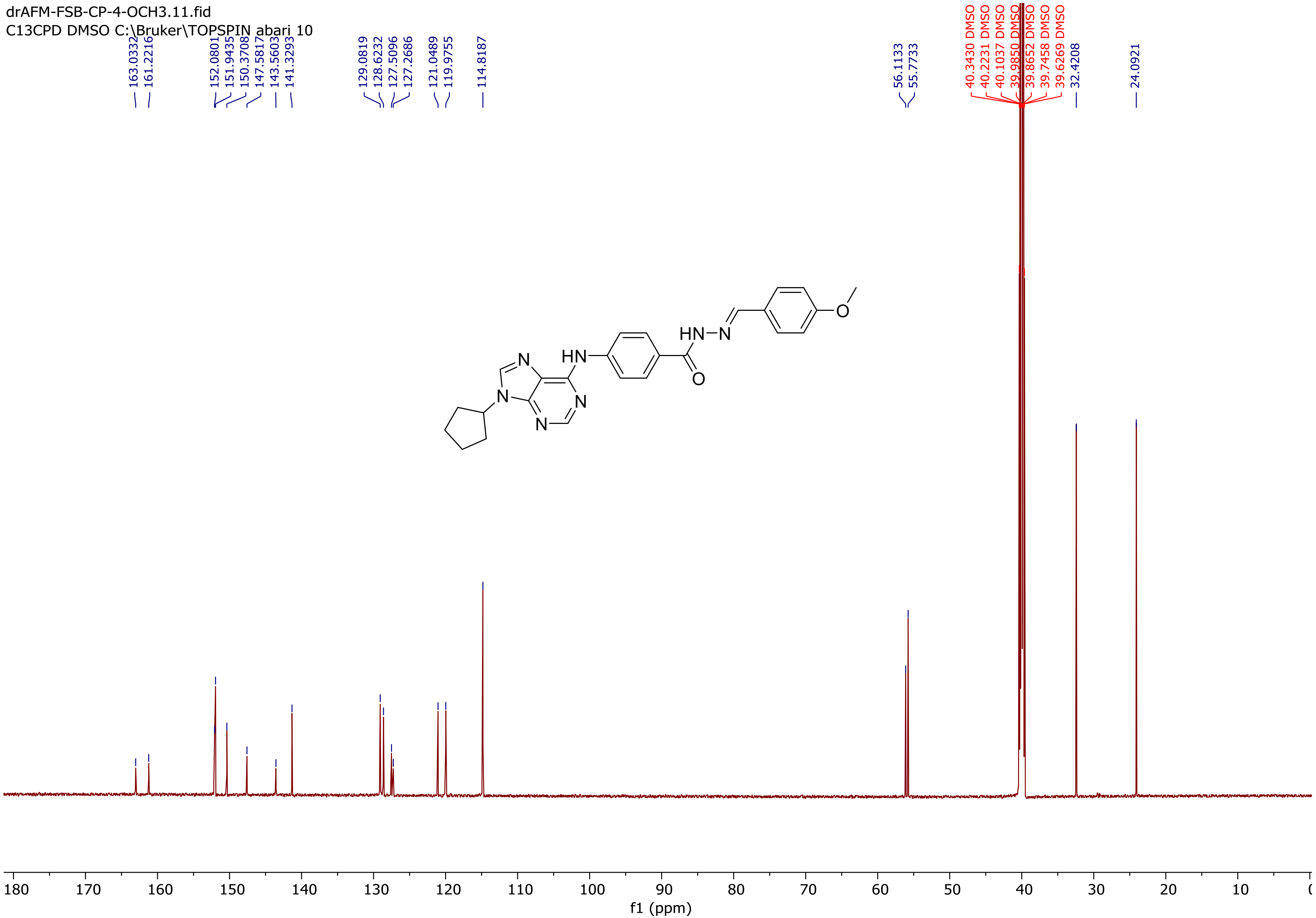

Mass spectra of compound **13b**

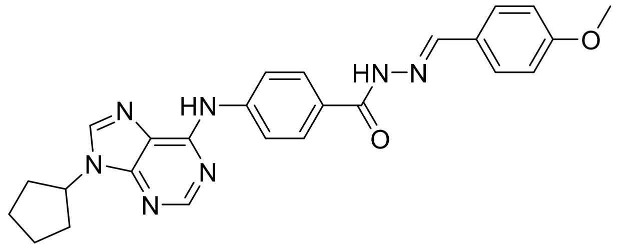

Chemical Formula: $C_{25}H_{25}N_7O_2$
Exact Mass: 455.2070

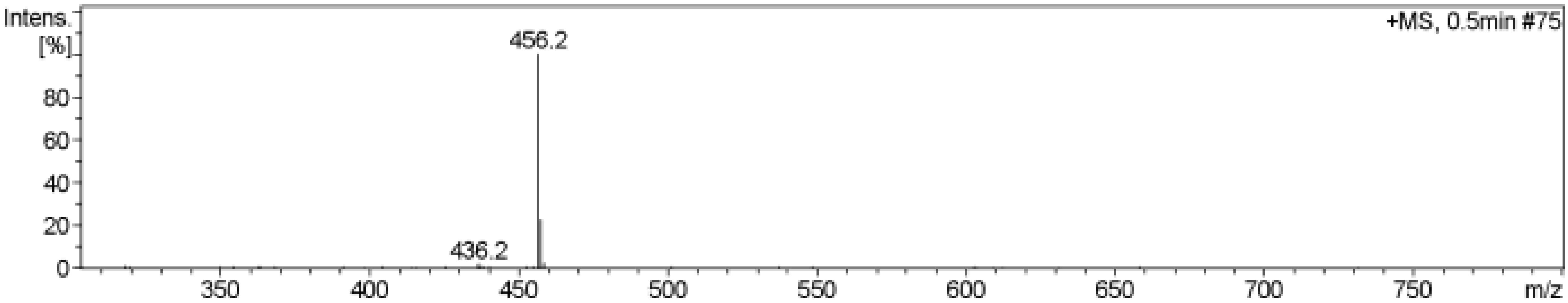

HRMS spectra of compound **13b**

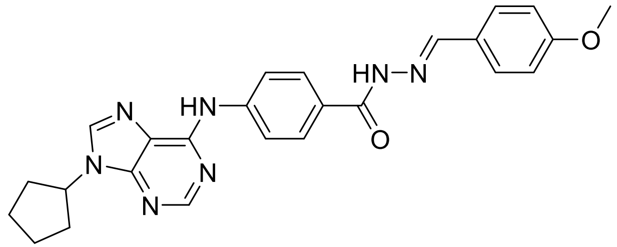

Chemical Formula: $C_{25}H_{25}N_7O_2$
Exact Mass: 455.2070

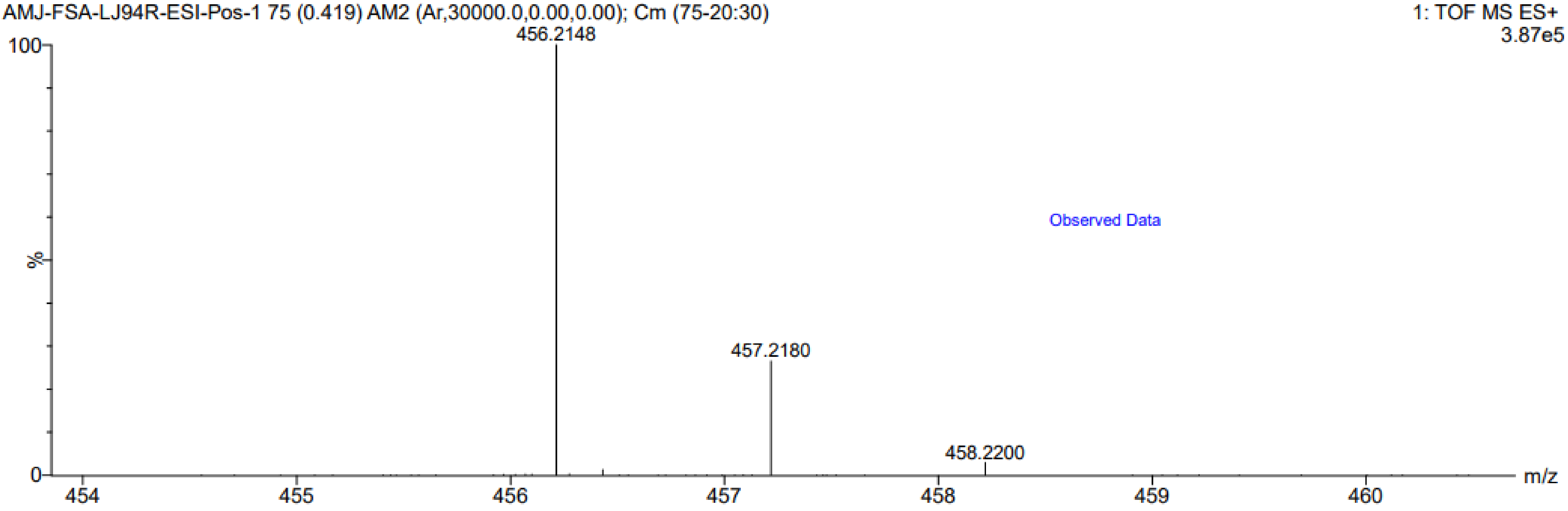

^{1}H-NMR spectra of compound **16b**

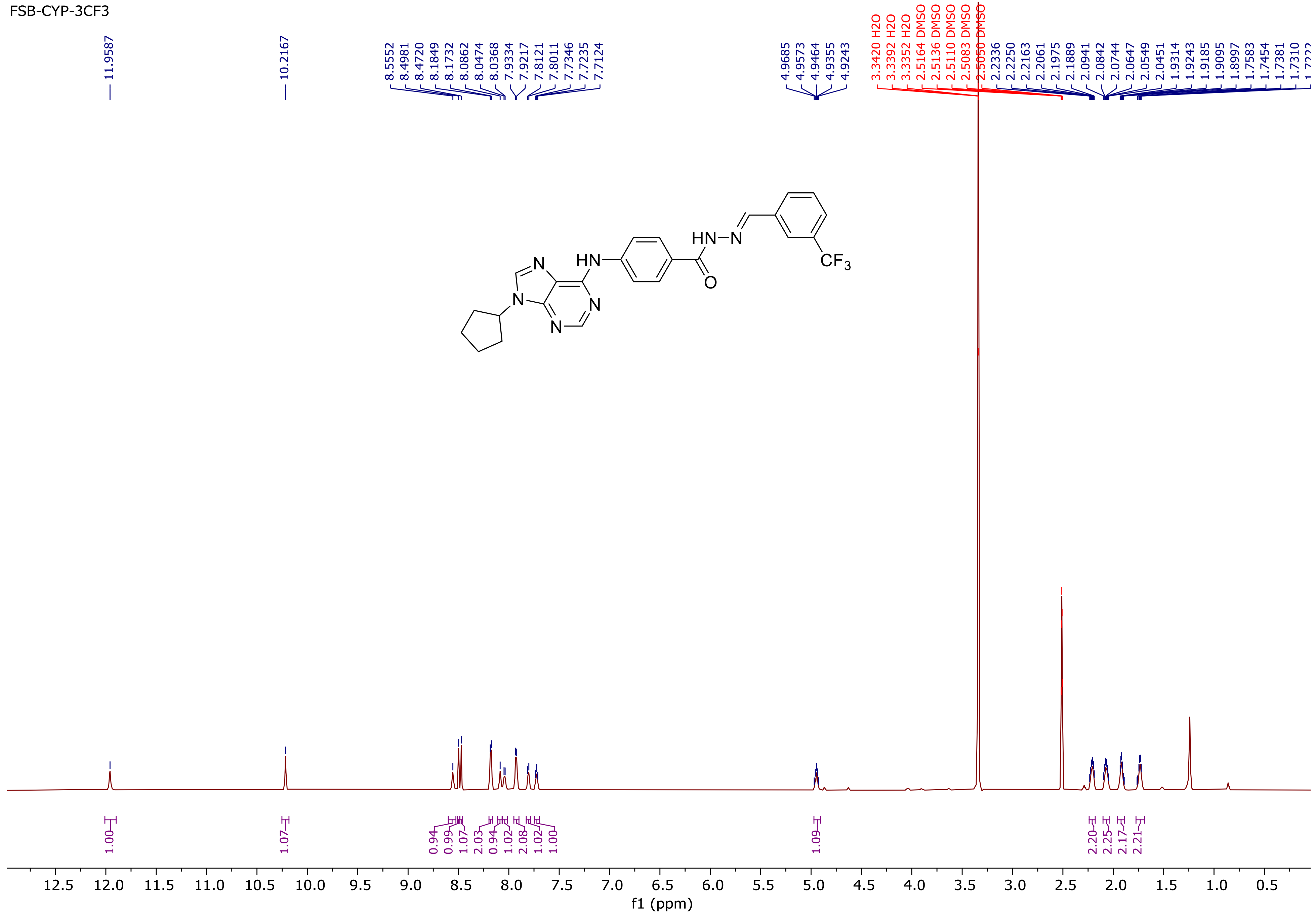

^{13}C-NMR spectra of compound **16b**

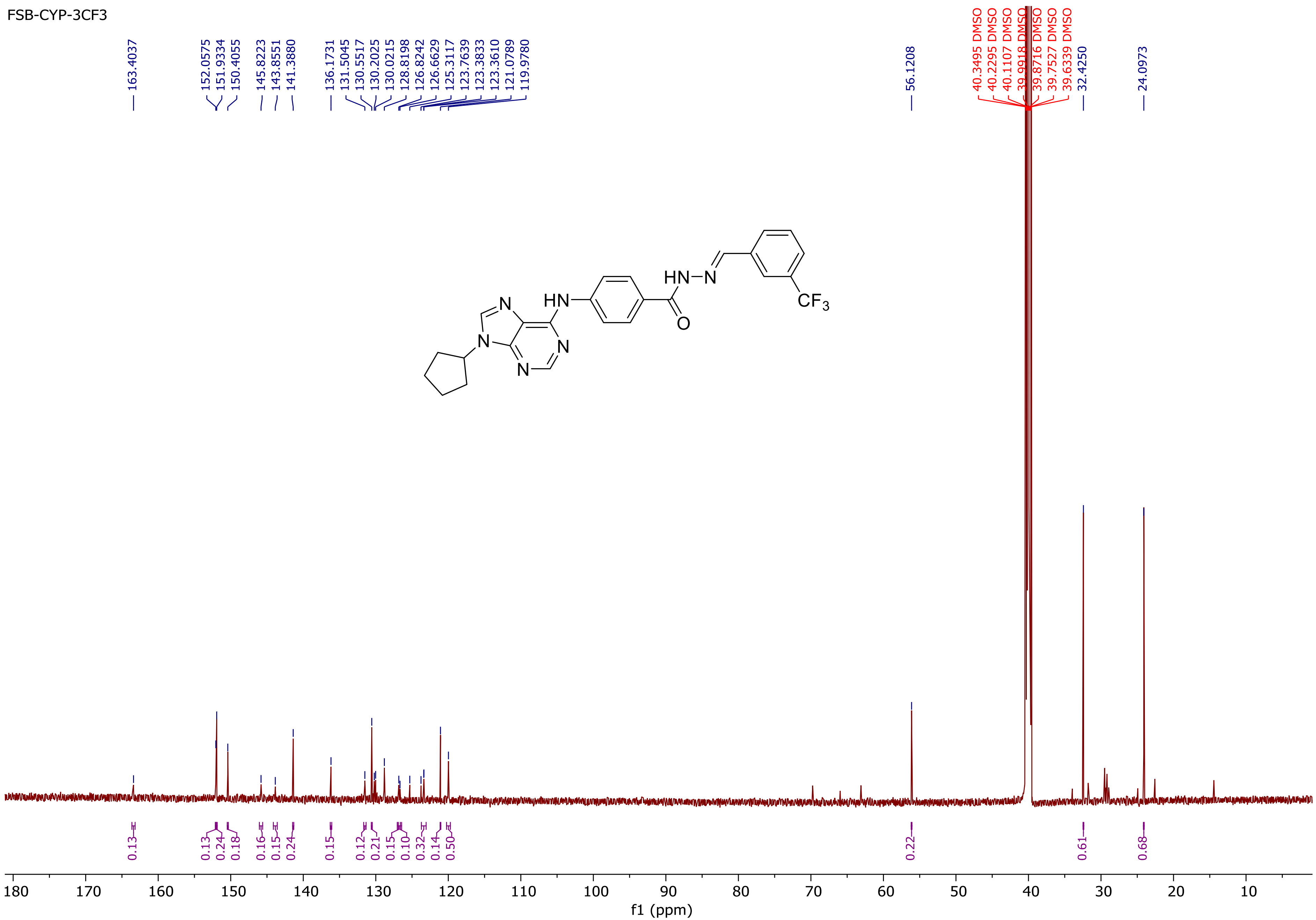

Mass spectra of compound **16b**

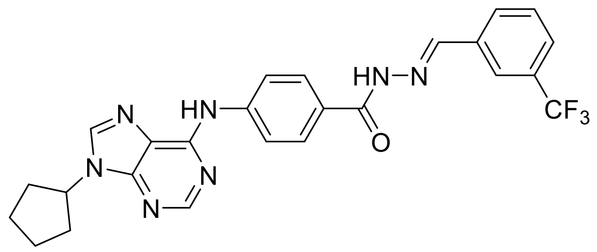

Chemical Formula: $C_{25}H_{22}F_3N_7O$
Exact Mass: 493.1838

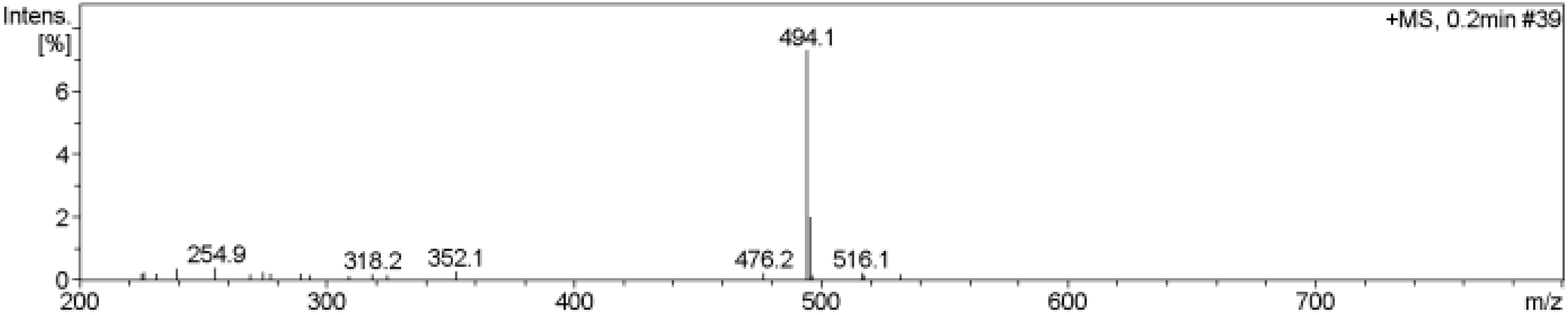

HRMS spectra of compound **16b**

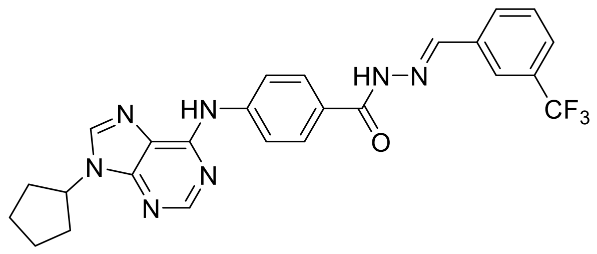

Chemical Formula: $C_{25}H_{22}F_3N_7O$
Exact Mass: 493.1838

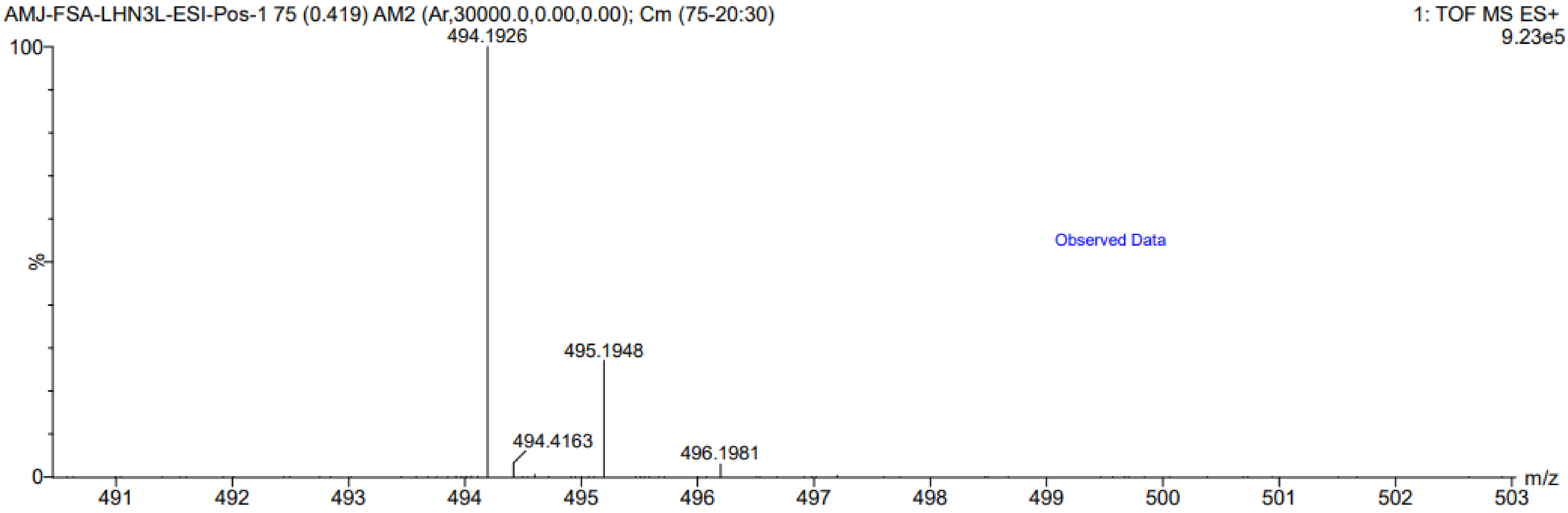

^{1}H-NMR spectra of compound **21b**

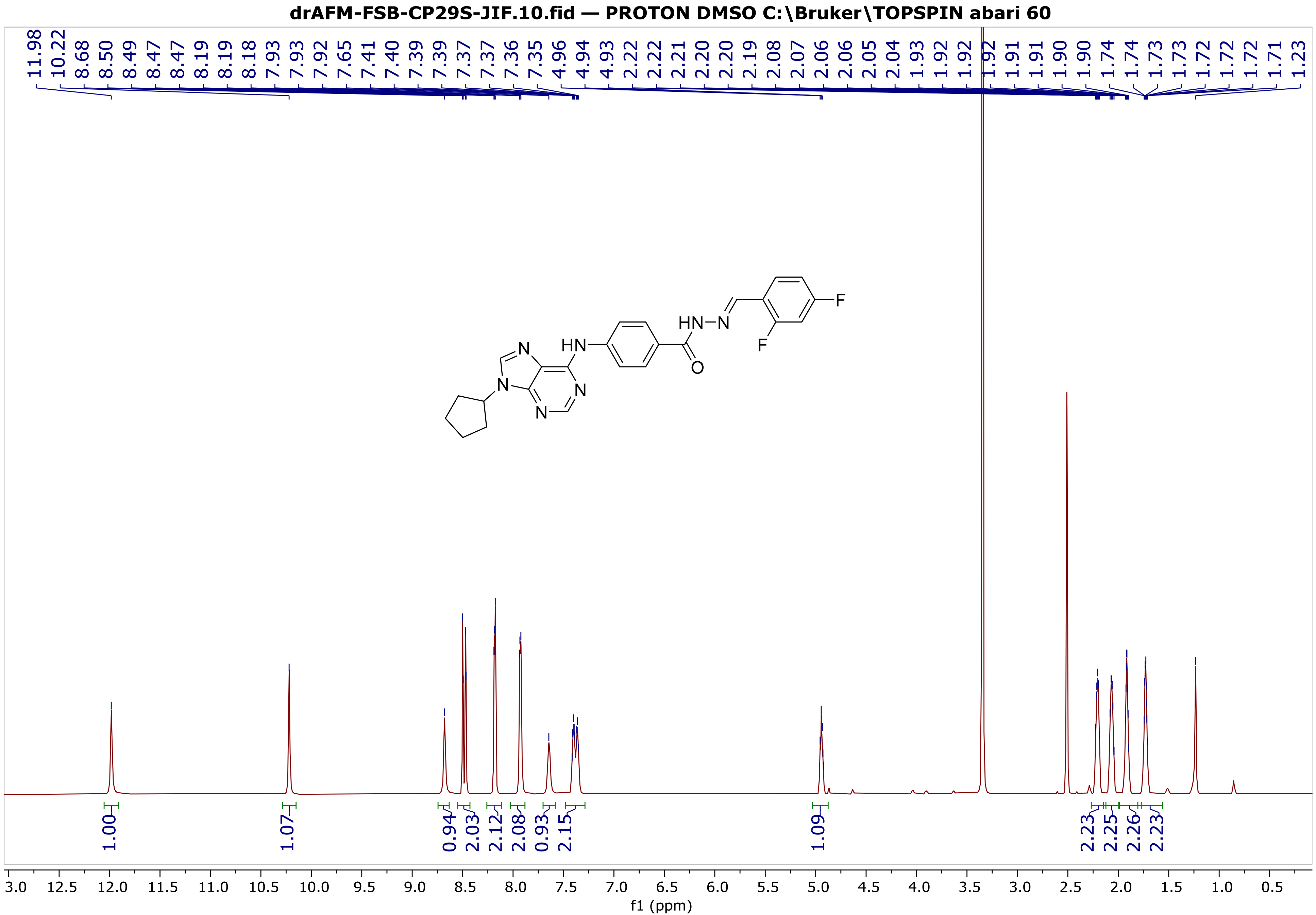

^{13}C-NMR spectra of compound **21b**

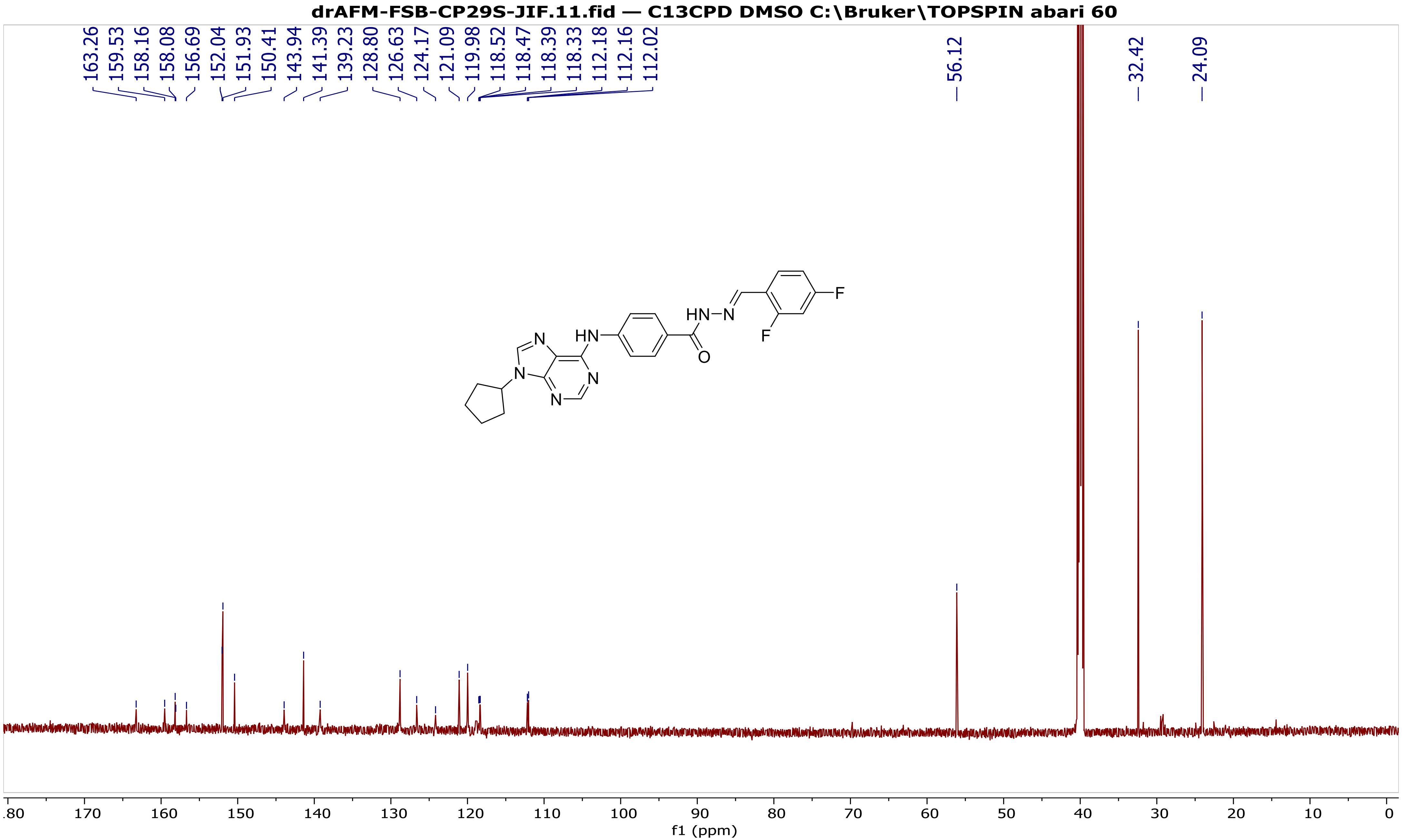

Mass spectra of compound **21b**

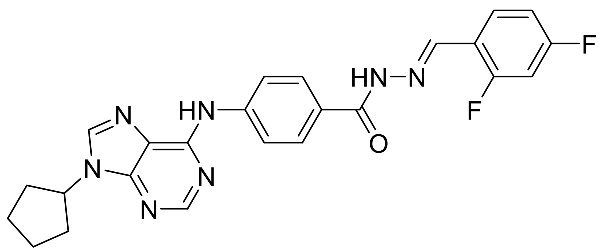

Chemical Formula: $C_{24}H_{21}F_2N_7O$
Exact Mass: 461.1776

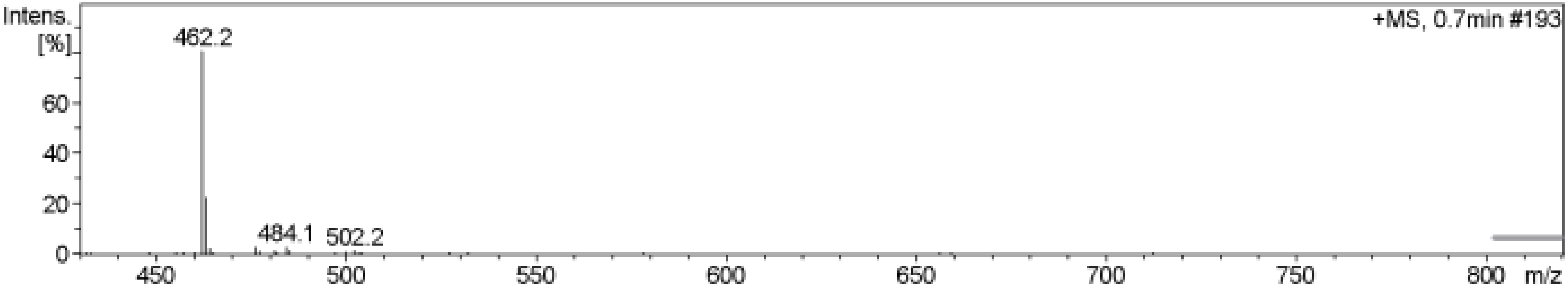

HRMS spectra of compound **21b**

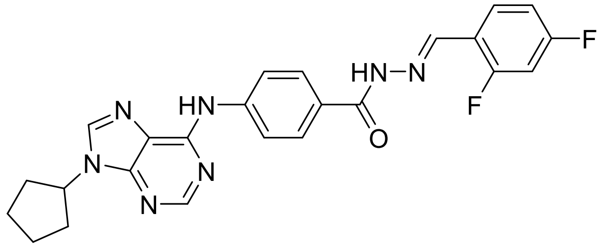

Chemical Formula: $C_{24}H_{21}F_2N_7O$
Exact Mass: 461.1776

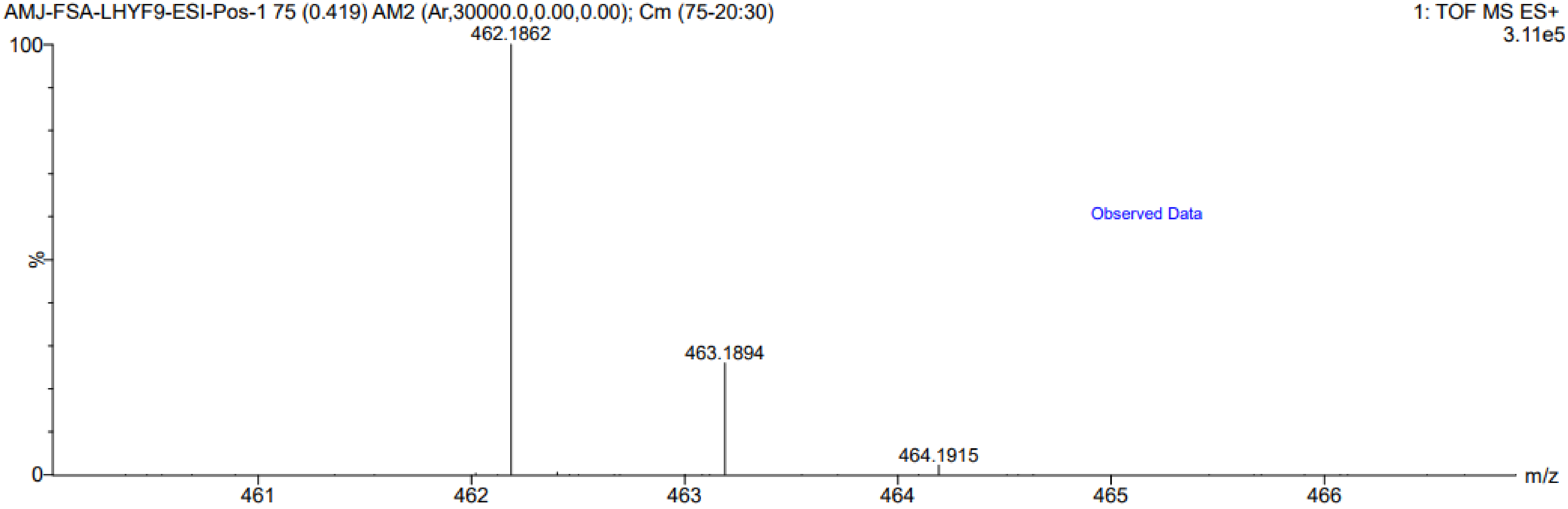

^{1}H-NMR spectra of compound **22b**

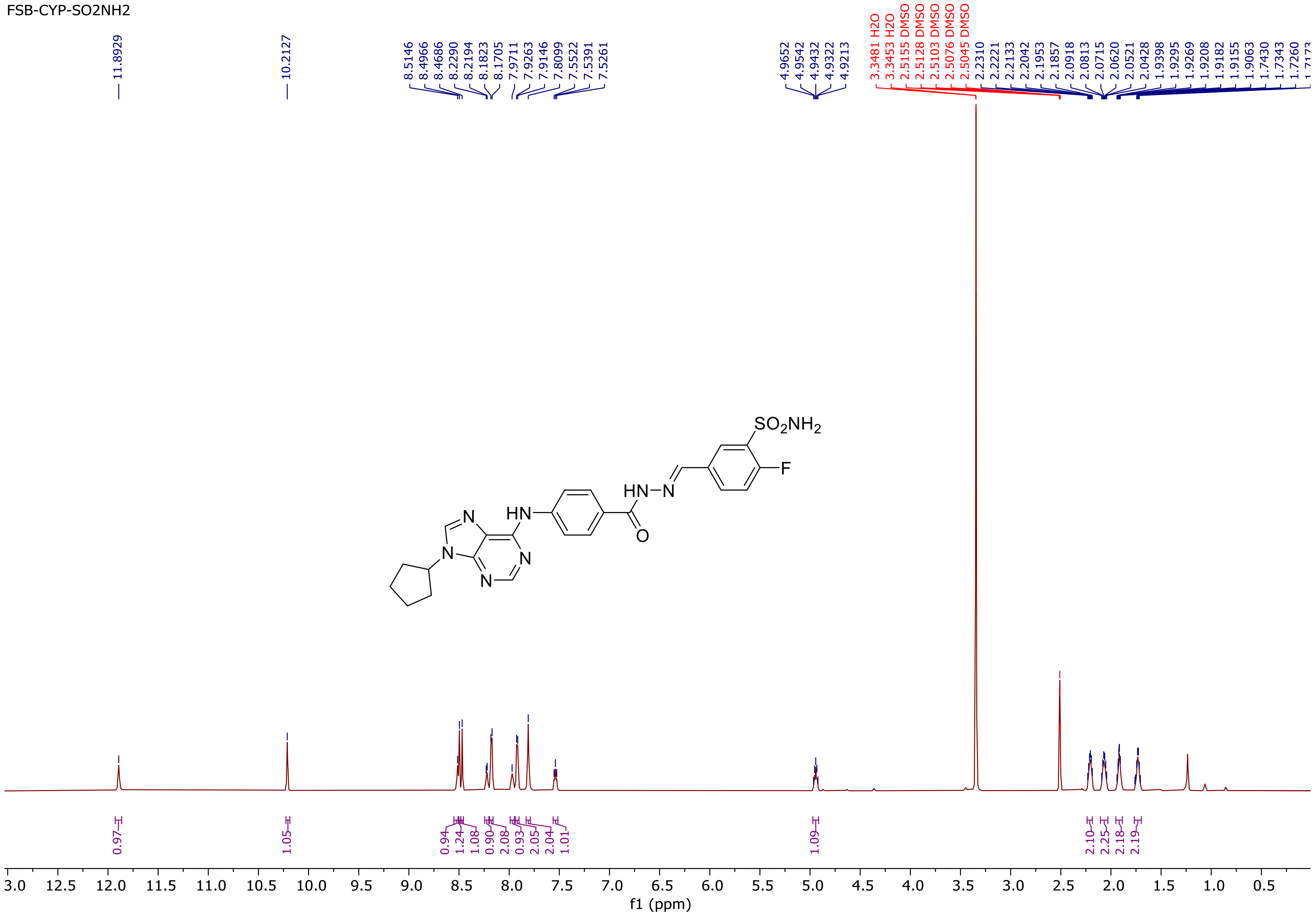

^{13}C-NMR spectra of compound **22b**

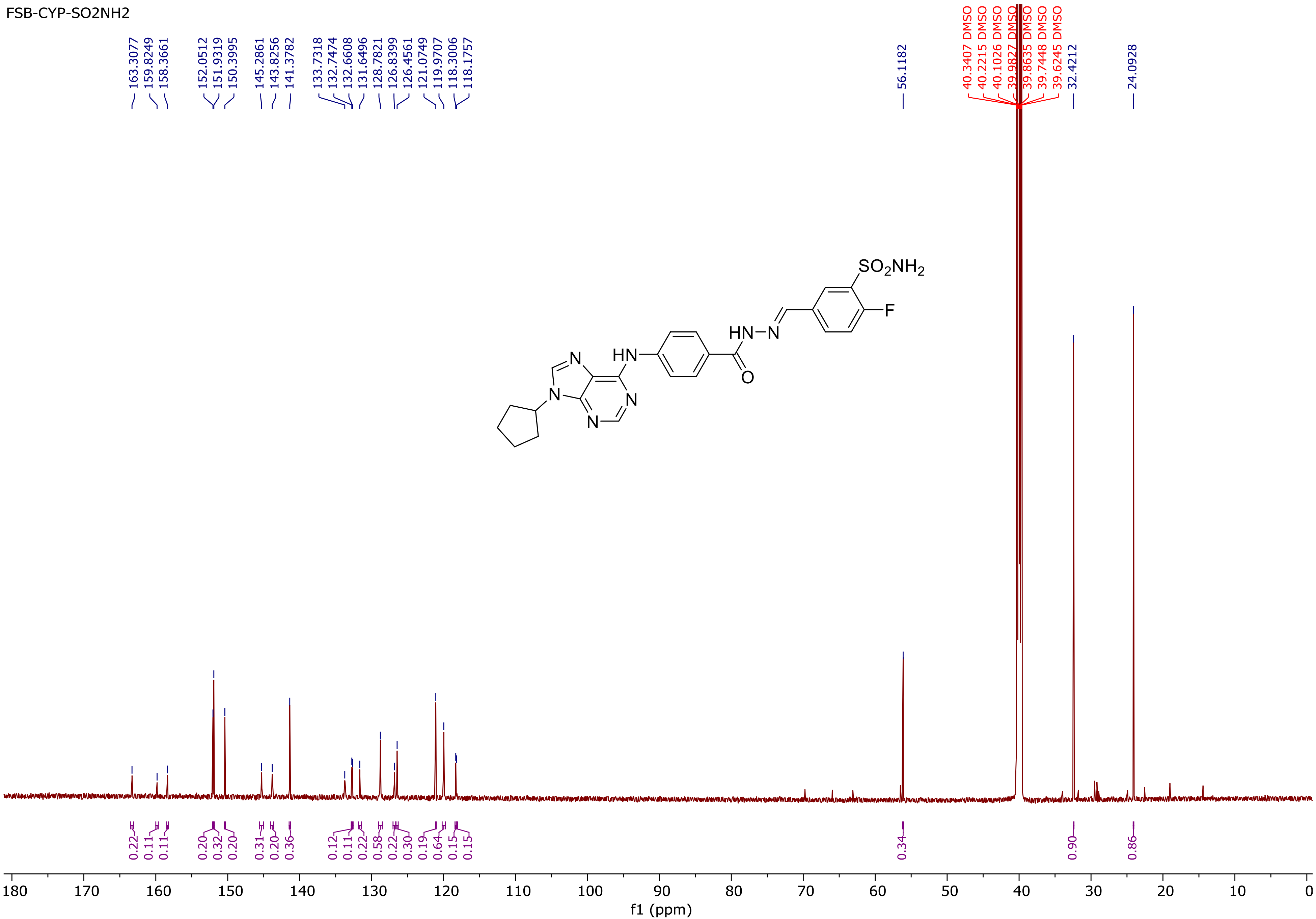

Mass spectra of compound **22b**

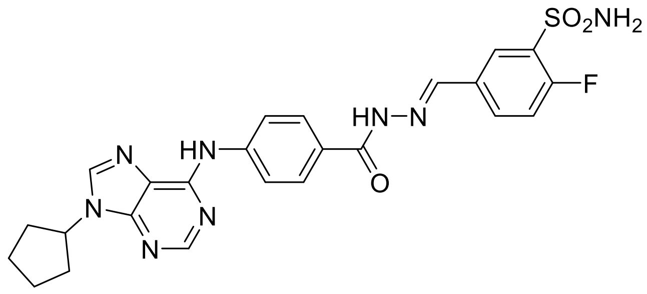

Chemical Formula: $C_{24}H_{23}FN_8O_3S$
Exact Mass: 522.1598

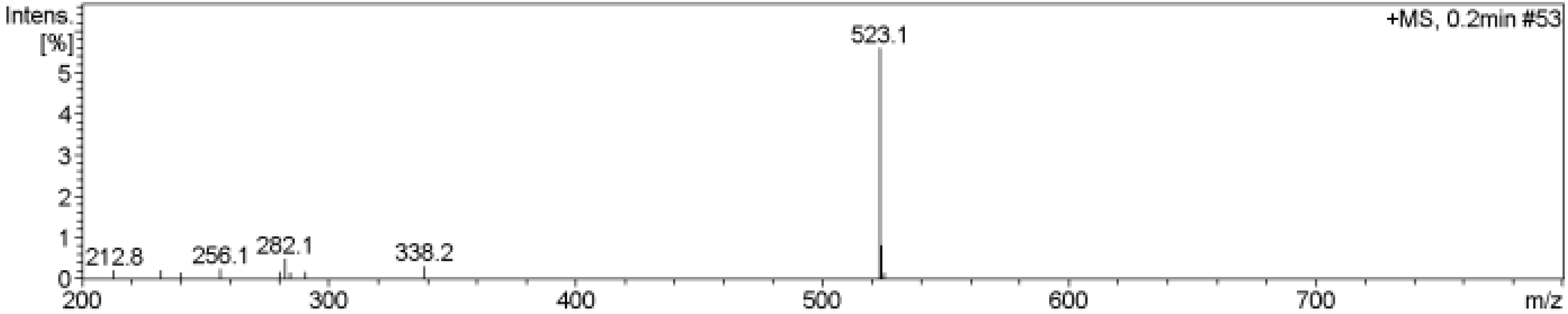

HRMS spectra of compound **22b**

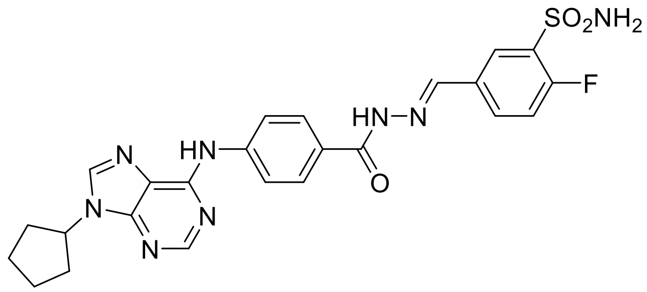

Chemical Formula: $C_{24}H_{23}FN_8O_3S$
Exact Mass: 522.1598

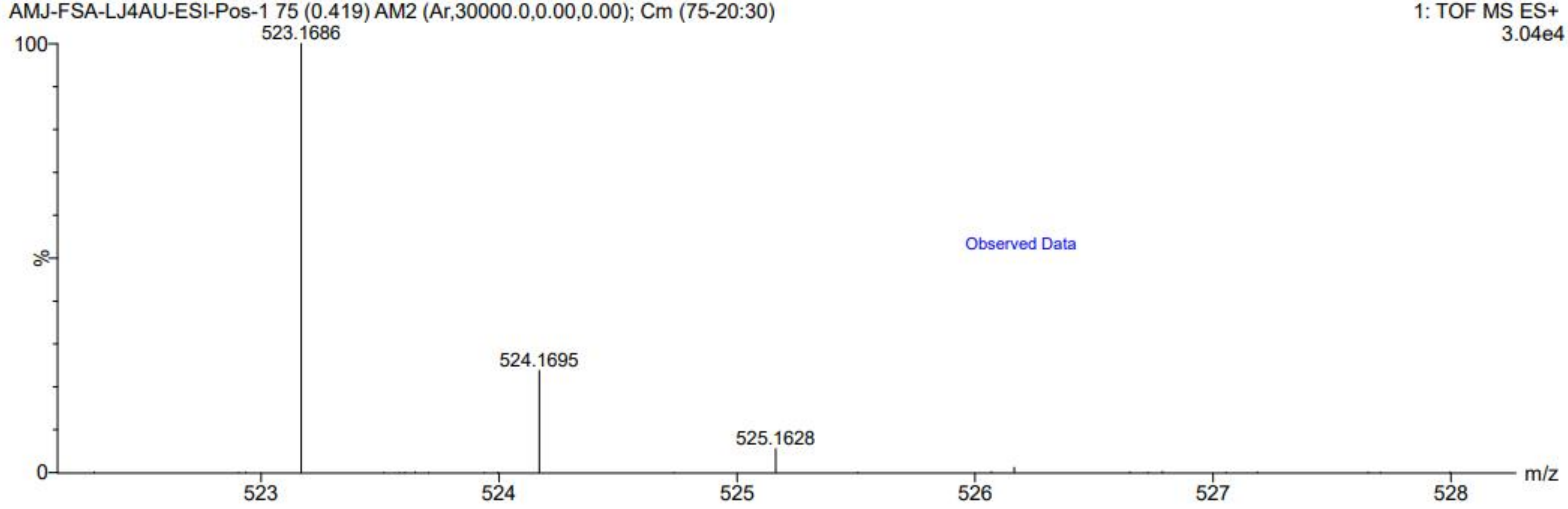

^{1}H-NMR spectra of compound **17b**

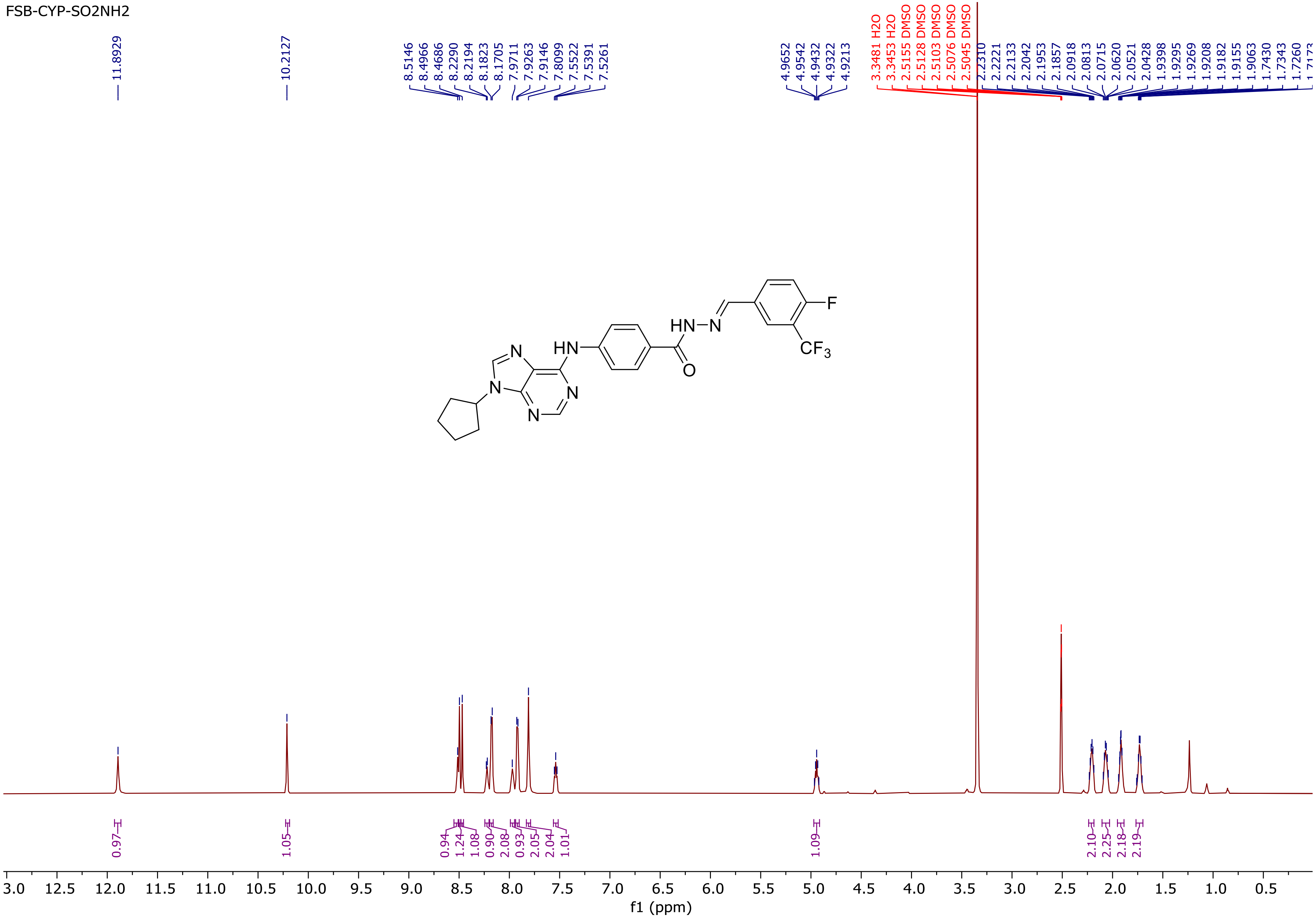

^{13}C-NMR spectra of compound **17b**

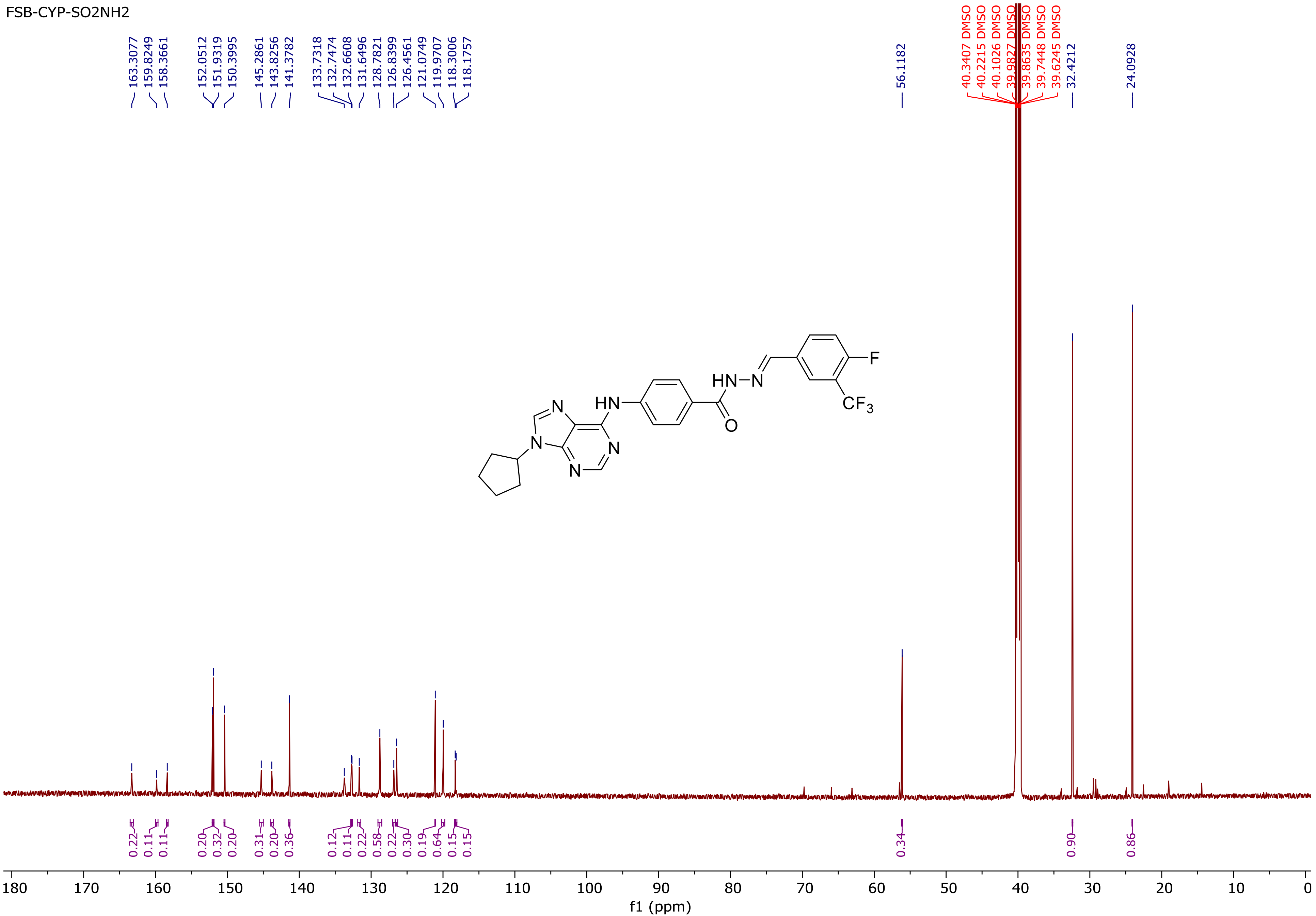

Mass spectra of compound **17b**

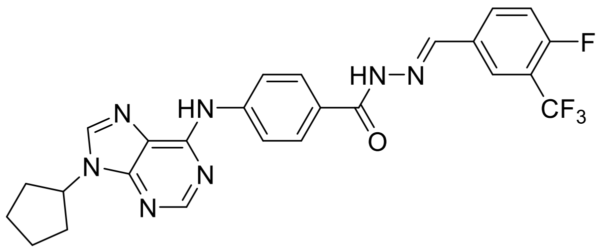

Chemical Formula: $C_{25}H_{21}F_4N_7O$
Exact Mass: 511.1744

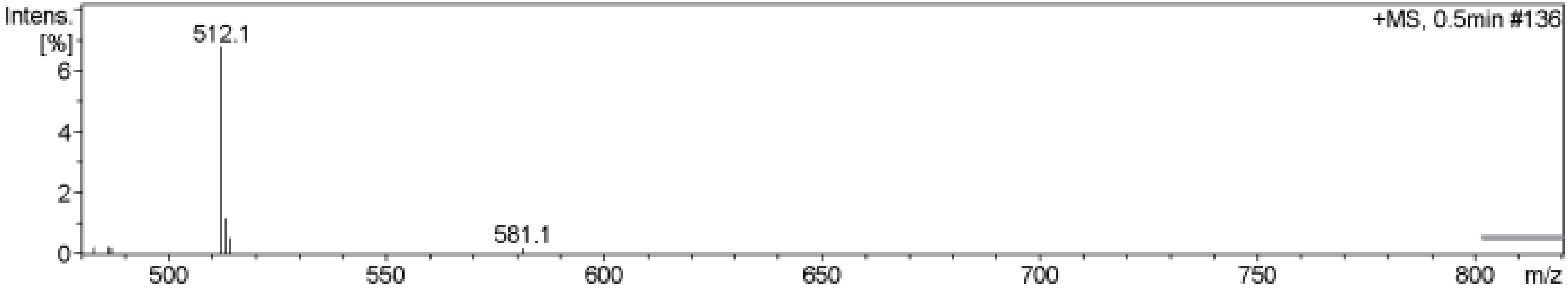

HRMS spectra of compound **17b**

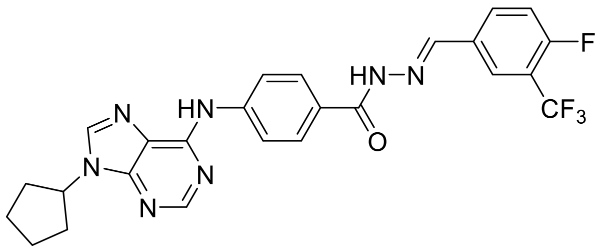

Chemical Formula: $C_{25}H_{21}F_4N_7O$
Exact Mass: 511.1744

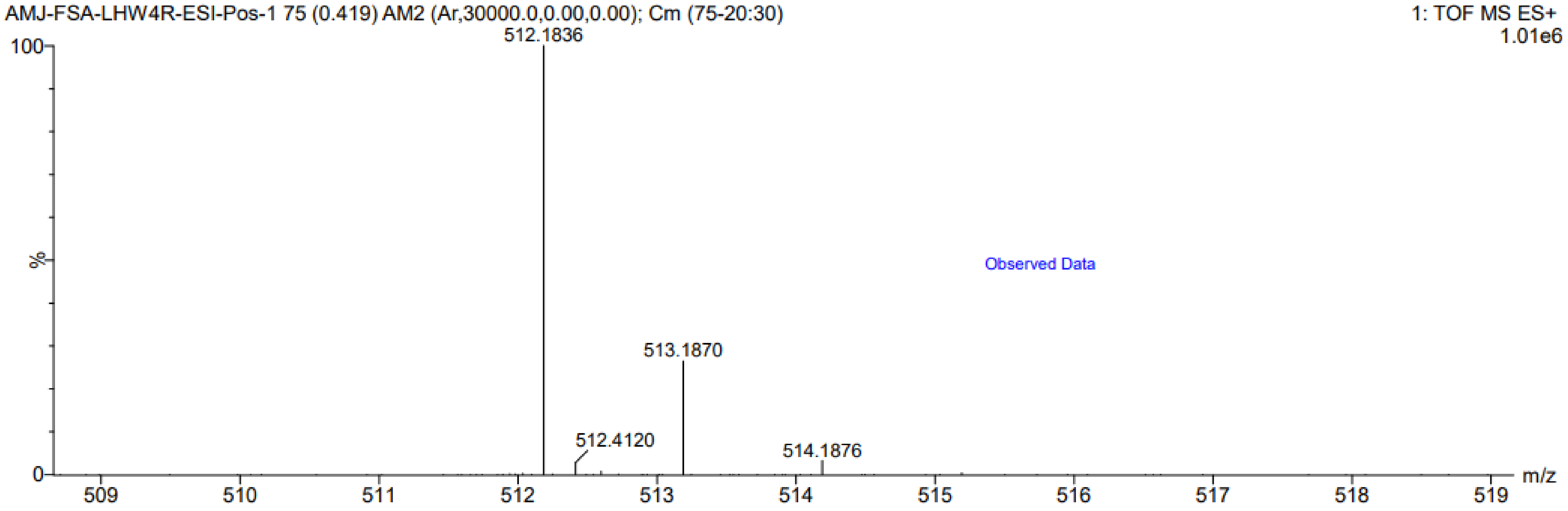

^{1}H-NMR spectra of compound **18b**

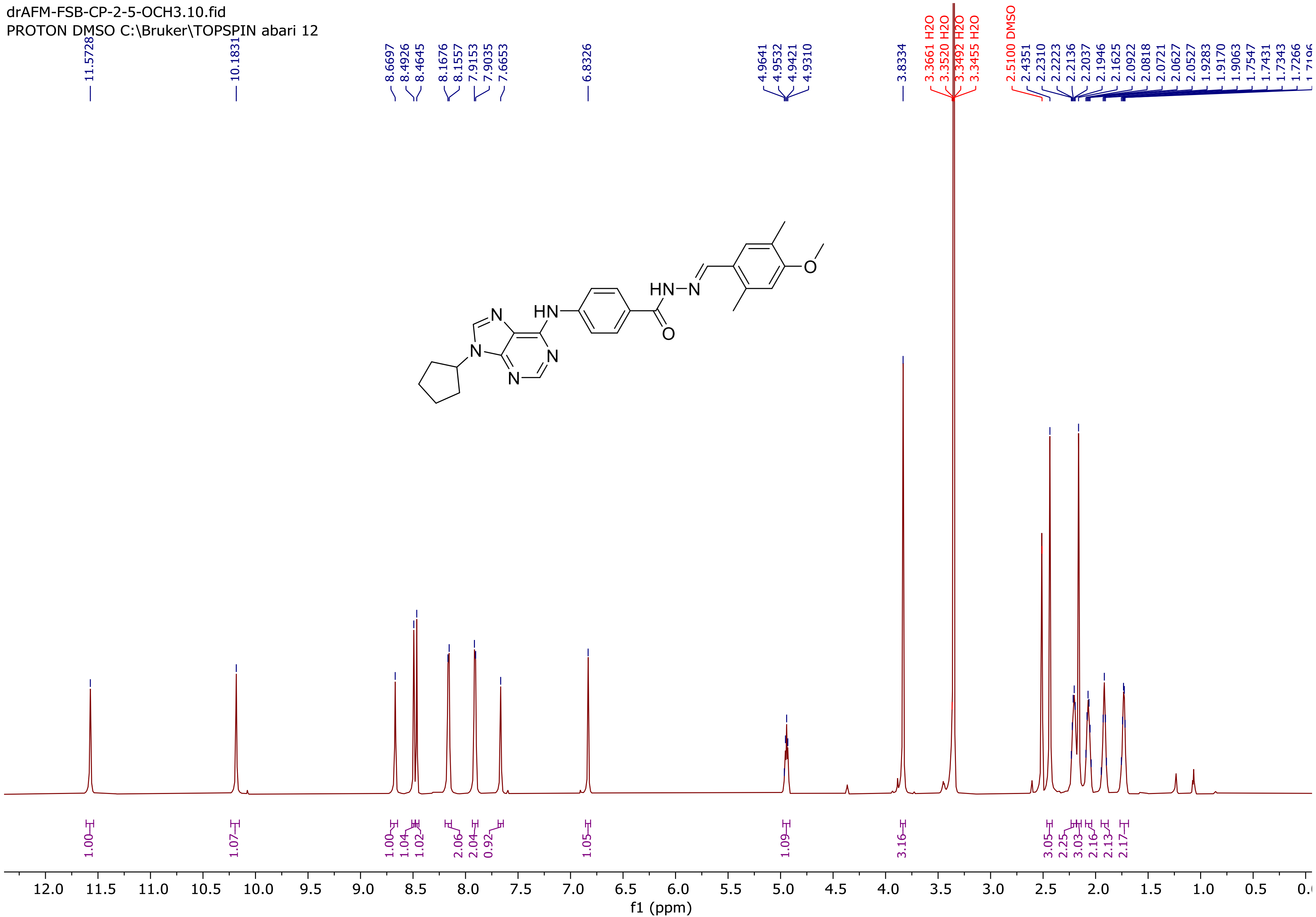

^{13}C-NMR spectra of compound **18b**

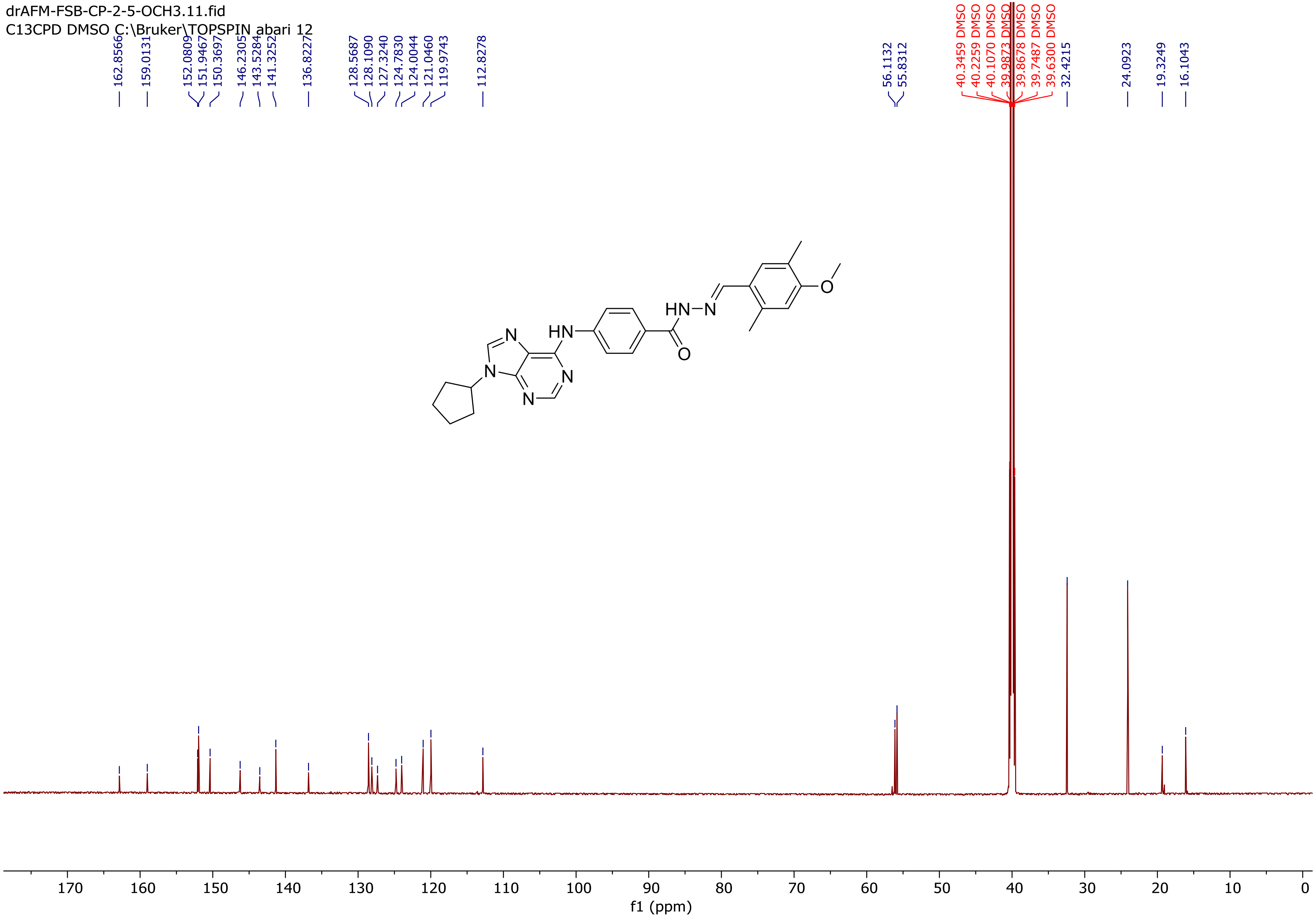

Mass spectra of compound **18b**

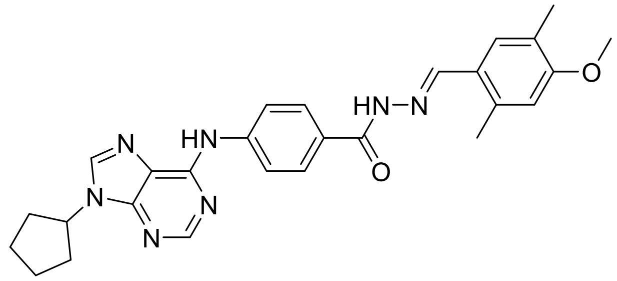

Chemical Formula: $C_{27}H_{29}N_7O_2$
Exact Mass: 483.2383

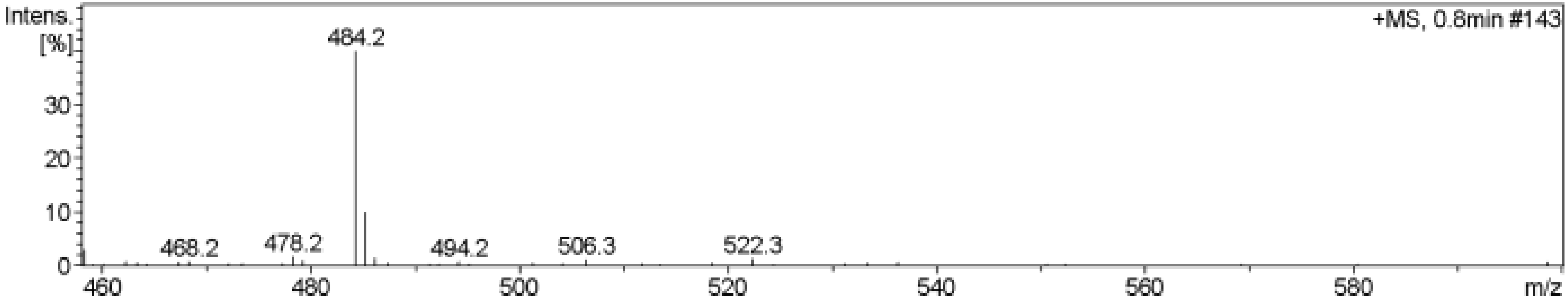

HRMS spectra of compound **18b**

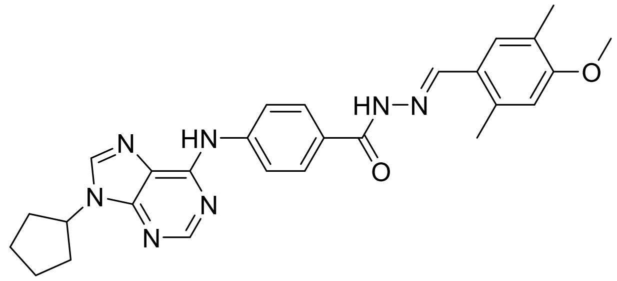

Chemical Formula: $C_{27}H_{29}N_7O_2$
Exact Mass: 483.2383

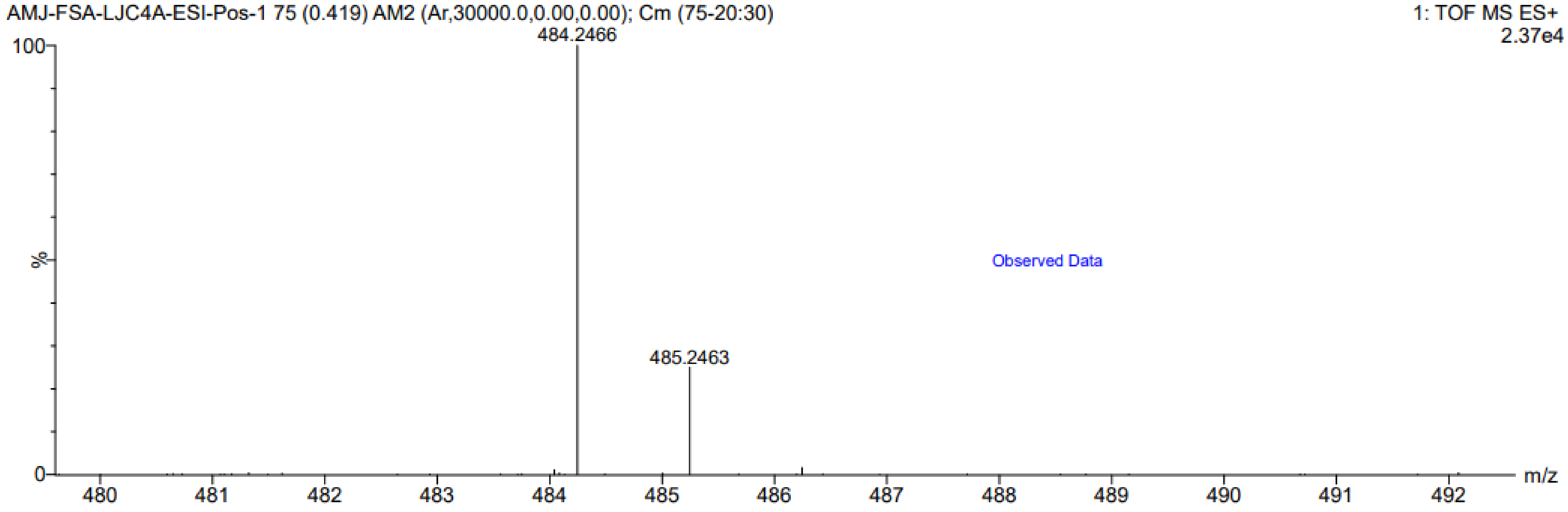

^{1}H-NMR spectra of compound **19b**

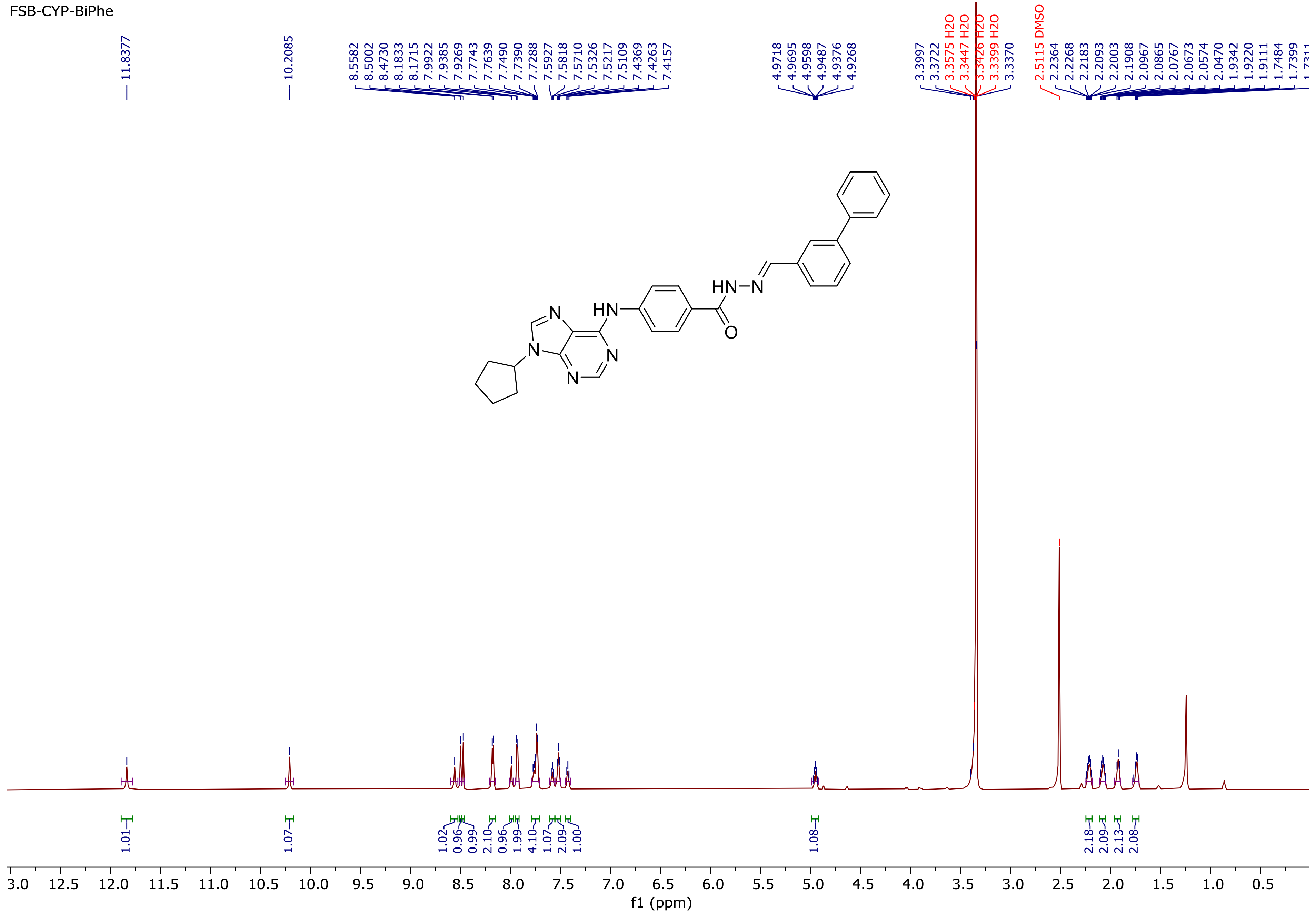

^{13}C-NMR spectra of compound **19b**

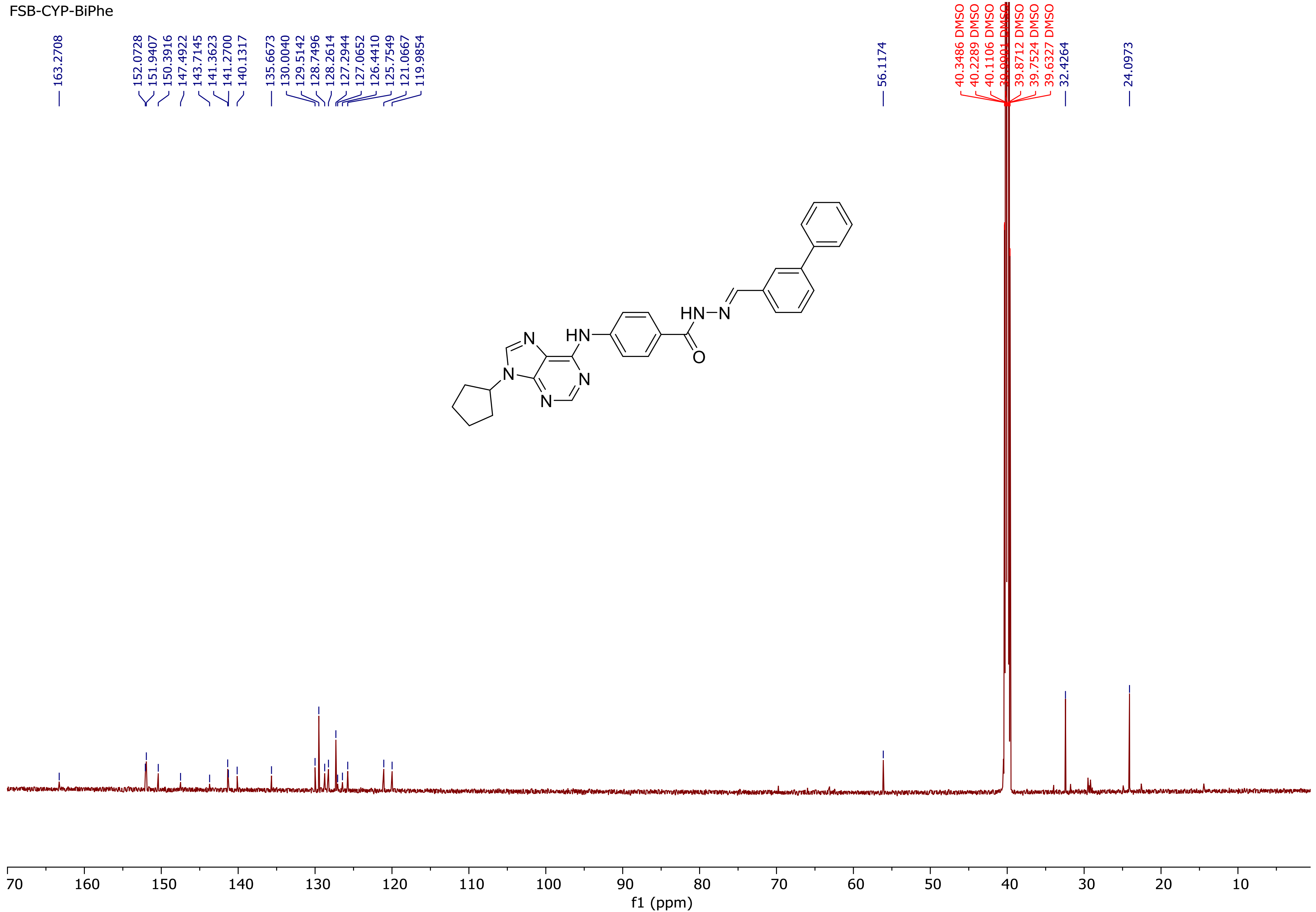

Mass spectra of compound **19b**

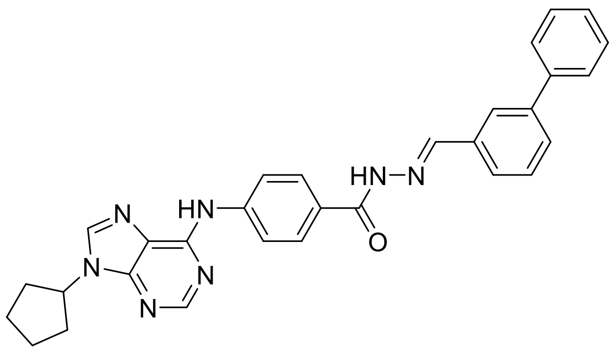

Chemical Formula: $C_{30}H_{27}N_7O$
Exact Mass: 501.2277

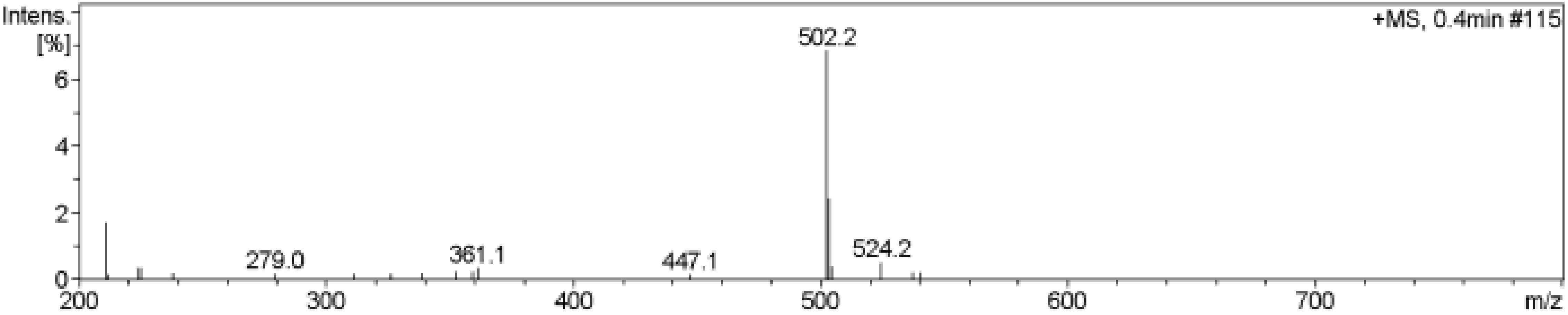

HRMS spectra of compound **19b**

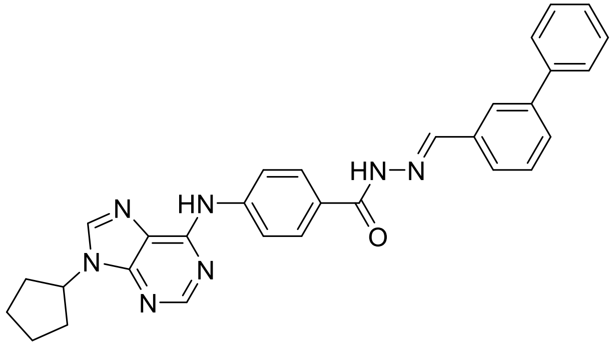

Chemical Formula: $C_{30}H_{27}N_7O$
Exact Mass: 501.2277

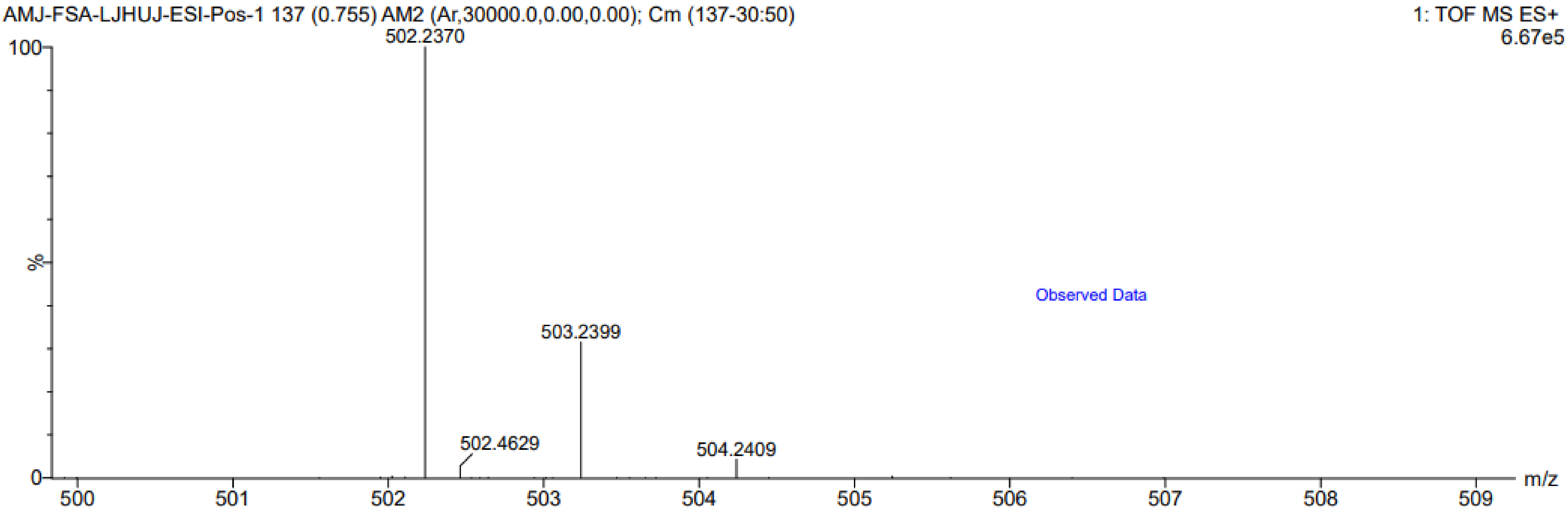

^{1}H-NMR spectra of compound **20b**

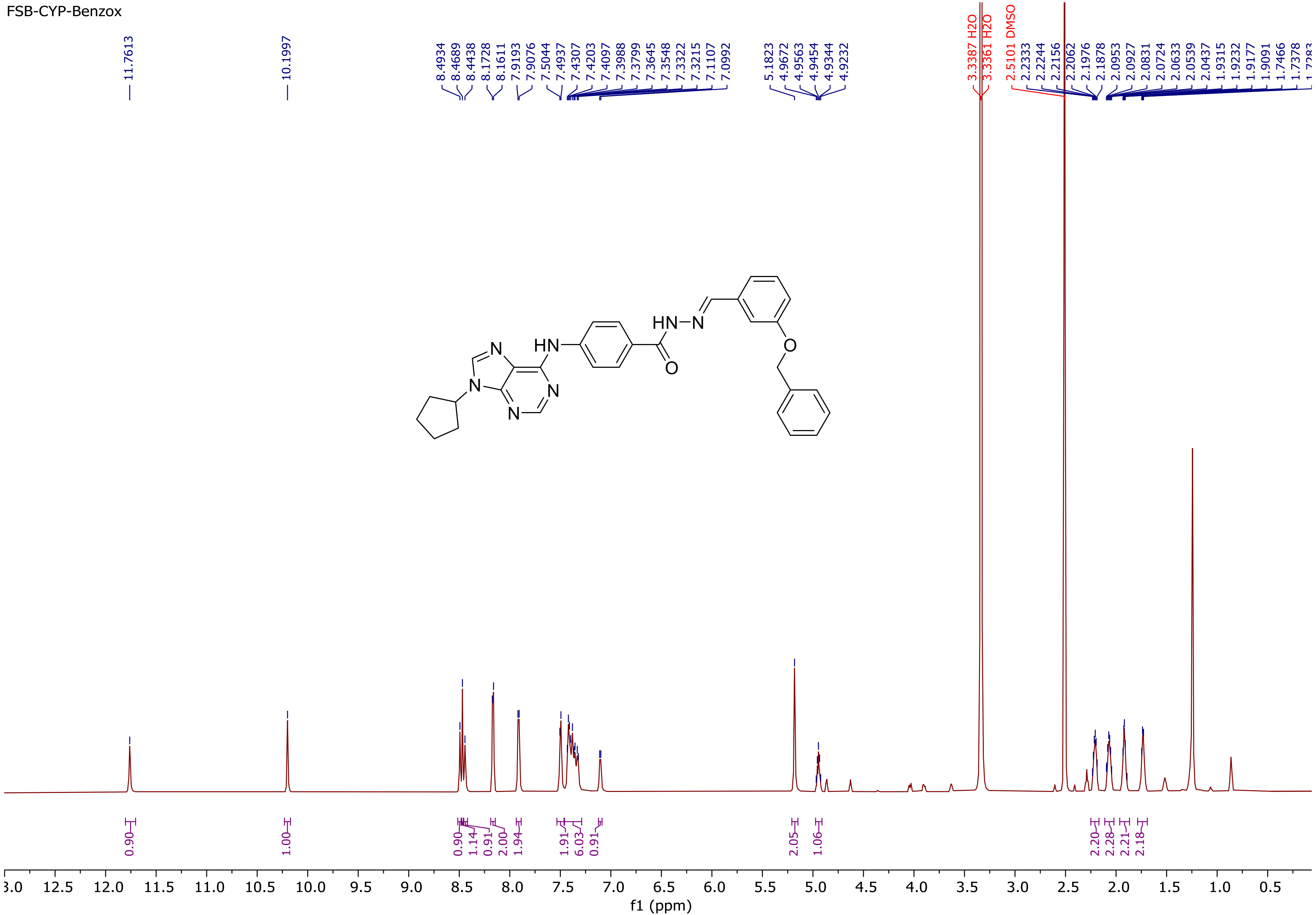

^{13}C-NMR spectra of compound **20b**

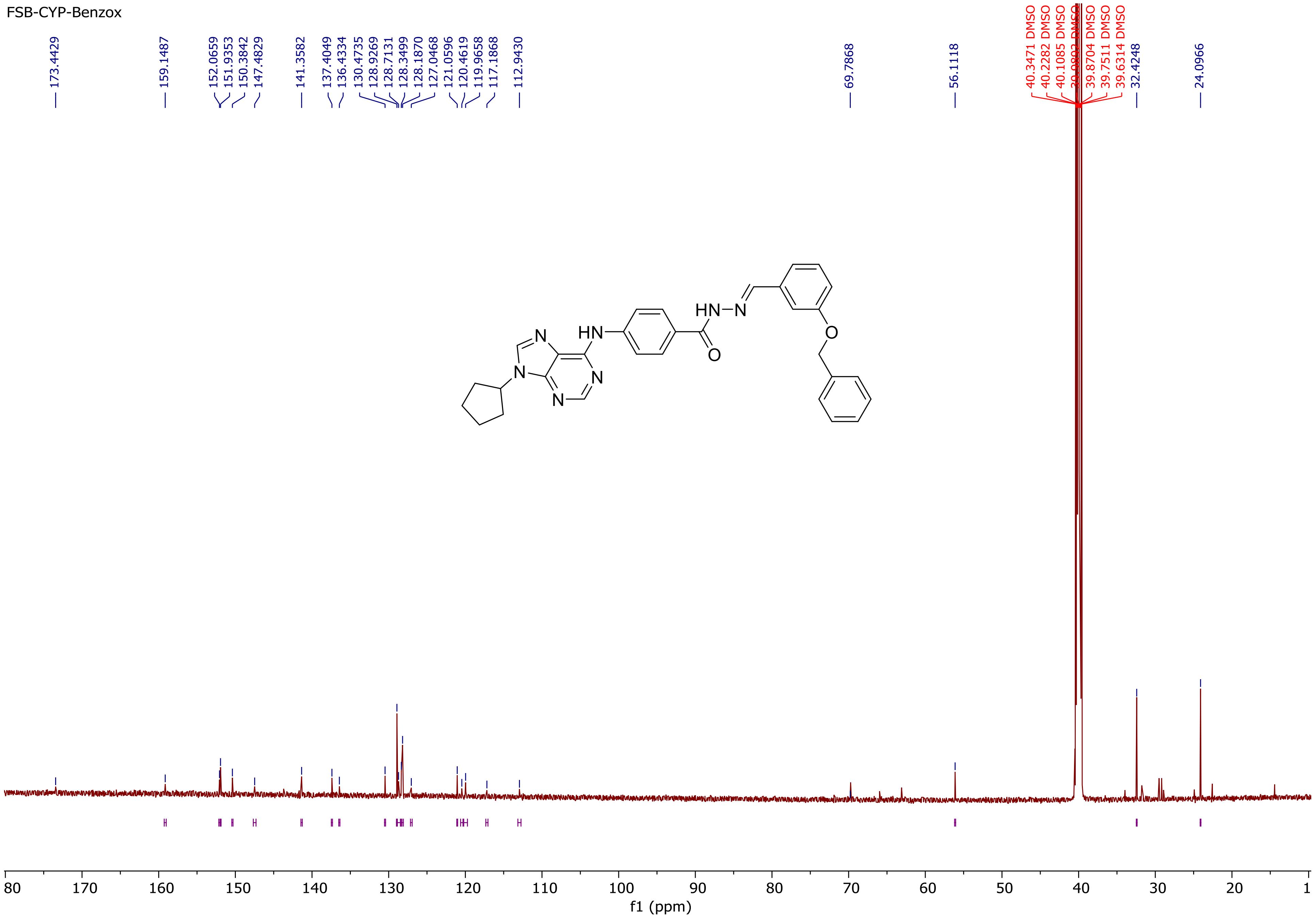

Mass spectra of compound **20b**

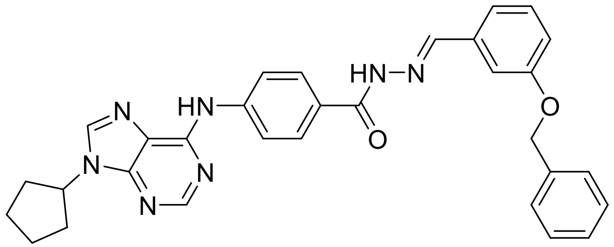

Chemical Formula: $C_{31}H_{29}N_7O_2$
Exact Mass: 531.2383

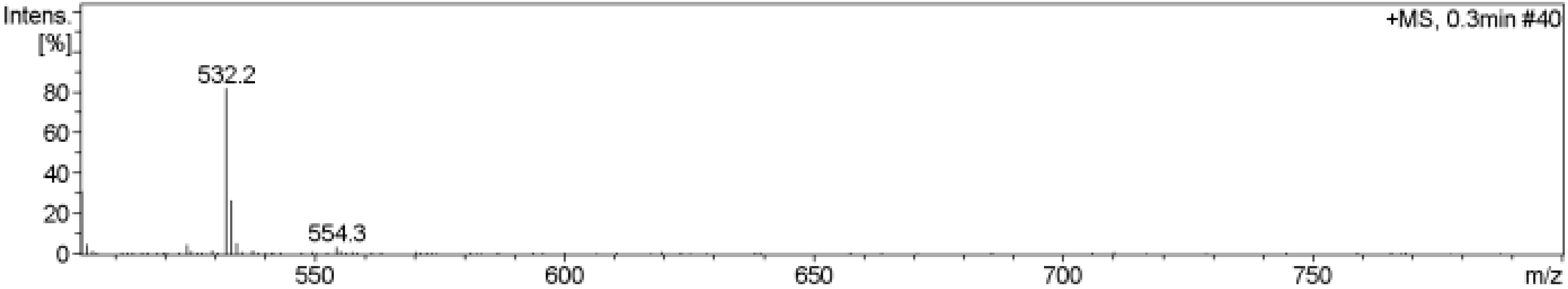

HRMS spectra of compound **20b**

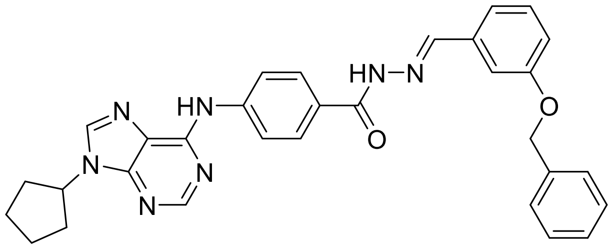

Chemical Formula: $C_{31}H_{29}N_7O_2$
Exact Mass: 531.2383

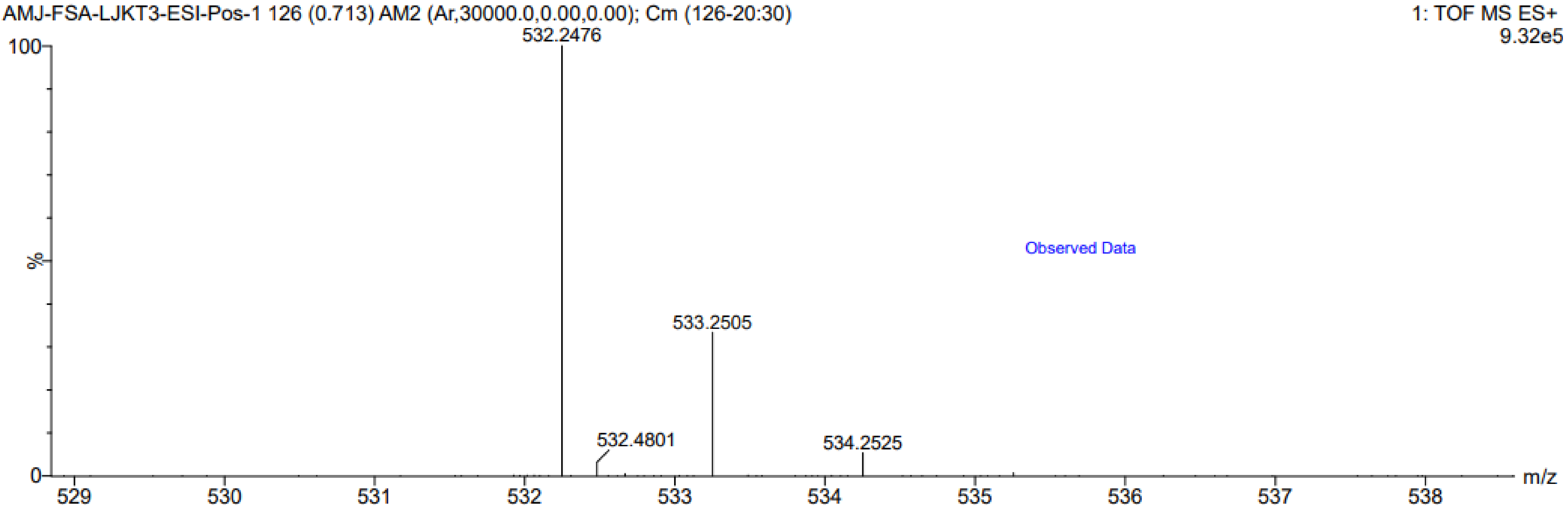

^{1}H-NMR spectra of compound **23b**

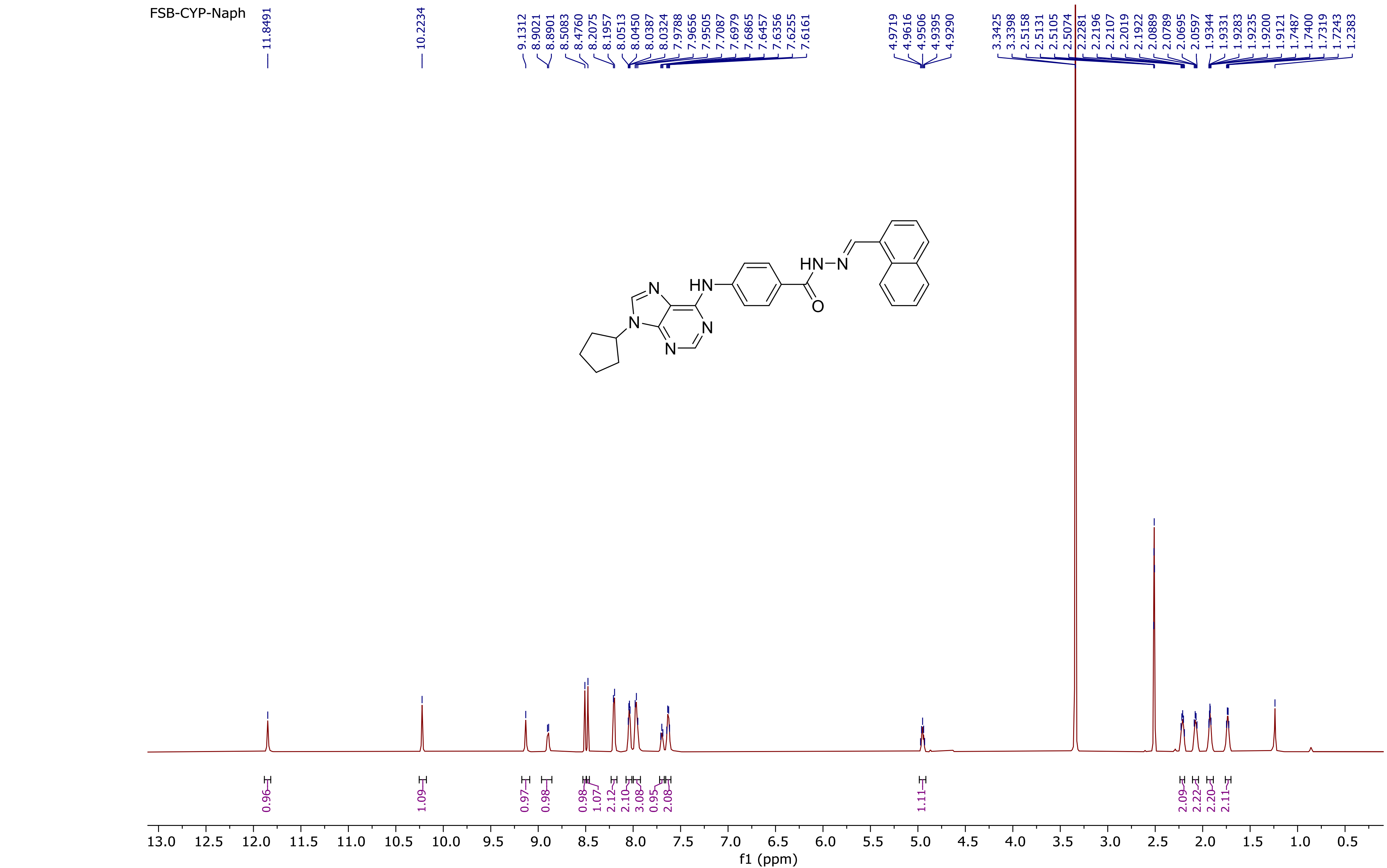

^{13}C-NMR spectra of compound **23b**

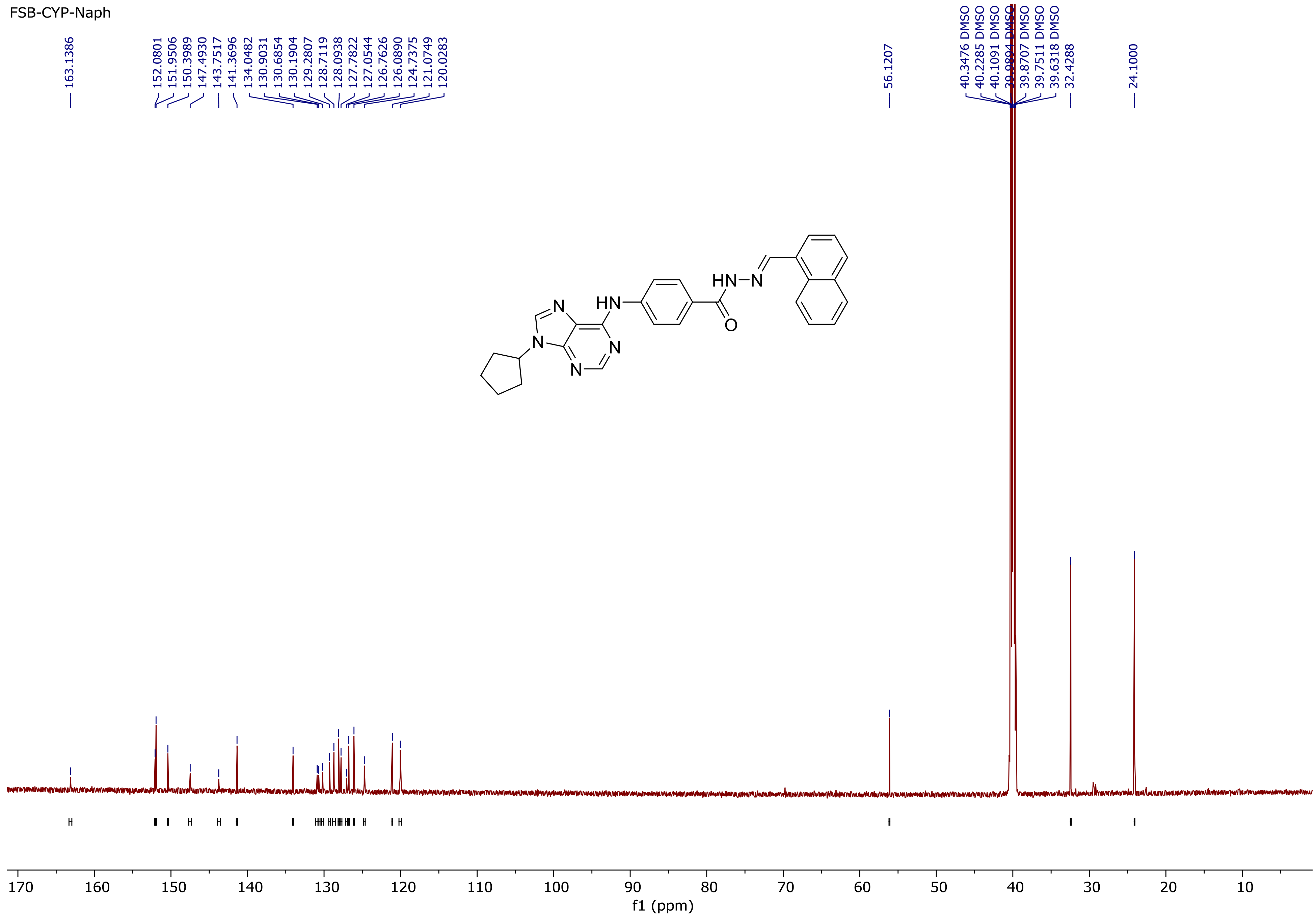

Mass spectra of compound **23b**

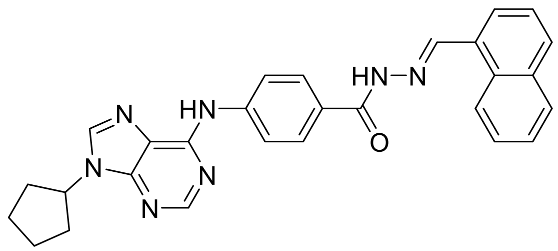

Chemical Formula: $C_{28}H_{25}N_7O$
Exact Mass: 475.2121

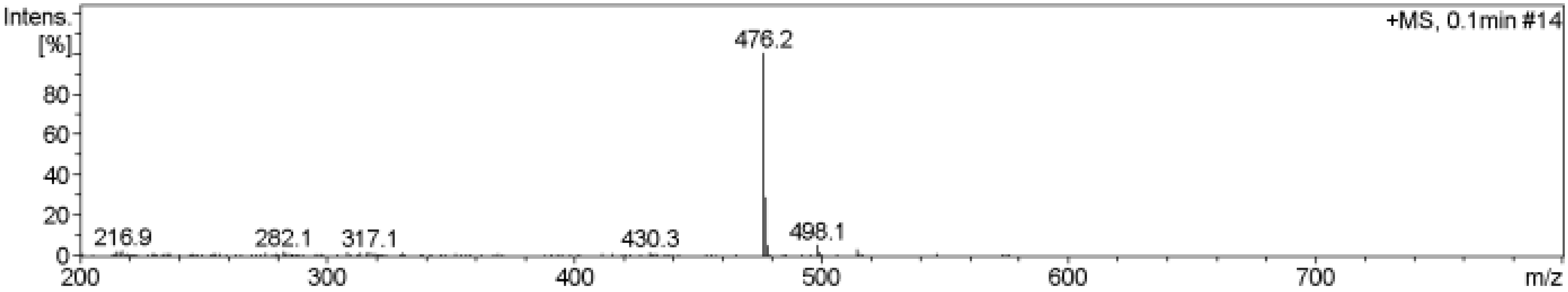

HRMS spectra of compound **23b**

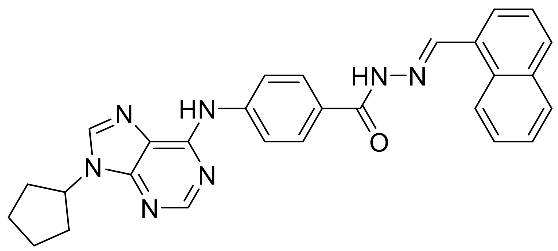

Chemical Formula: $C_{28}H_{25}N_7O$
Exact Mass: 475.2121

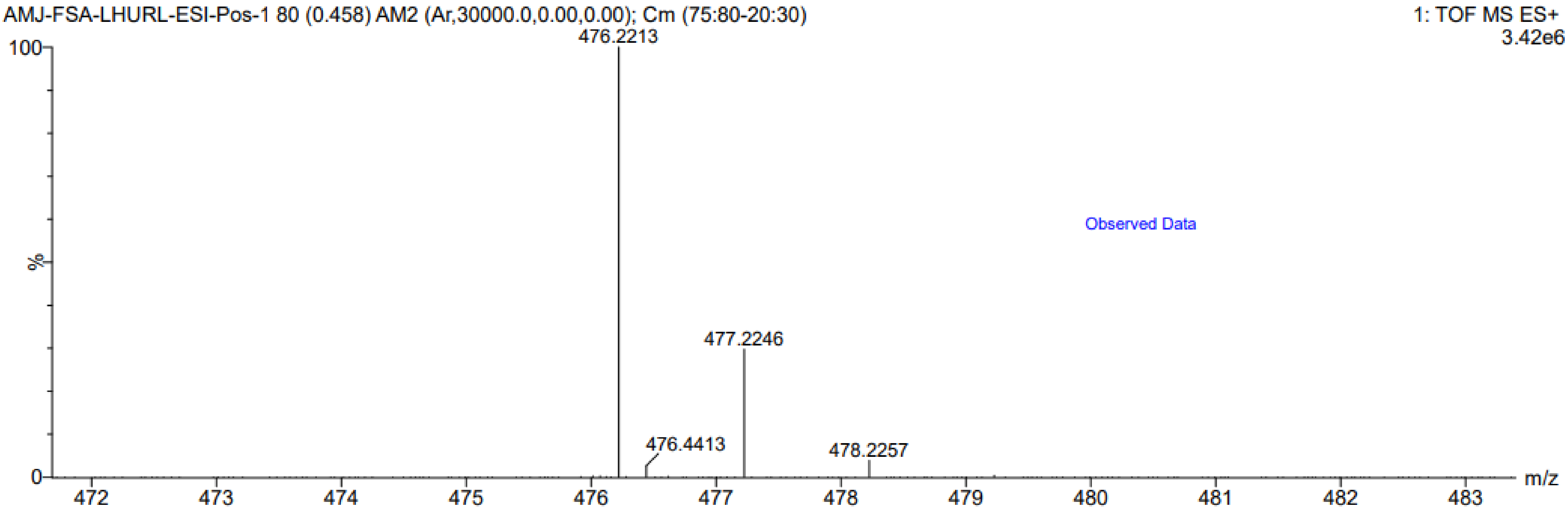

^{1}H-NMR spectra of compound **24b**

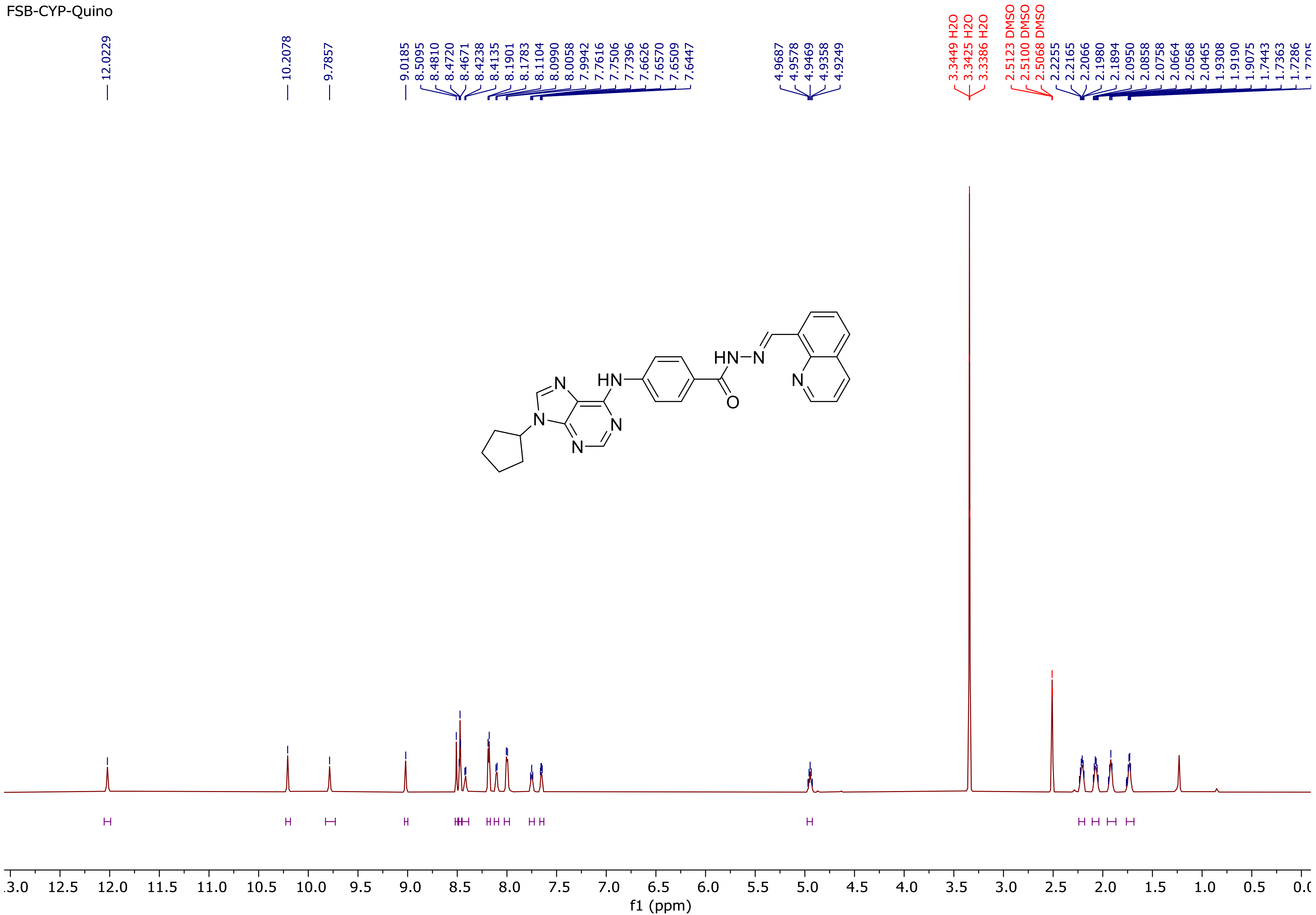

^{13}C-NMR spectra of compound **24b**

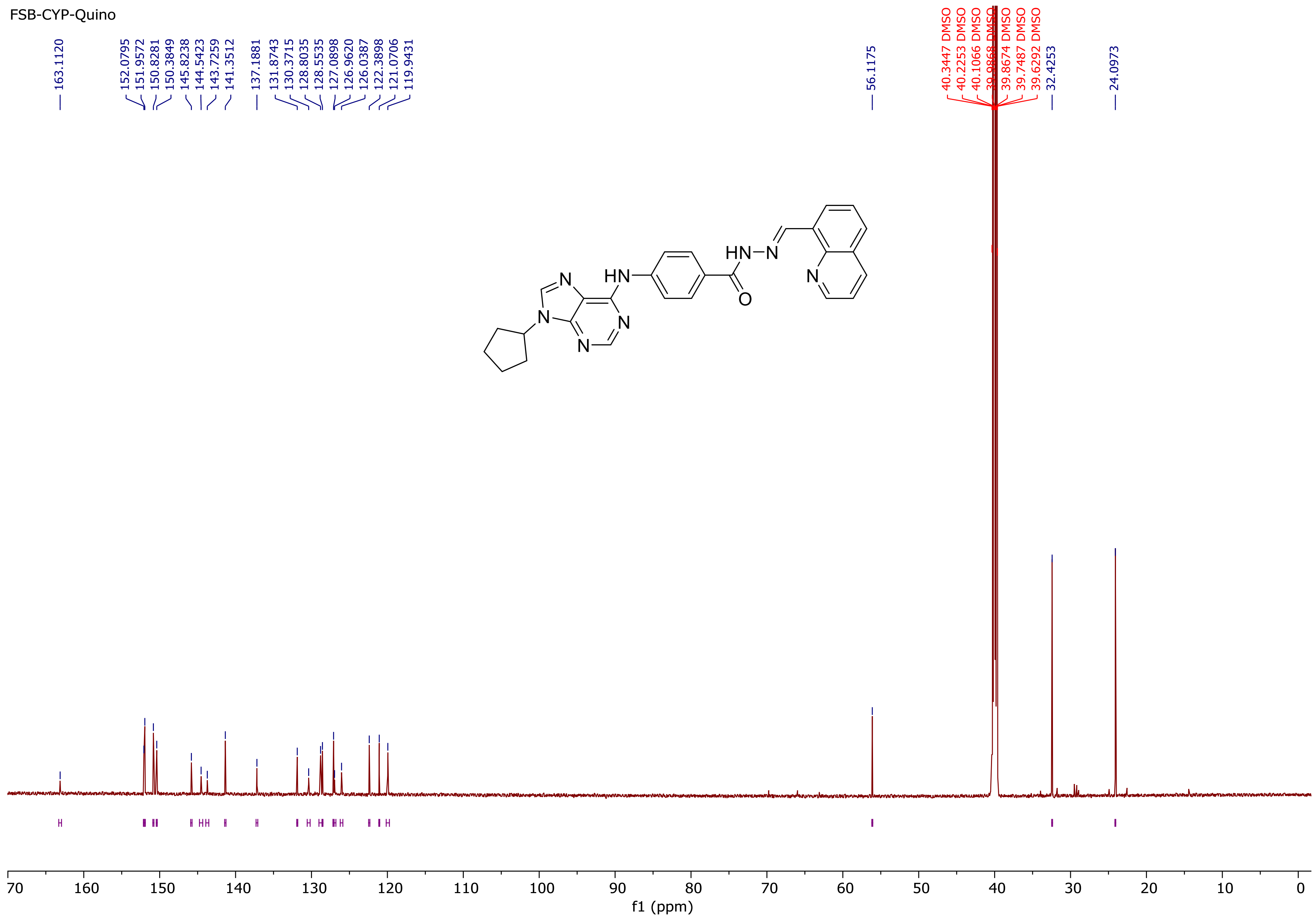

Mass spectra of compound **24b**

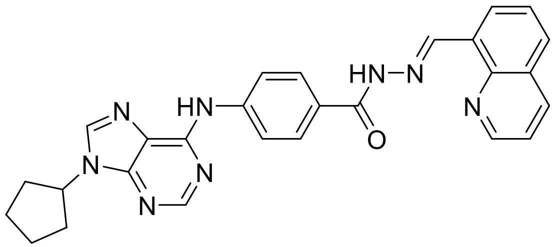

Chemical Formula: $C_{27}H_{24}N_8O$
Exact Mass: 476.2073

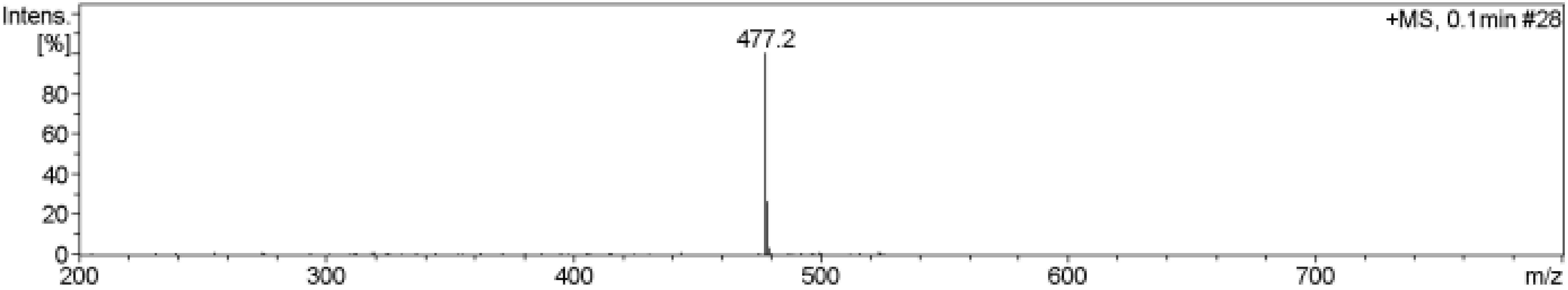

HRMS spectra of compound **24b**

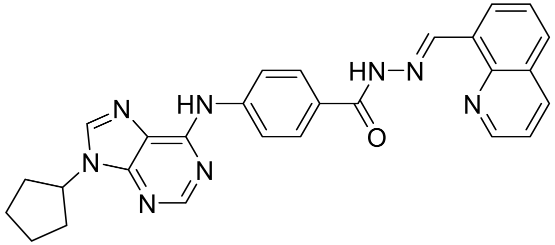

Chemical Formula: $C_{27}H_{24}N_8O$
Exact Mass: 476.2073

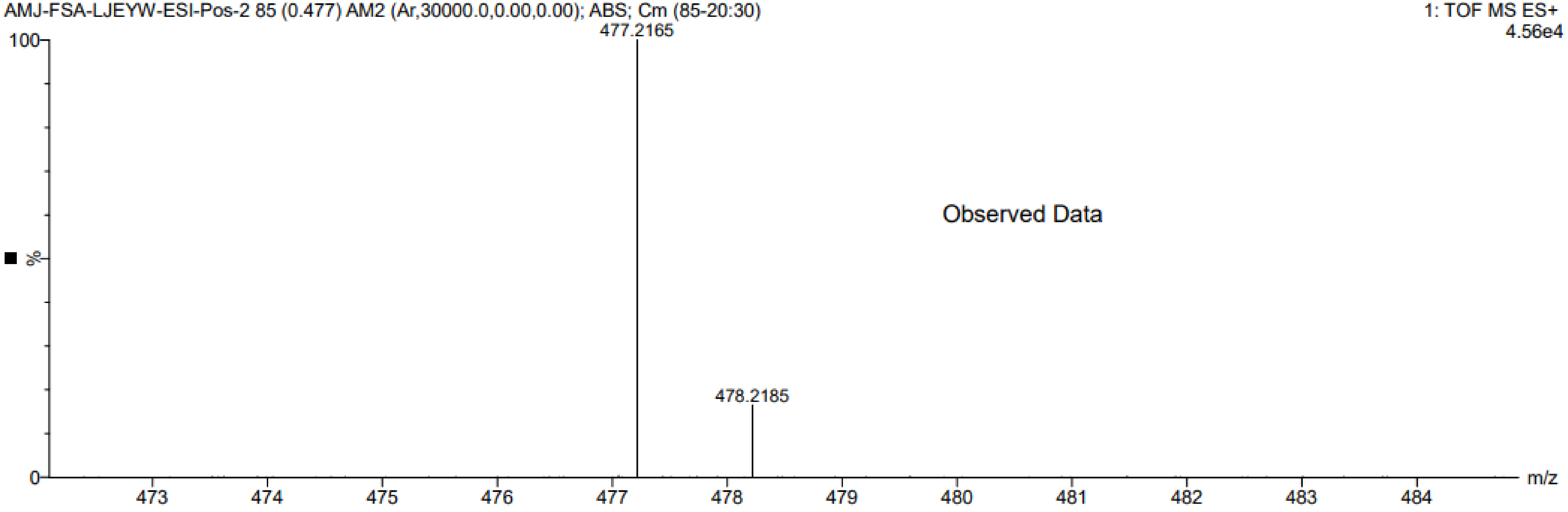

Molecular docking result:

Table S1: The binding scores of Lapatinib and (*E*)-4-((9*H*-purin-6-yl) amino)-*N′*-(substituted) -(benzylidene /naphthylidene) benzohydrazide derivatives (**6a-20a** and **23a**) within the EGFR and HER2 kinase domain.

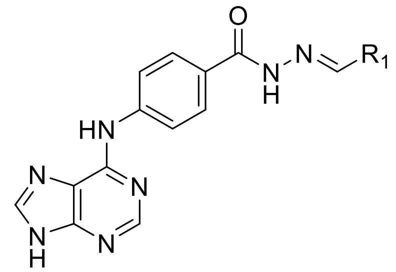

Compound #.	R1	Binding score (kcal/mol) EGFR	Binding score (kcal/mol) HER2	Compound #	R1	Binding score (kcal/mol) EGFR	Binding score (kcal/mol) HER2
**6a**	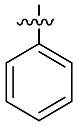	-9.2	-8.9	**15a**	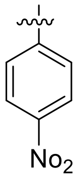	-9.4	-9.4
**7a**	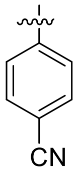	-9.2	-9.3	**16a**	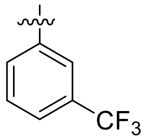	-10	-9.9
**8a**	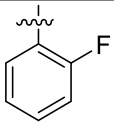	-9.3	-9.4	**17a**	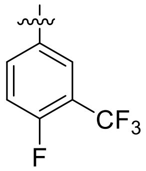	-10.1	-9.7
**9a**	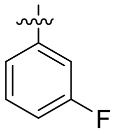	-9.4	-9.5	**18a**	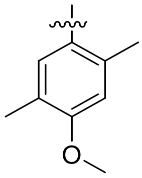	-9.6	-9.5
**10a**	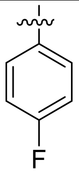	-9.3	-9.5	**19a**	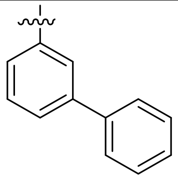	-11.0	-10.3

**11a**	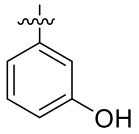	-9.3	-9.4	**20a**	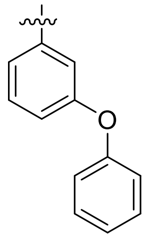	-10.9	-9.8
**12a**	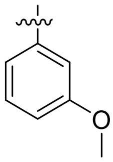	-9.1	-9.3	**23a**	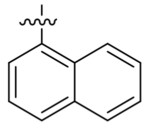	-10.2	-10.9
**13a**	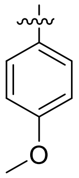	-9.2	-9.3	Lapatinib		-11.1	-10.3
**14a**	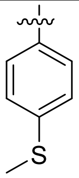	-9.3	-9.3				

Table S2. The binding scores of Lapatinib and (*E*)-4-((9-*cyclopentyl*-purin-6-yl) amino)-*N'*-(substituted) -(benzylidene /naphthylidene) benzohydrazide derivatives (**6b,11b,13b**, and **16b-24b**) within the EGFR and HER2 kinase domain.

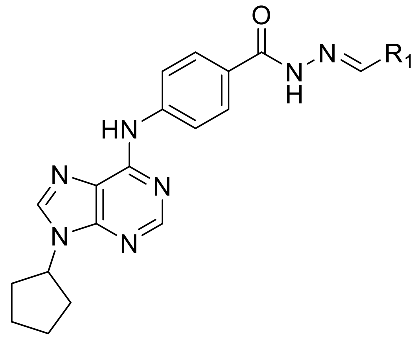

Compound #.		Binding score (kcal/mol)		Compound #.		Binding score (kcal/mol)	
	R_1	EGFR	HER2		R_1	EGFR	HER2
**6b**	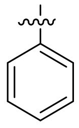	-9.8	-9.6	**20b**	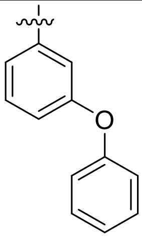	-10.4	-10.3

**11b**	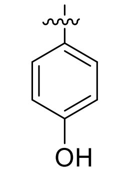	-9.5	-9.7	**21b**	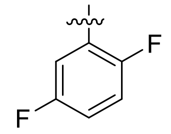	-10.6	-10.1
**13b**	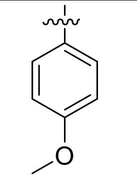	-9.9	-9.8	**22b**	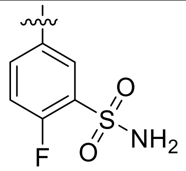	-11.0	-10.8
**16b**	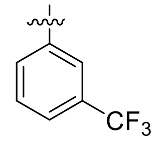	-10.6	-10	**23b**	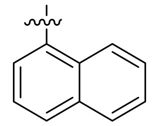	-10.3	-10.9
**17b**	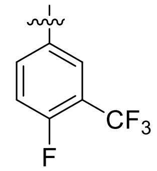	-11.0	-10	**24b**	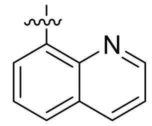	-10.5	-10.8
**18b**	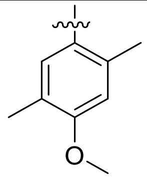	-9.9	-9.9	Lapatinib		-11.1	-10.3
**19b**	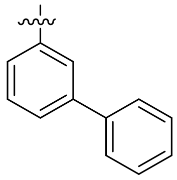	-11.7	-10.6				

The predicted mode of interaction of selected compounds inside the active site of EGFR kinase:

Table S3. Docking result of compounds **19a** and **22b** within EGFR and HER2 active sites (hydrogen bonds only).

Entry	Enzymes	Docking score (kcal/mol)	Interacting group	Amino acids	bond length Å
**19a**	EGFR	-11.0	N9 (purine)	Leu718	2.87
			NH (aniline)	Cys797	2.78
			Oxygen(amide)	Lys745	3.02
	HER2	-10.3	N7 (purine)	Cys805	3.07
**22b**	EGFR	-11.0	Oxygen(sulfonamide)	Thr854, Asp855	3.15, 2.70
			NH (sulfonamide)	Thr790	3.05
			NH (aniline)	Cys797, Asp800	3.08, 2.58
	HER2	-10.8	NH (sulfonamide)	Gly865, Glu770	2.48, 2.66
			NH (hydrazone)	Thr862	2.16
			Oxygen (amide)	Lys753	3.11

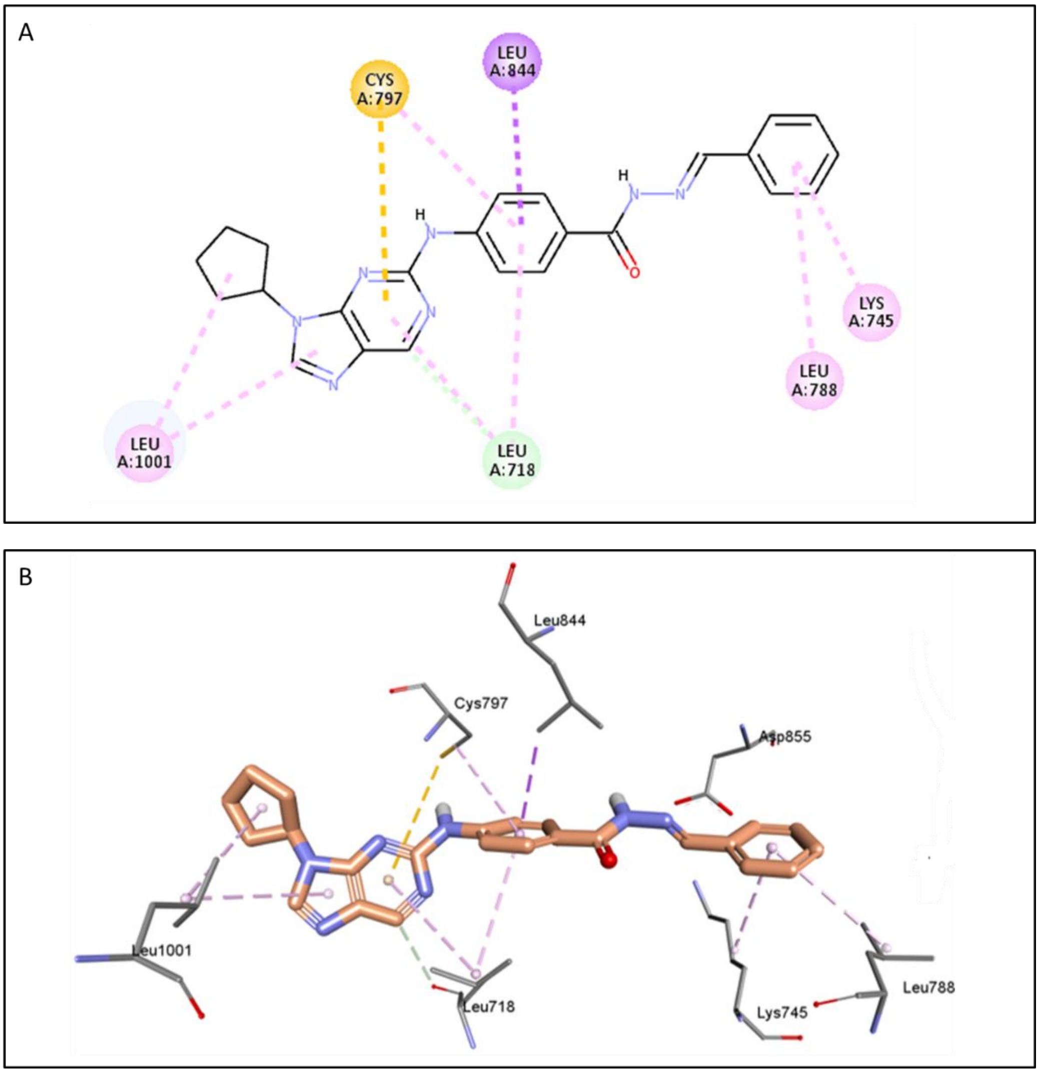

Figure S1: A) Two-Dimensional and B) Three-Dimensional predicted interaction patterns of compound **6b** inside the EGFR kinase domain.

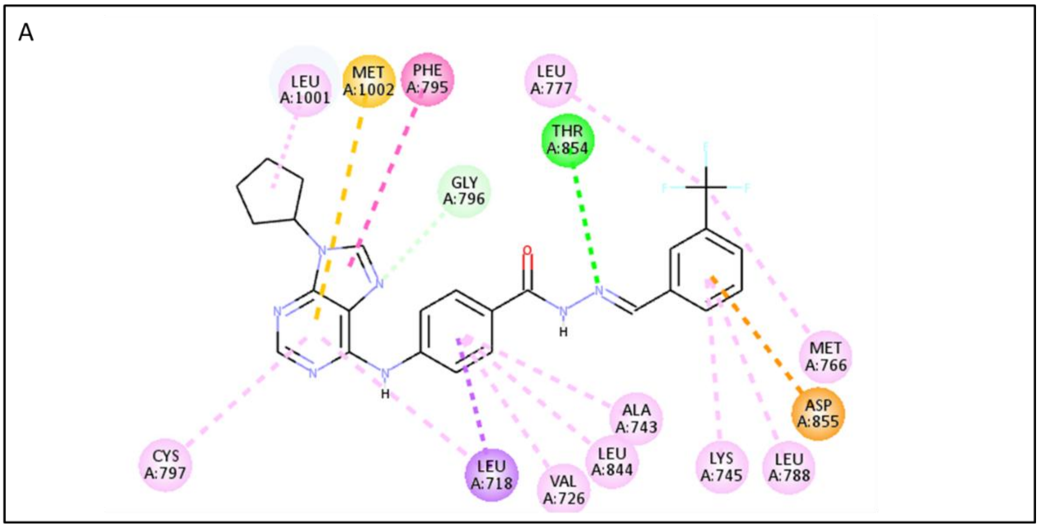

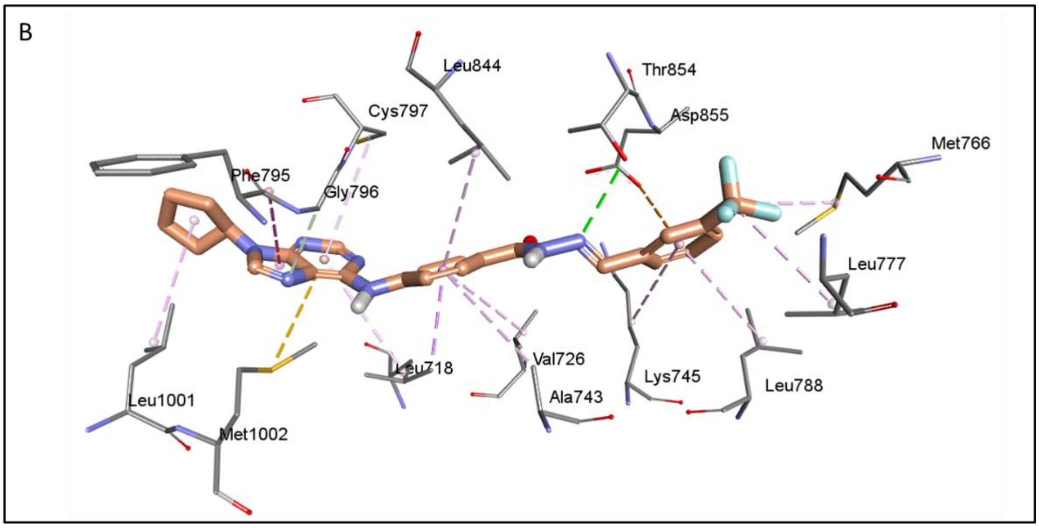

Figure S2: A) Two-Dimensional and B) Three-Dimensional predicted interaction patterns of compound **16b** inside the EGFR kinase domain.

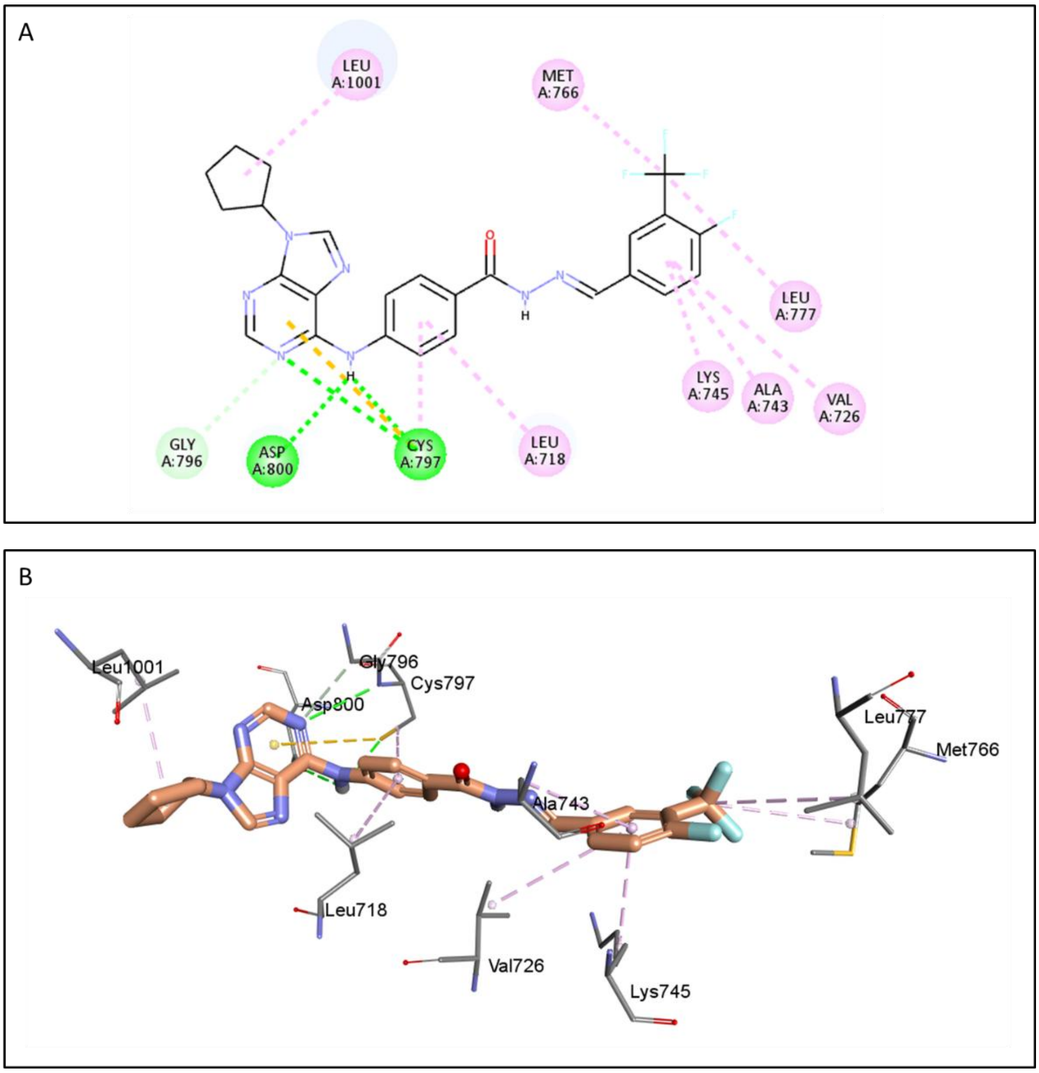

Figure S3: A) Two-Dimensional and B) Three-Dimensional predicted interaction patterns of compound **17b** inside the EGFR kinase domain.

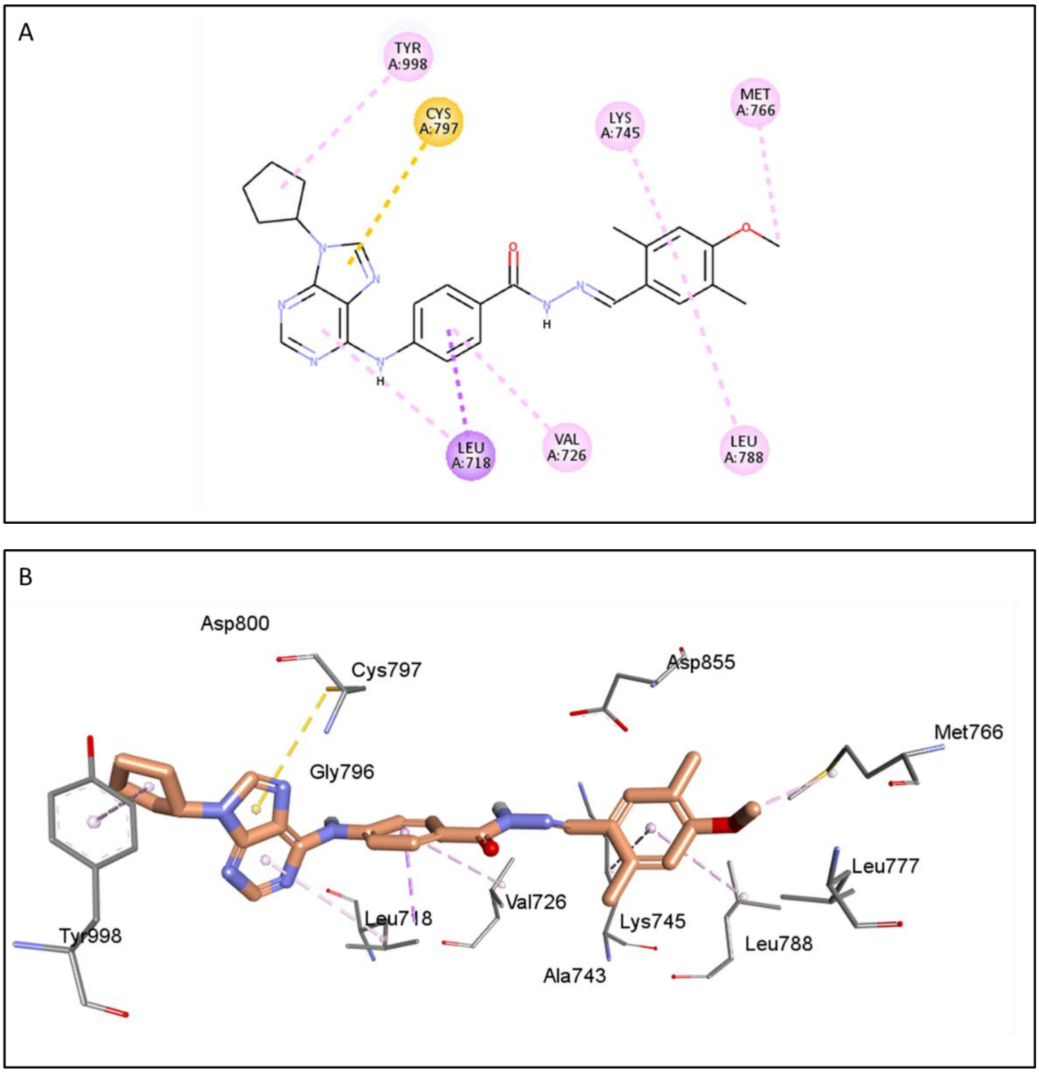

Figure S4: A) Two-Dimensional and B) Three-Dimensional predicted interaction patterns of compound **18b** inside the EGFR kinase domain.

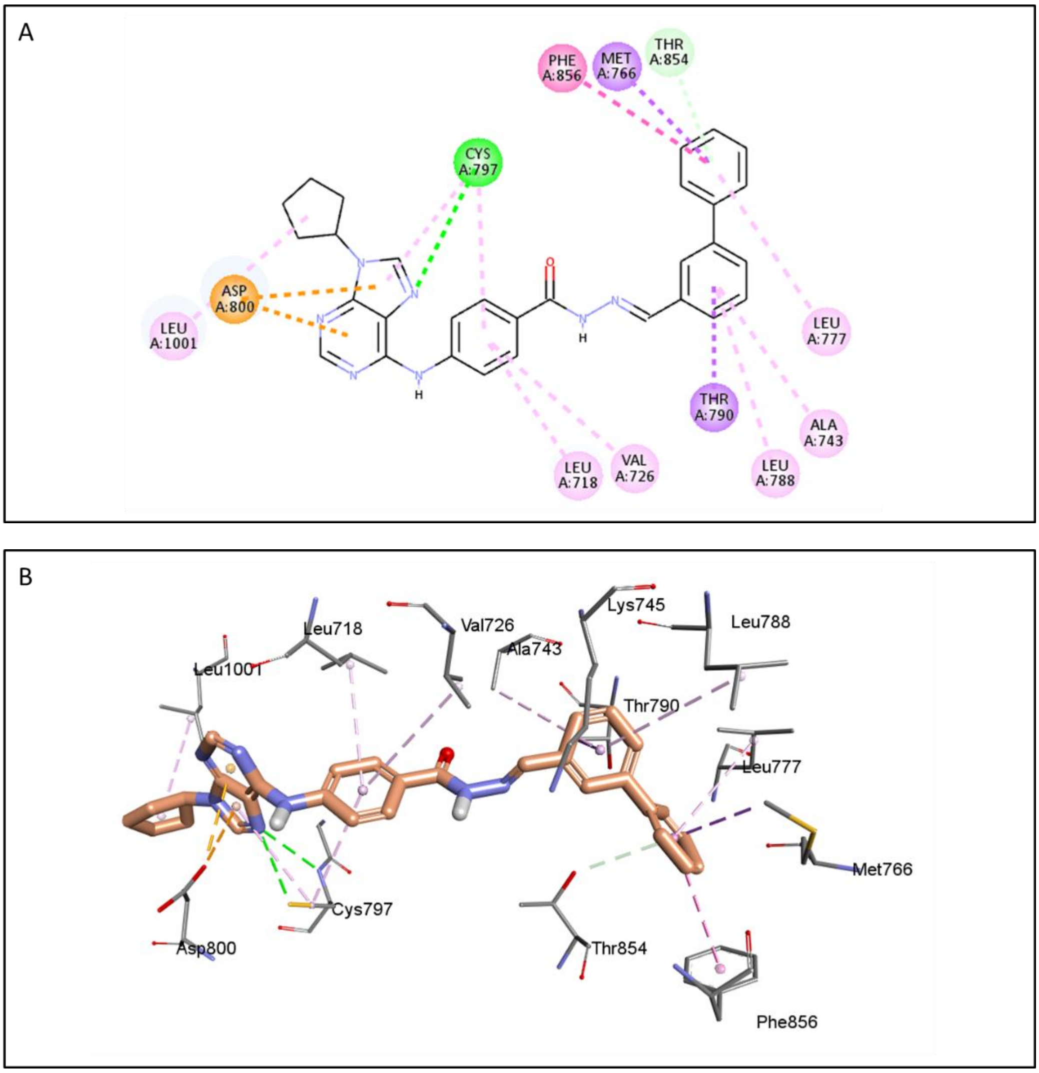

Figure S5: A) Two-Dimensional and B) Three-Dimensional predicted interaction patterns of compound **19b** inside the EGFR kinase domain.

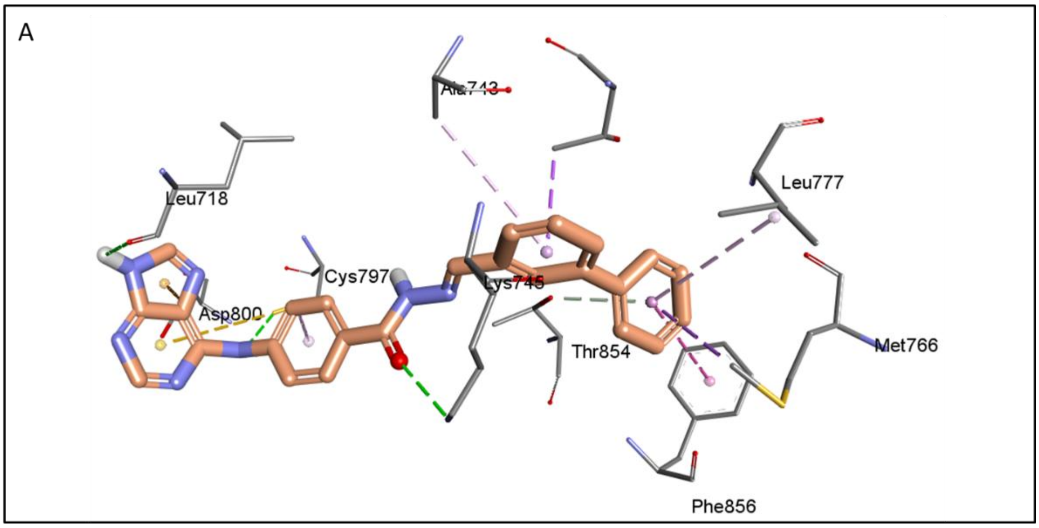

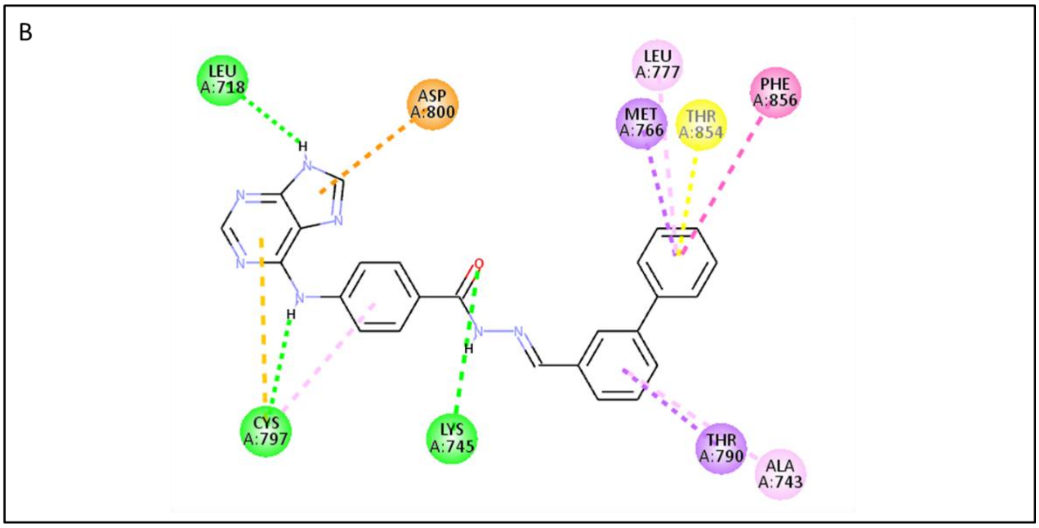

Figure S6: A) Two-Dimensional and B) Three-Dimensional predicted interaction patterns of compound **19a** inside the EGFR kinase domain.

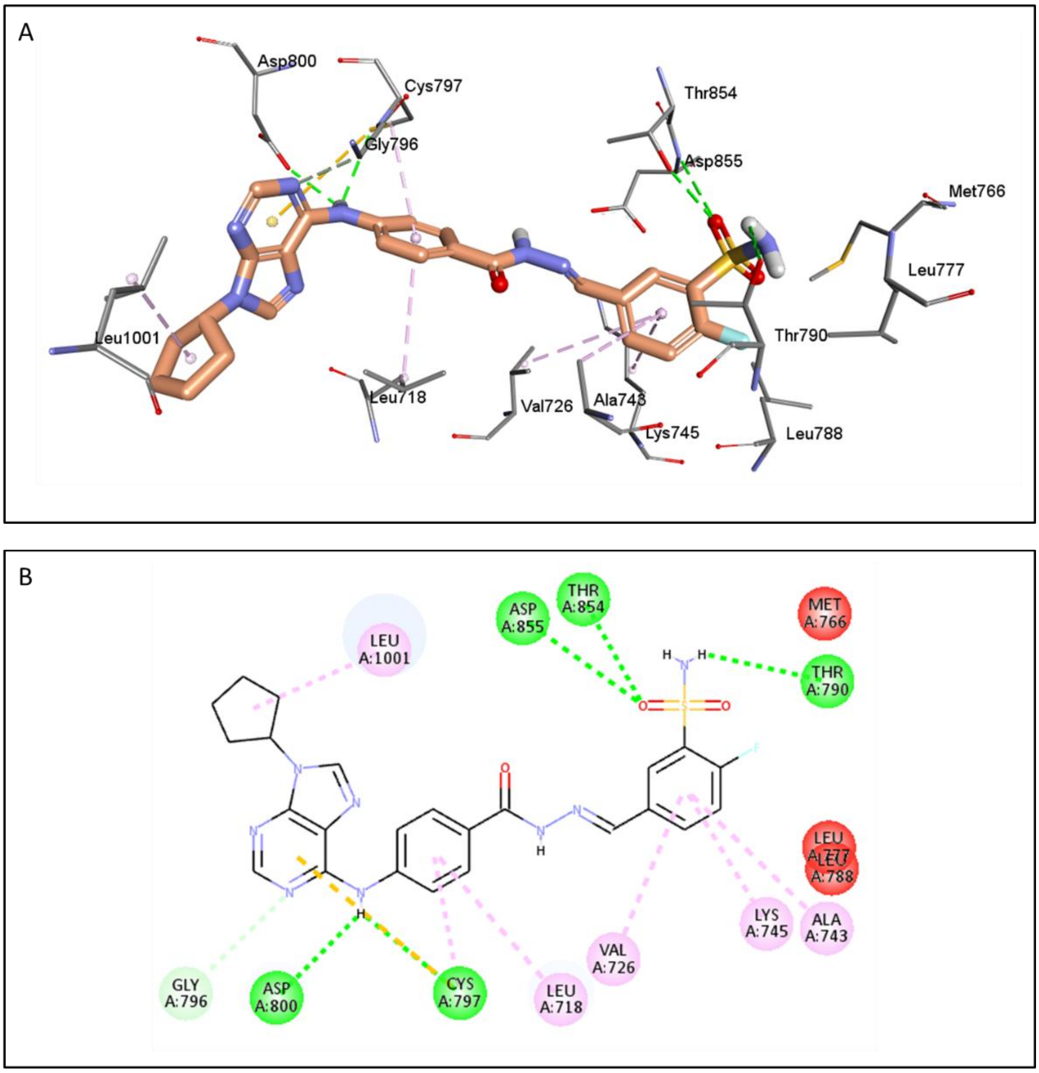

Figure S7: A) Two-Dimensional and B) Three-Dimensional predicted interaction patterns of compound **22b** inside the EGFR kinase domain.

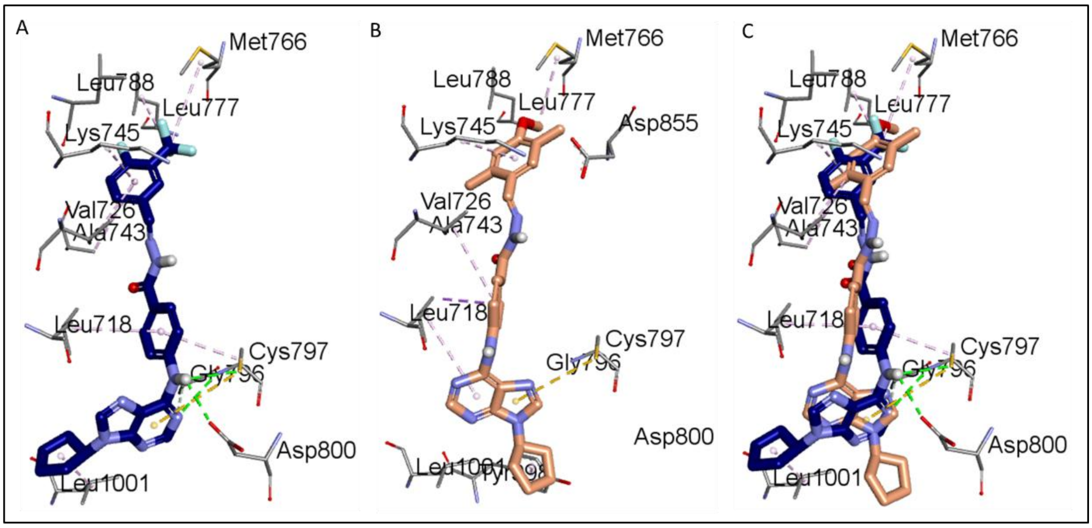

Figure S8: The predicted interaction mode of (A) **17b** and (B) **18b** within the EGFR (PDBID=1XKK) active site (C= overlay).
